# Supplementary material for: Performing arts as a health resource? An umbrella review of the health impacts of music and dance participation
Source: PLoS One. 2021 Jun 10;16(6):e0252956. doi: 10.1371/journal.pone.0252956 (PMC8191944; doi:10.1371/journal.pone.0252956)
Supplement: S1 Appendix — Additionally, details of excluded articles are included. (PDF) [file pone.0252956.s001.pdf]

Performing arts as a health resource? An umbrella review of the health impacts of music and dance participation

J. Matt McCrary, Emma Redding, Eckart Altenmüller

S1 Appendix

Table of Contents

Component 1 – Systematic review of systematic reviews of the health benefits of performing arts participation

Supplementary methods – GRADE Quality of evidence appraisal 3

Table S1. Specific outcome measures assigned to each health domain 5

Table S2. Umbrella review level evidence synthesis – all outcomes and GRADE Certainty of Evidence ratings 10

Table S3. Systematic review level evidence synthesis for each outcome measure from each included review of dance participation 20

Table S4. Systematic review level evidence synthesis for each outcome measure from each included review of music participation 34

Table S5. Summary of included systematic reviews 50

Table S6. GRADE publication bias ratings for included systematic reviews containing quantitative synthesis (meta-analysis) 58

Table S7. Details of primary studies in included systematic reviews of dance participation 59

Table S8. Details of primary studies in included systematic reviews of music participation 104

Table S9. GRADE Certainty of Evidence ratings for each outcome measure from primary randomized controlled trials of dance participation contained in included reviews 161

Table S10. GRADE Certainty of Evidence ratings for each outcome measure from primary non-randomized studies of dance participation contained in included reviews 181

Table S11. GRADE Certainty of Evidence ratings for each outcome measure from primary randomized controlled trials of music participation contained in included reviews 194

Table S12. GRADE Certainty of Evidence ratings for each outcome measure from primary non-randomized studies of music participation contained in included reviews 210

Table S13. Review articles excluded after full-text review, with reasons 245

Component 2 – Systematic review of observational studies investigating the impact of performing arts participation on mortality and non-communicable disease risk

Table S14. Details of included studies evaluating the impact of performing arts participation on mortality and non-communicable disease risk *247*

Table S15. GRADE Certainty of Evidence ratings for each outcome measure from included studies evaluating the impact of performing arts participation on mortality and non-communicable disease risk *249*

Table S16. Studies of mortality and non-communicable disease risk excluded after full-text review *251*

Component 3 – Systematic review of studies of heart rate responses to performing arts participation

Table S17. Details of included studies reporting mean heart rate data during dance participation *252*

Table S18. Details of included studies reporting mean heart rate data during music participation *259*

Table S19. Studies of the physical demands of performing arts participation excluded after full-text review, with reasons *262*

Reference list of included reviews and studies *(Components 1-3)* *269*

Supplementary Methods – GRADE Quality of Evidence Appraisal

GRADE quality of evidence determinations are based on study design and assessments of five factors which may negatively impact evidence quality: risk of bias; inconsistency; indirectness; imprecision; publication bias. Studies can also be rated higher if a large effect, dose response gradient and/or robust control for all plausible confounding is present. Randomized controlled trials begin the rating process as high-quality evidence, with observational and non-randomized studies beginning as low-quality evidence. For this review, assessment of these factors were conducted in the following manner:

Study limitations / risk of bias – assessed for each outcome for each primary study meeting umbrella review inclusion criteria in included reviews, as per GRADE and Cochrane guidelines.[1,2] Specific assessment criteria are per GRADE[3] and detailed in Tables S9-S12.

Imprecision – Omitted. Given the general, descriptive and imprecise nature of outcome classifications used in this review (positive, negative, no effect, unclear), assessment of imprecision is irrelevant to evidence quality appraisals in this review.

Indirectness – The impact of using surrogate outcomes for health impacts of interest was determined for each outcome at the individual study level. Indirectness related to population, intervention and head-to-head comparisons were addressed by umbrella review inclusion/ exclusion criteria and/or irrelevant to this review.[4]

Inconsistency – Omitted. The descriptive methods of outcome classification used in this review serve in lieu of analyses of inconsistency, which are based on analyses of heterogeneity within a pooled effect.[5]

Publication Bias – Assessed for included reviews presenting quantitively synthesized results (meta-analysis) only. Assessment of publications bias conducted using the validated criteria developed by Meader et al. for appraisal of publication bias in systematic reviews within the GRADE framework.[6] Rigorous assessment of publication bias for narratively synthesized evidence was precluded by the restriction of inclusion criteria to systematic reviews and further

potential exclusion of primary studies from included reviews for not meeting umbrella review inclusion criteria.

Up-rating – Conducted as appropriate per GRADE guidelines.[7]

Appraisals were conducted, except for publication bias, first for each outcome at the primary study level. When GRADE was used to assess quality of evidence of primary studies in included reviews, these GRADE ratings were also included in this umbrella review. Otherwise, all GRADE ratings were determined for the purposes of this review.

GRADE ratings for each outcome at the primary study level were then synthesized for each included review, with a GRADE rating assigned to each outcome at the review level. Where applicable, down rating of evidence quality for publication bias was conducted at this stage. GRADE ratings for each outcome at the review level were then further synthesized and a GRADE rating was assigned to each outcome at the umbrella review level.

References

1. Guyatt GH, Oxman AD, Vist GE, et al. GRADE: an emerging consensus on rating quality of evidence and strength of recommendations. Bmj 2008;336:924-6.
2. Higgins JP, Thomas J, Chandler J, et al. Cochrane handbook for systematic reviews of interventions: John Wiley & Sons; 2019.
3. Guyatt GH, Oxman AD, Vist G, et al. GRADE guidelines: 4. Rating the quality of evidence—study limitations (risk of bias). Journal of clinical epidemiology 2011;64:407-15.
4. Guyatt GH, Oxman AD, Kunz R, et al. GRADE guidelines: 8. Rating the quality of evidence—indirectness. Journal of clinical epidemiology 2011;64:1303-10.
5. Guyatt GH, Oxman AD, Kunz R, et al. GRADE guidelines 6. Rating the quality of evidence—imprecision. Journal of clinical epidemiology 2011;64:1283-93.
6. Meader N, King K, Llewellyn A, et al. A checklist designed to aid consistency and reproducibility of GRADE assessments: development and pilot validation. Systematic reviews 2014;3:1-9.
7. Guyatt GH, Oxman AD, Sultan S, et al. GRADE guidelines: 9. Rating up the quality of evidence. Journal of clinical epidemiology 2011;64:1311-6.

Table S1. Specific outcome measures assigned to each health domain

Domain	*Outcome*
Auditory	Auditory processing
	Auditory working memory
	Hearing threshold
	Melodic contour identification
	Pitch discrimination
	Speech in noise
	Vocal emotion identification
Autonomic Tone	Adrenaline
	Noradrenaline
Blood pressure	Blood pressure
Body Composition	% body fat
	Body composition (BMI, body composition)
	Body mass
	Skinfold measurements
	Total fat mass
	Waist circumference
Bone Health	Bone mineral density
Cognitive	Attention
	BDNF
	Brain structure/plasticity
	Cognitive function/Global cognition
	Confusion
	Executive function
	Fluid intelligence
	IQ
	IQ (nonverbal)
	Memory (long-/short-term, working)
	Mental status (Blessed Information Memory Concentration test)
	Mental flexibility
	Perceptual speed
	Spatial ability/reasoning
	Vocabulary
Developmental	Foot anthropometrics
	Growth
	Musculoskeletal development
	Puberty onset
Educational	Language
	Mathematics
	Phonological awareness
	Reading
	Writing
Endothelial function	Cortisol

	Cortisol:DHEA ratio
	DHEA
Glucose/Insulin	Glucose
	Insulin
Immune function	C-Reactive Protein
	IFN-alpha
	IL-2
	IL-6
	Immunological profile
	Immunoglobulin A
	LAK cell activity
	NK cell activity
	TNF-alpha
	White blood cell count
Lipid lipoprotein profile	HbA1c
	HDL cholesterol
	Total cholesterol
	Triglycerides
Mental health	Anxiety
	Burnout
	Compassion fatigue
	Depersonalization
	Depression
	Emotional exhaustion
	Emotional regulation
	Mood
	Psychological wellbeing/distress
	Self-actualization
	Self-esteem
	Self-perception
Non-communicable disease risk	All-cause mortality
	Asthma
	Cardiovascular disease
	Dementia
	Diabetes
	Hypertension
	Metabolic syndrome
	Osteoarthritis
	Rheumatoid arthritis
	Tinnitus
Physical fitness	Abdominal strength/endurance (sit ups)
	Cardiovascular capacity (VO2 max)
	Cardiovascular/cardiorespiratory fitness (Physical Working Capacity 130, Senior fitness test, VO2 peak)

	Endurance (6-minute walk test)
	Endurance (muscular - knee extensions)
	Endurance (1-mile walk/run)
	Heart rate variability
	Power (muscular/aerobic)
	Peak ventilation
	Respiratory exchange ratio
	Resting HR
	Strength
	VO2 at anaerobic threshold
Physical function	Agility
	Balance
	Falls
	Fine motor performance
	Flexibility/range of motion
	Gait (Gaitrite)
	Gait speed
	Lung function
	Mobility (timed up & go; sit to stand)
	Mobility (Step test)
	Oestrogen
	Proprioception
	Reaction time
	Sit and Reach (flexibility)
	Tactile performance
Self-reported health/wellbeing	Alcohol Consumption
	Balance confidence
	Body satisfaction (Cathexis)
	Daytime somnolence
	Drowsiness
	Fatigue
	Fear of falling
	Functional autonomy
	Health responsibility
	Health system utilization
	Lack of coordination
	Life satisfaction/Quality of Life/Wellbeing
	Loneliness
	Medication usage
	Morale
	Negative affect
	Nutrition
	Overall/general health

	Perceived self-efficacy
	Personal accomplishment
	Positive affect
	Quality of life
	Sexual activity
	Sleep quality
	Smoking
	Somatic symptoms
	Spiritual growth/wellbeing
	Stress
	Stress management
	Vigor
	Wellbeing
Social functioning	Aggression
	Anger
	Job engagement
	Social functioning
	Social activity level
	Social anxiety
	Social Support (perceived)

Table S2. Evidence Synthesis – All GRADE Certainty of Evidence Ratings

Domain	Outcome	GRADE	Effect	# reviews	# studies/ outcomes	Sex	Age group	Music/ Dance?	Style/instrument	Participation length
Auditory	Auditory processing	Moderate	Positive	1	13	Mixed	Adults	Music	Instrumental	Sustained
	Auditory working memory	Very Low	Mixed (positive, no effect)	1	2	Mixed	Adults	Music	Piano & violin, unspecified	Sustained
	Hearing threshold	Very Low	Negative	1	1	Mixed	Adults, Older adults	Music	Singing	Sustained
	Melodic contour identification	Low	Positive	1	1	Mixed	Adults	Music	Instrumental	Sustained
	Pitch discrimination	Moderate	Positive	1	7	Mixed	Adults	Music	Instrumental, unspecified	Sustained
	Speech in noise	Moderate	Positive	1	21	Mixed	Children, Adolescents, Adults	Music	Instrumental, vocal, unspecified	Sustained
	Vocal emotion identification	Low	Positive	1	1	Mixed	Adults	Music	Instrumental	Sustained
Autonomic Tone	Adrenaline	Very low	No effect	1	1	Mixed	Adults, Older Adults	Music	Drums	Acute
	Noradrenaline	Very low	Mixed (positive, no effect)	1	1	Mixed	Adults, Older adults	Music	Drums	Acute
Blood pressure	Blood pressure	Very Low	Mixed (positive, no effect)	3	8	Mixed	Adolescents, Adults	Dance	Ballroom, Dance Team, Zumba	Sustained
	Blood pressure	Very Low	Mixed (positive, no effect)	2	2	Mixed	Adults	Music	Drums, Singing	Acute
Body Composition	% body fat	Very Low	Mixed (positive, no effect)	2	6	Female	Adolescents, Adults	Dance	Dance team, Zumba	Sustained
	Body composition (BMI, body composition)	Very Low	Mixed (positive, no effect)	5	18	Mixed	Children, adolescents, adults, older adults	Dance	Aerobic dance, African Dance, Ballet, Ballroom, Bhangra, Greek traditional, Hip hop, Zumba	Sustained
	Body mass	Very low	Mixed (positive, no effect)	3	16	Mixed	Children, Adolescents, Adults, Older Adults	Dance	Aerobic dance, Ballroom, Dance team, Greek folk/traditional dance, Zumba	Sustained
	Skinfold measurements	Moderate	Positive	1	3	Female, Unspecified	Children, Adolescents, Adults	Dance	Aerobic dance	Sustained
	Total fat mass	Moderate	Positive	1	4	Female	Children, Adolescents, Adults	Dance	Aerobic dance, Zumba	Sustained

	Waist circumference	Very Low	Mixed (positive, no effect)	3	6	Mixed	Children, Adults	Dance	Aerobic dance, African dance, Ballroom, Greek folk/traditional dance, Hip-hop, Zumba	Sustained
Bone Health	Bone mineral density	Very Low	Mixed (positive, no effect)	3	6	Female	Adults, Older Adults	Dance	Aerobic dance, Folk, Waltz, Zumba	Sustained
	Bone mineral density	Low	Positive	1	2	Female	Children, Adolescents	Dance	Ballet	Sustained
Cognitive	Attention (selective, visual)	Low	No effect	1	3	Mixed	Adults	Music	Instrumental, Unspecified	Sustained
	Attention/'Mental status'	Moderate	Positive	1	2	Mixed	Older adults	Dance	Agilando, Multiple styles – line/jazz/rock'n'roll/square	Sustained
	BDNF	Moderate	Positive	1	1	Mixed	Older adults	Dance	Multiple styles – line/jazz/rock'n'roll/square	Sustained
	Brain structure/plasticity	Moderate	Positive	1	3	Mixed	Adults, Older adults	Dance	Multiple styles – line/jazz/rock'n'roll/square	Sustained
	Cognitive function/Global cognition	High	Positive	2	10	Mixed	Older adults	Dance	Agilando, Ballroom, Jazz, Latin, Tango, Square dance	Sustained
	Confusion	Very Low	Mixed (positive, no effect)	2	2	Mixed	Adults, Older Adults	Music	Drums	Acute
	Executive function	Low	No effect	1	1	Unspecified	Adults	Music	Instrumental	Unclear
	Executive function	High	No effect	3	11	Mixed	Older adults	Dance	Ballroom, Contemporary, Folk, Latin, Social, Tango, Waltz	Sustained
	Fluid intelligence	Low	No effect	1	2	Mixed	Adults, Older adults	Dance	Agilando, unspecified	Sustained
	IQ	Moderate	Positive	2	5	Mixed	Children, Adolescents, Adults	Music	Instrumental, Music education	Sustained
	IQ (nonverbal)	Low	No effect	2	9	Mixed	Children, Adolescents, Adults	Music	Instrumental, Music education	Sustained
	IQ (nonverbal)	Low	No effect	1	1	Mixed	Older adults	Dance	Agilando	Sustained
	Memory	Very Low	Mixed (positive, no effect	3	6	Mixed	Older adults	Dance	Ballroom, Social, Waltz	Sustained
	Memory (long-/short-term, working)	Moderate	Positive	1	42	Mixed	Adults	Music	Instrumental	Sustained
	Mental flexibility	Low	Positive	1	1	Unspecified	Adults	Music	Instrumental	Sustained
	Mental status (Blessed Information	Low	No effect	1	1	Mixed	Older adults	Dance	Social	Sustained

	Memory Concentration test)									
	Perceptual speed	High	No effect	1	2	Mixed	Adults, Older adults	Dance	Social, unspecified	Sustained
	Spatial ability/reasoning	High	Positive	1	23	Mixed	Children, Adolescents	Music	Music education (general, Kodaly, Kindermusik, snare drum, piano, vocal)	Sustained
	Vocabulary	High	No effect	1	1	Mixed	Older adults	Dance	Social	Sustained
Developmental	Foot anthropometrics	Low	No effect	1	1	Female	Adolescents	Dance	Ballet	Sustained
	Growth	Very Low	Mixed	1	1	Female	Adolescents	Dance	Ballet	Sustained
	Musculoskeletal development	Low	No effect	1	1	Female	Children, Adolescents	Dance	Ballet	Sustained
	Puberty onset	Very Low	Negative	1	1	Female	Children	Dance	Ballet	Sustained
Educational	Language	Very Low	Mixed (negative, no effect)	2	2	Mixed	Children, Adolescents	Music	Music education, instrumental	Sustained
	Mathematics	Very Low	Mixed (positive, no effect)	2	26	Mixed	Children, Adolescents	Music	Music education (general, Kodaly, Suzuki, piano)	Sustained
	Phonological awareness	Very Low	Positive	2	18	Mixed	Children	Music	Music education (general, rhythm intervention, music & movement)	Sustained
	Reading	Very Low	Mixed (positive, no effect)	2	37	Mixed	Children, Adolescents	Music	Orchestra, music education (general, instrumental, vocal, Suzuki)	Sustained
	Writing	Very Low	Mixed (positive, no effect)	1	2	Mixed	Children, Adolescents	Music	Music education (general)	Sustained
Endothelial function / Stress response	Cortisol	Very low	Mixed (positive, no effect)	4	7	Mixed	Adolescents, Adults	Music	Singing, music education, drums	Acute
	Cortisol:DHEA ratio	Low	No effect	2	2	Mixed	Adults	Music	Singing, Drums	Acute
	DHEA	Low	No effect	2	2	Mixed	Adults	Music	Singing, Drums	Acute
Glucose/Insulin	Glucose	High	No effect	3	6	Mixed	Adults	Dance	Aerobic Dance, Ballroom, Bhangra, Zumba	Sustained
	Insulin	High	No effect	2	2	Female	Adults	Dance	Bhangra, Zumba	Sustained
Inflammation / Immune function	CRP	Low	No effect	1	1	Female	Adults	Dance	Zumba	Sustained
	IFN-alpha	Very Low	Mixed (positive, no effect)	1	2	Mixed	Adults, Older adults	Music	Drums	Acute
	IL-2	Low	No effect	1	2	Mixed	Adults, Older adults	Music	Drums	Acute

	IL-6	Low	Unclear	1	1	Female	Adults	Dance	Zumba	Sustained
	Immunoglobulin A	High	Positive	3	4	Mixed	Adults, Older adults	Music	Singing, Drums	Acute
	Immunological profile	High	Positive	2	2	Mixed	Adults	Music	Singing, Drums	Acute
	LAK cell activity	Low	Positive	1	1	Mixed	Adults	Music	Drums	Acute
	NK cell activity	Very Low	Mixed (positive, no effect	2	3	Mixed	Adults, Older adults	Music	Drums	Acute
	TNF-alpha	Very Low	Mixed (positive, no effect)	1	1	Mixed	Adults	Music	Singing	Acute
	White blood cell count	Low	No effect	1	2	Mixed	Adults, Older adults	Music	Drums	Acute
	White blood cell count	Low	Negative	1	1	Female	Adults	Dance	Zumba	Sustained
Lipid lipoprotein profile	HbA1c	Very Low	No effect	1	1	Female	Adults	Dance	Zumba	Sustained
	HDL cholesterol	Very Low	Mixed (positive, no effect	2	6	Mixed	Adults	Dance	Aerobic dance, Ballroom, Waltz, Zumba	Sustained
	Total cholesterol	Very Low	Mixed (positive, no effect)	3	7	Mixed	Adults	Dance	Aerobic dance, Ballroom, Waltz, Zumba	Sustained
	Triglycerides	Very Low	Mixed (positive, no effect)	3	7	Mixed	Adults	Dance	Aerobic dance, Ballroom, Waltz, Zumba	Sustained
Mental health	Anxiety	Very Low	Mixed (positive, no effect)	6	9	Mixed	Adults, older adults	Music	Drums, Singing	Acute/Sustained
	Anxiety	Low	Positive	2	2	Mixed	Adults, Older Adults	Dance	Greek traditional dance, Modern	Acute/Sustained
	Burnout	Low	Positive	1	1	Mixed	Adults	Music	Drums	Sustained
	Compassion fatigue	Very Low	No effect	1	1	Mixed	Adults	Music	Instrumental	Sustained
	Depersonalization	Low	No effect	1	1	Mixed	Adults	Music	Drums	Sustained
	Depression	Very Low	Mixed (positive, no effect)	5	9	Mixed	Adolescents, Adults, Older Adults	Music	Drums, keyboard, singing	Acute/Sustained
	Depression	High	No effect	1	1	Mixed	Older adults	Dance	Turkish folk dance, Jazz, Social	Sustained
	Emotional exhaustion	Low	Positive	1	1	Mixed	Adults	Music	Drums	Sustained
	Emotional regulation	Low	Positive	1	14	Mixed	Adults	Music	Singing, piano, guitar	Acute

	Mood	Very Low	Mixed (positive, no effect)	6	10	Mixed	Adults, Older Adults	Music	Drums, Singing	Acute/Sustained
	Mood	Moderate	Positive	1	1	Mixed	Adults	Dance	Hip hop	Acute
	Psychological wellbeing/ distress	Low	Positive	1	1	Mixed	Older adults	Dance	Greek traditional dance	Acute
	Self-actualization	Very Low	Unclear	1	1	Mixed	Older adults	Music	Singing	Unspecified
	Self-esteem	Very Low	Mixed (positive, no effect)	2	2	Mixed	Adolescents	Music	Guitar, Music Education (general)	Sustained
	Self-esteem	Low	No effect	1	2	Female	Children, Adolescents	Dance	Dance team	Sustained
	Self-perception	Moderate	Positive	1	2	Female	Adolescents	Dance	Aerobic dance	Sustained
Non-communicable disease risk	All-cause mortality	Very Low	Mixed (positive, no effect)	N/A	2	Mixed	Adults, older adults	Music	Instrumental, singing	Sustained
	Asthma	Very Low	No effect	N/A	1	Mixed	Older adults	Music	Instrumental	Sustained
	Cardiovascular disease	Very Low	Mixed (positive, no effect)	N/A	1	Mixed	Adults, Older adults	Dance	Unspecified	Sustained
	Dementia	Low	Positive	N/A	2	Mixed	Older adults	Music	Instrumental	Sustained
	Dementia	Very Low	No effect	N/A	1	Mixed	Older adults	Music	Instrumental	Sustained
	Dementia	Low	Positive	N/A	1	Mixed	Older adults	Dance	Unspecified	Sustained
	Diabetes	Very Low	No effect	N/A	1	Mixed	Older adults	Music	Instrumental	Sustained
	Hypertension	Very Low	No effect	N/A	1	Mixed	Older adults	Music	Instrumental	Sustained
	Metabolic syndrome	Very Low	Positive	N/A	2	Mixed	Adults, Older adults	Dance	Unspecified, International standard	Sustained
	Osteoarthritis	Very Low	No effect	N/A	1	Mixed	Older adults	Music	Instrumental	Sustained
	Rheumatoid arthritis	Very Low	No effect	N/A	1	Mixed	Older adults	Music	Instrumental	Sustained
	Tinnitus	Very Low	Negative	N/A	1	Mixed	Older adults	Music	Instrumental	Sustained
Physical fitness	Abdominal strength/endurance (sit ups)	Moderate	Positive	2	5	Female/ Unspecified	Children, Adolescents, Adults	Dance	Aerobic dance	Sustained
	Cardiovascular capacity (VO2 max)	High	Positive	3	12	Mixed	Adolescents, Adults, Older Adults	Dance	Aerobic dance, Balinese, Dance Team, Greek folk/traditional dance, Waltz, Zumba	Sustained
	Cardiovascular/ cardiorespiratory fitness (Physical Working Capacity	Very Low	Mixed (positive, no effect)	2	5	Mixed	Adults, Older adults	Dance	Agilando, Bhangra, Creative, Social, multiple styles-line/jazz/rock‘n‘roll/ square	Sustained

	130, Senior fitness test, VO2 peak)									
	Endurance (1-mile walk/run)	Very Low	Mixed (positive, no effect)	2	4	Unspecified	Children, Adolescents, Adults	Dance	Aerobic dance, African dance, Hip-hop	Sustained
	Endurance (6MWT)	High	Positive	3	6	Mixed	Adults, Older adults	Dance	Aerobic Dance, Ballroom, Thai, Turkish folk, Zumba	Sustained
	Endurance (muscular/isokinetic)	Low	No effect	1	1	Female	Adults	Dance	Ballet	Sustained
	Heart rate variability	Very Low	Mixed	1	1	Mixed	Adults	Music	Singing	Acute
	Peak ventilation	High	Positive	1	4	Mixed	Adults, Older adults	Dance	Aerobic dance, Greek folk/traditional dance, Zumba	Sustained
	Power (aerobic)	Low	Positive	1	1	Female	Children, Adolescents	Dance	Aerobic dance	Sustained
	Power (muscular/aerobic)	High	No effect	2	2	Mixed	Adults, Older adults	Dance	Ballet, Salsa	Sustained
	Respiratory exchange ratio	Moderate	No effect	1	2	Mixed	Adults	Dance	Aerobic dance, Zumba	Sustained
	Resting HR	Very Low	No effect	1	1	Female	Adults	Dance	Zumba	Sustained
	Strength	Moderate	Positive	3	8	Mixed	Children, Adolescents, Adults, Older adults	Dance	Aerobic Dance, Dance Team, Social	Sustained
	Strength	Low	No effect	1	1	Female	Adults	Dance	Ballet	Sustained
	VO2 at anaerobic threshold	Low	Positive	1	1	Female	Adults	Dance	Aerobic dance	Sustained
Physical function	Agility	Low	Positive	1	1	Female	Children, Adolescents	Dance	Aerobic dance	Sustained
	Balance	High	Positive	6	47	Mixed	Adolescents, Adults, Older adults	Dance	Aerobic Dance, Agilando, Ballet, Ballroom, Caribbean, Contemporary, Greek traditional, Latin, Lebed Method, Line dance, Modern, Multiple-line/jazz/rock‘n‘roll/square, Opera, Salsa, Thai, Turkish folk, Unspecified, Zumba	Sustained
	Balance	Very Low	Mixed (positive, no effect	2	4	Mixed	Children, Adolescents	Dance	Ballet, Modern	Sustained
	Falls	Very Low	Mixed (positive, no effect	1	3	Mixed	Adults, Older adults	Dance	Ballroom, Folk, Low impact aerobic dance	Sustained

	Falls incidence	Very Low	Mixed (positive, no effect)	2	2	Mixed	Older adults	Music	Singing	Sustained
	Fine motor performance	Low	Positive	2	3	Mixed	Older adults	Dance	Agilando, Ballroom, unspecified	Sustained
	Flexibility/range of motion	High	Positive	6	19	Mixed	Children, Adolescents, Adults, Older Adults	Dance	Aerobic dance, Ballroom, Ballet, Folk/traditional dance, Social, Thai, Zumba	Sustained
	Gait (GaitRite)	Low	Positive	1	2	Mixed	Older adults	Dance	Social	Sustained
	Gait speed	Low	No effect	1	5	Mixed	Older adults	Dance	Line dance, Salsa, multiple-line/jazz/ square, Argentine, Folk dance	Sustained
	Lung function	Low	No effect	1	1	Mixed	Adults	Music	Singing	Sustained
	Mobility (Step test)	Very Low	Mixed	1	2	Female	Children, Adolescents, Adults	Dance	Aerobic dance	Sustained
	Mobility (timed up & go; sit to stand)	Moderate	Positive	2	12	Mixed	Older adults	Dance	Aerobic dance, Argentine Tango, Ballroom, Folk, Lebed method, Turkish, Thai	Sustained
	Oestrogen	Low	No effect	1	1	Female	Adults	Dance	Aerobic	Sustained
	Proprioception	High	Positive	1	1	Mixed	Older adults	Dance	Creative Dance	Sustained
	Reaction time	Low	Positive	2	4	Mixed	Older adults	Dance	Agilando, Ballroom, Social, Unspecified	Sustained
	Flexibility (Sit and reach)	Very Low	No effect	1	1	Female	Adolescents	Dance	Dance team	Sustained
	Tactile performance	Low	Positive	2	3	Mixed	Older adults	Dance	Agilando, Ballroom, Unspecified	Sustained
Self-reported health/wellbeing	Alcohol Consumption	Moderate	No effect	1	1	Mixed	Older adults	Dance	Caribbean	Sustained
	Balance confidence	High	Positive	1	1	Mixed	Older adults	Dance	Argentine tango	Sustained
	Body satisfaction (Cathexis)	Very Low	No effect	1	1	Female	Adolescents	Dance	Dance team	Sustained
	Daytime somnolence	Low	No effect	1	1	Mixed	Adults	Music	Singing	Sustained
	Drowsiness	Very Low	Positive	1	1	Mixed	Adults	Music	Drums	Acute
	Fatigue	Very low	No effect	1	1	Mixed	Older adults	Music	Drums	Acute
	Fatigue	High	Positive	2	2	Mixed	Adults	Music	Drums	Acute
	Fatigue	Low	Positive	1	1	Mixed	Older adults	Dance	Greek traditional dance	Acute
	Fear of falling	Very Low	Mixed	1	2	Mixed	Adults, Older adults	Dance	Video game dance, Low impact dance	Sustained
	Functional autonomy	High	Positive	1	1	Unspecified	Older adults	Dance	Ballroom	Sustained
	General health	Very Low	Mixed	1	2	Mixed	Older adults	Dance	Turkish folk, low impact aerobic	Sustained

	Health responsibility	Low	No effect	1	1	Mixed	Older adults	Music	Instrumental	Sustained
	Health system utilization	Very Low	Positive	2	2	Mixed	Older adults	Music	Singing	Sustained
	Lack of coordination	Low	No effect	1	1	Mixed	Adults	Music	Drums	Acute
	Life satisfaction	High	Positive	1	1	Mixed	Older adults	Dance	Creative Dance	Sustained
	Life satisfaction/ Quality of Life/ Wellbeing	Very Low	No effect	1	1	Mixed	Older adults	Music	Singing	Unspecified
	Lifestyle/quality of life	Low	Positive	1	1	Mixed	Older adults	Dance	Agilando	Sustained
	Loneliness	Very Low	Mixed (positive, no effect)	2	2	Mixed	Older Adults	Music	Singing	Sustained
	Medication usage	Very Low	Positive	2	2	Mixed	Older adults	Music	Singing	Sustained
	Morale	Very Low	No effect	1	1	Mixed	Older adults	Music	Singing	Sustained
	Negative affect	Very Low	Mixed (positive, no effect)	2	2	Mixed	Adults	Music	Singing, Drums	Acute
	Negative affect	Low	No effect	1	1	Female	Adults	Music	Drums	Sustained
	Nutrition	Low	No effect	1	1	Mixed	Older adults	Music	Instrumental	Sustained
	Overall health	Very Low	Mixed (positive, no effect)	2	2	Mixed	Older adults	Music	Singing	Sustained
	Perceived self-efficacy	Low	No effect	1	1	Female	Adults	Music	Drums	Sustained
	Personal accomplishment	Low	No effect	1	1	Mixed	Adults	Music	Drums	Sustained
	Positive affect	Low	Positive	5	5	Mixed	Adults	Music	Drums, Singing	Acute/Sustained
	Quality of life	High	Positive	2	2	Mixed	Adults, Older Adults	Music	Singing	Sustained
	Sexual activity	Moderate	Positive	1	1	Mixed	Older adults	Dance	Caribbean	Sustained
	Sleep quality	Moderate	Positive	1	1	Mixed	Older adults	Dance	Caribbean	Sustained
	Smoking	Moderate	No effect	1	1	Mixed	Older adults	Dance	Caribbean	Sustained
	Spiritual growth/wellbeing	Low	Positive	2	2	Mixed	Adults	Music	Singing	Sustained
	Stress	Low	Positive	2	2	Mixed	Adults	Music	Singing, Drums	Sustained/Acute
	Stress	Moderate	Positive	1	1	Mixed	Older adults	Dance	Social	Sustained
	Stress management	Low	No effect	1	1	Mixed	Older adults	Music	Instrumental	Sustained

	Vigor	Very Low	Mixed (positive, no effect)	2	2	Mixed	Adults, older adults	Music	Drums	Acute
	Wellbeing	Low	No effect	1	2	Mixed	Adults, Older adults	Music	Singing, instrumental	Acute
Social functioning	Aggression	Very Low	Positive	1	1	Mixed	Adults	Music	Drums	Acute
	Anger	Very Low	Mixed (positive, no effect)	2	2	Mixed	Adolescents, Adults	Music	Percussion, keyboard, singing	Sustained
	Anger	High	Positive	2	2	Mixed	Adults, Older adults	Music	Drums	Acute
	Job engagement	Low	No effect	1	1	Female	Adults	Music	Drums	Sustained
	Social activity level	Very Low	Positive	2	2	Mixed	Older Adults	Music	Singing	Sustained
	Social anxiety	Low	No effect	1	1	Mixed	Adolescents	Music	Music education (general)	Sustained
	Social functioning	Very Low	Mixed (positive, no effect)	5	6	Mixed	Adolescents, Older Adults	Music	Drums, keyboard, Singing, Instrumental, Music education (general)	Sustained
	Social Support (perceived)	Moderate	Positive	1	1	Mixed	Older adults	Dance	Social	Sustained
	Somatic symptoms	Low	No effect	1	1	Mixed	Older adults	Music	Singing	Sustained

Table S3. Synthesized evidence for each outcome measure from each included review of dance participation. Outcome measures grouped by health domain as per Table S1.

Outcome	GRADE	Effect	Author	Year	Type of evidence synthesis	# studies	Study design	Country of origin	Total N	Participant demographics	Intervention length	Frequency/duration of intervention sessions	Style/instrument
Domain: Auditory													
Domain: Autonomic Tone													
Domain: Blood pressure													
Blood pressure	Moderate	No effect	Fong Yan et al.	2018	Narrative	2	RCT, non-randomized experimental study	Italy, Norway	172	Hospital employees & Type II diabetic/obese adults, mean age 45-59, mixed sex	6 months – 40 weeks	1-2 hours/2x weekly	Ballroom
Blood pressure	Very Low	Positive	Burkhardt & Brennan	2012	Narrative	1	Non-randomized experimental study	USA	16	Young females (age range 14-15; 8 on dance team, 8 physical education control)	5 months	1 hour/5x weekly	Dance team
Blood pressure	Very Low	Mixed (positive & no effect)	Cugusi et al	2019	Narrative	5	RCT, Single group experimental study	Italy, Switzerland, UK, USA	118	Overweight & obese women (mean age 34-54)	8-16 weeks	50-60 min/1-3x weekly	Zumba
Domain: Body Composition													
% body fat	Very Low	Positive	Burkhardt & Brennan	2012	Narrative	1	Non-randomized experimental study	USA	16	Young females (age range 14-15; 8 on dance team, 8 physical education control	5 months	1 hour/5x weekly	Dance team
% body fat	Very Low	Mixed (positive, no effect	Cugusi et al	2019	Narrative	5	RCT, Single group experimental study	Italy, Switzerland, UK, USA	149	Healthy & overweight women (mean age 27-54)	8-16 weeks	50-60 min/1-3x weekly	Zumba
BMI	High	Positive	Fong Yan et al.	2018	Meta-analysis	6	RCT, non-randomized experimental study	Greece, Italy, Japan, Norway, UK, USA	341	Hospital/university employees, schoolgirls, type II diabetic/obese individuals, chronic heart failure (mean age 14-67), mixed sex	6-40 weeks	50 min-2 hours/2-3x weekly	Aerobic dance, Ballroom, Greek folk/traditional dance, Zumba
BMI	Very Low	Mixed (positive & no effect)	Burkhardt & Brennan	2012	Narrative	1	RCT	USA	142	Children (aged 8-13), mixed sex	12 weeks	50 min/3x weekly	Hip-hop, African dance, & aerobics
BMI	Very Low	Mixed (positive, no effect	Cugusi et al	2019	Narrative	6	RCT, Single group experimental study	Italy, Malta, Switzerland, UK, USA	171	Healthy & overweight women (mean age 21-54)	8-16 weeks	50-60 min/1-3x weekly	Zumba

Body composition	Very Low	Mixed (positive, no effect)	Rodrigues-Krause, Krause, Reischak-Oliveira	2019	Narrative	4	RCT, cross-sectional	Canada, Hong Kong, Taiwan	242	Older adults (mean age = 56.4 – 69.1), mixed sex	12-16 weeks; at least 3 years experience	50-60 min/2-3 weekly; at least 150 min weekly	Bhangra, Low impact aerobic dance
Body composition	Low	No effect	Letton, Thom, & Ward	2020	Narrative	1	Non-randomized experimental study	Israel	30	1st-3rd grade girls (age range 6-9)	6 months	90 min/2x weekly	Ballet
Body Mass	High	Positive	Fong Yan et al.	2018	Meta-analysis	8	RCT, non-randomized experimental study	Croatia, Greece, Italy, Japan, Norway, Turkey, USA	541	University/hospital employees; 3rd grade, 4th grade & university students, Type II diabetic/obese individuals, chronic heart failure (mean age 9-67), mixed sex	8 weeks – 8 months	45 min – 2 hours / 2-3x weekly	Aerobic dance, Ballroom, Greek folk/traditional dance, Zumba
Body mass	Very Low	No effect	Burkhardt & Brennan	2012	Narrative	1	Non-randomized experimental study	USA	16	Young females (age range 14-15; 8 on dance team, 8 physical education control	5 months	1 hour/5x weekly	Dance team
Body mass	Very Low	Mixed (positive, no effect	Cugusi et al	2019	Narrative	7	RCT, Single group experimental study	Italy, Malta, Switzerland, UK, USA	198	healthy & overweight women (mean age 27-54)	8-16 weeks	50-60 min/1-3x weekly	Zumba
Skinfold measurements	Moderate	Positive	Fong Yan et al.	2018	Narrative	3	RCT, non-randomized experimental study	Greece, India, Japan	175	1st grade & university students, moderately obese women, mean age 7-52, female/sex unspecified	6-12 weeks	30-60 min/2-3x weekly	Aerobic dance
Total fat mass	Moderate	Positive	Fong Yan et al.	2018	Meta-analysis	4	RCT, non-randomized experimental study, longitudinal cohort study	Croatia, Japan, Norway, USA	440	Female 3rd-4th graders/hospital employees/university students/moderately obese Japanese women (mean age ~9/10 – 52)	8-40 weeks	1 hour/2-3x weekly	Aerobic dance, Zumba
Waist circumference	Low	Positive	Fong Yan et al.	2018	Narrative	2	Non-randomized experimental cohort study	Italy, Japan	160	Obese/Type II diabetic adults, mean age 49-59, mixed sex	12 weeks – 6 months	1-2 hours/2-3x weekly	Aerobic dance, Ballroom, Greek folk/traditional dance, Zumba
Waist circumference	Low	No effect	Burkhardt & Brennan	2012	Narrative	1	RCT	USA	61	African American girls, age 8-10	12 weeks	3x weekly	Hip hop, African dance & step aerobics
Waist circumference	Very Low	Mixed (positive & no effect)	Cugusi et al	2019	Narrative	3	Single group experimental study	Italy, Switzerland, USA	85	Overweight/obese women (mean age 39-54)	12-16 weeks	50-60 min/2-3x weekly	Zumba
Domain: Bone Health													

Bone density (peripheral and spinal)	Very Low	Mixed (positive, no effect)	Keogh et al.	2009	Narrative	1	Single group experimental study	Austria	28	Female members of a senior dancing group, mean age = 66	12 months	Unspecified	Folk, waltz, aerobic dance
Bone density/composition	Very Low	Mixed (positive, no effect)	Rodrigues-Krause, Krause, Reischak-Oliveira	2019	Narrative	3	RCT, cross-sectional	Taiwan, USA	166	Female older adults (mean age = 56 - 59.1)	16 weeks - 6 months; at least 3 years	60 min/2-4x weekly; at least 150 min weekly	Aerobic dance
Bone mineral density	Moderate	No effect	Fong Yan et al.	2018	Narrative	2	RCT, non-randomized experimental study	Norway, UK	101	Female hospital employees & postmenopausal women (mean age 45-58)	10-40 weeks	1 hour/1-2x weekly	Aerobic dance, Zumba
Bone mineral density	Low	Positive	Burkhardt & Brennan	2012	Narrative	2	Cross-sectional, prospective cohort study	Australia	173	Young girls, mean age 8-14	3 years; unspecified	Unspecified	Ballet
Domain: Cognitive													
Attention	Moderate	Positive	Teixeira-Machado, Arida & de Jesus Mari	2019	Narrative	2	RCT	Germany	87	Healthy older adults, mean age 68-70, mixed sex	24 weeks - 18 months	60-90 min/1-2x weekly	Agilando, multiple styles – line/jazz/rock'n'roll/square
BDNF	Moderate	Positive	Teixeira-Machado, Arida & de Jesus Mari	2019	Narrative	1	RCT	Germany	52	Healthy older adults, mean age 68, mixed sex	18 months	90 min/twice weekly (1st 6 months); 90 min/weekly (last 12 months)	Multiple styles – line/jazz/rock'n'roll/square
Brain structure/plasticity	Moderate	Positive	Teixeira-Machado, Arida & de Jesus Mari	2019	Narrative	3	RCT, Cross-sectional	Germany, USA	92	University students & healthy older adults, mean age 22 – 68, mixed sex	18 months; mean 12 years experience	60 - 90 min/1-2x week; mean 14 hours/week	Multiple styles – line/jazz/rock'n'roll/square
Cognitive performance	Low	Positive	Rodrigues-Krause, Krause, Reischak-Oliveira	2019	Narrative	3	Cross-sectional, Single group experimental study	Germany, USA	124	Older adults (mean age = 68 - ~71), mixed sex	15 weeks; Mean 16.5 - 22.1 years dance experience	Unspecified; Mean 1.3 - 4.6 hours weekly dancing	Ballroom, jazz, unspecified
Executive function	High	No effect	Teixeira-Machado, Arida & de Jesus Mari	2019	Narrative	1	RCT	USA	188	Healthy older adults, mean age 65, mixed sex	6 months	3x/week	Social
Executive function	Low	No effect	Rodrigues-Krause, Krause, Reischak-Oliveira	2019	Narrative	1	Cross-sectional	USA	48	Older adults (mean age = 80.4), mixed sex	Mean 36.5 years social dance experience	Unspecified	'social dancing'
Executive function	High	No effect	Meng et al.	2020	Meta-analysis	9	RCT, Non-randomized experimental study	Australia, Brazil, Croatia, France, Greece, Japan, South Korea USA	1286	Older adults (age 60+)mixed sex	10 weeks - 12 months	45-120 min/1-2x weekly	Ballroom, Contemporary, Folk, Latin, Tango, Waltz

Fluid intelligence	Low	No effect	Teixeira-Machado, Arida & de Jesus Mari	2019	Narrative	2	RCT, Cross-sectional	Germany, USA	75	Healthy older adults & female university students, mean age 22-68, mixed sex	24 weeks; mean 12 years experience	1 hour/weekly, mean 14 hours weekly dancing	Agilando, unspecified
Global cognition	High	Positive	Meng et al.	2020	Meta-analysis	7	RCT, non-randomized experimental study	China, Germany, Greece, Japan, South Korea, USA	677	Older adults (age 60+), mixed sex	12-48 weeks	30-90 min/1-5x weekly	Agilando, Ballroom, Latin, Tango, Square dance
Intelligence (nonverbal)	Low	No effect	Teixeira-Machado, Arida & de Jesus Mari	2019	Narrative	1	RCT	Germany	35	Healthy older adults, mean age 70, mixed sex	24 weeks	1 hour/weekly	Agilando
Memory	Very Low	Mixed	Teixeira-Machado, Arida & de Jesus Mari	2019	Narrative	3	RCT, cross-sectional	Germany, USA	280	Healthy older adults & female university students, mean age 22-68, mixed sex	6 - 18 months; mean 12 years dance experience	60-90 min/1-3x weekly; mean 14 hours/week	Social, multiple styles – line/jazz/rock‘n‘roll/square, unspecified
Memory	Very Low	Mixed (positive, no effect)	Meng et al.	2020	Narrative	2	RCT	Australia, Croatia	139	Healthy older adults (age 60+), mixed sex	10 weeks - 8 months	45-60 min/1-2x weekly	Ballroom, Waltz
Memory (episodic)	Low	No effect	Rodrigues-Krause, Krause, Reischak-Oliveira	2019	Narrative	1	Cross-sectional	USA	48	Older adults (mean age = 80.4), mixed sex	Mean 36.5 years social dance experience	Unspecified	Social
Mental status	Low	No effect	Rodrigues-Krause, Krause, Reischak-Oliveira	2019	Narrative	1	Cross-sectional	USA	48	Older adults (mean age = 80.4), mixed sex	Mean 36.5 years social dance experience	Unspecified	Social
Perceptual speed	High	No effect	Teixeira-Machado, Arida & de Jesus Mari	2019	Narrative	2	RCT, Cross-sectional	USA	228	Healthy older adults & female university students, mean age 22-66, mixed sex	6 months; mean 12 years experience	3x/week; mean 14 hours/week	Social, unspecified
Vocabulary	High	No effect	Teixeira-Machado, Arida & de Jesus Mari	2019	Narrative	1	RCT	USA	188	Healthy older adults, mean age 65, mixed sex	6 months	3x/week	Social
Domain: Developmental													
Foot anthropometrics	Low	No effect	Letton, Thom, & Ward	2020	Narrative	1	Cross-sectional	Turkey	67	Female adolescents (mean age = ~11)	Mean 4.4 years ballet experience	Unspecified	Ballet
Growth	Very Low	Mixed	Letton, Thom, & Ward	2020	Narrative	1	Longitudinal cohort study	France	127	Young girls (mean age 12.6)	5 years	Mean 8.8 hours weekly ballet practice	Ballet
Musculoskeletal development	Low	No effect	Burkhardt & Brennan	2012	Narrative	1	Cross-sectional	Israel	1708	Young females, age 8-16	Unspecified	mean 2.4-11.3 hours weekly dance	Ballet
Puberty onset	Very Low	Negative	Letton, Thom, & Ward	2020	Narrative	1	Non-randomized experimental study	Israel	30	1st-3rd grade girls (age range 6-9)	6 months	90 min/2x weekly	Ballet

Domain: Educational												
Domain: Endothelial function												
Domain: Glucose/Insulin												
Glucose	High	No effect	Rodrigues-Krause, Krause, Reischak-Oliveira	2019	Narrative	2	RCT	Canada, Taiwan	107	Adult Women (mean age = 56.4 - 59)	12-16 weeks	60 min/3x weekly	Bhangra, Low impact aerobic dance
Glucose	Moderate	No effect	Fong Yan et al.	2018	Narrative	2	RCT, non-randomized experimental study	Italy, Norway	172	Hospital employees & Type II diabetic/obese adults, mean age 45-59, mixed sex	6 months – 40 weeks	1-2 hours/2x weekly	Ballroom dance, Zumba
Glucose	Very Low	No effect	Cugusi et al	2019	Narrative	2	Single group experimental study	USA	54	Overweight/obese women (mean age = 48-52)	12-16 weeks	60 min/2-3x weekly	Zumba
Insulin	High	No effect	Rodrigues-Krause, Krause, Reischak-Oliveira	2019	Narrative	1	RCT	Canada	75	Post-menopausal obese south east Asian women (mean age = 56 - 57)	12 weeks	60 min/3x weekly	Bhangra
Insulin	Very Low	No effect	Cugusi et al	2019	Narrative	1	Single group experimental study	USA	41	Obese women (mean age = 49.3)	16 weeks	60min/3x weekly	Zumba
Domain: Immune function												
CRP	Low	No effect	Cugusi et al	2019	Narrative	1	RCT	UK	20	Overweight and sedentary women (mean age = 34)	8 weeks	60 min/1-2x weekly	Zumba
IL-6	Low	Unclear	Cugusi et al	2019	Narrative	1	RCT	UK	20	Overweight and sedentary women (mean age = 34)	8 weeks	60 min/1-2x weekly	Zumba
White blood cell count	Low	Negative	Cugusi et al	2019	Narrative	1	RCT	UK	20	Overweight and sedentary women (mean age = 34)	8 weeks	60 min/1-2x weekly	Zumba
Domain: Lipid lipoprotein profile												
Cholesterol	High	Positive	Rodrigues-Krause, Krause, Reischak-Oliveira	2019	Narrative	1	RCT	Taiwan	32	Sedentary females (mean age 59)	16 weeks	60 min/3x weekly	Low impact aerobic dance
HbA1c	Very Low	No effect	Cugusi et al	2019	Narrative	1	Single group experimental study	USA	41	Obese women (mean age = 49.3)	16 weeks	60min/3x weekly	Zumba
HDL cholesterol	High	Positive	Fong Yan et al.	2018	Meta-analysis	4	RCT, non-randomized experimental study	Italy, Norway, Turkey	290	Female hospital employees & university students,	8-40 weeks	21 min-2 hours/2-3x weekly	Aerobic dance, Ballroom, Waltz, Zumba

										Type II diabetic/obese adults, chronic heart failure (mean age 21 - 59.5), mixed sex			
HDL cholesterol	Very Low	Mixed (no effect, unclear)	Cugusi et al	2019	Narrative	2	Single group experimental study	USA	54	Overweight/obese women (mean age = 49-52)	12-16 weeks	60 min/2-3x weekly	Zumba
Total cholesterol	High	Positive	Fong Yan et al.	2018	Meta-analysis	4	RCT, non-randomized experimental study	Italy, Norway, Turkey	290	Female hospital employees & university students, Type II diabetic/obese adults, chronic heart failure (mean age 21 - 59.5), mixed sex	8-40 weeks	21 min-2 hours/2-3x weekly	Aerobic dance, Ballroom, Waltz, Zumba
Total cholesterol	Low	No effect	Cugusi et al	2019	Narrative	2	RCT, Single group experimental study	UK, USA	61	Overweight/obese women (mean age 34-49)	8-16 weeks	60 min/1-3x weekly	Zumba
Triglycerides	High	Positive	Rodrigues-Krause, Krause, Reischak-Oliveira	2019	Narrative	1	RCT	Taiwan	32	Sedentary females (mean age 59)	16 weeks	60 min/3x weekly	Low impact aerobic dance
Triglycerides	High	Positive	Fong Yan et al.	2018	Meta-analysis	4	RCT, non-randomized experimental study	Italy, Norway, Turkey	290	Female hospital employees & university students, Type II diabetic/obese adults, chronic heart failure (mean age 21 - 59.5), mixed sex	8-40 weeks	21 min-2 hours/2-3x weekly	Aerobic dance, Ballroom, Waltz, Zumba
Triglycerides	Very Low	Mixed (positive & no effect)	Cugusi et al	2019	Narrative	2	Single group experimental study	USA	54	Overweight/obese women (mean age = 49-52)	12-16 weeks	60 min/2-3x weekly	Zumba
Domain: Mental health													
Anxiety	Low	Positive	Rodrigues-Krause, Krause, Reischak-Oliveira	2019	Narrative	1	RCT	Greece	111	Healthy older adults (mean age = 69.8), mixed sex	60 min	Once	Greek traditional dance
Anxiety	Low	Positive	Burkhardt & Brennan	2012	Narrative	1	Non-randomized experimental study	UK	84	University students, mean age 20, mixed sex	12 weeks	Unspecified	Modern
Depression	High	No effect	Rodrigues-Krause, Krause,	2019	Narrative	3	RCT, Single group experimental	Turkey, USA	98	Older adults (mean age = 68 – 80.4), mixed sex	8-15 weeks; mean 36.5 years	1 hour/3x weekly; unspecified	Turkish folk dance, Jazz, Social

			Reischak-Oliveira				study, cross-sectional				dancing experience		
Mood	Moderate	Positive	Burkhardt & Brennan	2012	Narrative	1	RCT	South Korea	277	High school & undergraduate students, mean age 21, mixed sex	50 min	Once	Hip hop
Psychological distress	Low	Positive	Rodrigues-Krause, Krause, Reischak-Oliveira	2019	Narrative	1	Non-randomized experimental study	Greece	111	Healthy older adults (mean age = 69.8), mixed sex	60 min	Once	Greek traditional dance
Psychological wellbeing	Low	Positive	Rodrigues-Krause, Krause, Reischak-Oliveira	2019	Narrative	1	Non-randomized experimental study	Greece	111	Healthy older adults (mean age = 69.8), mixed sex	60 min	Once	Greek traditional dance
Self-esteem	Low	No effect	Burkhardt & Brennan	2012	Narrative	2	Non-randomized experimental study, RCT	USA	77	Young females, mean age 8-15	12 weeks - 5 months	1 hour/3-5x weekly	Dance team
Self-perception	Moderate	Positive	Burkhardt & Brennan	2012	Narrative	2	Non-randomized experimental study, RCT	UK	163	Adolescent girls, mean age 14-16	5-6 weeks	50 - 60 min/1-2x weekly	Aerobic dance
Domain: Physical fitness													
6MWT	High	Positive	Cugusi et al	2019	Narrative	1	RCT	Switzerland	30	Healthy women (mean age = 21)	8 weeks	60 min/2x weekly	Zumba
Abdominal strength/endurance (sit ups)	Moderate	Positive	Fong Yan et al.	2018	Meta-analysis	4	RCT, longitudinal cohort study, non-randomized experimental study	Greece, India, USA	260	1st grade, 5th grade & university students, mean age 7-20, female/sex unspecified	6-12 weeks	30-60 min/3x weekly	Aerobic dance
Cardiovascular fitness (VO2max)	High	Positive	Fong Yan et al.	2018	Meta-analysis	6	RCT, non-randomized experimental study	Greece, Italy, Japan, Norway, USA	313	Hospital/university employees, moderately obese women, female university students, chronic heart failure (mean age 19-67), mixed sex	7-40 weeks	21-60 min/2-4x weekly	Aerobic dance, Greek folk/traditional dance, Waltz, Zumba
Cardiovascular/ cardiorespiratory fitness (Physical Working Capacity 130, VO2 peak)	High	No effect	Teixeira-Machado, Arida & de Jesus Mari	2019	Narrative	3	RCT	Germany, USA	275	Healthy older adults, mean age 65-70, mixed sex	24 weeks - 18 months	60-90 min/1-3x weekly	Social, Agilando, multiple styles-line/jazz/rock‘n‘roll/square
Core strength	Low	Positive	Burkhardt & Brennan	2012	Narrative	1	Non-randomized experimental study	Croatia	220	3rd & 4th grade girls	60 high school periods	Unspecified	Aerobic dance

Endurance	Low	No effect	Letton, Thom, & Ward	2020	Narrative	1	Cross-sectional	USA	27	Female university students (age range 18-35)	2+ years ballet experience	Unspecified	Ballet
Endurance (1-mile walk/run)	Moderate	Positive	Fong Yan et al.	2018	Narrative	3	RCT, non-randomized experimental study	Greece, India	172	Students (1st grade, 5th grade & university), mean age 6-~20, sex unspecified		6-12 weeks	Aerobic dance
Endurance (6-min walk test)	Moderate	Positive	Liu, Shen & Tsai	2020	Meta-analysis	4	RCT	Brazil, Hong Kong, Thailand, Turkey	206	Healthy older adults, mean age 65-74, mixed sex	6 weeks - 3 months	40-60 min/2-3x weekly	Ballroom, Turkish folk, low impact aerobic, Thai dance
Endurance (6-min walk test)	Low	Positive	Fong Yan et al.	2018	Narrative	1	Non-randomized experimental study	Italy	100	Obese/Type II diabetic adults, mean age 59, mixed sex	6 months	2 hours/2x weekly	Ballroom dance
Fitness (general/aerobic)	High	Positive	Rodrigues-Krause, Krause, Reischak-Oliveira	2019	Narrative	2	RCT	Canada, Portugal	132	Older women (mean age 57.1 - 72.8)	12-24 weeks	50-60 min/3x weekly	Bhangra, Creative Dance
Muscular power	High	No effect	Rodrigues-Krause, Krause, Reischak-Oliveira	2019	Narrative	1	RCT	Germany	28	Older adults (69-72), mixed sex	8 weeks	1 hour/2x weekly	Salsa
Peak ventilation	High	Positive	Fong Yan et al.	2018	Meta-analysis	4	RCT, non-randomized experimental study	Greece, Norway, USA	165	University students & employees, hospital employees, chronic heart failure patients (mean age 19-67), mixed sex	7 weeks-8 months	30-60 min/2-4x weekly	Aerobic dance, Greek folk/traditional dance, Zumba
Power	Low	No effect	Letton, Thom, & Ward	2020	Narrative	1	Cross-sectional	USA	27	Female university students (age range 18-35)	2+ years ballet experience	Unspecified	Ballet
Power	Low	Positive	Burkhardt & Brennan	2012	Narrative	1	Non-randomized experimental study	Croatia	220	3rd & 4th grade girls	60 high school periods	Unspecified	Aerobic dance
Respiratory exchange ratio	Moderate	No effect	Fong Yan et al.	2018	Narrative	2	RCT	Norway, USA	97	University & hospital employees, mean age 37-45, mixed sex	8-40 weeks	50-60 min/2-3x weekly	Aerobic dance, Zumba
Resting HR	Very Low	No effect	Cugusi et al	2019	Narrative	1	Single group experimental study	Switzerland	17	Overweight women (median age = 54)	12 weeks	60 min/3x weekly	Zumba
Strength	Moderate	Positive	Rodrigues-Krause, Krause, Reischak-Oliveira	2019	Narrative	5	RCT, Cross-sectional	Hong Kong, Taiwan, USA	311	Older adults (mean age = 56 – 80.4), mixed sex	3-6 months; 3+ years experience	50-60 min/2-3x weekly; 2-5 dances/2-4x weekly; 150+ min weekly	Aerobic dance, social
Strength	Low	No effect	Letton, Thom, & Ward	2020	Narrative	1	Cross-sectional	USA	27	Female university students (age range 18-35)	2+ years ballet experience	Unspecified	Ballet

Strength	Very Low	Positive	Burkhardt & Brennan	2012	Narrative	1	Non-randomized experimental study	USA	16	Young females (age range 14-15; 8 on dance team, 8 physical education control	5 months	1 hour/5x weekly	Dance team
Strength (upper body - push-ups)	Low	Positive	Fong Yan et al.	2018	Narrative	2	Non-randomized experimental cohort study	Greece	97	1st – 5th grade students, mean age 7-11, sex unspecified	8-12 weeks	45 min/3x weekly	Aerobic dance
Timed mile run	Moderate	No effect	Burkhardt & Brennan	2012	Narrative	1	RCT	USA	81	Adolescents, mean age 10-13, sex unspecified	12 weeks	50 min/3x weekly	Hip-hop, African dance, & aerobics
VO2 at anaerobic threshold	Low	Positive	Fong Yan et al.	2018	Narrative	1	Non-randomized experimental study	Japan	60	Moderately obese Japanese women, mean age = 52	12 weeks	1 hour/2-3x weekly	Aerobic dance
VO2 max	Low	Positive	Burkhardt & Brennan	2012	Narrative	2	Non-randomized experimental study	Indonesia, USA	76	Adolescents, age 14-20, mixed sex	8 weeks - 5 months	50-60 min/3-5x weekly	Balinese, Dance team
VO2 max	High	Positive	Cugusi et al	2019	Narrative	4	RCT, Single group experimental study	Switzerland, UK, USA	122	Healthy & overweight women (mean age 27-54)	8-16 weeks	60 min/1-3x weekly	Zumba
Domain: Physical function													
Agility	Low	Positive	Burkhardt & Brennan	2012	Narrative	1	Non-randomized experimental study	Croatia	220	3rd & 4th grade girls	60 high school periods	Unspecified	Aerobic dance
Balance	Low	Positive	Clarke et al.	2018	Narrative	27	Cross-sectional	Australia, France, Germany, Italy, Spain, Taiwan, Thailand, USA	1018	Individuals (dancers & non-dancers) aged 16-35; mixed sex	5+ years dance training	Variable, unspecified	Ballet, modern, Thai, opera
Balance	Moderate	Positive	Teixeira-Machado, Arida & de Jesus Mari	2019	Narrative	3	RCT, Cross-sectional	Germany, USA	127	Older adults & University students, mean age 22 – 70, mixed sex	24 weeks - 18 months; mean 12 years experience	60 - 90 min/1-2x week; mean 14 hours/week	Unspecified, Agilando, multiple styles
Balance	High	Positive	Liu, Shen & Tsai	2020	Narrative	4	RCT	Germany, Greece, Turkey, USA	114	Healthy older adults, mean age 69-74, mixed sex	8 - 10 weeks	1 hour/2-3x weekly	Line dance, Turkish folk, Salsa, Greek traditional
Balance	Moderate	Positive	Rodrigues-Krause, Krause, Reischak-Oliveira	2019	Narrative	10	RCT, cross-sectional, single group experimental study	Brazil, China, Germany, Hong Kong, Italy, USA	811	Older adults (mean age = 61.4-80.4), mixed sex	6 weeks - 8 months; mean 6.1-36.5 years experience	45-90 min/1-3x weekly; mean 1.3-4.8 hours weekly dancing	Ballroom, Caribbean, Jazz, Low impact aerobic dance, Lebed Method, Social
Balance	Low	Positive	Hwang & Braun	2015	Narrative	1	Non-randomized experimental study	France	41	Healthy older adults - mean age = 73-74, mixed sex	4.4 months (average)	1 hour/weekly	Contemporary

Balance	Very Low	Mixed (positive, no effect)	Costa, Ferreira & Felicio	2013	Narrative	3	Cross-sectional	Brazil, France, Taiwan	51	Adolescents & young adults (dancers & non-dancers), mean age 14-19, mixed sex	Mean 31-84 months experience	3+ hours/weekly; unspecified	Ballet, Modern
Falls	Very Low	Mixed	Veronese et al.	2017	Narrative	3	RCT	Australia, Brazil, Taiwan	621	Older adults, mean age 59-68, mixed sex	12 weeks - 12 months	50-60 min/2-3x weekly	Ballroom, Folk, Low impact aerobic dance
Fine motor performance	Low	Positive	Teixeira-Machado, Arida & de Jesus Mari	2019	Narrative	1	RCT	Germany	35	Healthy older adults, mean age 70, mixed sex	24 weeks	1 hour/weekly	Agilando
Fine motor performance	Low	Positive	Rodrigues-Krause, Krause, Reischak-Oliveira	2019	Narrative	2	Cross-sectional	Germany	111	Older adults (mean age = ~71), mixed sex	Mean 16.5 - 22.1	Mean 1.3 - 4.6 hours weekly dancing	Ballroom, unspecified
Flexibility	Moderate	Positive	Liu, Shen & Tsai	2020	Meta-analysis	4	RCT	Czech Republic, Hong Kong, Thailand	239	Healthy older adults, mean age 65-82, mixed sex	6 weeks - 3 months	40-75 min/1-3x weekly	Ballroom, low impact aerobic, Thai dance
Flexibility	Moderate	Positive	Rodrigues-Krause, Krause, Reischak-Oliveira	2019	Narrative	3	RCT, cross-sectional	China, Taiwan	474	Older adults (mean age = 59-62), mixed sex	16 weeks; 3+ years dance experience	60 min/3x weekly; 150+ min weekly	Low impact aerobic dance, social
Flexibility	Low	Positive	Letton, Thom, & Ward	2020	Narrative	2	Cross-sectional, non-randomized experimental study	Finland, Israel	65	Female children & adolescents (age range 6-12)	6 months; 2+ years ballet experience	90 min/2x weekly; Mean 7.25 - 11.1 hours weekly training	Ballet
Flexibility (sit & reach – each leg separately)	Low	Positive	Fong Yan et al.	2018	Narrative	2	Non-randomized experimental cohort study	Greece	97	1st – 5th grade students, mean age 7-11, sex unspecified	8-12 weeks	45 min/3x weekly	Aerobic dance
Flexibility (sit & reach)	High	Positive	Fong Yan et al.	2018	Meta-analysis	5	RCT, longitudinal cohort study, non-randomized experimental study	Croatia, India, Norway, Thailand, USA	493	3rd & 4th grade & university students, hospital employees, older women (mean age 9/10 - 67), female/unspecified sex	6-40 weeks	30-60 min/2-3x weekly	Aerobic dance, Folk/traditional dance, Zumba
Gait	Low	Positive	Rodrigues-Krause, Krause, Reischak-Oliveira	2019	Narrative	2	Cross-sectional	China, USA	452	Older adults (mean age 61.4 – 80.4), mixed sex	Mean 6.1 - 36.5 years dance experience	Mean 4.8 weekly dancing sessions; unspecified	Social
Gait Speed	Low	No effect	Liu, Shen & Tsai	2020	Meta-analysis	5	RCT	Australia, Canada, Germany, USA	641	Healthy older adults, mean age 67-78, mixed sex	8 weeks - 12 months	60-120 min/2x weekly	Line dance, Salsa, multiple-line/jazz/square, Argentine, Folk dance
Mobility	Low	Positive	Rodrigues-Krause, Krause, Reischak-Oliveira	2019	Narrative	3	Cross-sectional, single group experimental study	Germany, USA	122	Older adults (mean age = ~71), mixed sex	6 weeks; mean 16.5-22.1 years dance experience	45 min/3x weekly; mean 1.3-4.6 hours weekly dance	Ballroom, Lebed Method, Unspecified

Mobility (Step test)	Very Low	Mixed	Fong Yan et al.	2018	Narrative	2	Non-randomized experimental cohort study, longitudinal cohort study	Croatia, USA	308	3rd & 4th grade & university female students (mean age 9/10-20)	8 weeks – 60 high school periods	1 hour/3x weekly	Aerobic dance
Mobility (timed up & go; sit-to-stand)	Moderate	Positive	Liu, Shen & Tsai	2020	Meta-analysis	9	RCT	Australia, Brazil, Canada, Czech Republic, Hong Kong, Spain, Thailand, Turkey	917	Healthy older adults, mean age 65-81, mixed sex	6 weeks - 12 months	40-75 min/1-3x weekly	Ballroom, Turkish folk, Low impact aerobic, Thai dance, Argentine Tango, Folk, Multiple-Flamenco, Sevillanas, ballet
Oestrogen	Low	No effect	Rodrigues-Krause, Krause, Reischak-Oliveira	2019	Narrative	1	RCT	USA	96	Older Caucasian women (mean age = ~56)	6 months	2-5 dances, 2-4 days/week	Aerobic
Postural stability	High	Positive	Fong Yan et al.	2018	Narrative	2	RCT	Greece, Norway	96	Older adults & female hospital employees, mean age 45-71, mixed sex	12-40 weeks	1 hour/2x weekly	Latin, Zumba
Posture	Low	No effect	Letton, Thom, & Ward	2020	Narrative	1	Non-randomized experimental study	Israel	30	1st-3rd grade girls (age range 6-9)	6 months	90 min/2x weekly	Ballet
Proprioception	High	Positive	Hwang & Braun	2015	Narrative	1	RCT	Portugal	37	Inactive older adult - mean age = 64-65, mixed sex	12 weeks	90 min/3x weekly	Creative Dance
Range of motion	Low	Positive	Fong Yan et al.	2018	Narrative	3	Non-randomized experimental cohort study	Croatia, Greece	317	1st – 5th grade students, mean age 7-11, sex female/unspecified	8-12 weeks	45 min/3x weekly	Aerobic dance
Reaction time	Low	Positive	Teixeira-Machado, Arida & de Jesus Mari	2019	Narrative	1	RCT	Germany	35	Healthy older adults, mean age 70, mixed sex	24 weeks	1 hour/weekly	Agilando
Reaction time	Low	Positive	Rodrigues-Krause, Krause, Reischak-Oliveira	2019	Narrative	3	Cross-sectional	China, Germany	515	Older adults (mean age = 61 – 77), mixed sex	Mean 6.1 - 22.1 years dancing experience	Mean 1.3 - 48 hours weekly dancing	Ballroom, social, unspecified
Sit and reach	Very Low	No effect	Burkhardt & Brennan	2012	Narrative	1	Non-randomized experimental study	USA	16	Young females (age range 14-15; 8 on dance team, 8 physical education control	5 months	1 hour/5x weekly	Dance team
Tactile performance	Low	Positive	Teixeira-Machado, Arida & de Jesus Mari	2019	Narrative	1	RCT	Germany	35	Healthy older adults, mean age 70, mixed sex	24 weeks	1 hour/weekly	Agilando
Tactile performance	Low	Positive	Rodrigues-Krause, Krause,	2019	Narrative	2	Cross-sectional	Germany	111	Older adults (mean age = ~71), mixed sex	Mean 16.5 - 22.1	Mean 1.3 - 4.6 hours weekly dancing	Ballroom, unspecified

			Reischak-Oliveira										
Domain: Self-reported health/ wellbeing													
Alcohol Consumption	Moderate	No effect	Rodrigues-Krause, Krause, Reischak-Oliveira	2019	Narrative	1	RCT	Italy	40	Older adults (mean age = 61.3), mixed sex	3 months	35-60 min/2x weekly (35 min 1st 2 weeks, then 60 min)	Caribbean
Balance confidence	High	Positive	Rodrigues-Krause, Krause, Reischak-Oliveira	2019	Narrative	1	RCT	Canada	25	Older adults (mean age = 74-78), mixed sex	10 weeks	2 hours/2x weekly	Argentine tango
Body satisfaction (Cathexis)	Very Low	No effect	Burkhardt & Brennan	2012	Narrative	1	Non-randomized experimental study	USA	16	Young females (age range 14-15; 8 on dance team, 8 physical education control	5 months	1 hour/5x weekly	Dance team
Fatigue	Low	Positive	Rodrigues-Krause, Krause, Reischak-Oliveira	2019	Narrative	1	Non-randomized experimental study	Greece	111	Healthy older adults (mean age = 69.8), mixed sex	60 min	Once	Greek traditional dance
Fear of falling	Very Low	Mixed	Veronese et al.	2017	Narrative	2	RCT	Switzerland, Taiwan	63	Healthy older adults, mean age 59-86, mixed sex	12-16 weeks	10-60 min/2-3x weekly	Video game dance, Low impact dance
Functional autonomy	High	Positive	Rodrigues-Krause, Krause, Reischak-Oliveira	2019	Narrative	1	RCT	Brazil	75	Sedentary older subjects (mean age = 67-68), sex unspecified	8 months	50 min/3x weekly	Ballroom
General health	Very Low	Mixed	Liu, Shen & Tsai	2020	Narrative	2	RCT	Hong Kong, Turkey	134	Healthy older adults, mean age 68-74, mixed sex	8 weeks - 3 months	50-60 min/2-3x weekly	Turkish folk, low impact aerobic
Life satisfaction	High	Positive	Rodrigues-Krause, Krause, Reischak-Oliveira	2019	Narrative	1	RCT	Portugal	57	Older women (mean age 71-73)	24 weeks	50 min/3x weekly	Creative Dance
Lifestyle/quality of life	Low	Positive	Teixeira-Machado, Arida & de Jesus Mari	2019	Narrative	1	RCT	Germany	35	Healthy older adults, mean age 70, mixed sex	24 weeks	1 hour/weekly	Agilando
Sexual activity	Moderate	Positive	Rodrigues-Krause, Krause, Reischak-Oliveira	2019	Narrative	1	RCT	Italy	40	Older adults (mean age = 63.1), mixed sex	3 months	35-60 min/2x weekly (35 min 1st 2 weeks, then 60 min)	Caribbean
Sleep quality	Moderate	Positive	Rodrigues-Krause, Krause, Reischak-Oliveira	2019	Narrative	1	RCT	Italy	40	Older adults (mean age = 63.1), mixed sex	3 months	35-60 min/2x weekly (35 min 1st 2 weeks, then 60 min)	Caribbean

Smoking	Moderate	No effect	Rodrigues-Krause, Krause, Reischak-Oliveira	2019	Narrative	1	RCT	Italy	40	Older adults (mean age = 63.1), mixed sex	3 months	35-60 min/2x weekly (35 min 1st 2 weeks, then 60 min)	Caribbean
Stress	Moderate	Positive	Teixeira-Machado, Arida & de Jesus Mari	2019	Narrative	1	RCT	USA	247	Healthy older adults, mean age 65, mixed sex	6 months	3x/week	Social
Domain: Social functioning													
Social Support (perceived)	Moderate	Positive	Teixeira-Machado, Arida & de Jesus Mari	2019	Narrative	1	RCT	USA	247	Healthy older adults, mean age 65, mixed sex	6 months	3x/week	Social

Table S4. Synthesized evidence for each outcome measure from each included review of music participation. Outcome measures grouped by health domain as per Table S1.

Outcome	*GRADE*	*Effect*	*Author*	*Year*	*Type of evidence synthesis*	*# studies*	*Study design*	*Country of origin*	*Total N*	*Participant demographics*	*Intervention length*	*Frequency/duration of intervention sessions*	*Style/instrument*
Domain: Auditory													
Auditory processing	Moderate	Positive	Coffey, Mogilever & Zatorre	2017	Narrative	13	Cross-sectional, nonrandomized longitudinal cohort study	Canada, France, UK, USA	624	Adults & adolescents (musicians & nonmusicians); mean age = 15=56, mixed sex	3 years +	Variable, unspecified	Instrumental
Auditory working memory	Very Low	Mixed	Coffey, Mogilever & Zatorre	2017	Narrative	2	Cross-sectional	USA	64	Adults (musicians & nonmusicians), age 18-40, mixed sex	10+ years musical experience	At least 3 hours/week	Piano & violin, unspecified
Hearing threshold	Very Low	Negative	Clift et al	2010	Narrative	1	Cross-sectional	Austria	89	Adults (musicians & nonmusicians), age range 18-61, mixed sex	Mean 24 (male) – 27 (female) years performing experience	Mean weekly singing of 15 (male) – 29 (female) hours	Singing
Melodic contour identification	Low	Positive	Coffey, Mogilever & Zatorre	2017	Narrative	1	Cross-sectional	Netherlands	50	Adults (musicians & nonmusicians), mean age = 23, mixed sex	Average 14.6 years musical training	Unspecified ('regular musical training within the last 3 years')	Instrumental
Pitch discrimination	Moderate	Positive	Coffey, Mogilever & Zatorre	2017	Narrative	7	Cross-sectional	Canada, UK, USA	351	Adults (musicians & nonmusicians), mean age 23-31, mixed sex	6+ years music training	3+ hours/week; unspecified	Instrumental, unspecified
Speech in noise	Moderate	Positive	Coffey, Mogilever & Zatorre	2017	Narrative	21	RCT, cross-sectional	Canada, France, Netherlands, UK, USA	863	Adults & children, mean age 8 - 56, mixed sex	1+ years	2+ hours/week; unspecified	Instrumental, vocal, unspecified
Vocal emotion identification	Low	Positive	Coffey, Mogilever & Zatorre	2017	Narrative	1	Cross-sectional	Netherlands	50	Adults (musicians & nonmusicians), mean age = 23, mixed sex	Average 14.6 years musical training	Unspecified ('regular musical training within the last 3 years')	Instrumental
Domain: Autonomic Tone													
Adrenaline	Very low	No effect	Fancourt, Ockelford & Belai	2014	Narrative	1	Single group study (uncontrolled)	Japan	54	Adult & older adult nonmusicians (mean age 27-70, mixed sex)	1 hour	Once	Drums
Noradrenaline	Very low	Positive	Fancourt, Ockelford & Belai	2014	Narrative	1	Single group study (uncontrolled)	Japan	27	Older adult nonmusicians (mean age 70.3), mixed sex	1 hour	Once	Drums
Noradrenaline	Very low	No effect	Fancourt, Ockelford & Belai	2014	Narrative	1	Single group study (uncontrolled)	Japan	27	Younger adult nonmusicians (mean age 27.9), mixed sex	1 hour	Once	Drums
Domain: Blood pressure													
Blood pressure	Very Low	Mixed (positive-	Yap, Kwan & Ang	2017	Narrative	1	Single group experimental	South Africa	34	Young adult novice drummers & middle	40 minutes	Once	Drums

		middle aged adults, no effect young adults)					study (pre-post analysis)			aged experienced drummers, mean age 23 - 53, mixed sex			
Blood pressure	Very Low	No effect	Daykin et al	2018	Narrative	1	Nonrandomized experimental cohort study	UK	23	University singers, mean age 21, mixed sex	30 minutes	Once	Singing
Domain: Body Composition													
Domain: Bone Health													
Domain: Cognitive													
Confusion	Very low	No effect	Fancourt, Ockelford & Belai	2014	Narrative	1	Single group study (uncontrolled)	Japan	27	Older adult nonmusicians (mean age 70.3), mixed sex	1 hour	Once	Drums
Confusion	Very low	Positive	Fancourt, Ockelford & Belai	2014	Narrative	1	Single group study (uncontrolled)	Japan	27	Younger adult nonmusicians (mean age 27.9), mixed sex	1 hour	Once	Drums
Confusion	Very Low	Positive	Yap, Kwan & Ang	2017	Narrative	1	Nonrandomized experimental study	USA	50	University students, mean age 21, mixed sex	45 min - 1 hour	Once	Drums
Executive function	Low	No effect	Coffey, Mogilever & Zatorre	2017	Narrative	1	Cross-sectional	USA	34	Adults (musicians & nonmusicians), mean age = 24, sex unspecified	Unclear	Unspecified	Instrumental
Intelligence (nonverbal/logical – Raven's Standard Progressive Matrices)	Low	No effect	Hetland	2000	Meta-analysis	3	Longitudinal cohort study, Retrospective cross sectional analysis	Hungary, Switzerland, USA	694	Children (age 6-15), mixed sex	7 months – 3 years	4-6 classes, weekly	Music education (general, Kodaly)
IQ	Moderate (1 high quality study showing positive effects; rated down for publication bias)	Positive	Jaschke et al.	2013	Narrative	4	RCT, cross-sectional & longitudinal cohort study	Hong Kong, Canada, Australia	399	Children (age 6-13), mixed sex	6.5 months - 5 years	1-3 hours, weekly; multiple studies unspecified	Music education

IQ	Low	Positive	Coffey, Mogilever & Zatorre	2017	Narrative	1	Cross-sectional	USA	33	University students (musicians and nonmusicians), mean age = 21, mixed sex	At least 10 years of musical training, beginning before age 10	Currently playing at least 5 hours/week	Instrumental
IQ (nonverbal)	Low	No effect	Coffey, Mogilever & Zatorre	2017	Narrative	6	Cross-sectional	UK, USA	250	Adults (musicians & nonmusicians), mean age 22-56, mixed sex	10 + years musical experience	3+ hours/weekly; unspecified	Instrumental, vocal, unspecified
Long-term memory	Moderate	Positive	Talamini et al	2017	Meta-analysis	9	Cross-sectional	Canada, China, Germany, Hong Kong, UK, USA	411	Adult musicians and nonmusicians (mean age 19-28, mixed sex)	4+ years music training	Largely unspecified	Instrumental
Mental flexibility	Low	Positive	Coffey, Mogilever & Zatorre	2017	Narrative	1	Cross-sectional	UK, USA	50	Adults (musicians & nonmusicians) mean age = 22, sex unspecified	average 22.7 years of musical training	practice at least 3x/weekly over the 3 years before testing	Instrumental
Selective attention	Low	No effect	Coffey, Mogilever & Zatorre	2017	Narrative	2	Cross-sectional	UK, USA	84	Adults (musicians & nonmusicians), mean age 24-27, sex unspecified	Average 14-22 years musical training	3+ hours/week; unclear	Instrumental
Short-term memory	Moderate	Positive	Talamini et al	2017	Meta-analysis	17	Cross-sectional	Australia, Brazil, Canada, Denmark, Finland, France, Germany, Israel, Italy, Singapore, Taiwan, UK, Ukraine, USA	756	Adult musicians and nonmusicians (mean age 20-32, mixed sex)	5+ years music training	Variable, unspecified	Instrumental, singing
Spatial abilities (recognition, memory, mental rotation, spatial visualization)	Low (‚equivocal results of meta analysis‘)	Positive	Hetland	2000	Meta-analysis	8	RCT, Non randomized experimental cohort study, Longitudinal cohort study, Retrospective cross sectional analysis	Switzerland, USA	655	Children (age 3-15), mixed sex	4 weeks – 8 months	10 – 75 min	Music education (general, Kodaly, Kindermusik, snare drum, piano, vocal)

Spatial Reasoning	High	Positive	Hetland	2000	Meta-analysis	15	RCT, Non randomized experimental cohort study, Longitudinal cohort study, Retrospective cross sectional analysis	Canada, USA	701	Children (age 4-9), mixed sex	7 weeks – 8 months	10 – 60 minutes, 1-5x/week	Music education (general, Kodaly, piano, voice)
Visual attention	Low	No effect	Coffey, Mogilever & Zatorre	2017	Narrative	1	Cross-sectional	USA	33	University students (musicians and nonmusicians), mean age = 21, mixed sex	At least 10 years musical experience beginning before age 11	At least 3 hours/week	Unspecified
Working memory	Moderate	Positive	Talamini et al	2017	Meta-analysis	16	Cross-sectional	Australia, Canada, Denmark, Finland, France, Germany, Italy, Singapore, Taiwan, UK, USA	536	Adult musicians and nonmusicians (mean age 20-50, mixed sex)	9+ years music training	Variable, unspecified	Instrumental, singing
Domain: Developmental												
Domain: Educational												
Language expression (not otherwise specified)	Moderate	Negative	Jaschke et al.	2013	Narrative	1	RCT	Canada	117	Children (aged 8-12), mixed sex	3 years	30-45 min, weekly	Music education (general)
Language skills	Very low	No effect	Coffey, Mogilever & Zatorre	2017	Narrative	1	Nonrandomized longitudinal cohort study	USA	68	High school students, mean age 15, sex unspecified	3 years	2.33-3 hours/week	Instrumental
Mathematics	Very low (mixed no effect, positive results from moderate/high quality studies)	Mixed	Jaschke et al.	2013	Narrative	3	RCT	Australia, Canada	330	Children (aged 6-12), mixed sex	5 months - 3 years	30 min - 1 hour, weekly; 1 study unspecified	Music education (general)
Mathematics (causation - experimental)	Low	Positive	Vaughn	2000	Meta-analysis	5	Non-randomized longitudinal	Canada, USA	339	Students (kindergarten - 6th grade), mixed sex	14 months - 3 years	30 minutes+, 1-3/x weekly	Music education (general, Kodaly, Suzuki, piano)

							cohort study, longitudinal cohort study						
Mathematics (correlation with music instruction)	Moderate	Positive	Vaughn	2000	Meta-analysis	18	Cross-sectional, retrospective cohort study, unclear	USA	5738142	Students (3rd grade - college), mixed sex	8 months - 5 years+	70 min+, weekly; unspecified	Music education (general, Kodaly, Suzuki, piano)
Phonological awareness	Very Low	Positive	Gordon, Fehd & McCandliss	2015	Meta-analysis	18	RCT, Non-randomized experimental study, cross-sectional	Brazil, Canada, Germany, Spain, UK, USA	1230	Children (mean age 4.5-9.1; mixed sex)	4-26 weeks	10-120 min/1-5x weekly	Music education (general, rhythm intervention, music & movement)
Reading	Very Low (mixed negative, no effect & positive results from moderate & high quality studies)	Mixed	Jaschke et al.	2013	Narrative	7	RCT, cross-sectional & longitudinal cohort study	Germany, USA, Canada, Australia	463	Children (</= age 13), mixed sex	20 weeks - 1.27 years; unspecified duration in multiple studies	Minimum 30 min/week; multiple studies unspecified	Music education (general)
Reading abilities (causation)	Low	No effect	Butzlaff	2000	Meta-analysis	6	Cross-sectional, longitudinal cohort study, non-randomized experimental study, unclear (experimental)	UK, USA	253	Students (1st - 4th grade), mixed sex	3-6 months	30 - 60 min, 2-3x/week	Orchestra, music education (instrumental, vocal, Suzuki)
Reading abilities (correlation)	Moderate	Positive	Butzlaff	2000	Meta-analysis	24	Non-randomized experimental cohort study, cross-sectional, retrospective cohort study, longitudinal cohort study, unclear (correlational)	USA	5734913	Students (1st - 12th grade), mixed sex	4 months - 3 years	30 - 90 min, 1-2x week; 7-8 hours/weekly	Orchestra, music education (instrumental, vocal, Suzuki)
Reading Fluency	Low	No effect	Gordon, Fehd & McCandliss	2015	Meta-analysis	5	RCT, Non-randomized experimental study	Brazil, Portugal, UK, USA	434	Children aged 5-9, mixed sex	6-24 weeks	30-75 min/1-3x weekly	Music education (general, rhythm intervention)

Writing	Very Low (mixed no effect, positive results from Low/Very Low quality studies)	Mixed	Jaschke et al.	2013	Narrative	2	pseudo-RCT; non-randomized	USA, Australia	134	Children (age 4-13), mixed sex	30 weeks - 6.5 months	30 minutes, twice weekly; unspecified	Music education (general)
Domain: Endothelial Function / Stress Response													
Cortisol	Low	No effect	Fancourt, Ockelford & Belai	2014		3	RCT, non-randomized longitudinal cohort study, non-randomized intervention study	German, Sweden, USA	151	Adolescents & adults with mixed levels of musical experience (mean age 13-30), mixed sex	1 hour (2 studies); 8 months (1 study)	Once (2 studies); 1 hour/week (1 study)	Singing, music education, drums
Cortisol	Very Low	Mixed	Clift et al	2010	Narrative	1	Single group experimental study (pre-post analysis)	USA	42	Professional singers, mean age 46, mixed sex	1.5-2.5 hours	Once	Singing
Cortisol	Very Low	No effect	Gick	2012	Narrative	2	Cross-sectional, single group cohort study	Sweden, USA	26	Professional & amateur singers, mean age 21-40, mixed sex	45 minutes – one rehearsal	Once; 4-11 data collections	Singing
Cortisol/Cortisol:DHEA ratio	Low	No effect	Daykin et al	2018	Narrative	1	Nonrandomized experimental trial	Germany	21	Adults participating in choir for research purposes, median age ~50, mixed sex	30 minutes	Once	Singing
DHEA	Low	No effect	Fancourt, Ockelford & Belai	2014	Narrative	1	RCT	USA	60	Adults (unspecified musical experience; mean age = 30, mixed sex)	1 hour	Once	Drums
DHEA	Low	No effect	Daykin et al	2018	Narrative	1	Nonrandomized experimental trial	Germany	21	Adults participating in choir for research purposes, median age ~50, mixed sex	30 minutes	Once	Singing
DHEA to cortisol ratio	Low	No effect	Fancourt, Ockelford & Belai	2014	Narrative	1	RCT	USA	60	Adults (unspecified musical experience; mean age = 30, mixed sex)	1 hour	Once	Drums
Domain: Glucose/Insulin													

Domain: Immune function													
IFN-alpha	Very low (mixed effects from very low & low quality evidence)	Mixed	Fancourt, Ockelford & Belai	2014	Narrative	2	RCT, Single group study	Japan, USA	114	Adults (varying levels of musical experience), mean age 28-70, mixed sex	1 hour	Once	Drums
IL-2	Low	No effect	Fancourt, Ockelford & Belai	2014	Narrative	2	RCT, Single group study	Japan, USA	114	Adults (varying levels of musical experience), mean age 28-70, mixed sex	1 hour	Once	Drums
Immune function (B cells; CD4/CD8 ratio; CD8+ T cells; IL-10 production; IL-4 production; Naive T cells; Naive:memory T cell; Neutrophils; NK cell count)	Very low	No effect	Fancourt, Ockelford & Belai	2014	Narrative	1	Single group study (uncontrolled)	Japan	54	Adult & older adult nonmusicians (mean age 27-70, mixed sex)	1 hour	Once	Drums
Immune function (CD4+ T cells; IL-6 production; Lymphocytes; Memory T cells; T cells)	Very low	Positive	Fancourt, Ockelford & Belai	2014	Narrative	1	Single group study (uncontrolled)	Japan	27	Older adult nonmusicians (mean age 70.3), mixed sex	1 hour	Once	Drums
Immune function (CD4+ T cells; IL-6 production; Lymphocytes; Memory T cells; T cells)	Very low	No effect	Fancourt, Ockelford & Belai	2014	Narrative	1	Single group study (uncontrolled)	Japan	27	Younger adult nonmusicians (mean age 27.9), mixed sex	1 hour	Once	Drums
Immune function (IFN-gamma gene expression (mRNA), IL-10 gene expression (mRNA))	High	Positive	Yap, Kwan & Ang	2017	Narrative	1	RCT	Japan	40	Healthy males, mean age 38	60 minutes	Once	Drums
Immune function (IL-2 gene expression (mRNA), IL-6 gene expression (mRNA), CD-56+ cells, leukocyte counts)	High	No effect	Yap, Kwan & Ang	2017	Narrative	1	RCT	Japan	40	Healthy males, mean age 38	60 minutes	Once	Drums
Immunoglobulin A	Very Low	Positive	Clift et al	2010	Narrative	1	Single group experimental study (pre-post analysis)	USA	42	Professional singers, mean age 46, mixed sex	1.5 (performance) – 2.5 (rehearsal) hours	Once (3 pre-post tests; 2 rehearsal, 1 performance)	Singing

Immunoglobulin A	Very Low	Positive	Gick	2012	Narrative	1	Single group cohort study	USA	10	Conservatory singers, mean age 21, sex unspecified	One rehearsal/ performance	Data collections from 4-11 rehearsals/ performances over a 10 week period	Singing
Immunoglobulin A	High	Positive	Fancourt, Ockelford & Belai	2014	Narrative	2	RCT, non-randomized intervention study	USA, Germany	64	University students and amateur singers (age range 18-74), mixed sex	30 min – 1 hour	Once	Drums, Singing
LAK cell activity	Low	Positive	Fancourt, Ockelford & Belai	2014	Narrative	1	RCT	USA	60	Adults (unspecified musical experience; mean age = 30, mixed sex)	1 hour	Once	Drums
NK cell activity	Very low (mixed effects from very low & low quality evidence)	Mixed	Fancourt, Ockelford & Belai	2014	Narrative	2	RCT, Single group study	Japan, USA	114	Adults (varying levels of musical experience), mean age 28-70, mixed sex	1 hour	Once	Drums
NK cell activity	Very Low	Mixed	Yap, Kwan & Ang	2017	Narrative	1	RCT	Japan	40	Healthy males, mean age 38	60 minutes	Once	Drums
TNF-alpha	Very Low	Mixed	Gick	2012	Narrative	1	Cross-sectional	Sweden	16	Amateur/professiona l singers, mean age 40, mixed sex	45 min	Once	Singing
White blood cell count	Low	No effect	Fancourt, Ockelford & Belai	2014	Narrative	2	RCT, Single group study	Japan, USA	114	Adults (varying levels of musical experience), mean age 28-70, mixed sex	1 hour	Once	Drums
Domain: Lipid lipoprotein profile													
Domain: Mental health													
Anxiety	Very low	Mixed (positive, no effect)	Fancourt, Ockelford & Belai	2014	Narrative	2	RCT, Single group study	Japan, USA	114	Adults (varying levels of musical experience), mean age 28-70, mixed sex	1 hour	Once	Drums
Anxiety	Very Low	Positive	Yap, Kwan & Ang	2017	Narrative	1	Nonrandomized experimental study	USA	50	University students, mean age 21, mixed sex	45 min - 1 hour	Once	Drums
Anxiety	High	Positive	Daykin et al	2018	Narrative	2	RCT	Germany, UK	412	Adults, mean age 25-69, mixed sex	41 minutes, 14 weeks	Once, 90 minutes (weekly	Drums, singing
Anxiety	Low	No effect	Raglio et al.	2019	Narrative	1	RCT (only comparative, not inactive control group)	South Korea	20	Graduate female music therapy students, mean age 28	4 weeks	60 minutes, weekly	Drums

Anxiety	Low	Positive	Clift et al	2010	Narrative	1	Non randomized experimental cohort study	UK	61	Residents of retirement homes (mean age 84), mixed sex	4 weeks	1 hour, weekly	Singing
Anxiety (state)	Very Low	No effect	Phillips & Becker	2019	Narrative	1	Single group study (uncontrolled)	USA	62	Healthcare workers, mean age 48, mixed sex	5 weeks	90 minutes, weekly	Singing
Anxiety (trait)	Very Low	Positive	Phillips & Becker	2019	Narrative	1	Single group study (uncontrolled)	USA	62	Healthcare workers, mean age 48, mixed sex	5 weeks	90 minutes, weekly	Singing
Burnout	Low	Positive	Phillips & Becker	2019	Narrative	1	RCT	USA	125	Healthcare workers, mean age 44, mixed sex	6 weeks	1 hour, weekly	Drums
Compassion fatigue	Very Low	No effect	Phillips & Becker	2019	Narrative	1	Nonrandomized experimental study (2 experimental groups)	USA	17	Hospice workers, age range 28-60, mixed sex	6 weeks	60 minutes, weekly	Instrumental
Depersonalization	Low	No effect	Yap, Kwan & Ang	2017	Narrative	1	Nonrandomized crossover experimental study	USA	75	Nursing students, mean age 28, mixed sex	6 weeks	1 hour, weekly	Drums
Depression	Moderate	Positive	Daykin et al	2013	Narrative	1	RCT	USA	52	at risk' youth (aged 14-18), mixed sex		1 hour, weekly	Music (Percussion & keyboard education)
Depression	Very low (mixed effects from very low & low quality evidence)	Mixed	Fancourt, Ockelford & Belai	2014	Narrative	2	RCT, Single group study	Japan, USA	114	Adults (varying levels of musical experience), mean age 28-70, mixed sex	1 hour	Once	Drums
Depression	Very Low	Positive	Yap, Kwan & Ang	2017	Narrative	1	Nonrandomized experimental study	USA	50	University students, mean age 21, mixed sex	45 min - 1 hour	Once	Drums
Depression	High (when measured immediately post-intervention; depression measured ~6-months post intervention in	Positive	Daykin et al	2018	Narrative	3	RCT, Nonrandomized experimental study	Germany, UK, USA	578	Adults, mean age 25-69, mixed sex	41 minutes, 14-30 weeks	Once, Weekly (duration unspecified - 90 minutes)	Singing

	observational study)												
Depression	Very Low	Mixed	Clift et al	2010	Narrative	2	Nonrandomized experimental study	UK, USA	189	Older adults (over 64), mean age 79-84, mixed sex	4-30 weeks	Once weekly	Singing
Emotional exhaustion	Low	Positive	Yap, Kwan & Ang	2017	Narrative	1	Nonrandomized crossover experimental study	USA	75	Nursing students, mean age 28, mixed sex	6 weeks	1 hour, weekly	Drums
Emotional regulation	Low	Positive	Moore	2013	Narrative	14	Cross-sectional	Canada, Germany, Japan, Sweden, USA	255	Adults with varying musical experience (non-musicians - professional), mean age 22-38, mixed sex	<2 hours	Once	Singing, piano, guitar
Mood	Very low	Positive	Fancourt, Ockelford & Belai	2014	Narrative	1	Single group study (uncontrolled)	Japan	54	Adult & older adult nonmusicians (mean age 27-70, mixed sex)	1 hour	Once	Drums
Mood	High	Positive	Yap, Kwan & Ang	2017	Narrative	2	RCT, Nonrandomized crossover experimental study	USA, Japan	115	Nursing students & healthy males, mean age 21 - 38, mixed sex	60 minutes - 6 weeks	Once - 1 hour (weekly)	Drums
Mood	High	Positive	Daykin et al	2018	Narrative	4	RCT, nonrandomized experimental study	Germany, UK, USA	364	Adults, university students, mean age 21-50, mixed sex	30-41 minutes; 30 weeks	Once; once/week (duration unspecified)	Singing, drums
Mood	Low	Positive	Phillips & Becker	2019	Narrative	1	RCT	USA	125	Healthcare workers, mean age 44, mixed sex	6 weeks	1 hour, weekly	Drums
Mood	Low	No effect	Raglio et al.	2019	Narrative	1	RCT (only comparative, not inactive control group)	South Korea	20	Graduate female music therapy students, mean age 28	4 weeks	60 minutes, weekly	Drums
Mood	Very Low	Mixed	Clift et al	2010	Narrative	1	RCT	Australia	107	Adults (varying levels of musical experience), age range 18-73, mixed sex	30 minutes	Once	Singing
Self-actualization	Very Low	Unclear	Clift et al	2010	Narrative	1	Cross-sectional	USA	98	Retirees, mean age 65, mixed sex	Unspecified (chorus members generally with more robust musical histories)	Unspecified	Singing

Self-esteem	High	Positive	Daykin et al	2013	Narrative	1	RCT	USA	45	Juvenile delinquents / 'at risk' youth (aged 9-19), sex unspecified	3 months	1 hour, weekly	Guitar
Self-esteem	Low	No effect	Fancourt, Ockelford & Belai	2014	Narrative	1	Non-randomized longitudinal cohort study	Sweden	60	5th & 6th graders, sex unspecified (likely mixed)	8 months	1 hour, weekly	Music education (general)
Domain: Physical fitness													
Heart rate variability	Very Low	Mixed	Gick	2012	Narrative	1	Cross-sectional	Sweden	16	Amateur/professional singers, mean age 40, mixed sex	45 min	Once	Singing
Domain: Physical function													
Falls incidence	Very Low	Positive	Daykin et al	2018	Narrative	1	Nonrandomized experimental study	USA	166	Older adults, mean age 74, sex unspecified	30 weeks	Once per week (duration unspecified)	Singing
Falls incidence	Very Low	No effect	Clift et al	2010	Narrative	1	Nonrandomized experimental study	USA	128	Older adults (over 64), mean age 79, mixed sex	2 periods of 30 weeks over 2 years	Once per week (duration unspecified)	Singing
Lung function	Low	No effect	Clift et al	2010	Narrative	1	Cross-sectional	USA	113	Professional musicians, mean age 41-47, mixed sex	Mean years performing range 18.6-27.5 across groups	Unspecified	Singing
Domain: Self-reported health/wellbeing													
Daytime somnolence	Low	No effect	Gick	2012	Narrative	1	Cross-sectional	UK	107	Adults (musicians & nonmusicians), mean age 45, mixed sex	Mean 30.8 years singing	Unspecified	Singing
Drowsiness	Very Low	Positive	Yap, Kwan & Ang	2017	Narrative	1	Nonrandomized experimental study	USA	50	University students, mean age 21, mixed sex	45 min - 1 hour	Once	Drums
Fatigue	Very low	No effect	Fancourt, Ockelford & Belai	2014	Narrative	1	Single group study (uncontrolled)	Japan	27	Older adult nonmusicians (mean age 70.3), mixed sex	1 hour	Once	Drums
Fatigue	Very low	Positive	Fancourt, Ockelford & Belai	2014	Narrative	1	Single group study (uncontrolled)	Japan	27	Younger adult nonmusicians (mean age 27.9), mixed sex	1 hour	Once	Drums
Fatigue	High	Positive	Daykin et al	2018	Narrative	1	RCT	Germany	154	Adults, mean age 25, mixed sex	41 minutes	Once	Drums
Health responsibility	Low	No effect	Daykin et al	2018	Narrative	1	Non randomized longitudinal intervention study	UK	98	Older adults, mean age 68, mixed sex	10 weeks	60-90 mins, weekly	Instrumental

Health system utilization	Very Low	Positive	Daykin et al	2018	Narrative	1	Nonrandomized experimental study	USA	166	Older adults, mean age 74, sex unspecified	30 weeks	Once per week (duration unspecified)	Singing
Health system utilization	Very Low	Positive	Clift et al	2010	Narrative	1	Nonrandomized experimental study	USA	128	Older adults (over 64), mean age 79, mixed sex	2 periods of 30 weeks over 2 years	Once per week (duration unspecified)	Singing
Lack of coordination	Low	No effect	Yap, Kwan & Ang	2017	Narrative	1	Nonrandomized experimental study	USA	50	University students, mean age 21, mixed sex	45 min - 1 hour	Once	Drums
Life satisfaction	Very Low	No effect	Clift et al	2010	Narrative	1	Cross-sectional	USA	98	Retirees, mean age 65, mixed sex	Unspecified (chorus members generally with more robust musical histories)	Unspecified	Singing
Loneliness	Low	Positive	Daykin et al	2018	Narrative	1	Nonrandomized experimental study	USA	166	Older adults, mean age 74, sex unspecified	30 weeks	Once per week (duration unspecified)	Singing
Loneliness	Very Low	No effect	Clift et al	2010	Narrative	1	Nonrandomized experimental study	USA	128	Older adults (over 64), mean age 79, mixed sex	2 periods of 30 weeks over 2 years	Once per week (duration unspecified)	Singing
Medication usage	Very Low	Positive	Daykin et al	2018	Narrative	1	Nonrandomized experimental study	USA	166	Older adults, mean age 74, sex unspecified	30 weeks	Once per week (duration unspecified)	Singing
Medication usage (over the counter + prescription)	Very Low	Positive	Clift et al	2010	Narrative	1	Nonrandomized experimental study	USA	128	Older adults (over 64), mean age 79, mixed sex	2 periods of 30 weeks over 2 years	Once per week (duration unspecified)	Singing
Morale	Very Low	No effect	Clift et al	2010	Narrative	1	Nonrandomized experimental study	USA	128	Older adults (over 64), mean age 79, mixed sex	2 periods of 30 weeks over 2 years	Once per week (duration unspecified)	Singing
Negative affect	Low	Positive	Fancourt, Ockelford & Belai	2014	Narrative	1	Non-randomized intervention study	Germany	31	Adult amateur singers (mean age 56.9, range 29-74), mixed sex	1 hour	Once	Singing
Negative affect	Low	No effect	Yap, Kwan & Ang	2017	Narrative	1	Nonrandomized experimental study	UK	32	Adults, mean age 25 - 44, mixed sex	30 minutes	Once	Drums
Negative affect	Low	No effect	Raglio et al.	2019	Narrative	1	RCT (only comparative, not inactive control group)	South Korea	20	Graduate female music therapy students, mean age 28	4 weeks	60 minutes, weekly	Drums
Nutrition	Low	No effect	Daykin et al	2018	Narrative	1	Non randomized longitudinal intervention study	UK	98	Older adults, mean age 68, mixed sex	10 weeks	60-90 mins, weekly	Instrumental

Overall health	Very Low	Positive	Daykin et al	2018	Narrative	1	Nonrandomized experimental study	USA	166	Older adults, mean age 74, sex unspecified	30 weeks	Once/week (duration unspecified	Singing
Overall health	Very Low	No effect	Clift et al	2010	Narrative	1	Nonrandomized experimental study	USA	128	Older adults (over 64), mean age 79, mixed sex	2 periods of 30 weeks over 2 years	Once per week (duration unspecified)	Singing
Perceived self-efficacy	Low	No effect	Raglio et al.	2019	Narrative	1	RCT (only comparative, not inactive control group)	South Korea	20	Graduate female music therapy students, mean age 28	4 weeks	60 minutes, weekly	Drums
Personal accomplishment	Low	No effect	Yap, Kwan & Ang	2017	Narrative	1	Nonrandomized crossover experimental study	USA	75	Nursing students, mean age 28, mixed sex	6 weeks	1 hour, weekly	Drums
Positive affect	Low	Positive	Fancourt, Ockelford & Belai	2014	Narrative	1	Non-randomized intervention study	Germany	31	Adult amateur singers (mean age 56.9, range 29-74), mixed sex	1 hour	Once	Singing
Positive affect	Low	Positive	Yap, Kwan & Ang	2017	Narrative	1	Nonrandomized experimental study	UK	32	Adults, mean age 25 - 44, mixed sex	30 minutes	Once	Drums
Positive affect	Low	Positive	Raglio et al.	2019	Narrative	1	RCT (only comparative, not inactive control group)	South Korea	20	Graduate female music therapy students, mean age 28	4 weeks	60 minutes, weekly	Drums
Positive affect	Very Low	Positive	Clift et al	2010	Narrative	1	Single group experimental study (pre-post analysis)	Finland	212	Amateur/advanced choir members, mean age 53, mixed sex	1 rehearsal (duration unspecified)	Once	Singing
Positive affect	Very Low	Positive	Gick	2012	Narrative	1	Cross-sectional	Sweden	16	Amateur/professional singers, mean age 40, mixed sex	45 min	Once	Singing
Quality of life	Very Low	Positive	Phillips & Becker	2019	Narrative	1	Single group study (uncontrolled)	USA	62	Healthcare workers, mean age 48, mixed sex	5 weeks	90 minutes, weekly	Singing
Quality of life (mental)	High	Positive	Daykin et al	2018	Narrative	1	RCT	UK	258	Older adults, mean age 69, mixed sex	14 weeks	90 minutes, weekly	Singing
Quality of life (physical)	High	No effect	Daykin et al	2018	Narrative	1	RCT	UK	258	Older adults, mean age 69, mixed sex	14 weeks	90 minutes, weekly	Singing
Spiritual growth	Low	Positive	Daykin et al	2018	Narrative	1	Non randomized longitudinal intervention study	UK	98	Older adults, mean age 68, mixed sex	10 weeks	60-90 minutes, weekly	Instrumental

Spiritual Wellbeing	Very Low	Positive	Phillips & Becker	2019	Narrative	1	Single group study (uncontrolled)	USA	62	Healthcare workers, mean age 48, mixed sex	5 weeks	90 minutes, weekly	Singing
Stress	Very Low	Positive	Phillips & Becker	2019	Narrative	1	Single group study (uncontrolled)	USA	62	Healthcare workers, mean age 48, mixed sex	5 weeks	90 minutes, weekly	Singing
Stress management	Low	No effect	Daykin et al	2018	Narrative	1	Non randomized longitudinal intervention study	UK	98	Older adults, mean age 68, mixed sex	10 weeks	60-90 minutes, weekly	Instrumental
Stress/anxiety	Low	Positive	Yap, Kwan & Ang	2017	Narrative	1	Single group experimental study (pre-post analysis)	South Africa	34	Young adult novice drummers & middle aged experienced drummers, mean age 23 - 53, mixed sex	40 minutes	Once	Drums
Vigor	Very low	No effect	Fancourt, Ockelford & Belai	2014	Narrative	1	Single group study (uncontrolled)	Japan	54	Adult & older adult nonmusicians (mean age 27-70, mixed sex)	1 hour	Once	Drums
Vigor/arousal	High	Positive	Daykin et al	2018	Narrative	1	RCT	Germany	154	Adults, mean age 25, mixed sex	41 minutes	Once	Drums
Wellbeing	Low	No effect	Daykin et al	2018	Narrative	2	Nonrandomized experimental study	UK, USA	166	Male inmates; older adults, mean age 34-68, mixed sex	One performance; 10 weeks	Once; 60-90 mins/week	Singing, instrumental
Domain: Social functioning													
Aggression	Very Low	Positive	Yap, Kwan & Ang	2017	Narrative	1	Nonrandomized experimental study	USA	50	University students, mean age 21, mixed sex	45 min - 1 hour	Once	Drums
Anger	Moderate	No effect	Daykin et al	2013	Narrative	1	RCT	USA	52	at risk' youth (aged 14-18), mixed sex	6 weeks	1 hour, weekly	Music (Percussion & keyboard education)
Anger	Very low	Positive	Fancourt, Ockelford & Belai	2014	Narrative	1	Single group study (uncontrolled)	Japan	54	Adult & older adult nonmusicians (mean age 27-70, mixed sex)	1 hour	Once	Drums
Anger	High	Positive	Daykin et al	2018	Narrative	1	RCT	Germany	154	Adults, mean age 25, mixed sex	41 minutes	Once	Drums
Anger (state)	Very Low	No effect	Phillips & Becker	2019	Narrative	1	Single group study (uncontrolled)	USA	62	Healthcare workers, mean age 48, mixed sex	5 weeks	90 minutes, weekly	Singing
Anger (trait)	Very Low	Positive	Phillips & Becker	2019	Narrative	1	Single group study (uncontrolled)	USA	62	Healthcare workers, mean age 48, mixed sex	5 weeks	90 minutes, weekly	Singing

Job engagement	Low	No effect	Raglio et al.	2019	Narrative	1	RCT (only comparative, not inactive control group)	South Korea	20	Graduate female music therapy students, mean age 28	4 weeks	60 minutes, weekly	Drums
Psychosocial function	Moderate	No effect	Daykin et al	2013	Narrative	1	RCT	USA	52	at risk' youth (aged 14-18), mixed sex		1 hour, weekly	Music (Percussion & keyboard education)
Social activity level	Very Low	Positive	Clift et al	2010	Narrative	1	Nonrandomized experimental study	USA	128	Older adults (over 64), mean age 79, mixed sex	2 periods of 30 weeks over 2 years	Once per week (duration unspecified)	Singing
Social anxiety	Low	No effect	Fancourt, Ockelford & Belai	2014	Narrative	1	Non-randomized longitudinal cohort study	Sweden	60	5th & 6th graders, sex unspecified (likely mixed)	8 months	1 hour, weekly	Music education (general)
Social engagement (activity level)	Very Low	Positive	Daykin et al	2018	Narrative	1	Nonrandomized experimental study	USA	166	Older adults, mean age 74, sex unspecified	30 weeks	Once per week (duration unspecified)	Singing
Social functioning	Moderate	No effect	Daykin et al	2013	Narrative	1	RCT	USA	52	at risk' youth (aged 14-18), mixed sex	6 weeks	1 hour, weekly	Percussion & keyboard
Social functioning	Low	No effect	Fancourt, Ockelford & Belai	2014	Narrative	1	Non-randomized longitudinal cohort study	Sweden	60	5th & 6th graders, sex unspecified (likely mixed)	8 months	1 hour, weekly	Music education (general)
Social functioning	Low	Positive	Yap, Kwan & Ang	2017	Narrative	1	Nonrandomized intervention study	USA	101	5th graders, mean age 11, mixed sex	12 weeks	40-45 minutes, weekly	Drums
Social functioning	Low	No effect	Daykin et al	2018	Narrative	1	Non randomized longitudinal intervention study	UK	98	Older adults, mean age 68, mixed sex	10 weeks	60-90 minutes, weekly	Instrumental
Social functioning	Low	No effect	Clift et al	2010	Narrative	2	Nonrandomized experimental cohort study; cross-sectional	UK, USA	159	Retirees (amateur musicians & nonmusicians), mean age 65-84, mixed sex	4 weeks; unspecified	1 hour, weekly; unspecified	Singing
Somatic symptoms	Low	No effect	Clift et al	2010	Narrative	1	Non randomized experimental cohort study	UK	61	Residents of retirement homes (mean age 84), mixed sex	4 weeks	1 hour	Singing

Table S5. Summary of included reviews

Author	Year	Music / Dance?	Type of synthesis	Review objective(s)	# databases searched	Additional manual search?	Search date range	Inclusion criteria	Exclusion criteria	Domain(s)
Burkhardt & Brennan	2012	Dance	Narrative	Explore the effects of recreational dance interventions on the health and well-being of children and young people	6	No	1947-2009	1) studies reporting the effects of a recreational dance intervention on the physical, psychological or social health of young people aged 5-21; 2) controlled trials, cohort studies, case control studies, cross-sectional studies with control group for comparison	1) Dance Movement Therapy or video dance games; 2) dance programs leading to professional dance; 3) participants were professional dancers; 4) non-controlled studies, observational studies, studies with no quantitative or statistical analysis of results	Blood pressure; Body composition; Bone health; Developmental; Mental health; Physical fitness; Physical function; Self-reported health/wellbeing
Butzlaff	2000	Music	Meta-analysis	Can music be used to enhance reading abilities?	7	Yes	Inception-1998	1) standardized measure of reading ability was used as the dependent variable; 2) a test of reading followed music 'instruction'; 3) statistical information was sufficient to allow for the calculation of an effect size	None specified	Educational
Clarke et al.	2018	Dance	Narrative	Identify all relevant literature on balance and theatrical styles of dance, involving adult participants who were either in full-time dance training or professional dancers	7	Yes	1980-2016	1) experimental study; 2) referred to theatrical dance forms; 3) involved professional dancers and/or dance students in vocational and university training; 4) examined balance	1) recreational or competition dance; 2) participants aged younger than 17 and/or older than 45 years old	Physical function
Clift et al	2010	Music	Narrative	Systematically map the research literature on group singing, wellbeing and health	4	Yes	1985-2009	1) research which involves clearly identified singing groups (amateur & professional) & outcomes related to their wellbeing effects	1) studies concerned with individual singing/individual music therapy interventions involving singing, studies of song writing as a form of singing, care-giver singing; 2) singing combined with other forms of music-making or creative activity	Auditory; Endothelial function/stress response; Immune function/inflammation; Mental health; Physical function; Self-reported health/wellbeing; Social functioning
Coffey, Mogilever & Zatorre	2017	Music	Narrative	1) Consider how speech in noise tasks may vary; 2) consider coverage of the research area by different neuroimaging measures that offer complementary views of the neurophysiological basis of speech in noise differences between musicians and non-musicians; 3) suggest a means of comparing and planning future studies; 4) highlight specific research areas for future study	1	No	Inception-2016	Studies of musician effects on speech-in-noise perception within neurologically normal populations	None specified	Auditory; Cognitive; Educational

Costa, Ferreira & Felicio	2013	Dance	Narrative	Review studies about static and dynamic postural balance of ballet dancers, characterizing visual dependency in the postural control of these athletes to maintain balance	5	No	1997-2013	1) studies involving assessment of static or dynamic balance in ballet dancers	None specified	Physical function
Cugusi et al	2019	Dance	Narrative	Systematically summarize, analyze and interpret the cardiovascular benefits of Zumba Fitness in women, in order to outline its potential roles in primary prevention of cardiovascular disease in both healthy subjects and women presenting with specific cardiovascular risk factors	5	Yes	Inception-2017	1) women of all ages with or without specific cardiovascular risk factors; 2) analyses of cardiovascular outcomes arising from a Zumba fitness program lasting 2+ weeks	None specified	Blood pressure; Body composition; Glucose/insulin; Immune function/inflammation; lipid lipoprotein profile; Physical fitness
Daykin et al	2013	Music	Narrative	What is the evidence surrounding the impact of music-making on the well-being, health and behavior of young offenders?	11	Yes	1996-2013	Interventions with young people aged 11-25 in a range of youth justice settings (custodial settings + 'at risk' populations)	1) Studies conducted in the general population; 2) studies of disadvantaged and clinical populations who share some characteristics of young offenders but who were not targeted on the basis of having committed/being at risk of committing an offence	Mental health; Social functioning
Daykin et al	2018	Music	Narrative	What are the wellbeing outcomes of music and singing for adults and what are the processes by which wellbeing outcomes are achieved?	9	Yes	1996-2016	Empirical studies in any language from countries economically similar to the UK; studies assessed individual/group music interventions and any recognized measure of subjective wellbeing in adults; quantitative studies only included with a concurrent comparator	1) Research subjects are paid professionals; 2) music used for clinical purposes (pain management, symptom relief); 3) music therapy interventions seeking to deliver wellbeing outcomes	Blood pressure; Endothelial function/stress response; Mental health; Physical function; Self-reported health/wellbeing; Social functioning
Eells	2014	Music	Narrative	Explore the literature on the effects of music and singing as a nursing intervention on the symptoms of anxiety in older adults	3	Yes	Inception-2011	1) participants over the age of 65; 2) related to anxiety and the use of music/singing as a nursing intervention; 3) Related to service user experiences of anxiety and the use of music/singing interventions	None specified	***All studies meeting umbrella review inclusion criteria included in other reviews***
Fancourt, Ockelford & Belai	2014	Music	Narrative	Consolidate key findings to date, compare theories concerning the mechanisms behind music‘s effect, and highlight gaps in current knowledge, helping to guide the focus of future studies	6	Yes	1953-2013	1) new study; 2) ,controlled in order that the significance of alterations in biomarkers could be accurately assessed; 3) studies pairing music with other stimuli (i.e. exercise) were only included if they also contained a test incorporating just music on its own; 4) studies had to be	Animal studies	Autonomic tone; Endothelial function/stress response; Immune function/inflammation; Mental health; Self-reported health/wellbeing; Social functioning

								testing for potential positive effects of music		
Fernandez-Arguelles et al.	2015	Dance	Narrative	Determine the therapeutic effects of dancing as a physical exercise modality on balance, flexibility, gait and muscle strength in older adults	5	Unspecified	2000 - 2013	1) Studies analyzing the effects of dance (ballroom dance and/or dance based exercise) in older adults over 60 years of age with no disabling disease on balance, gait, dynamic mobility, strength, and flexibility outcomes; 2) Studies in English or Spanish	1) Qualitative studies that did not quantify results; 2) Studies in which participants presented with a cognitive deficit and/or mental or neurological (physical or psychological) condition	***All studies meeting umbrella review inclusion criteria included in other reviews***
Fong Yan et al.	2018	Dance	Meta-analysis	Systematically review the literature to investigate the effectiveness of structured dance interventions, in comparison to structured exercise programs, on physical health outcome measures	7	Yes	Inception-2017	1) original study comparing forms of dance intervention to a structured exercise program; 2) dance classes must have been used in the intervention; 3) minimum 4 week intervention duration; 4) measurable physical health outcomes; 5) comparison group that performed a structured exercise program	1) literature reviews or case studies; 2) investigated psychological and/or mental health outcome measures only; 3) examined the effect of dance therapy, movement therapy, or creative movement; 4) dance intervention combined with other types of structured exercise; 5) control group did not exercise and/or were given unstructured physical activity	Body composition; Lipid lipoprotein profile; Physical fitness; Physical function
			Narrative							Blood pressure; Body composition; Bone health; Glucose/insulin; Lipid lipoprotein profile; Physical fitness; Physical function
Gick	2012	Music	Narrative	Consolidate research on singing, health and well-being into a common conceptual framework in order to facilitate its evaluation, consideration of possible mechanisms by which singing may have its effects, and suggestions for further research	4	Yes	Inception-2012	1) articles investigating active singing and some health or well-being outcome; 2) English language articles	1) studies which investigated a multi-component treatment including singing with other interventions (musical or other); No data reported; attempts to obtain the article failed	Endothelial function/stress response; Immune function/inflammation; physical fitness; self-reported health/wellbeing
Gordon, Fehd & McCandliss	2015	Music	Meta-analysis	Synthesize previous research on music training and reading-related outcomes	14	Unspecified	Inception-2014	1) intervention with a control group; 2) peer-reviewed publication; 3) reported phonological or reading-related outcomes; 4) assessed outcomes pre- and post-intervention; 5) provided sufficient data to extract effect sizes; 6) intervention group received more music instruction than control group; 7) studies need to provide equivalent amounts of reading instruction across the intervention & control groups	None specified	Educational
Hetland	2000	Music	Meta-analysis	Does active instruction in music enhance preschool and elementary students' performance on spatial tasks?	7	Yes	Inception-1998	1) experiments reported in English that included a condition in which participants were taught to make music (vocal or instrumental); 2) one or more control conditions in which participants did not receive instruction in actively making	None specified	Cognitive

								music; 3) outcome measure of performance on one or more spatial tasks that required mental rotation and/or spatial visualization; 4) studies had to provide statistics necessary to compute an effect size		
Hwang & Braun	2015	Dance	Narrative	Examine the effectiveness of dance programs in improving the physical health of all older adults, both those with health conditions and those considered healthy	1	Yes	Inception-2013	1) evaluate the benefits of a dance intervention to the physical health of older adults (aged 65+) lacking prior dance experience; 2) present results in quantitative format	1) 'aerobic fitness classes taught to music' (i.e. Zumba, step-aerobics); 2) non-English publications	Physical function
Jaschke et al.	2013	Music	Narrative	To reflect the variation in published results in the field of music education and the far transfer effect and to show how difficult it is to interpret these results when different methods are used to measure them and their discussion is hampered by the absence of a proper classification of far transfer and the lack of a structured understanding of music and musicality'	6	Yes	2001-2013	Studies (RCT or non-randomized) of the effect of music education or participation on reading, spatial reasoning, writing, mathematics and intelligence in youth aged 4 to 13	1) Studies excluded when effects measured on the basis of study size rather than sample size; 2) no control group; 3) non-randomized longitudinal studies lasting <12 months; 4) meta-analyses; 5) analyzed near transfer	Cognitive; Educational
Keogh et al.	2009	Dance	Narrative	Review the physical benefits of dancing for healthy older adults	5	Yes	Inception-2009	1) apparently healthy older adults 60+ years old; 2) Compare a group of older dancers with an age matched group of nondancers or involve a dance intervention that lasted at least 8 weeks	None specified	Bone health
Letton, Thom, & Ward	2020	Dance	Narrative	Systematically identify and synthesize literature investigating the effectiveness of classical ballet training interventions on physical healthy and psychological outcome measures	10	Yes	Inception-2019	Original research investigating a classical ballet training intervention (>/= 4 weeks) or reporting on observations of preexisting ballet classes or reporting on observations of pre-exiting ballet classes; reporting on measurable health outcomes relating to physical health, psychological functioning, or physiological health	1) Review articles, single case studies; 2) non-English articles; 3) combined ballet training with other exercise or types of dance	Body composition; Developmental; Physical fitness; Physical function
Liu, Shen & Tsai	2020	Dance	Meta-analysis	Conduct a systematic analysis of random controlled research on the effectiveness of dance intervention in the physical function of healthy older adults	5	Yes	Inception-2018	1) RCT; 2) intervention duration at least 6 weeks; 3) healthy older adults aged at least 65; 4) English	1) Uncertain study design/data; 2) dance intervention combined with other exercise	Physical function; Self-reported health/wellbeing
			Narrative							Physical fitness; Physical function

Meng et al.	2020	Dance	Meta-analysis	Assess the effects of dance interventions on global cognition, executive function and memory in older adults	9	Yes	Inception-2018	1) RCT or quasi-experimental; 2) participants were aged 60+; 3) Dance intervention (any style) longer than 4 weeks; 4) cognitive function reported as the primary or secondary outcome	1) combined dance & other intervention	Cognitive
			Narrative							Cognitive
Moore	2013	Music	Narrative	Explore and synthesize results examining the effects of music on neural structures implicated in emotion regulation; Create preliminary clinical considerations for structuring the music stimulus when facilitating emotion regulation	8	No	Inception-2011	1) Primary research study; 2) participants are typically-developing humans, with no restrictions as to age, gender, ethnicity, or type of setting; 3) music was the primary stimulus, regardless of how it was implemented, the genre of music, or the music instruments incorporated; 4) study results reported on the impact of music on one or more of the following structures (amygdala, anterior cingulate cortex, orbitofrontal cortex, and lateral prefrontal cortex); 5) articles published in English language peer-reviewed journals	1) Review articles/theoretical paper; 2) participants had a disorder, brain damage, or a syndrome; 3) study of rhythm was associated with circadian rhythm, cardiac rhythm, respiratory rhythm, dietary rhythm, and other nonmusical references to rhythm	Mental health
Phillips & Becker	2019	Music *(no studies of dance meeting inclusion criteria)*	Narrative	To synthesize evidence about the effectiveness of expressive arts interventions used to reduce psychosocial stress among healthcare workers	4	No	1997 - 2017	1) intervention studies that address workplace stress, professional grief, compassion fatigue, secondary traumatic stress, or burnout; 2) interventions that used the expressive arts (music, poetry, narrative, storytelling, writing, singing, dancing, visual, or tactile art; 3) Quantitative and mixed methods studies that evaluated the effectiveness or acceptability of the intervention; 4) intervention studies that targeted any healthcare worker and/or any healthcare setting; 5) articles written in English	1) intervention not aimed at workplace stress and/or did not use the expressive arts; 2) intervention was for non-healthcare workers and/or students; 3) article was a systematic review, meta-analysis, primarily qualitative study, case report, dissertation/thesis or conference abstract	Mental health; Self-reported health/wellbeing
Raglio et al.	2019	Music	Narrative	Select the evidence-based studies that took advantage of music to improve work-related psychological conditions and cognitive performances, highlighting perspectives in the implementation of music intervention in occupational medicine	2	No	2000 - 2017	RCTs or clinical controlled trials in English from peer-reviewed journals	None specified	Mental health; Self-reported health/wellbeing; Social functioning
Rodrigues-Krause et al	2016	Dance	Narrative; Meta-analysis	Verify the level of evidence regarding the adaptations of dance interventions on CVR factors in the elderly	5	Yes	1980 - 2015	1) RCTs and nRCTs comparing dance interventions with a non-exercising control group and/or other exercising groups receiving another intervention; 2)	1) Less than 4 weeks of intervention; 2) elderly patients with neurodegenerative diseases; 3) combined interventions (exercise +	***All studies meeting umbrella review inclusion criteria included in other reviews***

								Elderly subjects (mean age >60 years) with or without health conditions; 3) Designed to improve outcomes of interest; 4) Data reported as means or difference in between means and respective dispersion values at baseline and after the intervention period; 5) English, Portuguese or Spanish full text	dance); 3) duplicate publications/sub-studies; 5) studies including outcomes of no interest (i.e. aerobic fitness estimated by the 6-minute walk test)	
Rodrigues-Krause, Krause, Reischak-Oliveira	2019	Dance	Narrative	Review the literature on the use of dance as a form of intervention to promote functional and metabolic healthy in older adults	5	Yes	1980 - 2016	1) RCT; 2) quasi-experimental trials, including pre- and postintervention studies with 1 group, nonrandomized controlled trials and studies with other nonrandom distributions; 3) observational designs - cross-sectional or cohorts	1) evaluated older patients with cognitive and/or emotional impairment; 2) dance environment categorized as a dance videogame or dance on the water; 3) had study designs that did not fit the current study's protocols or were case studies	Bone health; Cognitive; Glucose/insulin; Lipid lipoprotein profile; Mental health; Physical fitness; Physical function; Self-reported health/wellbeing;
Skingley & Vella-Burrows	2010	Music	Narrative	Can music and singing contribute to the health and wellbeing of older people and are they relevant to the role of nurses?	2	Yes	1997-2010	1) Empirical research studies or reviews of music or singing interventions for older people (65 years+); 2) Qualitative studies exploring music and singing for older people; 3) Studies published in English; 4) Studies appearing in nursing and other arts and health journals	1) Studies of music therapy; 2) Studies unrelated to older people; 3) Studies where the intervention is primarily relevant to health professionals other than nurses	***All studies meeting umbrella review inclusion criteria included in other reviews***
Talamini et al	2017	Music	Meta-analysis	Whether there really is a difference between musicians and nonmusicians and whether the magnitude of any difference varies as a function of the memory system involved, and the type of stimuli presented	9	Yes	Inception-2017	1) adult participants; 2) study included a group of expert musicians ('participants who attended music conservatories or music schools') and a group of nonmusicians; 3) studies that administered a memory task to both groups related to long-term, short-term or working memory; 4) studies using verbal, visual, spatial or tonal stimuli; 5) studies published in English	1) Missing data; 2) tasks not comparable with other studies considered	Cognitive
Teixeira-Machado, Arida & de Jesus Mari	2019	Dance	Narrative	What is the influence of dance practice on neuroplasticity in already mature brains?	3	No	Inception-2018	1) randomized clinical trial studies that observed the maturation and activation of the central nervous system through dance; 2) studies on humans of all ages and both genders included; 3) dance interventions and research comparing dancing with other interventions (i.e. physical exercise, sports)	None specified	Cognitive; Physical fitness; Physical function; Self-reported health/wellbeing; Social functioning
Vaughn	2000	Music	Meta-analysis	Conduct meta-analyses investigating the relationship between music and mathematics	7	Yes	Inception-1998	1) studies testing an association between music and mathematics; 2) studies with a measured math outcome; 3) studies with a control group; 4) sufficient statistical	1) studies in which music was used as a reward for good performance in math; 2) studies in which musical jingles were used as memory aids to	Educational

								information for an effect size to be computed	teach math; 3) studies assessing whether individuals who have a high musical aptitude also have a high mathematical aptitude	
Veronese et al.	2017	Dance	Narrative	Investigate if dancing is more effective than usual care in preventing falling and improving fear of falling through a systematic revision of the RCTs available	5	Yes	Inception - 1 March 2017	1) RCT; 2) investigated the effect of a dancing program; 3) included data regarding falls or fear of falls using validated scales	1) did not include humans; 2) did not include a control group; 3) did not use validated scales for assessing fear of falls; 4) did not report data at follow-up evaluation regarding falls/fear of falling	Physical function
Yap, Kwan & Ang	2017	Music	Narrative	To provide a systematic review on the current literature of the effects of rhythm-based music making on different aspects of health	2	Yes	Inception-October 2015	English-language peer reviewed journal articles that studies the effects of drumming or percussion music on an individual	1) Review articles, meta-analyses, case series, case reports; 2) studies without full articles; 3) studies that included drumming or percussing music making as part of a protocol with the inclusion of other intervention modalities	Blood pressure; Immune function/inflammation; Mental health; Self-reported health/wellbeing; Social functioning

Table S6. GRADE publication bias ratings for included reviews containing quantitative synthesis (meta-analysis). NB: publication bias ratings of 'Serious' and 'Very Serious' resulted in a down-rating of the certainty of corresponding review-level evidence by 1 or 2 levels, respectively, as per GRADE guidelines. Evidence evaluated as per criteria established and validated in:

Meader et al. (2014) A checklist designed to aid consistency and reproducibility of GRADE assessments: development and pilot validation. *Systematic Reviews*.

		**GRADE publication bias evaluation**						
**Author**	**Year**	*Did the authors conduct a comprehensive search?*	*Did the authors search for grey literature*	*Authors did NOT apply restrictions to study selection on the basis of language?*	*There was no industry influence on included studies?*	*(meta-analysis only) There was no evidence of funnel plot asymmetry?*	*There was no discrepancy in findings between published and unpublished trials?*	**GRADE publication bias**
Butzlaff	2000	Yes	Yes	No	Yes	Yes	N/A (not analyzed)	Uncertain
Fong Yan et al.	2018	Yes	Yes	Yes	Yes	Yes	N/A (not analyzed)	Uncertain
Gordon, Fehd & McCandliss	2015	No	No (explicit exclusion of grey literature)	No	Yes	Somewhat (no funnel plot asymmetry, but significant heterogeneity)	N/A (not analyzed)	Very serious (significant heterogeneity, plus no hand searches of included articles, exclusion of grey literature, and language restriction)
Hetland	2000	Yes	Yes	No	Yes	Yes	N/A (not analyzed)	Uncertain
Liu, Shen & Tsai	2020	Yes	No	No	Yes	Yes	N/A (not analyzed)	Serious (restricted to English studies + no grey literature)
Meng et al.	2020	Yes	Yes	Yes	Yes	Unclear	N/A (not analyzed)	Uncertain
Talamini et al	2017	Yes	No (but statistical corrections for potential publication bias from this exclusion)	No	Yes	Yes	N/A (not analyzed)	Uncertain
Vaughn	2000	Yes	Yes	No	Yes	Yes	N/A (not analyzed)	Uncertain

Table S7. Details of individual studies in included reviews of dance participation.

Author	*Year*	*In other review / other exclusion?*	*Industry support?*	*Design*	*Subjects*	*Study country*	*Type of dance participation*	*Length of participation*	*Frequency/ duration of each session*	*Outcome*	*Effect of dance participation*	*Outcome details*	*GRADE Certainty of Evidence*
Burkhardt & Brennan, 2012													
Adiputra et al	1996		No	Non-randomized experimental study	60 male students of the Senior Dancing School of Denpasar, Bali (age range 17-19 years; mean 19.6)	Indonesia	Balinese dance	8 weeks	50 min/3x weekly	VO2 max	Positive	Significant increase in VO2 max in dance vs control group	Low
Bennell et al	2000		No	Cross-sectional	130 females aged 8-11 (mean age = 9.6; 78 pre-and early pubertal novice dancers, 52 age-matched controls)	Australia	Ballet + various	Unspecified	Unspecified	Bone mineral density	Positive	Significantly greater bone mineral density in dancers vs controls (hip & femoral neck)	Low
Blackman et al	1988		No	Non-randomized experimental study	16 females (age range 14-15; 8 on dance team, 8 physical education control	USA	Dance team	5 months	1 hour/5x weekly	VO2 max	Positive	Significant pre-post increase in VO2 max in dance group	Very Low
										Sit and reach	No effect	No pre-post effect	Very Low
										Strength	Positive	Significant pre-post increase in dance group (1-RM bench press)	Very Low
										% body fat	Mixed	Significant pre-post difference in total skinfold measurements, but not hydrostatic weighing (skinfolds, hydrostatic weighing)	Very Low
										Self esteem	NO effect	No significant effects (Coopersmith Self-Esteem Inventory, Tennessee Self-Concept Scale)	Very Low
										Body satisfaction (Cathexis)	No effect	No significant effects (Body Cathexis Scale)	Very Low
										Blood Pressure	Positive	Significant pre-post decrease in blood pressure	Very Low
										Body mass	No effect	No significant pre-post effect	Very Low

Burgess, Grogan & Burwitz	2006	No	RCT	50 healthy British schoolgirls (mean age = 13.5; 25 dance group, 25 physical education)	UK	Aerobic dance	6 weeks	50 min/2x weekly	Self-perception	Positive	Significant group*time effects (Children & Youth Physical Self-Perception Profile)	Moderate
		Other review - Fong Yan et al., 2018							BMI			
Daley & Buchanan	1999	No	Non-randomized experimental study	113 females (age range 15-16; 43 aerobic dance group, 70 physical education control)	UK	Aerobic dance	5 weeks	1 hour/weekly	Self-perception	Positive	Significant group*time effects (Physical Self Perception Profile)	Low
Flores	1995	No	RCT	81 children (age range 10-13; 43 dance group, 38 physical activity control), sex unspecified	USA	Hip-hop, African dance, & aerobic	12 weeks	50 min/3x weekly	Timed mile run	No effect	No significant pre-post or intervention vs control differences	Moderate
									BMI	Positive	Significant pre-post reduction vs control	Moderate
Kim & Kim	2007	No	RCT	277 high school and undergraduate students (mean age = 20.6; 45 hip hop dancing group - all female, 84 aerobic exercise group - 40 male/44 female, 64 body conditioning group - 44 male, 20 female, 84 ice skating group - 60 male/24 female)	South Korea	Hip hop	50 min	Once	Mood	Positive	Significant increase in positive well-being and decrease in psychological distress and fatigue in dance & aerobics groups vs ice skating & body conditioning groups (Subjective Exercise Experiences Scale)	Moderate
Leste & Rust	1990	No	Non-randomized experimental study	84 university students (mean age = 19.9; 23 dance group, 16 sport group, 7 music group, 38 mathematics group), sex breakdown unspecified - mixed	UK	Modern	12 weeks	Unspecified	Anxiety	Positive	Significant benefit of dance intervention vs music/physical exercise/math groups (Spielberger State-Trait Anxiety Inventory)	Low
Matthews et al	2006	No	Prospective cohort study	143 girls (age range 8-14; 82 non-elite dancers, 61 controls)	Australia	Ballet	3 years	Unspecified	Bone mineral density	Positive	Significantly greater bone mineral content in dancers vs controls (total body - DXA)	Low
Mavridis et al	2004	Other review - Fong Yan et al., 2018										

Robinson et al	2003		RCT	61 African American females (age range 8-10, BMI > 50th percentile for age with at least 1 overweight parent/guardian (BMI = 25+)	USA	Hip hop, African dance & step aerobics	12 weeks	3x weekly	BMI	No effect	No significant group*time interaction (study underpowered to detect these differences)	Low
									Waist circumference	No effect	No significant group*time interaction (study underpowered to detect these differences)	Low
									Insulin	Not reported		
									Glucose	Not reported		
									Total cholesterol	Not reported		
									HDL cholesterol	Not reported		
									Triglycerides	Not reported		
									Self-esteem	No effect	No significant group*time interaction (study underpowered to detect these differences)(Rosenberg self-esteem scale)	Low
Silvestri	2004	Excluded - dance with target HR (exercise, not performing arts)										
Steinberg et al	2008		Cross-sectional	1708 young females (age range 8-16; 1482 dancers, 226 non-dancers)	Israel	Ballet and various	Unspecified	Mean 2.4-11.3 hours weekly dance	Musculoskeletal development	No effect	No significant differences between dance & control groups (14 anthropometric measurements)	Low
Viscki-Stalec et al	2007	No	Non-randomized experimental study	220 3rd & 4th grade girls (115 aerobic dance group, 105 physical education group; unspecified age)	Croatia	Aerobic dance	60 high school periods	Unspecified	Core strength	Positive	Significant group* time effect (Trunk Lift Test)	Low
									Power	Positive	Significant group*time effect (standing long jump)	Low
									Agility	Positive	Significant group*time effect (side step test)	Low
		Other review - Fong Yan et al., 2018							% fat mass; Body mass; Range of motion; Sit & reach; Step test			
Clarke et al., 2018												

Ambegaonkar et al.	2013		Cross-sectional	33 female dancers and nondancers (18 dancers, mean age = 20; 15 nondancers, mean age = 22.1)	USA	Modern dance	mean 13.1 years dance experience	Unspecified	Balance	Positive	Significantly better balance performance in dancers vs non-dancers (Balance Error Scoring System & SEBT)	Low
Ambegaonkar et al.	2016	Excluded - single group descriptive analysis of dancers only										
Barcellos & Imbiriba	2002	Excluded - single group descriptive analysis of dancers only										
Batson	2010	Excluded - single group descriptive analysis of dancers only										
Bronner	2012	No	Cross-sectional	27 dancers (9 expert, 5 male/4 female, mean age = 24.9; 9 advanced, 2 male/7 female, mean age = 19.6; 9 intermediate, 4 male/5 female, mean age = 19.8)	USA	Unspecified	Expert - mean 13.3 years dancing; advanced - mean 11.7 years dancing; intermediate - mean 6.1 years dancing	Unspecified	Balance	Positive	Significantly reduced postural variability in advanced/expert dancers vs intermediate	Low
Bruyneel et al.	2010	Excluded - single group descriptive analysis of dancers only										
Bruyneel et al.	2010	Excluded - single group descriptive analysis of dancers only										
Caplan & Gibson	2011	Excluded - single group descriptive analysis of dancers only										
Casabona et al.	2016	No	Cross-sectional	20 female dancers & non-dancers (10 professional dancers, mean age = 23.7; 10 untrained dancers, mean age = 27.6)	Italy	Ballet	Unspecified	Unspecified	Balance	Positive	Significantly better balance in dancers vs untrained, albeit only in 'duck' stance familiar to dancers	Low
Clark & Redding	2012	Excluded - single group descriptive analysis of dancers only										
Cloak et al.	2010	Excluded - analysis of intervention study evaluating the impact of vibration training on balance in dancers only										
Coutts et al.	2006	Excluded - single group descriptive analysis of dancers only										
Crotts et al.	1996	No	Cross-sectional	30 university students (mean age 27; 15 dancers; 15 non-dancers from physical therapy department), sex breakdown unspecified - mixed	USA	University dance major (varied)	mean 14.9 years dance experience	Unspecified	Balance	Positive	Significantly better balance in dancers in challenged visual & surface conditions	Low

da Costa et al.	2013	Excluded - single group descriptive analysis of dancers only										
Denardi, Ferraciolo & Rodrigues	2008	Excluded - single group descriptive analysis of dancers only										
Gerbino, Griffin & Zurakowski	2007	No	Cross-sectional	64 female university students (32 dancers - mean age = 20.3; 32 soccer players - mean age = 19.7)	USA	Modern dance & ballet	Unspecified	mean 5.5 hours/day, 5.6 days/week	Balance	Positive	Significantly better balance performance by dancers in 5 of 20 measures	Low
Golomer & Dupui	2000	No	Cross-sectional	41 dancers and nondancers (23 dancers - mean age 23.6; 13 female/10 male; 18 untrained - mean age - 21.5; 11 female/7 male)	France	Opera	Unspecified	Unspecified	Balance	Positive	Significantly better balance in dancers vs controls	Low
Golomer et al.	1999	Excluded - single group descriptive analysis of dancers only										
Golomer et al.	1999	No	Cross-sectional	46 professional dancers & untrained males (17 professional dancers - mean age 23.8; 29 untrained - mean age ~22.6)	France	Ballet	Unspecified	Unspecified	Balance	Positive	Dancers displayed significantly improved balance in eyes closed tasks vs. untrained	Low
Golomer et al.	2009	No	Cross-sectional	15 female dancers & untrained individuals (8 dancers, 7 untrained; mean age = 19)	France	Ballet	Unspecified	Unspecified	Balance	Positive	Significantly better shoulder-hip stabilization in dancers	Low
Golomer et al.	2010	No	Cross-sectional	14 professional and untrained female dancers (mean age = 19)	France	Ballet	Minimum 11 years dance training	20 hours/week from age 8-17 + 1-year professional training (35 hours/week)	Balance	Mixed	Significantly better balance in dancers vs control in 1-leg stance conditions; significantly worse performance by dancers vs control in 'roll equilibrium' conditions	Very Low
Golomer, Dupui & Monod	1997	No	Cross-sectional	52 post-pubescent adolescents (31 professional dancers - mean age ~17.8, 15	France	Ballet	Minimum 1 year professional experience	Unspecified	Balance	Positive	Significantly better balance in dancers vs. untrained cohort (4	Low

				male/16 female; 21 untrained - mean age 18.6, 10 male/11 female							conditions - 2 eyes open, 2 eyes closed)	
Golomer, Gravenhorst & Toussaint	2009	Excluded - single group descriptive analysis of dancers only										
Guillou, Dupui & Golomer	2007	No	Cross-sectional	36 male dancers, gymnasts, athletes & laypeople (7 dancers, mean age = 18; 9 acrobatic gymnasts, mean age = 19.1; 10 professional soccer players, mean age = 17.1; 10 untrained, mean age = 21.4)	France	Opera	Unspecified	Unspecified	Balance	No effect	No significant differences between dancers & untrained	Low
Hopper et al.	2014	No	Cross-sectional	23 dancers of varying experience levels (no gender listed) (9 professional dancers, mean age = 18.8; 6 pre-professional dancers, mean age = 17; 8 recreational dancers, mean age = 20.6)	Australia	Ballet	Professional - mean 12.3 years training; pre-professional - mean 12.7 years training; recreational - mean 16 years training	Professional - mean 20 hours/week; pre-professional - mean 15 hours/week; recreational - mean 3.7 hours/week	Balance	Positive	Significantly better balance after turns and fatiguing activity in professional vs pre-professional & recreational dancers	Low
Hugel et al.	1999	No	Cross-sectional	64 professional & nondancers (18 dancers - age range 16-35, 6 male/12 female; 46 nondancers - age range 16-37)	France	Ballet	Mean 10-15 years training	Unspecified	Balance	Positive	Dancers displayed significantly improved balance in eyes open tasks vs. untrained	Low
Jarvis, Smith & Kulig	2014	No	Cross-sectional	20 female dancers & non-dancers (10 professional dancers, mean age = 27.1; 10 non-dancers, mean age = 24.8)	USA	Unspecified	Minimum 10 years dance training	Unspecified	Balance	Positive	Significantly reduced variability in intersegmental coordination in dancers vs non-dancers	Low
Kiefer et al.	2011	Yes (Cincinnati Ballet Company)	Cross-sectional	56 dancers and non-dancers (28 professional dancers, 10 male/18 female,	USA	Ballet	Unspecified	Unspecified	Balance	Positive	Significantly more consistent ankle hip coordination in	Low

				mean age = 23.6; 28 untrained, 10 male/18 female, mean age = 23.4)							postural corrections in dancers	
Kilroy et al.	2016	No	Cross-sectional	14 female university students (age range 18-23; 7 dancers, 7 non-dancers)	USA	Unspecified	Minimum 7 years dance experience	Unspecified	Balance	Positive	Significantly better postural control in dancers vs non-dancers	Low
Krityakiarana & Jongkamonwiwat	2016	No	Cross-sectional	50 female university students (25 dancers, mean age = 21.2; 25 non-dancers, mean age 21.2)	Thailand	Thai classical dance	mean 11.4 years dace experience	4 hours/day, 3-4 days/week	Balance	Positive	Significantly better postural stability in dancers vs. non dancers in the majority of conditions	Low
Li et al.	2014	Excluded - single group descriptive analysis of dancers only										
Lin et al.	2011	No	Cross-sectional	22 university females (mean age = 19.9; 11 uninjured dancers, 11 non-dancers)	Taiwan	Ballet	minimum 7 years training	Unspecified	Balance	No effect	No significant differences in any balance (postural sway) measure between dancers & non-dancers	Low
Lin et al.	2014	No	Cross-sectional	18 female dancers (9 super experienced dancers, mean age = 18.2; 9 experienced dancers, mean age = 18.3)	Taiwan	Ballet	novice - 2-5 years ballet training; experienced/ super-experienced (stratified based on maintenance of retire position for min 7 sec with each leg) - at least 6 years ballet training	novice - 1.5 - 3 hours training per week; (super)experienced - at least 3 hours training per week	Balance	Mixed	Experienced dancers had significantly better balance on the non-dominant leg; super experienced dancers had a greater maximum COM-COP distance in AP direction	Low
Lin et al.	2014	Excluded - single group descriptive analysis of dancers only										
Lin, Su & Wu	2005	Excluded - single group descriptive analysis of dancers only										
Mertz & Docherty	2012	Excluded - single group descriptive analysis of dancers only										
Morrin & Redding	2013	Excluded - single group descriptive analysis of dancers only										

Pappas et al.	2011	Excluded - single group descriptive analysis of dancers only										
Perez et al	2014	No	Cross-sectional	48 female university students (18 dancers, mean age 23.3; 30 non-dancers, mean age 22.2)	Spain	Ballet & contemporary	Minimum 5 years dance training	Unspecified	Balance	Positive	Significantly better balance in dancers vs non-dancers in eyes open conditions	Low
Perrin et al.	2002	No	Cross-sectional	56 professional dancers & nondancers (14 female dancers - mean age 22.1; 42 nondancers - mean age 23.9, 21 male/21 female)	France	Ballet	Mean 10-15 years training	Unspecified	Balance	Mixed	Significantly better balance in dancers vs controls in eyes closed conditions; significantly worse balance in dancers vs controls in eyes open conditions	Low
Rein et al.	2011	Yes (Aircast Europe)	Cross-sectional	90 dancers & non dancers (30 professional dancers - mean age = 27, 20 female/10 male; 30 amateur dancers - mean age = 34, 20 female/10 male; 30 non-dancers, mean age = 31, 15 female/15 male)	Germany	Unspecified	Unspecified	Unspecified	Balance	Mixed	Significantly better balance performance in professional, but not amateur, dancers vs control	Very Low
Schmit, Regis & Riley	2005	No	Cross-sectional	20 university students (10 dancers - mean age 20, 5 female/5 male; 10 varsity track runners - mean age = 19.5, 5 female/5 male)	USA	Ballet	Minimum 5 years dance training	Unspecified	Balance	No effect	No significant differences in balance in dancers vs track athletes	Low
Schmitt, Kuni & Sabo	2005	No	Cross-sectional	82 dancers & non-dancers (42 dancers, 31 female/11 male, mean age = 18.3; 40 untrained, 29 female/11 male, mean age = 19.5)	Germany	Ballet & modern dance	Minimum 7 years	23-30 hours/weekly	Balance	Positive	Significantly better balance in dancers vs controls	Low
Simmons	2005	No	Cross-sectional	34 female community/university dancers & untrained	USA	Ballet	Mean 10.9 years ballet training	Unspecified	Balance	Mixed	Significantly worse balance control in dancers vs. control in some conditions;	Very Low

				individuals (mean age 21.5)							significantly better balance control in dancers vs controls in others	
Simmons	2005	No	Cross-sectional	31 female community/university dancers and untrained individuals (15 dancers - mean age 21.4; 16 untrained - mean age 21.2)	USA	Ballet	mean 10.5 years ballet training	Unspecified	Balance	Positive	Significantly faster and more consistent long-latency balance responses in dancers vs controls; no significant differences between groups for short- or medium latency responses	Low
Thullier & Moufti	2004	No	Cross-sectional	12 participants (6 elite ballet dancers, 6 gymnasts; no age or gender specified)	France	Ballet	Unspecified	Unspecified	Balance	Positive	Dancers & gymnasts equally stable	Low
Wyon et al.	2013	Excluded - single group descriptive analysis of dancers only										
Zaferiou, Wilcox & McNitt-Gray	2016	Excluded - single group descriptive analysis of dancers only										
Costa, Ferreira & Felicio, 2013												
Barcellos & Imbiriba	2002	Excluded - descriptive analysis of dancers only (no comparison group)										
Bruyneel et al	2010	Excluded - descriptive analysis of dancers only (no comparison group)										
Cheng et al	2011	No	Cross-sectional	51 female adolescents (26 dancers - mean age 17.5; 25 healthy active students - mean age 18.1)	Taiwan	Ballet; Modern	Mean 5.8 years dance experience	3 hours+/weekly	Balance	Positive	Significantly improved postural stability in single leg standing condition (dominant leg; eyes open & closed)	Low
Denardi, Ferraciolo & Rodrigues	2006	Excluded - descriptive analysis of dancers only (no comparison group)										
Gerbino, Griffin & Zurakowski	2007	Other review - Clarke et al., 2018										
Gillou, Dupu & Golomer	2007	No	Cross-sectional	36 right handed & right footed male adults (7 professional dancers – mean age = 18; 9 acrobatic gymnasts – mean age	France	Ballet	Unspecified	Unspecified	Balance	Positive	Significantly reduced roll energy in dancers vs control; significantly greater spectral energy in dancers vs soccer	Low

				= 19.1; 10 professional second division soccer players – mean age = 17.1; 10 inactive control – mean age = 21.$)							players during left leg stance; no other significant differences (unipedal stance on unstable seesaw platform)	
Golomer et al	1999	Excluded – analysis of dancers only (multiple groups of young/older dancers but analysis of experience unclear)										
Golomer et al.	1999	Other review - Clarke et al., 2018										
Lin et al.	2011	Other review - Clarke et al., 2018										
Lin, Su & Wu	2005	Other review - Clarke et al., 2018										
Lobo da Costa	2012	Excluded - descriptive analysis of dancers only (no comparison group)										
Perrin et al	2002	Other review - Clarke et al., 2018										
Rein et al	2011	Other review - Clarke et al., 2018										
Schmitt, Kuni & Sabo	2005	Other review - Clarke et al., 2018										
Simmons	2005	Other review - Clarke et al., 2018										
Thiesen & Sumiya	2011	No	Cross-sectional	15 female dancers (mean age = 13.8; 9 beginner; 6 intermediate)	Brazil	Ballet	Mean 31;84 months experience (beginner; intermediate)	Unspecified	Balance	No effect	No significant differences between beginner & intermediate dancers (standing oscillation speed & plantar arch structure/function)	Low
Cugusi et al, 2019												
Araneta & Tanori	2015	No	Single group experimental study	13 overweight women with at least 2 metabolic syndrome risk factors (mean age = 52.5)	USA	Zumba	12 weeks	60 min/2x weekly	Blood pressure	Positive	Significantly reduced blood pressure pre-post intervention	Very Low
									Triglycerides	Positive	Significantly reduced triglycerides pre-post intervention	Very Low
									HDL cholesterol	Unclear	Significantly decreased HDL cholesterol pre-post intervention	Very Low
									Body mass	No effect	No significant pre-post change	Very Low

									Glucose	No effect	No significant pre-post change	Very Low
Barene et al	2014	Other review - Fong Yan et al., 2018										
Barene et al	2014	Other review - Fong Yan et al., 2018										
Cugusi et al	2016	No	Single group experimental study	27 overweight women (mean age = 38.9)	Italy	Zumba	12 weeks	50 min/2-3x weekly	Blood pressure	Positive	Significantly reduced blood pressure pre-post Zumba intervention	Very Low
									% fat mass	Positive	Significantly reduced fat mass pre-post Zumba intervention	Very Low
									Waist circumference	Positive	Significantly reduced waist circumference pre-post Zumba intervention	Very Low
									Body mass	Positive	Significantly reduced body mass pre-post Zumba intervention	Very Low
									BMI	Positive	Significantly reduced BMI pre-post Zumba intervention	Very Low
Delextrat et al.	2016	No	RCT	44 healthy, low active women (22 Zumba group - mean age = 26.6; 22 control – mean age = 27.9)	UK	Zumba (home DVD)	8 weeks	60 min/3x weekly	VO2 max	Positive	Significant improvement in VO2 max vs control	High
									% fat mass	No effect	No significant change vs control(DEXA)	High
									Body mass	No effect	No significant change vs control	High
Domene et al	2016	No	RCT	20 overweight and sedentary women (mean age = 34)	UK	Zumba	8 weeks	60 min/1-2x weekly	Blood pressure	No effect	No pre-post effect of dance or control	Low
									Body mass	No effect	No pre-post effect of dance or control	Low
									% fat mass	No effect	No pre-post effect of dance or control	Low
									White blood cell count	Negative	Significantly reduced white blood cell count in dance group (pre-post analysis only; no comparison vs control – no significant pre-post difference in control group)	Low

									CRP	No effect	No pre-post effect of dance or control	Low
									IL-6	Unclear	Significantly reduced IL-6 in dance group (pre-post analysis only; no comparison vs control group – no significant pre-post difference in control group)	Low
									Total cholesterol to HDL ratio	No effect	No pre-post effect of dance or control	Low
									VO2 max	Positive	Significantly improved VO2 max in dance group (pre-post analysis only; no comparison vs control group – no significant pre-post difference in control group)	Low
									BMI	No effect	No pre-post effect of dance or control	Low
Donath et al	2014	No	RCT	30 healthy women (mean age = 21)	Switzerland	Zumba	8 weeks	60 min/2x weekly	6-minute walk test	Positive	Significantly increased 6MWT distance in dance vs control group	High
									BMI	Positive	Significantly reduced BMI in intervention vs control group	High
Krishnan et al	2015	No	Single group experimental study	41 obese women (mean age = 49.3)	USA	Zumba	16 weeks	60min/3x weekly	Body mass	Positive	Significant pre-post reduction	Very Low
									BMI	Positive	Significant pre-post reduction	Very Low
									% body fat	Positive	Significant pre-post reduction (bioelectrical impedance)	Very Low
									Waist circumference	Positive	Significant pre-post reduction	Very Low
									Blood pressure	No effect	No significant pre-post change	Very Low
									Glucose	No effect	No significant pre-post change	Very Low
									HbA1c	No effect	No significant pre-post change	Very Low

									Insulin	No effect	No significant pre-post change	Very Low
									Total cholesterol	No effect	No significant pre-post change	Very Low
									HDL cholesterol	No effect	No significant pre-post change	Very Low
									Triglycerides	No effect	No significant pre-post change	Very Low
									VO2 max	Positive	Significant pre-post increase (estimated from 1 mile walk time)	Very Low
Micallef	2014	No	Single group experimental study	36 overweight women (BMI 25+; mean age = 34.3)	Malta	Zumba	8 weeks	60 min/2x weekly	Body mass	Positive	Significant pre-post reduction	Very Low
									BMI	Positive	Significant pre-post reduction	Very Low
Rossmeissl et al	2016	No	Single group experimental study	17 overweight women (median age = 54)	Switzerland	Zumba	12 weeks	60 min/3x weekly	VO2 max	No effect	No significant pre-post change	Very Low
									Body mass	No effect	No significant pre-post change	Very Low
									Waist circumference	No effect	No significant pre-post change	Very Low
									BMI	No effect	No significant pre-post change	Very Low
									Blood pressure	No effect	No significant pre-post change	Very Low
									Resting HR	No effect	No significant pre-post change	Very Low
									% fat mass	No effect	No significant pre-post change	Very Low
Fernandez-Arguelles et al., 2015												
Eyigor et al	2009	Other reviews - Liu, Shen & Tsai, 2020; Rodrigues-Krause, Krause & Reischak-Oliveira, 2019										
Granacher et al	2012	Other reviews - Liu, Shen & Tsai, 2020; Rodrigues-Krause, Krause & Reischak-Oliveira, 2019										
Hui, Chui & Woo	2009	Other reviews - Liu, Shen & Tsai, 2020; Rodrigues-Krause, Krause & Reischak-Oliveira, 2019										
Holmerova et al	2010	Other review - Liu, Shen & Tsai, 2020										
Shigematsu et al	2002	Excluded – intensity of dance-based exercise program set at individual‘s lactate threshold (exercise, not performing arts, intervention)										
Sofianidis et al	2009	Other reviews - Liu, Shen & Tsai, 2020; Fong Yan et al., 2018										

Young, Weeks & Beck	2007	Excluded - dance a small part of a broader fitness program (foot stamping, progressively loaded squats & line dancing)											
Fong Yan et al., 2018													
Arzglou et al.	2013	Excluded - clinical population (Parkinson's), narratively analyzed outcomes only (postural stability)											
Barene et al.	2016		No	RCT	72 female hospital employees (mean age = 45; 35 Zumba group, 37 soccer group)	Norway	Zumba	40 weeks	1 hour/2x week	Sit & Reach	Meta-analysis		Moderate
										Postural stability	Positive	Significantly greater improvement in postural stability in Zumba vs. soccer groups	Moderate
Barene et al.	2014		No	RCT	72 female hospital employees (mean age = 45; 35 Zumba group, 37 soccer group)	Norway	Zumba	40 weeks	1 hour/2x week	Fat mass	Meta-analysis		Moderate
										VO2 max	Meta-analysis		Moderate
										Triglycerides	Meta-analysis		Moderate
										Glucose	No effect	No significant intergroup or pre-post changes	Moderate
										Peak ventilation	Meta-analysis		Moderate
										Blood pressure	No effect	No significant intergroup or pre-post changes	Moderate
										Respiratory exchange ratio	No effect	No significant intergroup or pre-post changes	Moderate
										Total cholesterol	Meta-analysis		Moderate
										Bone mineral density	No effect	No significant intergroup or pre-post changes	Moderate
										HDL cholesterol	Meta-analysis		Moderate
										Body mass	Meta-analysis		Moderate
										BMI	Meta-analysis		Moderate
Barene, Krustrup & Holtermann	2014	Excluded - unclear outcomes ('questionnaire', but not one listed in review table 3)											
Belardinelli et al.	2008	Clinical population, but included in meta-analysis	No	RCT	88 stable chronic heart failure patients (74 male/14 female; mean age = 59.5; 44 dance group, 44 cardio exercise group)	Italy	Waltz	8 weeks	21 min/3x weekly	VO2 max	Meta-analysis		High
										Triglycerides	Meta-analysis		High
										Total cholesterol	Meta-analysis		High
										HDL cholesterol	Meta-analysis		High

		Excluded - clinical population (chronic heart failure)								Glucose; Peak O2 pulse; Respiratory exchange ratio; VE/VCO2; VO2 at anaerobic threshold			
Burgess, Grogan & Burwitz	2006		No	RCT	50 healthy British schoolgirls (mean age = 13.5; 25 dance group, 25 physical education)	UK	Aerobic dance	6 weeks	50 min/2x weekly	BMI	Meta-analysis		Moderate
Ford et al.	1989		No	Longitudinal cohort study	88 healthy female university students (mean age = 19.8; 21 aerobic dance group, 17 jogging group, 22 progressive resistance training, 15 swimming group, 13 sport training)	USA	Aerobic dance	8 weeks	1 hour/3x weekly	Sit & reach	Meta-analysis		Low
										Sit ups	Positive	Significant pre-post improvement vs control in aerobic dance but not weight training group	Low
										Step test	No effect		Low
										Fat mass	Meta-analysis	(skinfold measurements)	Very Low
Garber, McKinney & Carleton	1992		No	RCT	25 university employees (12 male/13 female; mean age = ~37; 14 aerobic dance group, 11 cardio group)	USA	Aerobic dance	8 weeks	50 min/3x weekly	VO2 max	Meta-analysis		Moderate
										Peak ventilation	Meta-analysis		Moderate
										Respiratory exchange ratio	No effect	No significant intergroup or pre-post changes	Moderate
										Body mass	Meta-analysis		Moderate
										BMI	Meta-analysis		Moderate
Hackney, Kantorovich & Earhart	2007	Excluded - clinical population (Parkinson's), narratively analyzed outcomes only (gait velocity, postural stability)											
Hackney et al.	2007	Clinical population, but included in meta-analysis	No	RCT	19 idiopathic Parkinson's disease patients & matched controls (12 male/7 female; mean age = ~71; 9 Latin dance group, 10 progressive resistance training group)	USA	Latin dance	13 weeks	1 hour/20 classes over 13 weeks	Timed up and go	Meta-analysis		High
										Berg balance test	Meta-analysis		High
		Excluded - clinical population (Parkinson's)								Gait velocity			
Hackney & Earhart	2009	Excluded - clinical population (Parkinson's); narratively analyzed outcomes only (questionnaire)											
Hashimoto et al.	2015	Clinical population, but included in	No	RCT	32 Parkinson's disease patients (15 aerobic dance group - 3 male/12 females,	Japan	Aerobic dance	12 weeks	1 hour/weekly	Timed up and go	Meta-analysis		High
										Berg balance test	Meta-analysis		High

		meta-analysis			mean age = 67.9; 17 progressive resistance training group - 2 male/15 female, mean age = 62.7)								
Heffron, Davey & Cochrane	1997		No	Non-randomized experimental cohort study	29 postmenopausal women (mean age = 58; 14 aerobic dance group, 15 progressive resistance training group)	UK	Aerobic dance	10 weeks	1 hour/weekly	Bone mineral density	No effect	No significant intergroup or pre-post changes	Low
Janyacharoen et al	2013	Other review - Liu, Shen & Tsai, 2020 (included in meta-analysis)	No	RCT	38 healthy older women (20 folk/traditional dance group - mean age = 64.9; 18 physical education group - mean age = 66.8)	Thailand	Folk/traditional dance	6 weeks	40 min/3x weekly	Sit & Reach	Meta-analysis		High
		Other review - Liu, Shen & Tsai, 2020								6-minute walk test; sit to stand			
Kalsatou et al.	2014	Clinical population, but included in meta-analysis	No	RCT	34 Greek males with chronic heart failure (mean age = 67; 18 folk/traditional dance group, 16 cardio & progressive resistance training group)	Greece	Greek folk/traditional dance	8 months	1 hour/3x weekly	VO2 max	Meta-analysis		High
										Peak ventilation	Meta-analysis		High
										Berg Balance test	Meta-analysis		High
										Body mass	Meta-analysis		High
										BMI	Meta-analysis		High
		Excluded - clinical population (chronic heart failure)								Blood pressure; Peak O2 pulse; Sit to stand; VE/VCO2; VO2 at anaerobic threshold			
Kin Isler, Kosar & Korkusuz	2001	Clinical population, but included in meta-analysis	No	RCT	30 sedentary female college students (mean age = 21.1, 15 aerobic dance group, 15 cardio group)	Turkey	Aerobic dance	8 weeks	45 min/3x weekly	Triglycerides	Meta-analysis		High
										Body weight	Meta-analysis		High
										Total cholesterol	Meta-analysis		High
										HDL cholesterol	Meta-analysis		High
Kouli et al	2009		No	Non-randomized experimental study	57 5th graders (mean age = 10.6; 33 aerobic dance group, 24 physical education group), sex unspecified	Greece	Aerobic dance	8 weeks	45 min/3x weekly	Range of motion	Positive	Significantly greater improvement in dance vs. physical education group	Low
										Sit ups	Positive	Significantly greater improvement in dance vs. physical education group	Low

									1-mile walk/run	Positive	Significantly greater improvement in dance vs. physical education group	Low
									Sit & reach (each leg separated)	Positive	Significantly greater improvement in dance vs. physical education group	Low
									Push ups	Positive	Significantly greater improvement in dance vs. physical education group	Low
Mangeri et al.	2014	No	Non-randomized experimental study	100 Type 2 diabetic/obese adults (42 ballroom dance group - mean age = 58.5, 18 male/24 female; 58 cardio group - mean age = 59.4, 34 male/24 female)	Italy	Ballroom dance	6 months	2 hours/2x weekly	Blood pressure	No effect	No significant intergroup or pre-post changes	Low
									Glucose	No effect	No significant intergroup or pre-post changes	Low
									6-minute walk test	Positive	Significantly greater improvement in dance vs. cardio group	Low
									Triglycerides	Meta-analysis		Low
									Body mass	Meta-analysis		Low
									BMI	Meta-analysis		Low
									Waist circumference	Positive	Significant pre-post improvement in dance & cardio groups	Low
									Total cholesterol	Meta-analysis		Low
									HDL cholesterol	Meta-analysis		Low
Mavridis et al.	2004	No	Non-randomized experimental study	40 1st grade children (mean age = 6.6; 21 aerobic dance group, 19 physical education), sex unspecified	Greece	Aerobic dance	12 weeks	45 min/3x weekly	Range of motion	Positive	Significantly greater improvement in dance vs. physical education group	Low
									Sit ups	Positive	Significantly greater improvement in dance vs. physical education group	Low
									1-mile walk/run	Positive	Significant pre-post improvement in dance & physical education groups	Low

										Sit & reach (each leg separated)	Positive	Significantly greater improvement in dance vs. physical education group	Low
										Push ups	Positive	Significantly greater improvement in dance vs. physical education group	Low
										Skinfold thickness	Positive	Significantly greater improvement in dance vs. physical education group	Low
McKinley et al.	2008	Other review - Liu, Shen & Tsai, 2020; narratively synthesized outcomes only (Gait velocity, sit to stand)											
Milburn & Butts	1983	Dance with target HR, but included in meta-analysis	No	Non-randomized experimental study	34 female college students (15 aerobic dance group - mean age = 21.4; 19 jogging group - mean age = 19)	USA	Aerobic dance	7 weeks	30 min/4x weekly	VO2 max	Meta-analysis		Low
										Peak ventilation	Meta-analysis		Low
Rani & Singh	2013		No	RCT	75 college students (unspecified age & gender; aerobic dance & yoga groups)	India	Aerobic dance	6 weeks	30-60 min/3x weekly	Sit & reach	Meta-analysis		Moderate
										Sit ups	Positive	Significantly greater improvement in dance vs. yoga group	Moderate
										1-mile walk/run	Positive	Significantly greater improvement in dance vs. yoga group	Moderate
										Skinfold thickness	Positive	Significant pre-post improvement in dance & yoga groups	Moderate
Rehfeld et al.	2017	Other review - Teixeira-Machado, Arida & de Jesus Mari, 2019, narratively analyzed outcomes only (balance)											
Rios Romenets et al	2015	Clinical population, but included in meta-analysis	No	RCT	33 Parkinson's disease patients (18 Latin dance group - mean age = 63.2, 12 male/6 female; 15 physiotherapy exercises - mean age = 64.3, 7 male/8 female)	Canada	Latin	12 weeks	1 hour/2x weekly	Timed up and go	Meta-analysis		High
Shimamoto et al.	1998		No			Japan	Aerobic dance	12 weeks		VO2 max	Meta-analysis		Low

				Non-randomized experimental study	60 moderately obese Japanese women (37 aerobic dance group - mean age = 49.1; 23 cardio group - mean age = 51.7)				1 hour/2-3x weekly	VO2 at anaerobic threshold	Positive	Significant pre-post improvement in dance & cardio groups	Low
										Body mass	Meta-analysis		Low
										BMI	Meta-analysis		Low
										% fat mass	Meta-analysis		Low
										Waist circumference	Positive	Significant pre-post improvement in dance & cardio groups	Low
										Skinfold thickness	Positive	Significant pre-post improvement in dance & cardio groups	Low
Sofianidis, Dimitriou & Hatzitaki	2017		No	RCT	24 older adults (7 male/17 female, mean age = 70.7, 12 Latin dance group/12 pilates group)	Greece	Latin	12 weeks	1 hour/2x weekly	Postural stability	Positive	Significant pre-post improvement in dance & pilates groups	High
Viskic et al	2007		No	Non-randomized experimental study	220 3rd & 4th grade girls (115 aerobic dance group, 105 physical education group; unspecified age)	Croatia	Aerobic dance	60 high school periods	Unspecified	Sit & reach	Meta-analysis		Low
										Range of motion	Positive	Significantly greater improvement in dance vs. physical education group	Low
										Step test	Positive	Significantly greater improvement in dance vs. physical education group	Low
										Body mass	Meta-analysis		Low
										% fat mass	Meta-analysis		Low
Volpe et al.	2013	Clinical population, but included in meta-analysis	No	RCT	24 Parkinson's disease patients (mean age = 63.3; 13 male/11 female; 12 dance group/12 physiotherapy exercise group)	Italy	Folk/traditional dance	26 weeks	90 min/weekly	Timed up and go	Meta-analysis		High
										Berg balance test	Meta-analysis		High
Hwang & Braun, 2015													
Alpert et al.	2009	Other review - Rodrigues-Krause, Krause & Reischak-Oliveira, 2019											
Borges et al	2012	Other review - Rodrigues-Krause, Krause & Reischak-Oliveira, 2019											

Coubard et al	2011	Other review - Meng et al., 2020										
Eyigor et al.	2009	Other reviews - Liu, Shen & Tsai, 2020; Rodrigues-Krause, Krause & Reischak-Oliveira, 2019										
Ferrufino et al.	2011	No	Non-randomized experimental study	41 healthy older adults (16 dance group - 16 female/0 male, mean age = 73.7; 25 falls prevention training group - 23 female/2 male, mean age = 72.9)	France	Contemporary	4.4 months (average)	1 hour/weekly	Balance	Positive	Significant pre-post improvement in balance performance (superior to falls prevention group) in balance performance (posturography - TechnoConcept platform)	Low
Granacher et al	2012	Other reviews - Liu, Shen & Tsai, 2020; Rodrigues-Krause, Krause & Reischak-Oliveira, 2019										
Hackney et al.	2013	Excluded - investigation of clinical population (visual impairment)										
Holmerova et al	2010	Other review - Liu, Shen & Tsai, 2020 meta-analysis; excluded from further narrative analysis due to clinical population (functional impairment)										
Hui, Chui & Woo	2008	Other reviews - Liu, Shen & Tsai, 2020; Rodrigues-Krause, Krause & Reischak-Oliveira, 2019										
Kim et al	2011	Excluded - Latin dance performed with a target heart rate (exercise, not performing arts, intervention)										
Krampe	2013	Excluded - clinical population (90% of cohort with at least 1 chronic illness)										
Krampe et al	2010	Other review - Rodrigues-Krause, Krause & Reischak-Oliveira, 2019										
Marmeleira et al	2009	No	RCT	37 inactive older adults (19 dance group - 4 male/15 female, mean age = 63.6; 18 control group - 5 male/10 female, mean age = 65.3)	Portugal	Creative dance	12 weeks	90 min/3x weekly	Proprioception	Positive	Significant improvements in arm positioning vs control; significant pre-post improvements in knee proprioception but not vs control (JPS & kinesthesia - knee & upper limb)	High
McKinley et al	2008	Other reviews - Liu, Shen & Tsai, 2020; Rodrigues-Krause, Krause & Reischak-Oliveira, 2019										
Sofianidis et al	2009	Other review - Liu, Shen & Tsai, 2020										
Song et al.	2004	Excluded - Korean dance intervention with a target heart rate (exercise, not performing arts, initiative)										
Wallmann	2008	Other review - Rodrigues-Krause, Krause & Reischak-Oliveira, 2019										
Young, Weeks & Beck	2007	Excluded - dance a small part of a broader fitness program (foot stamping, progressively loaded squats & line dancing)										

Keogh et al., 2009												
Engels et al	1998	Excluded - investigation of aerobic dance performed with target HR of 50-70% maximum with participants instructed to monitor HR throughout (exercise, not performing arts, activity)**										
Eyigor et al	2009	Other reviews - Liu, Shen & Tsai, 2020; Rodrigues-Krause, Krause & Reischak-Oliveira, 2019										
Federici, Bellagamba & Rocchi	2005	Other review - Rodrigues-Krause, Krause & Reischak-Oliveira, 2019										
Hackney, Kantorovich & Earhart	2007	Excluded - investigation of an intervention designed for clinical population (Parkinson's), but delivered jointly to a healthy/clinical cohort										
Holmerova et al	Personal Communi cation	Other review - Liu, Shen & Tsai, 2020 meta-analysis; excluded from further narrative analysis due to clinical population (functional impairment)										
Hopkins et al	1990	Excluded - investigation of aerobic dance performed with target HR of 100-120 beats per minute with participants instructed to monitor HR throughout (exercise, not performing arts, activity)										
Jeon et al	2005	Excluded - Korean language article, very similar intervention to Jeon et al 2000 (exercise, not performing arts)										
Jeon et al	2000	Excluded - Korean dance intervention with a target heart rate (exercise, not performing arts, intervention)										
Kim, June & Song	2003	Excluded - combined analysis of dance within context of 'health promotion program' including health education classes and counseling sessions										
Kudlacek et al	1997	No	Single group experimental study	28 female members of a senior dancing group (mean age 66; 15 osteoporotic, 13 non-osteoporotic)	Austria	Folk, waltz, aerobic	12 months	Unspecified	Bone density (peripheral and spinal)	Mixed (positive, no effect)	Significant pre-post increase in bone density in osteoporotic participants but not in participants with non-clinical bone density (single photon absorptiometry - X-Ray)	Very Low
McKinley et al	2008	Other reviews - Liu, Shen & Tsai, 2020; Rodrigues-Krause, Krause & Reischak-Oliveira, 2019										
Shigematsu et al	2002	Excluded – intensity of dance-based exercise program set at individual‘s lactate threshold (exercise, not performing arts, intervention)										
Sofianidis et al	2009	Other reviews - Liu, Shen & Tsai, 2020; Fong Yan et al., 2018										
Song et al	2004	Excluded - Korean dance intervention with a target heart rate (exercise, not performing arts, initiative)										
Young, Weeks & Beck	2007	Excluded - dance a small part of a broader fitness program (foot stamping, progressively loaded squats & line dancing										
Letton, Thom, & Ward, 2020												

Annino et al.	2007	Excluded - study of whole body vibration in full time professional ballerinas										
Candelario-Gorbea et al	2014	Excluded - study of PNF stretching in students at a ballet school (mean >3 years training)										
da Silva et al	2015	Excluded - study of 17-week ballet training intervention in preprofessional ballet dancers (5 years experience)										
Hedgepeth	1987	No	Cross-sectional (**analysis of the 'pre' data in dancers vs control only; no analysis of intervention results in trained dancers as per exclusion criteria)**	27 female university students (age range 18-35; 18 ballet students (min 2 years experience); 9 sedentary individuals)	USA	Ballet	2+ years ballet experience	Unspecified	Strength	No effect	No significant differences between dancers & control (peak knee extension torque - Cybex)	Low
									Power	No effect	No significant differences between dancers & control ((total work / contraction time) of 30 maximal isokinetic knee extensions - Cybex)	Low
									Endurance (muscular)	No effect	No significant differences between dancers & control ((total work 1st 6 trials / total work last 6 trials) from 30 maximal isokinetic knee extensions - Cybex)	Low
Houston & McGill	2013	Excluded - clinical population (Parkinson's)										
Khan et al.	2000	Excluded - longitudinal study of ballet training in elite ballet dancers										
Kirkendall et al.	1984	Excluded - longitudinal study of ballet training in professional ballet dancers										
Koutedakis & Sharp	2004	Excluded – longitudinal study of dance intervention in professional ballerinas										
Koutedakis, Cross & Sharp	1996	Excluded - longitudinal study of strength training in professional male ballet dancers										
Kujala et al	1997	No	Cross-sectional (**consideration of pre- and post-intervention results in experienced dancers vs	35 female adolescents (mean age = 11.9; 18 ballet dancers, 17 non-athletic controls)	Finland	Ballet	2+ years ballet experience	Mean 7.25 - 11.1 hours weekly training	Flexibility	Positive	Significantly greater hip flexor & hamstring flexibility in dancers vs controls; conclusion that additional lumbar flexibility likely not possible through	Low

			controls as cross-sectional outcomes**)								training (hip flexor, hamstring & lumbar flexibility measured by goniometer)	
Liederbach, Gleim & Nicholas	1992	Excluded - study of ballet training in professional dancers										
Lopez-Ortiz et al	2016	Excluded - clinical population (cerebral palsy)										
Lopez-Ortiz et al	2012	Excluded - clinical population (cerebral palsy)										
McGill, Houston & Lee	2019	Excluded - clinical population (Parkinson's)										
McMillan, Proteau & Lebe	2019	Excluded - study of pilates intervention in elite ballet dancers										
Micheli et al	2005	Excluded - study of ballet training in professional dancers										
Moller & Masharawi	2011	No	Non-randomized experimental study	30 1st-3rd grade girls (age range 6-9; 15 ballet group, 15 control group	Israel	Ballet	6 months	90 min/2x weekly	Flexibility	Positive	Significantly greater hyper flexibility and hip external rotation in dance vs control groups (hip external rotation (goniometer); Beighton score (hyperflexibility)	Low
									Body composition	No effect	No significant differences between groups (BMI)	Low
									Posture	No effect	No significant difference between dance & control groups (thoracic kyphosis; lumbar lordosis)	Low
Notarnicola et al.	2014	Excluded - study of pedagogical methods in experienced ballet dancers (mean 4+ years experience)										
Ozdinc & Turan	2016	No	Cross-sectional	67 young females (mean age ~11; 36 dancers (mean 4+ years dancing experience); 31 non-dancer controls)	Turkey	Ballet	Mean 4.4 years ballet experience	Unspecified	Foot anthropometrics	No effect	No significant differences between dance & control groups on any anthropometric measurement	Low
Pigeon et al	1997	No	Longitudinal cohort study	127 young girls (mean age 12.6)	France	Ballet	5 years	Mean 8.8 hours weekly ballet practice	Growth	Mixed (no effect, negative)	Significantly stunted growth in subgroup of	Very Low

											dancers with comorbid insufficient nutrition	
									Puberty onset	Negative	Later pubertal onset in dancers vs control	Very Low
Scheidler et al.	2018	Excluded - clinical population (multiple sclerosis)										
Stalder, Noble & Wilkinson	1990	Excluded - study of weight training in experienced ballet dancers										
Tsanaka, Manou & Kellis	2017	Excluded - study of pedagogy in college ballet dancers										
Liu, Shen & Tsai, 2020												
Bennett & Hackney	2017	No	RCT	23 adults with mobility limitations (20 female/3 male; 12 intervention, 11 control; aged 65 or over)	USA	Line dance	8 weeks	1 hour/2x weekly	Balance	Positive	Significant improvement in intervention vs control (Berg Balance Scale)	Moderate
									Gait speed	Meta-analysis		Moderate
Cepeda et al	2015	No	RCT	34 female older adults (19 intervention (mean age = 69.1); 15 control (mean age = 71.5))	Brazil	Ballroom	8 weeks	1 hour/3x weekly	Endurance	Meta-analysis		High
									Mobility	Meta-analysis		High
Eyigor et al.	2009	No	RCT	37 female older adults (19 intervention (mean age 73.5); 18 control (mean age = 71.2))	Turkey	Turkish folkloric dance	8 weeks	1 hour/3x weekly	Balance	Positive	Significant improvement in intervention vs control (Berg Balance Scale)	High
									Endurance	Meta-analysis		High
									Mobility	Meta-analysis		High
									General health	Mixed	Significant improvements vs control on a few subscales only (mental health, general health, physical health)(SF-36)	High
Granacher et al.	2012	No	RCT	28 older adults (17 female/11 male; 14 intervention (mean age = 71.6); 14 control (mean age = 68.9))	Germany	Salsa	8 weeks	1 hour/2x weekly	Balance	No effect	No significant difference vs control (center of pressure - dominant leg)	High
									Gait speed	Meta-analysis		High

Hamacher et al.	2015	No	RCT	35 older adults (19 intervention - 11 female, mean age = 67.2; 16 control - 10 female, mean age = 68.5)	Germany	Multiple (Line, Jazz, Square)	6 months	90 min/2x weekly	Gait speed	Meta-analysis		High
Holmerova et al.	2009	No	RCT	52 older adults (27 intervention - 25 female/2 male, mean age = 81; 25 control - 21 female/4 male, mean age = 82.8)	Czech Republic	Ballroom	3 months	75 min/1x weekly	Flexibility	Meta-analysis		High
									Mobility	Meta-analysis		High
Hui, Chui & Woo	2009	No	RCT	97 older adults (52 intervention - 50 female/2 male, mean age = 68; 45 control - 44 female/1 male, mean age = 69.1)	Hong Kong	Low impact aerobic dance	3 months	50 min/2x weekly	Endurance	Meta-analysis		Moderate
									Flexibility	Meta-analysis		Moderate
									Mobility	Meta-analysis		Moderate
									General health	Mixed	Significant improvements vs control on a few subscales only (general health, bodily pain)(SF-36)	Moderate
Janyacharoen et al.	2013	No	RCT	38 female older adults (20 intervention (mean age = 64.9); 18 control (mean age = 66.8))	Thailand	Thai dance	6 weeks	40 min/3x weekly	Endurance	Meta-analysis		High
									Flexibility	Meta-analysis		High
									Mobility	Meta-analysis		High
Machacova et al.	2015	No	RCT	52 older adults (27 intervention - 25 female/2 male, mean age = 81; 25 control - 21 female/4 male, mean age = 82.8)	Czech Republic	Ballroom	3 months	75 min/1x weekly	Flexibility	**same study as Holmerova et al 2009**		High
									Mobility	Meta-analysis		High
McKinley et al.	2008	No	RCT	25 older adults (14 intervention - 11 female/3 male, mean age = 78.1; 11 control - 8 female/3 male, mean age = 74.6)	Canada	Argentine tango dance	10 weeks	2 hours/2x weekly	Mobility	Meta-analysis		High
									Gait speed	Meta-analysis		High
Merom et al.	2016	No	RCT	530 older adults aged at or over 65 (279 intervention - 231 female/38 male; 251	Australia	Folk dance	12 months	1 hour/2x weekly	Gait speed	Meta-analysis		Moderate
									Mobility	Meta-analysis		Moderate

				control - 217 female/34 control)								
Sofianidis et al.	2009	No	RCT	26 older adults (14 intervention - 13 female/1 male, mean age = 69.2; 12 control - 7 female/5 male, mean age = 72.6)	Greece	Greek traditional dance	10 weeks	1 hour/2x weekly	Balance	Positive	Significantly improved balance in intervention group vs control (COP variations)	High
Serrano-Guzman et al.	2016	No	RCT	52 female adults (27 intervention (mean age = 69.1); 25 control (mean age = 69.5))	Spain	Multiple (Flamenco, Sevillanas, ballet)	8 weeks	50 min/3x weekly	Mobility	Meta-analysis		Moderate
						Meng et al., 2020						
Alves	2013	No	RCT	65 healthy older adults (25 dance group - 4 male/21 female, mean age = 69.4; 15 walking group - 15 female, mean age = 66.7; 25 'no contact' control - 1 male/24 female, mean age = 68.4)	Brazil	Ballroom	4 months	2 hours/2x weekly	Executive function	Meta-analysis		High
Chen	2014		RCT	125 older adults (age range 61-82; 65 dance group, 60 control group)	China	Square dancing	48 weeks	30-60 min/4-5x weekly	Global cognition	Meta-analysis		High
Coubard et al	2011	No	Non-randomized experimental study	110 healthy older adults (16 dance group - 16 female/0 male, mean age = 74.7; 67 falls prevention training group - 64 female/3 male, mean age = 74.7; 27 Tai Chi group - 24 female/3 male, mean age = 71.5)	France	Contemporary	5.9 months (mean)	1 hour/weekly	Executive function	Meta-analysis		Low
Doi et al	2017	No	RCT		Japan	Ballroom	40 weeks	1 hour/weekly	Global cognition	Meta-analysis		High

		Clinical population, but included in meta-analysis			201 Japanese adults with mild cognitive impairment (67 dance group - 51% female, mean age = 75.7; 67 music training group - 58% female, mean age = 76.2; 67 health education control group - 46% female, mean age = 76)					Executive function	Meta-analysis	High
		Excluded - clinical population (cognitive impairment)								Memory		
Hackney et al	2015		No	Non-randomized experimental study	74 healthy residents of independent living facilities (62 tango group - 14 male/48 female, mean age = 82.3; 12 health education control group - mean age = 84.1, 7 male/5 female)	USA	Tango	12 weeks	90 min / 20 classes in 12 weeks	Global cognition	Meta-analysis	Very Low
										Executive function	Meta-analysis	Very Low
Kattenstroth et al	2013		No	RCT	35 healthy elderly volunteers (25 dance group - mean age = 68.6, 17 female/8 male; 10 control - mean age = 72.3, 7 female/3 male)	Germany	Agilando (dance program developed for elderly people; no partner necessary)	24 weeks	1 hour/weekly	Global cognition	Meta-analysis	Moderate
Kim et al	2011	Study of Latin dance intervention with target heart rate, but included in meta-analysis	No	Non-randomized experimental study	38 older adults with metabolic syndrome (26 exercise - 7 male/19 female, mean age = 68.2; 12 control - 2 male/10 female, mean age = 68.2)	South Korea	Latin	6 months	1 hour/2x weekly	Global cognition	Meta-analysis	Low
										Executive function	Meta-analysis	Low
		Excluded - Latin dance performed with target heart rate (exercise, not performing arts)								Memory		
Kosmat & Vranic	2017		No	RCT	24 healthy older adults (12 dance - 6	Croatia	Waltz	10 weeks	45 min/weekly	Executive function	Meta-analysis	High

					male/6 female, mean age = 80.1; 12 active control - 9 female/3 male, mean age = 79.1)					Memory	Positive	Significant group*time effect, with significant benefits for memory in dance vs active control (auditory verbal learning test)	High
Lazarou et al	2017	Clinical population, but included in meta-analysis	No	RCT	129 older adults with amnesiatic mild cognitive impairment (66 dance group - 53 female/13 male, mean age = 65.9; 63 control group - 48 female/15 male, mean age = 67.9)	Greece	Ballroom (International)	10 month	1 hour/2x weekly	Global cognition	Meta-analysis		High
										Executive function	Meta-analysis		High
		Excluded - clinical population (cognitive impairment)								Memory			
Merom et al	2016		No	RCT	115 healthy older adults (aged 60+; 60 dance group - 73% female; 55 walk group - 80% female)	Australia	Ballroom	8 months	1 hour/2x weekly	Executive function	Meta-analysis		Moderate
										Memory	No effect	Significant pre-post effect only, no effect vs control (Rey Auditory Verbal Learning Test; Brief Visuospatial Memory Test)	Moderate
Merom et al	2016		No	RCT	530 older adults aged at or over 65 (279 intervention - 231 female/38 male; 251 control - 217 female/34 control)	Australia	Folk dance	12 months	1 hour/2x weekly	Executive function	Meta-analysis		Moderate
Muller et al	2017	Excluded by review authors from meta-analysis - 'insufficient intervention'								Global cognition			
Zhang, Ni & Liu	2012		No	RCT	75 older adults (age range 60-70 years; 39 dance group, 37 control group)	China	Square dancing	48 weeks	30-60 min/4-5x weekly	Global cognition	Meta-analysis		High
Rodrigues-Krause et al., 2016													
Belardinelli et al	2008	Other review - Fong Yan et al., 2018											
Cruz-Ferreira et al	2015	Other review - Rodrigues-Krause, Krause & Reischak-Oliveira, 2019											

Engels et al	1998	Excluded - dance performed with target HR (exercise, not performing arts intervention)										
Hopkins et al	1990	Excluded - dance performed with target HR (exercise, not performing arts)										
Kalsatou et al	2014	Other review - Fong Yan et al., 2018										
Kattenstroth et al	2013	Other review - Teixeira-Machado, Arida & de Jesus Mari, 2019										
Kim et al	2011	Excluded - dance performed with target HR (exercise, not performing arts)										
Rodrigues-Krause, Krause, Reischak-Oliveira, 2019												
Alpert et al	2009	No	Single group experimental study	13 older women enrolled in a senior dance class (mean age = 68)	USA	Jazz	15 weeks	Unspecified	Depression	No effect	No significant pre-post change in depression (Geriatric Depression Scale)	Very Low
									Balance	Positive	Significant pre-post improvement in balance in intervention vs control groups (Sensory Organization Test)	Very Low
									Cognition	No effect	No significant pre-post effect of dance on depression (MMSE)	Very Low
Belardinelli et al.	2008	Excluded - clinical population (heart failure)										
Borges et al	2012	No	RCT	75 sedentary elderly subjects (sex unspecified; 39 dance group (mean age = 68); 36 control (mean age = 67.2))	Brazil	Ballroom	8 months	50 min/3x weekly	Functional autonomy	Positive	Significant increase in functional autonomy in dance group vs control (Latin American Group for Maturity protocol)	High
									Balance	Positive	Significant balance improvement in balance group vs control (postural sway; stabilometer)	High
Chen et al	2013	Included under 'epidemiologic' studies										
Cruz-Ferreira et al	2015	No	RCT	57 older women (32 dance group (mean age = 71.1); 25	Portugal	Creative Dance	24 weeks	50 min/3x weekly	Physical Fitness	Positive	Significantly better fitness improvement in dance group vs control (Senior Fitness Test)	High

				control group (mean age = 72.8))					Life Satisfaction	Positive	Significantly greater improvement in life satisfaction in dance vs control groups (Satisfaction with Life Scale)	High
Duncan et al	2014	Excluded - clinical population (Parkinson's)										
Engels et al	1998	Excluded - investigation of aerobic dance performed with target HR of 50-70% maximum with participants instructed to monitor HR throughout (exercise, not performing arts, activity)										
Eyigor et al.	2009	No	RCT	37 female older adults (19 intervention (mean age 73.5); 18 control (mean age = 71.2))	Turkey	Turkish folkloric dance	8 weeks	1 hour/3x weekly	Depression	No effect	No significant differences in depression in intervention group vs control (GDS)	High
		Other review - Liu, Shen & Tsai, 2020							Balance, Mobility, Endurance, General Health			
Federici et al	2005	No	RCT	40 older adults (14 male/26 female; mean age = 63.1)	Italy	Caribbean dance	3 months	35-60 min/2x weekly (35 min 1st 2 weeks, then 60 min)	Balance	Positive	Significant improvements vs control on all balance tests (Tinetti test, Romberg test, improved Romberg test, Sit-up-and-go test)	High
									Smoking	No effect	(bespoke binary questionnaire)	Moderate
									Alcohol Consumption	No effect	(bespoke binary questionnaire)	Moderate
									Sexual activity	Positive	Significant improvement in sexual activity in experimental vs control groups (bespoke binary questionnaire)	Moderate
									Sleep quality	Positive	Significant improvement in sleep quality in experimental vs control group (bespoke binary questionnaire)	Moderate
Goertzen et al	1984	Excluded - descriptive population survey of physical activity participation										

Granacher et al	2012	Yes	No	RCT	28 older adults (17 female/11 male; 14 intervention (mean age = 71.6); 14 control (mean age = 68.9))	Germany	Salsa	8 weeks	1 hour/2x weekly	Muscular Power (lower leg)	No effect	NO significant group*time effect (maximal countermovement jumps on force platform)	High
		Other review - Liu, Shen & Tsai, 2020								Balance, Gait Speed			
Grant et al	2002	Excluded - descriptive analysis of HR & VO2 responses during aerobic dance (included in HR data)											
Hackney et al	2009	Excluded - clinical population (Parkinson's)											
Hackney et al	2007	Excluded - investigation of an intervention designed for clinical population (Parkinson's), but delivered jointly to a healthy/clinical cohort											
Hackney et al	2013	Excluded - clinical population (visual impairment)											
Hackney et al	2009	Excluded - clinical population (Parkinson's)											
Holmerova et al	2010	Excluded - clinical population (functionally impaired, non-independent)											
Hopkins et al	1990	Excluded - investigation of aerobic dance performed with target HR of 100-120 beats per minute with participants instructed to monitor HR throughout (exercise, not performing arts, activity)											
Hui et al	2009		No	RCT	97 older adults (52 intervention - 50 female/2 male, mean age = 68; 45 control - 44 female/1 male, mean age = 69.1)	Hong Kong	Low impact aerobic dance	3 months	50 min/2x weekly	Strength	Positive	Significant improvement vs control of dance intervention (sit and stand test)	Moderate
										Balance	No effect	No significant effect of dance intervention vs control (4-test balance scale)	Moderate
										Body composition	No effect	No significant effect of dance intervention vs control (BMI & waist to hip ratio)	Moderate
		Other review - Liu, Shen & Tsai, 2020								Endurance, Mobility, Flexibility, Quality of Life			
Janyacharoen et al	2013	Other review - Liu, Shen & Tsai, 2020											
Kalsatou et al	2014	Excluded - clinical population (Chronic Heart Failure)											
Kattenstroth et al	2011		No	Cross-sectional	49 healthy older adults (11 expert competitive dancers – mean age = 71.2, 5	Germany	Ballroom	Mean 22.1 years competitive	Mean 4.6 hours weekly dance	Independent living	Positive	Significantly higher scores in dance vs control group (Everyday	Low

				female/11 male; 38 sedentary subjects – mean age = 71.7, 30 women/8 men)			dance experience				Competence Questionnaire)	
									Cognitive performance	Positive	Significantly better performance in dance vs control (both groups)(Raven's Standard Progressive Matrices; nonverbal geriatric concentration test (AKT))	Low
									Reaction time	Positive	Significantly faster reaction times in experimental vs control groups (multiple choice RT)	Low
									Balance	Positive	Significantly better performance in dance vs control group in Romberg test (eyes open only) and standing turn test (Romberg test; standing turn test))	Low
									Mobility	Positive	Significantly better mobility performance in dance vs control groups (timed up and go)	Low
									Fine motor performance	Positive	Significantly more precise fine motor performance in dance vs control group (computer-based test battery)	Low
									Tactile performance	Positive	Significantly more sensitivity in dance vs control groups (von Frey filaments; 2-point discrimination)	Low
Kattenstroth et al	2013	Other review - Teixeira-Machado, Arida & de Jesus Mari, 2019										
Kattenstroth et al	2010		No	Cross-sectional	62 healthy elderly adults (24 dance group - 19 female/5	Germany	Unspecified ('amateur dancing')	Mean 16.5 years of	Mean 1.3 hours weekly dancing	Independent living	Positive	Significantly higher scores in dance vs control group	Low

	male, mean age = 71.7; 38 control - 30 female/8 male, mean age = 71.7)	amateur dancing			(Everyday Competence Questionnaire)	
			Cognitive performance	Positive	Significantly better performance in dance vs control in general intelligence (RPSM) but not concentration (AKT)(Raven‘s Standard Progressive Matrices; nonverbal geriatric concentration test (AKT))	Low
			Reaction time	Positive	Significantly faster reaction times in dance vs control group(multiple choice RT)	Low
			Balance	Positive	Significantly better performance in dance vs control group in standing turn test; no significant differences in Romberg test (Romberg test; standing turn test))	Low
			Mobility	Positive	Significantly better mobility performance in dance vs control group (timed up and go)	Low
			Fine motor performance	Positive	Significantly more precise fine motor performance in dance vs control group (computer-based test battery)	Low
			Tactile performance	Positive	Significantly better 2-point discrimination in dance vs control group; no significant difference in von Frey touch threshold (von	Low

											Frey filaments; 2-point discrimination)	
Kim et al	2011	Excluded - Latin dance performed with a target heart rate (exercise, not performing arts, intervention)										
Kim et al	2007	Excluded - combined analysis of dance + nutrition education intervention										
Kim et al	2003	Excluded - combined analysis of dance within context of 'health promotion program' including health education classes and counseling sessions										
Krampe	2013	Excluded - clinical population (90% of cohort with at least 1 chronic condition)										
Krampe et al	2014	Excluded - clinical population (chronic pain)										
Krampe et al	2010	No	Single group experimental study	11 elderly adults (7 women/4 men; age unspecified)	USA	Lebed Method	6 weeks	45 min/3x weekly	Balance	No effect	No significant pre-post effect (functional reach test)	Very Low
									Mobility	No effect	No significant pre-post effect (Timed Up & Go)	Very Low
Lesser et al	2016	No	RCT	75 post-menopausal obese south east Asian women (no type 2 diabetes or CVD)(26 dance – mean age = 57.7; 23 standard exercise group – mean age = 56.4; 26 control – mean age =57.7)	Canada	Bhangra	12 weeks	60 min/3x weekly	Body composition	Positive	Significant reduction in abdominal adipose tissue (total & subcutaneous) in dance vs control group; no significant change in visceral adipose tissue or body fat/lean mass (Visceral adipose tissue (tomography scan); lean body mass/fat mass/% body fat (DEXA))	High
									Glucose	No effect	No significant group*time interaction	High
									Insulin	No effect	No significant group*time interaction	High
									Aerobic fitness	Positive	Significant improvement in dance group vs control (VO2 peak)	High
Lim et al	2015	Excluded – Korean dance included as a small portion of a broader exercise program										
Marin et al	2009	Excluded – dance included as part of broader fitness program (1 day/week dance; 2 days/week toning exercise)										
Mavrovouniotis et al	2008	No	Non-randomized	111 healthy older adults (mean age =	Greece		60 min	Once	Anxiety	Positive	(State-Trait Anxiety Inventory)	Low

			experimental study	69.8; 75 female/36 male; 76 dance group, 35 control)		Greek traditional dance			Psychological wellbeing	Positive	(Subjective Exercise Experiences Scale (SEES))	Low
									Psychological distress	Positive	(Subjective Exercise Experiences Scale (SEES))	Low
									Fatigue	Positive	(Subjective Exercise Experiences Scale (SEES))	Low
McKee et al	2013	Excluded - clinical population (Parkinson's)										
McKinley et al	2008	No	RCT	25 older adults (14 intervention - 11 female/3 male, mean age = 78.1; 11 control - 8 female/3 male, mean age = 74.6)	Canada	Argentine tango dance	10 weeks	2 hours/2x weekly	Balance confidence	Positive	Significant improvement in tango vs walk group (Activity specific Balance Confidence Scale (ABC))	High
		Other review - Liu, Shen & Tsai, 2020							Gait, Mobility			
Murrock et al	2014	Excluded - clinical population (depression)										
Park et al	2015	Excluded – Korean dance included as a small portion of a broader exercise program										
Rios Romenets et al	2015	Excluded - clinical population (Parkinson's)										
Shigematsu et al	2002	Excluded – intensity of dance-based exercise program set at individual‘s lactate threshold										
Sofianidis et al	2009	Other review - Liu, Shen & Tsai, 2020										
Song et al	2004	Excluded - Korean dance intervention with a target heart rate (exercise, not performing arts)										
Uusi-Rasi et al	1999	Excluded - combined analysis of Finnish gymnastics and folk dancing										
Verghese	2006	No	Cross-sectional	48 older adults (mean age = 80.4; 16 men/32 women; 24 social dancers/24 non-dancers)	USA	Various ('social' dancing)	Mean 36.5 years social dance experience	Unspecified	General mental status	No effect	No significant differences between dance & control groups (Blessed Information Memory Concentration test)	Low
									Memory (episodic)	No effect	No significant differences between dance & control groups (Free and Cued Selective Reminding Test)	Low

									Executive function	No effect	No significant differences between dance & control groups (Wechsler Adult Intelligence Scale - revised (Digit Span, Digit Symbol, Block Design Tests); Verbal Fluency Test; Trail Making Test	Low
									Depression	No effect	No significant differences between dance & control groups (Geriatric Depression Scale)	Low
									Gait	Positive	Significantly greater gait speed, step/stride length & more favorable balance of % swing & stance phase in social dancers vs control (GaitRITE)	Low
									Balance	Positive	Significantly better performance in standing (physical performance battery, unipedal stance) but not dynamic (chair rise) balance vs control (Physical Performance Battery; unipedal stance; 5x sit to stand)	Low
									Strength	No effect	No significant differences between dance & control groups (grip strength)	Low
Wallmann et al	2009	No	Single group experimental study	12 healthy older women (mean age = 68)	USA	Jazz	15 weeks	90 min/weekly	Balance	Positive	Significant pre-post improvement(Sensory Organization Test)	Very Low
White et al	1984	No	RCT	96 older Caucasian women (age ~56; 36 walking group, 36 aerobic dancing, 24 control)	USA	Aerobic dance	6 months	2-5 dances, 2-4 days/week	Bone Composition	Positive	Significant pre-post increase in bone width & cross-sectional moment of inertia; no significant change in	Low

											bone mineral content (bone mineral content, bone width, cross-sectional moment of inertia of radius)	
									Oestrogen	No effect		Low
									Strength (arm)	Positive	Significant pre-post improvement vs control (isometric – elbow flexion)	Low
Wu et al	2016	No	RCT	32 sedentary females (mean age 59)	Taiwan	Low impact aerobic dance (no 'fitness parameters' (i.e. target HR, intensity))	16 weeks	60 min/3x weekly	Body composition	Positive	Significant reduction in dance vs control group (% body fat – InBody 720)	High
									Cholesterol	Positive	Significant increase in HDL in dance vs control group (no change in LDL or total cholesterol)	High
									Triglycerides	Positive	Significant decrease in triglycerides vs control group	High
									Glucose	No effect	No significant difference between groups	High
									Bone density	No effect	No significant difference between groups (DEXA)	High
									Flexibility	Positive	Significant increases in ankle inversion, eversion and dorsiflexion and knee extension vs control (range of motion – knee, ankle)	High
									Strength	Positive	Significant increase vs control (peak knee extension torque)	High
		Other review - Liu, Shen & Tsai, 2020							Falls, Fear of Falling			
Wu et al	2011	No	Cross-sectional	38 sedentary and active healthy females (12 sedentary	Taiwan	Low impact aerobic dance	At least 3 years aerobic	At least 150 min/week	Body composition	No effect	No significant differences between dance & control	Low

				group (<20 mins weekly PA) - mean age = 62; 26 exercise group - mean age = 59.1)			dance experience				groups (% body fat - InBody 720)	
									Flexibility	Positive	Significantly greater ankle ROM in dance vs control group, no significant differences in knee ROM (range of motion of knee & ankle joints - goniometer)	Low
									Bone density	Positive	Significantly greater bone mineral density T-score in dance vs control group (GE bone ultrasonometer)	Low
									Strength	Positive	Significantly greater knee extension torque in dance vs control group (knee extension torque)	Low
Young et al	2007	Excluded - dance a small part of a broader fitness program (foot stamping, progressively loaded squats & line dancing)										
Zhang et al	2008	No	Cross-sectional	404 older adults (mean age = 61.4; mixed sex - exact breakdown unspecified)	China	Various ('social' dancing)	Mean 6.1 years dancing	Mean 4.8 weekly dancing sessions	Gait speed	Mixed	Significantly faster gait speed vs control in males only (analysis adjusted for BMI, years of education, hypertension & diabetes)	Low
									Flexibility	Mixed	Significantly increased flexibility in younger (50-59 year old) & male subjects vs. control only ((analysis adjusted for BMI, years of education, hypertension & diabetes)(sit & reach test)	Low
									Reaction time	Positive	Significantly improved reaction time vs control (analysis adjusted for BMI, years of education,	Low

												hypertension & diabetes)(leg - Motor Choice Reaction Test)	
										Balance	Mixed	Significantly improved balance control in older cohort only (analysis adjusted for BMI, years of education, hypertension & diabetes)(postural sway)	Low
Teixeira-Machado, Arida & de Jesus Mari, 2019													
Baniqued et al.	2018		No	RCT	188 older adults (127 female/61 male; mean age = ~65; minimum Mini-Mental State Examination (MMSE) score = 27; 48 dance group, 42 walk group, 44 walk+ beta-alanine group, 54 stretch-strength-stability)	USA	'Social' dance sequences (i.e. Contra & English country dancing)	6 months	3x/week	VO2 max	No effect	No significant pre-post difference in cardiorespiratory fitness in dance group; group*time interaction - walk & walk+ group generated significantly greater fitness gains	High
										Executive function	No effect	No significant effect of dance intervention on executive function; group*time interaction demonstrating significant advantages of walk, walk+ & SSS groups vs. dance in executive function improvement(Virginia Cognitive Aging Project fluid reasoning tasks + Switching Task & Spatial Working Memory Task (principal components analysis run to verify relations between tests))	High
										Perceptual speed	No effect	No significant effects of any intervention on perceptual speed	High

												(Virginia Cognitive Aging Project perceptual speed category)	
										Episodic memory	No effect	No significant effects of any intervention on episodic memory(Virginia Cognitive Aging Project episodic memory category)	High
										Vocabulary	No effect	No significant effects of any intervention on vocabulary (Virginia Cognitive Aging Project vocabulary category)	High
										Fluid intelligence	No effect	No significant difference between groups (Virginia Cognitive Aging Project)	Low
										Perceptual speed	No effect	No significant difference between groups (Virginia Cognitive Aging Project)	Low
Burzynska et al.	2017		No	Cross-sectional	40 female university students (mean age = 21.5; 20 dancers, 20 non-dancers)	USA	Unspecified	Mean 12 years dance practice	Mean 14 hours/week	Spatial/relationa l memory	No effect	No significant difference between groups (Virginia Cognitive Aging Project)	Low
										Balance	Positive	significantly better balance in dancers vs non-dancers (eyes closed, 1-legged stance on foam block with dominant leg)	Low
										Cognitive structure & function	Positive	Significantly different brain structure & function in dancers vs non-dancers (fMRI)	Low
Doi et al.	2017	Excluded - study of clinical population (mild cognitive impairment)											

Ehlers et al.	2017		No	RCT	247 older adults (169 female/78 male; mean age = ~65; minimum Mini-Mental State Examination (MMSE) score = 23; 69 dance group, 54 walk group, 54 walk+ beta-alanine group, 70 stretch-strength-stability)	USA	'Social' dance sequences (i.e. Contra & English country dancing)	6 months	3x/week	Loneliness	Positive	Significant reduction in pre-post loneliness across all 4 groups(UCLA Loneliness Scale)	Moderate
										Social Support	Positive	Significant improvements in social support across all 4 groups (no inter-group differences)(Social Provisions Scale)	Moderate
										Stress	Positive	Significant pre-post reduction in stress across all 4 groups (no inter-group differences)(Perceived Stress Scale)	Moderate
Kattenstroth et al.	2013		No	RCT	35 healthy elderly volunteers (25 dance group - mean age = 68.6, 17 female/8 male; 10 control - mean age = 72.3, 7 female/3 male)	Germany	Agilando (dance program developed for elderly people; no partner necessary)	24 weeks	1 hour/weekly	Lifestyle/quality of life	Positive	Significant pre-post improvement in dance group but not control (Everyday Competence Questionnaire; Fragebogen zur Lebenszufriedenheit (FLZ))	Low
										Attention	Positive	Significant pre-post improvement in dance group but not control group (non-verbal geriatric concentration test (AKT); Frankfurt Attention Inventory (FAIR))	Low
										Intelligence (nonverbal)	No effect	No pre-post effect of dance on nonverbal intelligence (Non-verbal learning test (NVLT))	Low
										Fluid intelligence	No effect	No significant pre-post differences in either dance or control groups (Raven	Low

											Standard Progressive Matrices (RSPM))	
									Reaction time	Positive	Significant pre-post improvement in dance group but not control group	Low
									Balance	Positive	Significant pre-post improvement in dance group but not control group	Low
									Fine-motor performance	Positive	Significant pre-post improvement in dance group but not control group (MLS)	Low
									Tactile performance	Positive	Significant pre-post improvement in dance group but not control group (touch threshold (von Frey filaments), 2-point discrimination threshold, haptic object recognition)	Low
									Cardiorespiratory fitness	No effect	No significant pre-post differences in dance or control groups (VO2 peak)	Low
		Other review (Meng et al., 2020)							Global cognition (RBANS)			
Muller et al.	2017	No	RCT	52 healthy older adults (Baseline sex breakdown unspecified; mean age = ~68, 26 dance/26 sport; final analyses completed on only 22 subjects (10 female/12 male; 12 dance, 10 sport))	Germany	Multiple (line, jazz, rock'n'roll, square dance)	18 months	90 min/twice weekly (1st 6 months); 90 min/weekly (last 12 months)	Cardiovascular fitness	No effect	No significant effect of group or time on physical fitness (Physical Working Capacity 130)	Moderate
									Memory	Positive	Significant effect of time across both groups; No significant group*time effect (Rey Auditory Verbal Learning Test)	Low
									Attention	Positive	Significant effect of time across both groups; No significant group*time interaction	Low

											(Test of Attentional Performance (TAP))	
									BDNF	Positive	Significant increase in BDNF vs control in dance group at 6-month timepoint (sustained to 18 month timepoint)	Moderate
									Cognitive structure	Positive	Significant increase in grey matter in dancers vs control	Moderate
Rehfeld et al.	2018	Excluded - same study as Muller et al., 2017										
Rehfeld et al.	2017	No	RCT	52 healthy older adults (Baseline sex breakdown unspecified; mean age = ~68, 26 dance/26 sport; final analyses completed on only 22 subjects (10 female/12 male; 12 dance, 10 sport))	Germany	Multiple (line, jazz, rock'n'roll, square dance)	18 months	90 min/twice weekly (1st 6 months); 90 min/weekly (last 12 months)	Balance	Positive	Significant group*time effect favoring the dance group (Sensory Organization Test - Balance Master System)	Moderate
									Hippocampal grey matter volume	Positive	Significant increases in hippocampal grey matter volume in right hippocampus in dance group only (MRI)	Moderate
Veronese et al., 2017												
da Silva Borges	2014	No	RCT	59 elderly healthy retirement home residents (sex unspecified; dance group - mean age = 68; control group - mean age = 67)	Brazil	Ballroom	12 weeks	50 min/3x weekly	Falls	Positive	Significant reduction Significant reduction in falls in dance vs control groups (interviews confirmed with medical records)	High
Merom et al.	2016	No	RCT	530 older adults aged at or over 65 (279 intervention - 231 female/38 male; 251 control - 217 female/34 male)	Australia	Folk dance	12 months	1 hour/2x weekly	Falls	No effect	(diaries)	High
									Falls risk	No effect	(PPA)	High
Pichierri, Murer & de Bruin	2012	No	RCT	31 retirement home residents (mixed sex - sex breakdown only available for analyzed	Switzerland	Video game dance	12 weeks	10-15 minutes/2x weekly (on top of weekly exercise/	Fear of falling	No effect	No significant differences between dance & control groups (Falls efficacy scale international)	Moderate

				participants; mean age = 86.2)				balance training program)				
Ventura et al	2016	Excluded - clinical population (Parkinson's)										
Volpe et al	2013	Excluded - clinical population (Parkinson's)										
Wu et al.	2016	No	RCT	32 healthy female older people (16 dance/16 control; mean age = 59)	Taiwan	Low impact aerobic dance (no 'fitness parameters' (i.e. target HR, intensity))	16 weeks	60 min/3x weekly	Fear of falling	Positive	Significantly improved fear of falling in dance group vs control (Modified Falls Efficacy Scale)	High
									Falls	NO effect	No significant differences in falls in dance vs control group (self-report)	High

Table S8. Details of individual studies in included reviews of music participation.

Author	*Year*	*In other review / exclusion?*	*Industry support?*	*Design*	*Subjects*	*Study country*	*Type of music participation*	*Length of intervention*	*Frequency/duration of each session*	*Outcome*	*Effect of music participation*	*Outcome details*	*GRADE Certainty of Evidence*
Butzlaff, 2000													
College Board	1988-1998 (not 1993)		No	Cross-sectional	5,732,282 high school seniors, mixed sex	USA	Instrumental or vocal music class	At least 1 course	Not specified	Reading ability (correlation)	Meta-analysis		Moderate
Douglas & Willats	1994		No	Cross-sectional	78 4th graders (mean age 8), 40 female/38 male	UK	N/A (correlational analysis of rhythm, pitch, reading & spelling)			Reading ability (experimental)	Meta-analysis		Low
Engdahl	1994		No	Retrospective cohort study	598 elementary school students (enrolled as 3rd graders), sex unspecified - mixed	USA	Instrumental music education (orchestra & band)	3 years	At least 90 minutes, weekly (school weeks)	Reading ability (correlation)	Meta-analysis		Low
Fetzer	1994		No	Longitudinal cohort study	30 children (unspecified age), sex unspecified	USA	Singing	5-6 months	Unspecified	Reading ability (experimental)	Meta-analysis		Low
Friedman	1959		No	Longitudinal cohort study	254 5th & 6th graders, sex unspecified - mixed	USA	Instrumental music education	8 months	7-8 hours, weekly	Reading ability (correlation)	Meta-analysis		Low
Groff	1963		No	Longitudinal cohort study	460 6th graders (278 boys, 182 girls)	USA	Instrumental music education	Unclear	30 min, 1-2x/week	Reading ability (correlation)	Meta-analysis		Low
Kelly	1981		No	RCT	42 1st graders (mean age 6.6), sex unspecified - mixed	USA	Orff-Schulwerk music education	6 months	30 min, 3x/week	Reading ability (experimental)	Meta-analysis		Moderate
Kvet	1985		No	Retrospective cohort study	175 6th graders, sex unspecified	USA	Instrumental music education	8 months	70 min, weekly	Reading ability (correlation)	Meta-analysis		Low
Lamar	1989		No	Cross-sectional	140 students (70 1st graders; 70 4th graders), sex unspecified	USA	'Music education'	Musical experience unclear	30 min, weekly	Reading ability (correlation)	Meta-analysis		Low

McCarthy	1992		No	Unclear (correlational)	957 college music majors, age & sex unavailable	USA	Orchestra	At least 2 years college music instruction	Unspecified	Reading ability (correlation)	Meta-analysis		Low
Olanoff & Kirschener	1969		No	Unclear (experimental)	46 students, age & sex unavailable	USA	Music training	Unclear	Unspecified	Reading ability (experimental)	Meta-analysis		Low
Roberts	1978		No	Unclear (experimental)	33 students, age & sex unavailable	USA	'Note reading, keyboard instrument'	Unclear	Unspecified	Reading ability (experimental)	Meta-analysis		Low
Roskam	1979		No	Non-randomized experimental cohort study	24 learning disabled children (age range 6-9), sex unspecified	USA	Music therapy/educatio n	3 months	1 hour, 2x/week	Reading ability (experimental)	Meta-analysis		Low
Weeden	1971		No	Non-randomized experimental cohort study	47 1st & 2nd graders, sex unspecified	USA	Suzuki violin education	4 months	2x/week	Reading ability (correlation)	Meta-analysis		Low
Clift et al, 2010 *(**Tables 1 & 2 in review analyzed only; Tables 3 & 4 excluded (clinical populations)**)*													
Beck et al	2000			Single group experimental study (pre-post analysis)	42 professional singers (mean age 46.4; mean singing experience 36.6 years; 23 female, 18 male)	USA	Singing	1.5 (performance) – 2.5 (rehearsal) hours	Once (3 pre-post tests; 2 rehearsal, 1 performance)	Immunoglobulin A	Positive	Significant pre-post increase in IgA after both rehearsals and performance (enhanced immune activity)	Very Low
										Cortisol	Mixed	Significant decrease in cortisol after rehearsals; significant increase in cortisol after performances	Very Low
Clift et al	2009	Excluded - qualitative outcomes only											
Clift et al	2010	Excluded - descriptive, uncontrolled survey-based cross sectional study											
Clift et al	2010	Excluded - descriptive, uncontrolled survey-based cross sectional study											
Cohen	2009	Other review - Daykin et al., 2018											
Cohen et al	2007		No	Nonrandomized experimental study	128 English speaking healthy, ambulatory adults older than 64 (mean age = ~79; 60 control group; 68 intervention group), 78-80% female	USA	Singing	2 periods of 30 weeks over 2 years	Once per week (duration unspecified)	Overall health	No effect	No significant group*time interaction (Self-report - bespoke instrument)	Very Low
										Health system utilization	Positive	Intervention significantly reduced number of doctor visits in 24 study months in	Very Low

											intervention vs control group (group*time interaction)(Self-report - bespoke instrument)	
									Medication usage (over the counter + prescription)	Positive	Intervention significantly impacted reduced usage of over the counter and prescription medication in intervention vs control group (Self-report - bespoke instrument)	Very Low
									Falls incidence	No effect	No significant group*time effect (Self-report - bespoke instrument)	Very Low
									Morale	No effect	No significant group*time interaction (Philadelphia Geriatric Center Morale Scale)	Very Low
									Depression	No effect	NO significant group*time interaction (Geriatric Depression Scale - Short Form)	Very Low
									Loneliness	No effect	No significant group*time interaction (Loneliness Scale - III)	Very Low
									Social activity level	Positive	Significantly greater participation in weekly and yearly social activities over time (group*time interaction) in intervention vs control group (Self-report - bespoke instrument)	Very Low
Cohen et al	2007	Excluded - descriptive, uncontrolled survey-based cross sectional study										
Cohen et al	2006	Other review - Daykin et al., 2018										
Hillman	2002	Excluded - descriptive, uncontrolled survey-based cross sectional study										
Houston et al	1998	No	Non randomized experimental cohort study	61 residents of retirement homes	UK	Singing	4 weeks	1 hour	Anxiety	Positive	Significantly decreased anxiety vs control in singing group (General	Low

				(mean age 83.7, 44 female, 17 male)							Health Questionnaire (GHQ) & Hospital Anxiety & Depression Scale (HADS))	
									Somatic symptoms	No effect	No effect of singing intervention on somatic symptoms (General Health Questionnaire (GHQ))	Low
									Depression	Mixed	Significantly decreased depression vs control (Hospital Anxiety and Depression Scale (HADS)); no change in severe depression (General Health Questionnaire (GHQ))	Low
									Social functioning	No effect	No effect of singing on social functioning (General Health Questionnaire (GHQ))	Low
Kreutz et al	2004	Other review - Fancourt, Ockelford & Belai, 2014										
Kuhn	2002	Other review - Fancourt, Ockelford & Belai, 2014										
Lally	2009	Excluded – qualitative outcomes only										
Latimer	2008	Excluded - descriptive, uncontrolled survey-based cross sectional study										
Louhivuori, Salminen & Lebaka	2005	Excluded - descriptive, uncontrolled survey-based cross sectional study focused on motivations for singing rather than health benefits										
Sandgren	2009	No	Single group experimental study (pre-post analysis)	212 amateur/advanced choir members (mean age ~53; 152 women, 60 men)	Finland	Singing	1 rehearsal (duration unspecified)	Once	Positive affect	Positive	Significantly increased feelings of alertness, happiness & pride following choral rehearsal (bespoke tool completed by the authors)	Very Low
Schorr-Lesnick et al	1985	No	Cross-sectional	113 professional musicians (31 string & percussion (mean age 43.5); 48 wind/brass (mean age 47.4); 34 vocalists (mean age	USA	Singing; Wind Instrumentalists	Mean years performing range 18.6-27.5 across groups	Unspecified	Lung function	No effect	No significant differences between percussion/strings, wind/brass or vocalists on any lung function measure (maximum voluntary ventilation; forced expiratory	Low

				41.3)), 94 male/19 female							volume; forced vital capacity; mean forced expiratory flow during the middle half of forced vital capacity; ratio forced expiratory volume:forced vital capacity; peak expiratory pressure; peak inspiratory pressure)	
Steurer et al	1998	No	Cross-sectional	89 adults (62 professional singers – 30 women (age range 34 – 58), 32 men (age range 39 – 61); 27 noise exposed workers (11 female, 16 male; all under 30 years old without signs of hearing impairment)	Austria	Singing	Mean 24 (male) – 27 (female) years performing experience	Mean weekly singing of 15 (male) – 29 (female) hours	Hearing threshold	Negative	Significantly impaired hearing thresholds in professional singers vs control	Very Low
Unwin, Kenny & Davis	2002	No	RCT	107 adult volunteers with varied singing experience (age range 18 – 73; 23 male, 84 female)	Australia	Singing	30 minutes	Once	Mood	No effect	No significant effect of singing on mood vs active control (music listening)(POMS)	Moderate
Valentine & Evans	2001	Other review - Daykin et al., 2018										
Wise, Hartmann & Fisher	1992	No	Cross-sectional	98 retirees (49 singing in a retirement village community chorus; 49 not in choir; mean age 64.6), sex unspecified - mixed	USA	Singing	Unspecified (chorus members generally with robust musical histories)	Unspecified	Life satisfaction	No effect	No significant differences between choral & comparison groups (Life Satisfaction Index A)	Very Low
									Self-actualization	Unclear	Significantly lower self-actualization in choral vs comparison group, however this is hypothesized to be a surrogate for enhanced social quality of life (healthy dependency) (Personal Orientation Inventory)	Very Low

									Social isolation (alienation)	No effect	No significant differences between choral & comparison groups (Middleton's Alienation Scale)	Very Low
Coffey, Mogilever & Zatorre, 2017												
Baskent & Etienne	2016	No	Cross-sectional	38 adults (mean age 22.2; 24 females, 14 males; 20 musicians, 18 nonmusicians)	Netherlands	Music training	At least 10 years of musical training, beginning before age 7	Unspecified	Speech in speech	Positive	Significantly improved speech in speech perception in musicians vs nonmusicians (protocol as per Versfeld et al 2000)	Low
Bidelman & Weiss	2014	No	Cross-sectional	24 adults (mean age 24.3; 12 musicians, 12 nonmusicians), 16 female/8 male	Canada	Instrumental	Average 13.6 musical training	Unspecified	Auditory processing	Positive	Significantly faster classification of speech and enhanced nervous system responsiveness to speech in musicians vs nonmusicians	Low
Boebinger et al.	2015	No	Cross-sectional	50 adults (mean age 27.2; 25 musicians, 25 nonmusicians), sex unspecified	UK	Instrumental	Average 22.7 years of musical training	Practice at least 3x/weekly over the 3 years before testing	Speech in noise (Masked speech perception)	No effect	No significant differences between musicians and nonmusicians (Bamford-Kowal-Bench lists)	Very low
									Duration discrimination	No effect	No significant differences between musician and nonmusician groups (duration discrimination thresholds test - Grassi & Soranzo adaptive staircase procedure)	Low
									Pitch discrimination	Positive	Significantly better pitch discrimination performance in musicians vs nonmusicians (frequency discrimination thresholds test - Grassi & Soranzo adaptive staircase procedure)	Low

									IQ (nonverbal)	No effect	No significant differences between musicians & nonmusicians (Matrix Reasoning subtest of Wechsler Abbreviated Scale of Intelligence (WASI))	Low
									Selective attention	No effect	No significant differences between musicians & nonmusicians (Stroop task)	Low
									Mental flexibility (Motor speed)	No effect	No significant differences between musicians & nonmusicians (Trail making test)	Low
									Mental flexibility (Fluid intelligence)	No effect	No significant differences between musicians & nonmusicians (Trail making test)	Low
									Mental flexibility (Task switching)	No effect	No significant differences between musicians & nonmusicians (Trail making test)	Low
		Other review - Talamini et al., 2017							Short-term memory			
		Other review - Talamini et al., 2017							Working memory			
Clayton et al	2016	No	Cross-sectional	34 adults (mean age 23.5; 17 musicians, 17 nonmusicians), sex unspecified	USA	Instrumental	Average 14.4 years of music training	Unclear	IQ (nonverbal)	No effect	No significant differences between musicians & nonmusicians (Wechsler Abbreviated Scale of Intelligence - matrix reasoning)	Low

									Executive function (Inhibition control)	No effect	No significant differences between musicians & nonmusicians (color-word interference sub-test of Delis-Kaplan Executive Function System (DKEFS))	Low
									Executive function (Cognitive flexibility/goal directed behavior)	No effect	No significant differences between musicians & nonmusicians (design fluency sub-test of the DKEFS)	Low
									Speech in speech	Positive	Significantly better speech in speech perception in musicians vs nonmusicians(as per Swaminathan et al. 2015)	Low
									Selective attention (Multiple object tracking)	No effect	No significant differences between musicians & nonmusicians (as per Pylyshyn & Storm, 1988; Drew & Vogel , 2008)	Low
		Other review - Talamini et al., 2017							Working memory			
Coffey et al	2016	Excluded - no analysis of the impact of musical training/participation on outcomes										
Coffey et al	2016	No	Cross-sectional	20 adults (mean age 25.7), 12 female/8 male	Canada	Unspecified	N/A (correlational analysis with musical experience as variable)	N/A	Pitch discrimination	Positive	Positive correlation between hours of musical training and enhanced fine pitch discrimination ability	Very Low
									Auditory processing	Positive	Positive correlation between hours of musical training and enhanced P2 amplitude	Very Low
Du & Zatorre	2016	No	Cross-sectional	30 healthy adults (mean age ~21), sex unspecified	USA	Unspecified (conference abstract)	Unspecified	Unspecified	Speech in noise	Positive	Significantly better speech in noise perception in musicians vs nonmusicians	Low

Fuller et al.	2014	No	Cross-sectional	50 adults (mean age 22.7); 25 musicians, 25 nonmusicians; 14 male/36 female	Netherlands	Instrumental	Average 14.6 years musical training	Unspecified ('regular musical training within the last 3 years')	Speech in noise	Positive	Significantly better speech in noise processing in musicians vs nonmusicians	Low
									Vocal emotion identification	Positive	Significantly better vocal emotion identification in musicians vs nonmusicians	Low
									Melodic contour identification	Positive	Significantly better melodic contour identification in musicians vs nonmusicians	Low
Lee et al.	2009	No	Cross-sectional	26 adults (10 musicians (mean age 25.8), 11 nonmusicians (mean age 23.5), 5 amateur musicians (mean age 23.2)), 16 female/10 male	USA	Instrumental/ vocal	At least 10 years musical experience beginning before age 7	Unspecified	Auditory processing	Positive	Significantly more precise neural phase locking to the temporal periodicity of the amplitude modulated envelope (underlies perception of harmony) in musicians vs nonmusicians; significantly better perception of upper harmonic voice (behavioral adaptation) in musicians vs nonmusicians; neural enhancements strongly correlated with years of musical training	Low
Musacchia et al.	2007	No	Cross-sectional	29 adults (mean age 25.6; 14 female, 15 male); 16 musicians, 13 controls	USA	Instrumental	At least 10 years musical experience, begun before age 10	At least 4 hours/week	Auditory processing	Positive	Significantly larger brainstem responses to both speech and music stimuli in auditory and audiovisual conditions in musicians vs nonmusicians; enhanced phase-locking to stimulus periodicity (underlies perception of pitch) in musicians vs nonmusicians	Low

										(correlated with increasing number of musical practice hours)	
Musacchia, Strait & Kraus	2008	No	Cross-sectional	26 adults (mean age 25.6; 14 female); 14 musicians, 12 nonmusicians	USA	Instrumental	10 or more years musical experience, started at or before age 5	>4 hours weekly practice	Auditory processing (neural timing)	Positive	Significantly faster cortical and subcortical responses to auditory stimuli in musicians vs nonmusicians	Low
Oxenham et al.	2003	No	Cross-sectional	24 adults (mean age 24.7); 12 musicians, 12 nonmusicians; sex unspecified	USA	Instrumental	Unspecified	Unspecified	Sound in noise	Positive	Significantly lower threshold values for detecting sound masked in noise in musicians vs nonmusicians	Low
									Speech in noise (Frequency selectivity)	No effect	No significant differences in frequency selectivity in musicians vs nonmusicians (notched-noise method)	Low
Parbery-Clark et al.	2009	No	Cross-sectional	31 right handed English speaking adults (mean age 23; 16 musicians, 15 nonmusicians), 19 female/12 male	USA	Piano & violin	Average 16 years music training	Practice at least 3x/weekly over the 3 years before testing	Speech in Noise	Positive	Significantly better performance in musicians vs non-musicians (Quick Speech in Noise (QuickSIN) & Hearing in Noise Test (HINT))	Low
									Working memory	Positive	Significantly better performance in musicians vs non-musicians(Woodcock-Johnson, numbers reversed & auditory working memory tests)	Low
									Pitch discrimination	Positive	Significantly better performance in musicians vs non-musicians (adaptive staircase method as per Levitt 1971)	Low
Parbery-Clark et al.	2011	No	Cross-sectional	37 adults (mean age 50; 18 musicians, 19 nonmusicians); age range 45-65, sex unspecified	USA	Piano & violin	Average 50 years music training	Practice at least 3x/weekly over the 3	Speech in noise	Positive	Significantly better speech in noise perception in musicians vs nonmusicians (Hearing in Noise Test	Low

								years before testing			(HINT) + Quick Speech in noise test (QuickSIN) + Words in Noise Test (WIN))	
									Auditory processing (temporal acuity)	Positive	Significantly better auditory temporal acuity in musicians vs nonmusicians (Backward masking subtest from IHR Multi-center Battery for Auditory Processing)	Low
		Other review - Talmini et al., 2017							Working memory			
Parbery-Clark et al.	2011	No	Cross-sectional	31 young adults (mean age 22.4; 16 musicians, 15 nonmusicians), 22 female/9 male	USA	Instrumental	Average 16.4 years musical participation, begun at or before age 7	Undefined	Speech in noise	Positive	Significantly better speech in noise perception in musicians vs nonmusicians (Hearing in Noise Test (HINT)	Low
									IQ (nonverbal)	No effect	No significant differences between musicians and nonmusicians (Test of Nonverbal Intelligence (TONI))	Low
Parbery-Clark et al.	2012	No	Cross-sectional	48 middle-aged adults (mean age 56;23 musicians, 25 non-musicians), sex breakdown unspecified - mixed	USA	Instrumental (predominantly piano & violin)	Average 49 years musical experience	Practice at least 3x/weekly	Speech in noise	Positive	Significantly improved speech in noise perception in musicians vs nonmusicians (HINT; Speech subscale of Speech, Spatial and Qualities Questionnaire (self-report); more robust neural encoding of speech in both quiet and noise (neurophysiology)	Low
									IQ (nonverbal)	No effect	No significant differences between musicians and nonmusicians (Abbreviated Wechsler's Adult Scale	Low

											of Intelligence matrix reasoning subtest)	
Parbery-Clark et al.	2012	No	Cross-sectional	87 adults (50 younger - mean age 23; 37 older - mean age 56); 46 musicians, 41 nonmusicians; 62 female/25 male	USA	Unspecified	Consistent musical training started before age 9	Engaged in musical activities a minimum of 3 times weekly	Auditory processing	Positive	Musical experience significantly counteracts age-related delays in subcortical response timing to formant transition	Low
Parbery-Clark, Skoe & Kraus	2009	No	Cross-sectional	31 adults (mean age 23; 10 females); 16 musicians, 15 nonmusicians	USA	Instrumental	Practiced consistently for at least 10 years prior to study enrollment	Unspecified	Speech in noise	Positive	Significantly improved speech in noise perception in musicians vs nonmusicians (HINT; more robust subcortical representation of the acoustic stimulus in the presence of noise (neurophysiology))	Low
Ruggles et al.	2014	No	Cross-sectional	33 university students (mean age 21.2; 18 female, 15 male); 16 participants with at least 10 years formal music training	USA	Instrumental	At least 10 years of musical training, beginning before age 10	Currently playing at least 5 hours/week	IQ	Positive	Significantly higher IQ (Wechsler Abbreviated Scale of Intelligence, 2nd edition) in musicians vs nonmusicians	Low
									Speech in noise (Voiced speech discrimination)	No effect	No significant differences between musicians vs non-musicians in voiced speech discrimination (as per Helfer & Freyman)	Low
									Speech in noise (Whispered speech discrimination)	No effect	No significant differences between musicians vs non-musicians in whispered speech discrimination (as per Helfer & Freyman)	Low

Ruggles et al.	2014	No	Cross-sectional	26 university students (unspecified age/sex); 14 participants with at least 10 years formal music training	USA	Instrumental	At least 10 years of musical training, beginning before age 10	Currently playing at least 5 hours/week	Pitch discrimination	Positive	Significantly better pitch discrimination in musicians vs nonmusicians (unclear if this is a validated assessment)	Low
									Speech in Noise	No effect	No significant differences between musicians and non-musicians on clinical speech in noise assessments (QuickSIN; Adaptive HINT)	Low
Slater & Kraus	2016	No	Cross-sectional	38 adult males (mean age 23.3; 21 vocalists, 17 nonmusicians)	USA	Vocal	Average 14.7 years musical experience	Undefined	Speech in noise	No effect	No significant differences between vocalists and nonmusicians in speech in noise perception (QuickSIN)	Low
									Speech in noise	Positive	Significantly better speech in noise perception in percussionists vs nonmusicians (QuickSIN)	Low
									Words in noise	No effect	No significant differences between musicians and nonmusicians in words in noise perception (Words in noise test as per Wilson et al. 2007)	Low
									IQ (nonverbal)	No effect	No significant differences between musicians and nonmusicians in nonverbal IQ (Test of Nonverbal Intelligence (TONI))	Low
Slater et al.	2015	No	RCT	38 elementary school children from disadvantaged backgrounds (mean age 8.2; 17 males,	USA	General music education + (after initial competency demonstrated in	1 year	1 hour, twice weekly	Speech in Noise	Positive	Significantly improved speech in noise perception in music group vs control group (Hearing in Noise Test);	Low

				21 females; 19 music training group; 19 control)		general classes (up to 1 year)) instrumental music lessons					**significant group*time main effect, but main significant difference at 2-year timepoint (music group with 2 years training; control group with 1 year music training)	
Strait & Kraus	2011	No	Cross-sectional	23 adults (age range 18-35), sex unspecified	USA	Instrumental	Average 16.5 years musical experience	Engaged in musical activities a minimum of 3 times weekly	Auditory processing	Positive	Significantly greater impact of selective auditory attention on prefrontal evoked activity	Low
Strait et al.	2010	No	Cross-sectional	33 adults (age range 18-40; 18 musicians, 15 nonmusicians), sex unspecified	USA	Unspecified	At least 10 years musical experience beginning before age 9	At least 3 hours/week	Auditory working memory	No effect	No significant differences between musicians and nonmusicians (reverse digit task)	Low
									Auditory processing (attention)	Positive	Significantly better in musicians vs nonmusicians ('similar to the' Test of Attentional Performance)	Low
									Visual attention	No effect	No significant differences between musicians and nonmusicians ('similar to the' Test of Attentional Performance)	Low
									Frequency discrimination	Positive	Significantly better in musicians vs nonmusicians (3 down, 1 up procedure as per Amitay et al. 2006)	Low
									Speech in noise (backward masking)	Positive	Significantly better in musicians vs nonmusicians (3 down, 1 up procedure as per Amitay et al. 2006)	Low

									Speech in noise (forward masking)	No effect	No significant differences between musicians and nonmusicians (3 down, 1 up procedure as per Amitay et al. 2006)	Low
									IQ (nonverbal)	No effect	No significant differences between musicians and nonmusicians (Wechsler Abbreviated Scale of Intelligence matrix reasoning subtest (WASI))	Low
Swaminathan et al.	2015	No	Cross-sectional	24 adults (mean age 21.7 years - 12 musicians, 12 nonmusicians), sex unspecified	USA	Instrumental	At least 10 years music training	Undefined - 'most practiced at least 5 hours per week'	Speech in noise	Positive	Significantly better speech in noise perception in musicians vs. nonmusicians when masking speech panned 15-degress to the right and left; no significant improvement in speech in noise perception when target and masking speech are co-located (procedure as per Kidd et al. 2010)	Very low
Tierney, Krizman & Kraus	2015	No	Nonrandomized longitudinal cohort study	68 high schoolers (mean age 14.7), sex unspecified	USA	Instrumental	3 years	2.33-3 hours/week	Language skills (Phonological awareness)	Positive	Significantly greater gains in phonological awareness in music vs control group (Comprehensive Test of Phonological Processing)	Very Low
									Language skills (Phonological memory)	No effect	No significant differences in music vs control group in changes in phonological memory (Comprehensive Test of Phonological Processing)	Very Low

									Language skills (Rapid naming)	No effect	No significant differences in music vs control group in changes in rapid naming (Comprehensive Test of Phonological Processing)	Very Low
									Auditory processing (Subcortical response consistency)	Positive	Significantly delayed waning of subcortical response consistency (characteristically observed between adolescence and young adulthood) in music vs control group	Very Low
									Auditory processing (cortical onset response)	Positive	Significantly greater increase in cortical onset response (correlate of auditory maturation) in music vs control group	Very Low
Varnet et al.	2015	No	Cross-sectional	38 adults (mean age 22.9); 19 musicians, 19 nonmusicians; 12 male/26 female	France	Instrumental	7-22 years musical practice	Unspecified	Speech in noise	Positive	Significantly lower speech in noise threshold in musicians vs nonmusicians (Levitt 1971 3-down, 1-up staircase procedure)	Low
									Auditory processing	Positive	Significantly more consistent response to auditory stimuli and greater speed of auditory learning in musicians vs nonmusicians (Auditory Classification Image approach)	Low
Zendel & Alain	2012	No	Cross-sectional	163 adults (age range 18-91); 74 musicians, 89 nonmusicians; 86 female, 77 male	Canada	Unspecified	At least 6 years formal music lessons	Unspecified	Speech in noise	Positive	Significant association between musical training hours and superior speech in noise perception (QuickSIN)	Low

										Auditory processing (threshold)	NO effect	NO significant effect of music training on auditory threshold, only age significant (minimum detectable amplitude at various frequencies)	Low
										Auditory processing (temporal acuity (gap detection))	Positive	Significantly less decline in auditory temporal acuity in musicians vs nonmusicians (minimum detectable gap between sounds - 3 down, 1 up as per Levitt 1971)	Low
										Pitch discrimination (Mistuned harmonic detection)	Positive	Significantly better, across all ages, in musicians vs nonmusicians (2AFC procedure)	Low
Zendel & Alain	2009		No	Cross-sectional	28 adults (mean age 30.6; 14 musicians, 14 nonmusicians), 15 female/13 male	USA	Unspecified	Unspecified	Unspecified	Pitch discrimination (Two-tone detection)	Positive	Significantly more sensitive detection of two mis-matched tones (harmonically) in musicians vs nonmusicians	Low
Zendel et al.	2015		No	Cross-sectional	26 adults (age range 18-35; 13 musicians, 13 nonmusicians), 14 female/12 male	Canada	Instrumental & vocal	Average 15.5 years musical experience	Average 23.4 hours weekly music practice	Words in noise	Positive	Significantly better words in noise perception in musicians vs nonmusicians in most difficult condition (original test procedure)	Low
Daykin et al, 2013													
Anderson & Overy	2010	Excluded - Descriptive outcomes only, no statistical analyses of time or intergroup differences											
Baker & Homan	2007	Excluded - descriptive outcomes only, no quantitative data or statistical analyses											
Bittman, Dickson & Coddington	2009		Yes (Yamaha & Remo)	RCT (crossover design)	52 'at risk' youth aged 14-18 (30 females; 22 males)	USA	Percussion & keyboard education	6 weeks	1 hour, weekly	Social functioning	No effect	No significant benefit of music intervention vs control on social functioning (Child and Adolescent Functional	Moderate

											Assessment Scale (CAFAS))	
									Psychosocial function	No effect	No significant benefit of music intervention vs control on psychosocial functioning (Adolescent Psychopathology Scale (APS))	Moderate
									Anger	No effect	No significant benefit of music intervention vs control on anger (Adolescent Anger Rating Scale (AARS))	Moderate
									Depression	Positive	Significant improvement vs. control in depression (Reynolds Adolescent Depression Scale, 2nd edition (RADS 2))	Moderate
de Carlo & Hockman	2003	Excluded - analysis of rap music for therapeutic reasons, but no active music participation										
de Roeper & Savelsberg	2009	Excluded - qualitative outcomes only, no attempt to measure effects										
Gann	2010	Excluded - unpublished doctoral dissertation, no access available										
Kennedy	1998	No	RCT	45 juvenile delinquents or 'at risk' youth aged 7-19 with little/no guitar experience, sex unspecified (n=9 across 5 groups; all groups received 30 mins weekly guitar lessons + 30 min performance training, 15/15 min performance/cognitive training, 30 min cognitive training, 30 min vicarious	USA	Guitar instruction + performance training	3 months	1 hour, weekly (30 mins guitar instruction + 30 mins performance training)	Self-esteem	Positive	Significant improvement in self esteem (Rosenberg Self Esteem measure) in guitar instruction + performance (with or without cognitive training) groups vs. other groups with no performance training	High

				experience no training)								
Lashua	2005	Excluded - analysis of 'remixing' intervention for at-risk youth - participants remixed songs using computer software, not ‘active performing arts’ participation										
Lotter	2003	Excluded - qualitative outcomes only										
Tyson	2002	Excluded - focus on listening and analysis to rap music										
Woodward, Sloth-Nielson & Mathiti	2008	Excluded - mostly qualitative outcomes, no analysis or contextualization of the one quantitative outcome (recidivism rate)										
Daykin et al., 2018 *(**Supplementary Table 1. Quantitative Outcomes – included only**)*												
Anderson & Overy	2010	Excluded - Descriptive outcomes only, no statistical analyses of time or intergroup differences										
Baldari et al.	2010	Excluded - music listening during exercise										
Berkiroglu et al.	2013	Excluded - clinical population (hypertension)										
Bensimon et al.	2015	Excluded - music listening										
Bensimon & Gilboa	2010	Excluded - study of 'Musical Presentation' (a therapeutic activity in which participants in a group present themselves using music of their choice)										
Boothby & Robbins	2011	Excluded - music listening										
Burns et al.	2002	Excluded - music listening										
Campion & Levita	2014	Excluded - music listening										
Carissoli et al	2015	Excluded - music listening										
Chan et al.	2010	Excluded - music listening										
Chan et al.	2012	Excluded - music listening										
Chang et al.	2015	Excluded - music listening										
Chang et al.	2008	Excluded - music listening										
Chen et al.	2015	Excluded - music listening										
Cohen et al.	2006	No	Nonrandomized experimental study	166 English speaking healthy, ambulatory adults older than 64 (mean age = ~79; 76 control group; 90 intervention group), sex unspecified	USA	Singing	30 weeks	Once per week (duration unspecified)	Overall health	Positive	Significantly improved overall health in intervention vs control group (*statistical comparison only of post-intervention data, no accounting for baseline levels/intergroup analysis of change)(Self-report - bespoke instrument)	Very Low

	Health system utilization	Positive	Significantly reduced number of doctors visits in last 12 months in intervention vs control group (*statistical comparison only of post-intervention data, no accounting for baseline levels/intergroup analysis of change)(Self-report - bespoke instrument)	Very Low
	Medication usage (over the counter)	Positive	Significantly reduced usage of over the counter medication in intervention vs control group (*statistical comparison only of post-intervention data, no accounting for baseline levels/intergroup analysis of change)(Self-report - bespoke instrument)	Very Low
	Medication usage (prescription)	No effect	No significant difference in prescription medication usage in intervention vs control group (*statistical comparison only of post-intervention data, no accounting for baseline levels/intergroup analysis of change)(Self-report - bespoke instrument)	Very Low
	Falls incidence	Positive	Significantly reduced number of falls in intervention vs control group (*statistical comparison only of	Very Low

					post-intervention data, no accounting for baseline levels/intergroup analysis of change)(Self-report - bespoke instrument)	
			Other health problems	Positive	Significantly reduced reporting of other health problems in intervention vs control group (*statistical comparison only of post-intervention data, no accounting for baseline levels/intergroup analysis of change)(Self-report - bespoke instrument)	Very Low
			Morale	Positive	Significantly improved morale in intervention vs control group (*statistical comparison only of post-intervention data, no accounting for baseline levels/intergroup analysis of change)(Self-report - bespoke instrument)	Very Low
			Depression	No effect	No effect of intervention on depression vs control (statistical analysis of change in depression)(Geriatric Depression Scale - Short Form)	Low
			Loneliness	Positive	Significantly reduced loneliness in intervention vs control group (appropriate statistical analysis	Low

											accounting for baseline levels)(Loneliness Scale - III)	
									Social activity level	Positive	Significantly higher numbers of weekly social activities in intervention vs control group (*statistical comparison only of post-intervention data, no accounting for baseline levels/intergroup analysis of change)(Self-report - bespoke instrument)	Very Low
Cohen	2009	No	Nonrandomized experimental study	20 male inmates (mean age 35.5; 10 singers, 10 control)	USA	Singing	9 weeks (data analyzed only pre-post the final performance)	90 minutes, weekly	Wellbeing	No effect	No significant acute wellbeing benefit of intervention vs control (Friedman Well-being Scale)	Low
				58 male inmates and community members (10 control - mean age 34.5; 13 'general population inmate singers' - mean age 37.9; 25 'volunteer singers' from surrounding community)	USA	Singing	One performance	Once	Wellbeing	No effect	No significant acute wellbeing benefit of intervention vs control (Friedman Well-being Scale)	Low
Cooke et al.	2010	Excluded - clinical population (dementia)										
Cooke et al.	2010	Excluded - clinical population (dementia)										
Coulton et al.	2015	No	RCT	258 older adults (inclusion criteria: >/= 60 years old)(mean age 69.2; 214 female, 44 male; 27 control, 131 intervention	UK	Singing	14 weeks	90 minutes, weekly	Quality of Life (mental)	Positive	Significant improvement in quality of life vs control at both immediate post (3 month) and 3 month-post (SF12)	High
									Quality of Life (physical)	No effect	No significant effect of intervention on physical quality of life at either immediate post or 3-	High

												month FU timepoints(SF12)	
										Anxiety	Positive	Significantly reduced anxiety in control vs intervention participants at immediate post but not 3-month FU timepoints (Hospital Anxiety and Depression Scale)	High
										Depression	Positive	Significantly reduced depression in control vs intervention participants at immediate post but not 3-month FU timepoints (Hospital Anxiety and Depression Scale)	High
Field et al.	1997	Excluded - music listening											
Gold et al	2014	Excluded - multimodal music therapy approach judged to not closely resemble 'normal' performing arts participation due to prevalence of inactive modalities (i.e. verbal reflection, music listening)											
Geutin et al.	2009	Excluded - clinical population (dementia)											
Gupta & Gupta	2005	Excluded - music listening											
Haslam et al.	2014	Excluded - 'music-based reminiscence'											
Kim et al.	2011	Excluded - clinical population (stroke)											
Koelsch, Offermanns & Franzke	2010		No	RCT	154 adults (81 music group (improvised percussion play-along to recorded music) - mean age 24.4; 73 control group (music listening with rhythmic tapping to beat) - mean age 24.9), 78 females/76 males	Germany	Group drumming	41 minutes	Once	Depression/ anxiety	Positive	Significant reductions in depression in music vs control groups (POMS)	High
										Fatigue	Positive	Significant reductions in fatigue in music vs control groups (POMS)	High
										Vigor	Positive	Significant increase in vigor in music vs control group (POMS)	High
										Mood (Irritability)	Positive	Significant reduction in irritability in music vs control group (POMS)	High
										Valence / positive affect	Positive	Significantly increased attractiveness of music playing vs music listening (control)	High

											activity (Self-assessment manikins (SAMs))	
									Arousal	Positive	Significantly increased arousal in music vs control group (Self-assessment manikins (SAMs))	High
									Mood (Happiness)	Positive	Significantly increased happiness in music vs control group (Self-assessment manikins (SAMs))	High
									Mood (Anger)	Positive	Significantly reduced anger in music vs control group (Self-assessment manikins (SAMs))	High
									Mood (Sadness)	Positive	Significantly reduced sadness in music vs control group (Self-assessment manikins (SAMs))	High
									Anxiety	Positive	Significantly reduced anxiety in music vs control group (Self-assessment manikins (SAMs))	High
									Mood (Disgust)	Positive	Significantly reduced feelings of disgust in music vs control group (Self-assessment manikins (SAMs))	High
Kreutz	2014	No	Nonrandomized experimental trial	21 adults participating in a research choir (age range 18-65; median age ~50; 16 female, 5 male)	Germany	Group singing	30 minutes	Once	Mood (Positive feelings)	Positive	Significantly increased positive feelings in singing group vs control (ad hoc Likert scale)	Very Low
									Mood (Negative feelings)	No effect	No significant differences in negative feelings between singing vs control groups (ad hoc Likert scale)	Very Low

									Cortisol	No effect	No significant differences in cortisol changes in singing group vs control	Low
									DHEA	No effect	No significant differences in cortisol changes in singing group vs control	Low
									Cortisol-DHEA ratio	No effect	No significant differences in cortisol changes in singing group vs control	Low
Lee et al.	2010	Excluded - music listening										
Lord et al.	2012	Excluded - clinical population (COPD)										
Perkins & Williamon	2014	No	Non randomized longitudinal intervention study	98 older adults (age >/= 50)(mean age 67.9; 30 controls, 32 music-learning (higher socioeconomic status), 36 music-learning (lower socioeconomic status)), 74 female/22 male/2 unspecified	UK	Instrumental music lessons; 'creative music workshops'	10 weeks	60-90 mins, weekly	Wellbeing (mental)	No effect	No significant differences between music & control group (Short Warwick-Edinburgh Mental Well-being Scale)	Low
									Nutrition	NO effect	No significant difference between music & control (Health-Promoting Lifestyle Profile II (HPLPII))	Low
									Interpersonal Relations	No effect	No significant difference between music & control (Health-Promoting Lifestyle Profile II (HPLPII))	Low
									Spiritual Growth	Positive	Significantly greater spiritual growth in music group (high socioeconomic status ONLY) vs control (Health-Promoting Lifestyle Profile II (HPLPII))	Low
									Stress Management	No effect	No significant difference between music & control (Health-Promoting	Low

											Lifestyle Profile II (HPLPII))	
									Health responsibility	No effect	No significant difference between music & control (Health-Promoting Lifestyle Profile II (HPLPII))	Low
Sarkamo et al.	2014	Excluded - clinical population (dementia)										
Sole et al.	2010	Excluded - health outcomes only analyzed as a composite of singing, music therapy & music appreciation groups										
Sun & Buys	2012	Excluded - clinical population (chronic conditions)										
Sun et al.	2013	Excluded - clinical population (one or more chronic diseases)										
Sole et al.	2010	Excluded - health outcomes only analyzed as a composite of singing, music therapy & music appreciation groups										
Valentine & Evans	2001	No	Non randomized experimental cohort study	23 university singers (mean age 21.2), sex unspecified - mixed	UK	Singing	30 minutes	Once	Blood pressure	No effect	No significant pre-post changes	Very Low
									Mood (tense arousal)	Positive	Significant pre-post improvement in singing group, although not to the same magnitude as in swimming group (UWIST model adjective checklist (UMACL)	Very Low
									Mood (energetic arousal)	Positive	Significant pre-post improvement in singing group, although not to the same magnitude as in swimming group (UWIST model adjective checklist (UMACL)	Very Low
									Mood (hedonic tone)	Positive	Significant pre-post improvement in singing group, although not to the same magnitude as in swimming group (UWIST model adjective checklist (UMACL)	Very Low
Warth et al.	2015	Excluded - clinical population (cancer)										

Wlodarczyk	2007	Excluded - clinical population (hospice)											
Wu	2002	Excluded - clinical population (depression)											
Eells, 2014													
Chang et al.	2008	Excluded - music listening											
Clift et al.	2008	Excluded - review											
Cooke et al.	2010	Excluded - clinical population (dementia)											
Guetin et al.	2009	Excluded - clinical population (dementia)											
Hays and Minichiello	2005	Excluded - qualitative results only											
Hicks-Moore	2005	Excluded - music listening											
Houston et al.	1998	Other review - Clift et al., 2010											
Lee, Chan & Mok	2010	Excluded - music listening											
Lesta and Petocz	2006	Excluded - clinical population (dementia)											
McCaffrey and Freeman	2003	Excluded - music listening											
McCaffrey and Locsin	2006	Excluded - music listening											
Mok and Wong	2003	Excluded - music listening											
Pickles	2003	Excluded - qualitative results only											
Skingley and Bungay	2010	Excluded - qualitative results only											
Sung et al.	2010	Excluded - music listening											
Twiss et al.	2006	Excluded - music listening											
Fancourt, Ockelford & Belai, 2014 *(**'active participation' studies only**)*													
Bittman et al	2001		Yes (Remo Drums)	RCT	60 adults (mean age ~30 (precise age unspecified); 31 men, 29 women): musical experience unspecified	USA	Group drumming	1 hour	Once	Anxiety	No effect	No effect of group drumming vs. control in anxiety (Beck Anxiety Inventory)	Low
										Depression	No effect	No effect of group drumming vs. control in depression (Beck Depression Inventory II)	Low

White blood cell count	No effect	No effect of group drumming vs. control on white blood cell count	Low
Serum cortisol	No effect	No effect of group drumming vs. Control on cortisol	Low
NK cell activity	Positive	Increased NK activity vs. Control at effector:target ratios of 6:1 & 12:1; no significant change in NK activity (vs control) at effector:target ratios of 25:1 & 50:1	Low
LAK cell activity	Positive	Increased LAK activity vs control in 2 conditions (with IFN-alpha at effector:target of 12:1 & 25:1); No change in LAK activity vs control in 2 conditions (with IL-2 at effector:target of 12:1 and 25:1); **potential confounder →** significant variability in change in baseline IFN-alpha levels pre-post in experimental vs control groups at effector:target 12:1 & 25:1	Low
IL-2	No effect	No effect of group drumming vs control on IL-2 (a posteriori measurement)	Low
IFN-alpha	No effect	No effect of group drumming vs control on IFN-alpha (a posteriori measurement)	Low
Plasma DHEA	No effect	No effect of group drumming vs control on plasma DHEA	Low

										DHEA to cortisol ratio	No effect	No effect of group drumming vs control on DHEA to cortisol ratio; *positive touted effect only within drumming group – not significant vs control	Low
Cai et al.	2001	Excluded - clinical population (cancer)											
Koyama et al.	2009		Yes (Yamaha Corp)	Single group study (uncontrolled)	27 young adults (mean age 27.9; 19 women, 8 men); no drumming experience	Japan	Group drumming	1 hour	Once	Tension/anxiety	Positive	Reduction pre-post drumming intervention (POMS subscale)	Very Low
										Depression/dejection	Positive	Reduction pre-post drumming intervention (POMS subscale)	Very Low
										Anger/hostility	Positive	Reduction pre-post drumming intervention (POMS subscale)	Very Low
										Vigor/activity	No effect	No change pre-post drumming intervention (POMS subscale)	Very Low
										Fatigue/inertia	Positive	Reduction pre-post drumming intervention (POMS subscale)	Very Low
										Confusion/bewilderment	Positive	Reduction pre-post drumming intervention (POMS subscale)	Very Low
										Total mood disturbance	Positive	Reduction pre-post drumming intervention (total POMS)	Very Low
										Adrenaline	No effect		Very Low
										Noradrenaline	No effect		Very Low
										NK cell activity	No effect		Very Low
										White blood cells	No effect		Very Low
										Neutrophils	No effect		Very Low
										Lymphocytes	No effect		Very Low
										T cells	No effect		Very Low
										B cells	No effect		Very Low
										CD4+ T cells	No effect		Very Low
										CD8+ T cells	No effect		Very Low
										CD4/CD8 ratio	No effect		Very Low

						Naive T cells	No effect		Very Low
						Memory T cells	No effect		Very Low
						Naive/memory cell ratio	No effect		Very Low
						NK cells (count	No effect		Very Low
						IFN-alpha production	No effect		Very Low
						IL-2 production	No effect		Very Low
						IL-4 production	No effect		Very Low
						IL-6 production	No effect		Very Low
						IL-10 production	No effect		Very Low
	27 older adults (mean age 70.3; 15 women, 12 men); no drumming experience	Japan	Group drumming	1 hour	Once	Tension/anxiety	Positive	Reduction pre-post drumming intervention (POMS subscale)	Very Low
						Depression/ dejection	Positive	Reduction pre-post drumming intervention (POMS subscale)	Very Low
						Anger/hostility	Positive	Reduction pre-post drumming intervention (POMS subscale)	Very Low
						Vigor/activity	No effect	No change pre-post drumming intervention (POMS subscale)	Very Low
						Fatigue/inertia	No effect	No change pre-post drumming intervention (POMS subscale)	Very Low
						Confusion/ bewilderment	No effect	No change pre-post drumming intervention (POMS subscale)	Very Low
						Total mood disturbance	Positive	Reduction pre-post drumming intervention (full POMS)	Very Low
						Adrenaline	No effect		Very Low
						Noradrenaline	Positive	Significant pre-post increase	Very Low
						NK cell activity	No effect		Very Low
						White blood cells	No effect		Very Low
						Neutrophils	No effect		Very Low
						Lymphocytes	Positive	Significant pre-post increase	Very Low

									T cells	Positive	Significant pre-post increase	Very Low
									B cells	No effect		Very Low
									CD4+ T cells	Positive	Significant pre-post increase	Very Low
									CD8+ T cells	No effect		Very Low
									CD4/CD8 ratio	No effect		Very Low
									Naive T cells	No effect		Very Low
									Memory T cells	Positive	Significant pre-post increase	Very Low
									Naive/memory ratio	No effect		Very Low
									NK cells (count	No effect		Very Low
									IFN-alpha production	Positive	Significant pre-post increase	Very Low
									IL-2 production	No effect		Very Low
									IL-4 production	No effect		Very Low
									IL-6 production	Positive	Significant pre-post increase	Very Low
									IL-10 production	No effect		Very Low
									Positive affect	Positive	Significant pre-post increase in positive affect in singing group vs control (Positive and Negative Affect Schedule (PANAS))	Low
Kreutz et al.	2004	No	Non-randomized intervention study (week 1 – singing; week 2 – listening (control))	31 members of an amateur choir (mean age = 56.9, range 29-74), 23 female/8 male	Germany	Singing	1 hour	Once	Negative affect	Positive	Significant pre-post decrease in negative affect vs control (Positive and Negative Affect Schedule (PANAS))	Low
									Serum immunoglobulin A / albumin	Positive	Significant pre-post increase in S-IgA/albumin vs control	Low
									Cortisol	No effect	No significant pre/post effect of singing on cortisol	Low
Kuhn	2002	No	RCT	33 undergraduate students (mean age = 20; 5 men, 28 women); 6	USA	Group music making (singing and drumming)	30 minutes	Once	Serum Immunoglobulin-A	Positive	Significant increase in S-IgA levels in active music group vs inactive	High

				participants with >1 year formal music training at high school/college level – group allocation unclear)							(listening) & control (no activity) groups	
Lane	1994	Excluded - clinical population (hospitalized patients)										
Lindblad, Hogmark & Theorell	2007	No	Non-randomized longitudinal cohort study	60 5th & 6th graders, age & sex unspecified	Sweden	Music education	8 months	1 hour, weekly	Cortisol	No effect	No effect of music education on cortisol levels from saliva samples collected at any timepoint vs control (significant within group change in music group in afternoon sample only)	Low
									Self-esteem	No effect	No effect of music education on self-esteem (within or between group) ('I think I am' questionnaire)	Low
									Social anxiety	No effect	No effect of music education on social anxiety (within or between group) (Social Anxiety Scale for Children-Revised (SASC-R) & Social Phobia and Anxiety Inventory-Parental (SPAI-P))	Low
									Social functioning	No effect	No effect of music education on self-esteem (within or between group) (Child Behaviour Check List (CBCL), completed by parents)	Low
Okada et al	2009	Excluded - clinical population (cerebrovascular disease/dementia)										
Suzuki et al.	2005	Excluded - clinical population (dementia)										
Gick, 2012												

Beck et al	2006	No	Single group cohort study	10 conservatory music majors (mean age 20.8; average 10.1 years singing), sex unspecified	USA	Singing	One rehearsal/ performance	Data collections from 4-11 rehearsals/performances over a 10 week period	Immunoglobulin A	Positive	Significant increase in IgA during performance and rehearsals	Very Low
Beck et al	2000	Other review - Clift et al., 2010										
Bygren et al	2009	Excluded – combined analysis of receptive (film, music or art events) & active (choir singing) arts participation										
Clift & Hancox	2001	Excluded - descriptive, uncontrolled survey-based cross-sectional study										
Clift et al	2007	Excluded - descriptive, uncontrolled survey-based cross sectional study of active participation										
Cohen et al	2006	Other review - Daykin et al., 2018										
Fechir et al	2008	Excluded – descriptive analysis of the demands and response to singing with incompatible outcomes										
Grape et al	2003	No	Cross-sectional	16 singers (8 professionals – mean age 40.2, 4 male/4 female; 8 amateurs – mean age 40.2, 2 male/6 female)	Sweden	Singing	45 min	Once	Cortisol	No effect	No significant time or group*time interactions	Very Low
									Heart rate variability	Mixed	Significant group * time interaction – increased HR variability (total power) in professionals, decreased HR variability in amateurs	Very Low
									TNF-alpha	Mixed	Significant group*time effect; increased TNF-alpha in professionals, but not amateurs	Very Low
									Positive affect	Positive	Significant increase in energy & feelings of joy and relaxation in both amateurs and professionals; significantly greater feelings of joy in amateurs vs professionals	Very Low
Harmat & Theorell	2010	Excluded – descriptive analysis of HR responses to music performance only (included in HR data)										
Hillman	2002	Excluded - descriptive, uncontrolled survey-based cross sectional study										
Houston et al	1998	Other review - Clift et al., 2010										
Kenny, David & Oates	2004	Excluded - descriptive, uncontrolled survey-based cross sectional study of anxiety in professional singers										
Pai et al	2008	No	Cross-sectional	107 adults (52 semiprofessional	UK	Singing	Mean 30.8 years singing	Unspecified	Daytime somnolence	No effect	No significant differences between	Low

				singers (mean age 46.3; 20 males, 36 females); 55 non-singers (mean age 43.3; 23 males, 32 females))							singing & non-singing groups (Epworth Sleepiness Scale)	
Silber	2005	Excluded – qualitative outcomes only										
Skingley & Bungay	2010	Excluded – qualitative outcomes only										
Unwin, Kenny & Davis	2002	Other review - Clift et al., 2010										
Valentine & Evans	2001	Other review - Daykin et al., 2018										
Wise, Hartmann & Fisher	1992	Other review - Clift et al., 2010										
Gordon, Fehd & McCandliss, 2015												
Bolduc & Lefebvre	2012	No	Non-randomized experimental study	54 kindergarten students (mean age = 4.8; 26 language group, 28 music and language group), 27 male/27 female)	Canada	Music education (general)	10 weeks	40 min/weekly	Phonological awareness (other)	Meta-analysis	Phonological awareness measure	Low
Cogo-Moreira et al.	2013	No	RCT	235 elementary school children with below average reading ability (mean age = 9.15; 114 music education group, 121 control group), 38.3% female	Brazil	Music education (general)	5 months	50 min/3x weekly	Reading fluency	Meta-analysis	Test of phonological awareness	Moderate
									Phonological awareness (other)	Meta-analysis	Accuracy of word reading (custom measure)	Moderate
Dege & Schwarzer	2011	No	RCT	41 pre-schoolers, 17 female/22 male; age 5-6	Germany	Music education (general)	20 weeks	10 min, daily	Phonological awareness (other)	Meta-analysis	Total of 4 subtests of Bielefelder Screening	Moderate
Gromko	2005	No	Non-randomized longitudinal cohort study	103 kindergarten children (across 2 schools; 43 treatment, 60 control; sex unspecified - mixed)	USA	Music education (general - singing & percussion focus)	4 months	30 min, weekly	Reading fluency	Meta-analysis	DIBELS letter naming fluency	Very Low
									Phonological awareness (other)	Meta-analysis	DIBELS phoneme-segmentation fluency	Very Low

Herrera et al	2011		No	RCT	29 preschool children (mean age = 4.5), sex breakdown unspecified – mixed	Spain	Music education (general)	8 weeks	1 hour/2x weekly	Phonological awareness (rhyming)	Meta-analysis	Rhyme oddity task (custom)	Moderate
										Phonological awareness (other)	Meta-analysis	Rhyme oddity task (custom)	Moderate
					27 preschool children (mean age = 4.5), sex breakdown unspecified – mixed					Phonological awareness (other)	Meta-analysis	Initial phoneme oddity task (custom)	Moderate
										Phonological awareness (rhyming)	Meta-analysis	Rhyme oddity task (custom)	Moderate
Moreno et al.	2011		No	RCT	60 children (age range 4-6; 30 music group – 12 male/18 female; 30 visual art group – 14 male/16 female)	Canada	Music education (computer administered general music education)	4 weeks	2, 1-hour sessions/5x weekly	Phonological awareness (rhyming)	Meta-analysis	Rhyming task from WJ-III	High
Moreno et al.	2009		No	RCT (pseudo?)	32 3rd graders (mean age = 8.3), 19 male/13 female	Portugal	Music education (general)	24 weeks	75 min/2x weekly	Reading fluency	Meta-analysis	Reading inconsistent words (from Portuguese European reading battery)	Moderate

Moritz et al	2013	No	Cross-sectional	30 kindergarteners (mean age = 5.6), 17 male/13 female	USA	Music education (general)	Unspecified	45 min/daily	Phonological awareness (rhyming)	Meta-analysis	Rhyming discrimination from Phonological Awareness Test (PAT)	Low
									Phonological awareness (other)	Meta-analysis	Isolation of initial phonemes from PAT	Low
Myant, Armstrong & Healy	2008	No	Non-randomized experimental study	59 children (mean age = 4.3), sex unavailable (no article access)	UK	Music education (general)	No article access	No article access	Phonological awareness (rhyming)	Meta-analysis	Rhyme test from Phonological Assessment Battery (PhAB)	Low
									Phonological awareness (other)	Meta-analysis	Alliteration test from PhAB	Low
Register	2004	No	Non-randomized experimental study	43 Kindergarten students (age 5-7), sex unspecified – mixed	USA	Music education (therapy focus)	15+ sessions	30 min/session	Reading fluency	Meta-analysis	Letter-naming fluency from DIBELS	Low
									Phonological awareness (other)	Meta-analysis	Initial sounds fluency from DIBELS	Low
Thomson, Leong & Goswami	2013	No	Non randomized experimental study	21 dyslexic children (mean age = 9.3), sex unspecified	UK	Rhythm intervention	6 weeks	30 min/weekly	Reading fluency	Meta-analysis	TOWRE	Low
									Phonological awareness (rhyming)	Meta-analysis	Rhyme test from Phonological Assessment Battery (PhAB)	Low
									Phonological awareness (other)	Meta-analysis	Spoonerisms from PhAB	Low
Yazejian & Peisner-Feinberg	2009	No	Non randomized experimental study	181 Head Start children (mean age = 4.4), sex breakdown unspecified – mixed	USA	Music & movement intervention	26 weeks	30 min/2x weekly	Phonological awareness (rhyming)	Meta-analysis	Rhyming from Early Phonological Awareness Profile (EPAP)	Low
									Phonological awareness (other)	Meta-analysis	Phoneme deletion from EPAP	Low
Hetland, 2000												
Bilhartz, Terry & Olson	2000	Yes (Kinder-musik Inter-national)	Non randomized experimental cohort study	66 children (4-5 years old), sex unspecified - mixed	USA	Kindermusic	30 weeks	75 min, weekly	Spatial ability	Meta-analysis		Low
Costa-Giomi	1999	No	Quasi-randomized longitudinal cohort study	81 4th graders; age 9 at start of intervention (58 girls, 59 boys)	Canada	Piano lessons	3 years	30-45 minutes, weekly (instruction time only)	Spatial reasoning	Meta-analysis		Very Low
Flohr et al	1998	No	RCT	22 children (aged 4-6), sex unspecified	USA	General music education	7 weeks	25 minutes, twice weekly	Spatial reasoning	Meta-analysis		High

Flohr, Miller and Persellin	1998		RCT	19 children (4-5 year old), sex unspecified	USA	General music education	10 weeks	25 minutes, twice weekly	Spatial reasoning, spatial ability	Meta-analysis	High
Flohr, Miller and Persellin	2000		RCT	20 children (age 4), sex unspecified	USA	General music education	16-18 weeks	25 minutes, twice weekly	Spatial reasoning	Meta-analysis	High
Graziano, Peterson & Shaw	1999	Yes (Yamaha Corp)	Non randomized experimental cohort study	53 2nd graders (age 6-8), sex unspecified	USA	Piano lessons	14 weeks	60 minutes, 3x/week	Spatial reasoning	Meta-analysis	Low
Gromko & Poorman	1998	No	Non randomized experimental cohort study	30 pre-schoolers (age 3-4), sex unspecified	USA	General music education	24 weeks	30 minutes, weekly	Spatial reasoning, spatial ability	Meta-analysis	Low
Hurwitz et al	1975	No	Retrospective cross sectional analysis	40 children (age 6-7), 20 male/20 female	USA	Kodaly music instruction	7 months	40 minutes, 5x/week	Spatial reasoning, intelligence, spatial ability	Meta-analysis	Very Low
Lazco	1985	No	Longitudinal cohort study	301 children (2 groups – 154 3rd graders (age 9-10); 147 6th graders (age 12-13)), sex unspecified	Hungary	Kodaly music instruction	Unspecified	4-6 classes, weekly	Intelligence	Meta-analysis	Low
Mallory & Philbrick	1995	Unknow n	Longitudinal cohort study	44 children (3-5 years old), sex unspecified	USA	General music education	6 months	60 min, weekly	Spatial reasoning	Meta-analysis	Low
Parente & O'Malley	1975	No	Non randomized experimental cohort study	24 children (5-9 years old), 12 male/12 female	USA	Snare drum	4 weeks	60 minutes, 2x/weekly	Spatial ability	Meta-analysis	Low
Persellin	1999	Unknow n	Longitudinal cohort study	12 children (5-6 years old), sex unspecified	USA	General music education	6 weeks	45 minutes, 3x/week	Spatial reasoning	Meta-analysis	Low
Rauscher	1999	Unknow n	RCT	66 children (5-6 years old), sex unspecified	USA	General music education	8 months	40 minutes, 1x/week	Spatial reasoning	Meta-analysis	High
Rauscher	1999	Unknow n	RCT	87 children (age 3-6), sex unspecified	USA	General music education	Two 6-month periods over 2 years	10 min, weekly	Spatial reasoning	Meta-analysis	High
Rauscher et al	1994	Unknow n	Longitudinal cohort study	33 children (3-4 years old), sex unspecified	USA	Voice & Piano lessons	8 months	30 minutes, 3x/week	Spatial reasoning	Meta-analysis	Low
Rauscher et al	1997	Yes (Yamaha Corp)	Longitudinal cohort study	78 children (3-4 years old), 42 male/36 female	USA	Voice & Piano lessons	6-8 months	10 min, weekly	Spatial reasoning, spatial ability	Meta-analysis	Low
Ruascher & Zupan	1999	No	Longitudinal cohort study	48 children (5-7 years old), sex	USA	General music education	Two 8-month periods over 2 years	20 minutes, 2x/week	Spatial reasoning	Meta-analysis	Low

				breakdown unspecified - mixed								
Taetle	1999	No	Longitudinal cohort study	68 children (5-6 years old), sex unspecified	USA	Orff, singing	4 months	30 min, 2x/week	Spatial reasoning, spatial ability	Meta-analysis		Low
Zulauf	1993	No	Longitudinal cohort study	353 adolescents (mean age unspecified), 174 female/179 male	Switzerland	General music education	3 years	5x/weekly (school days)	Intelligence, spatial ability	Meta-analysis		Low
Jaschke et al., 2013												
Costa-Giomi	2004	No	RCT	117 4th graders; 58 girls/59 boys; age 8-12	Canada	Piano instruction	3 years	30-45 minutes, weekly (instruction time only)	Language processing	No effect	No significant differences in language processing or mathematical achievement (Canadian Achievement Test 2)	Moderate
									Mathematical ability	No effect	No significant differences in language processing or mathematical achievement (Canadian Achievement Test 2)	Moderate
Courey et al.	2012	Excluded - not 'music participation', targeted music theory instruction to teach fractions										
Dege & Schwarzer	2011	Other review - Gordon, Fehd & McCandliss, 2015										
Gromko	2005	No	Non-randomized longitudinal cohort study	103 kindergarten children (across 2 schools; 43 treatment, 60 control; sex unspecified - mixed)	USA	Music education (general - singing & percussion focus)		30 min, weekly	Reading ability	No effect	No effect of music training on nonsense-word fluency (Dynamic Indicators of Basic Early Literacy Skills (DIBELS) test) vs. control	Very Low
		Yes - Gordon, Fehd & McCandliss, 2015							Reading ability		(Dynamic Indicators of Basic Early Literacy Skills (DIBELS) test - letter naming & phoneme segmentation subtest)	
Ho, Cheung and Chan	2003	No	Cross-sectional	90 male right handed participants (average age ~11)	Hong Kong	Classical instrumental education	1-5 years (music education experience)	1+ hour, weekly	IQ	No effect	No significant differences in IQ between individuals with and without music	Low

											training (Hong Kong Weschler Intelligence Scale for Children)	
Jentschke, Koelsch & Friederici	2005	No	Cross-sectional	28 participants (14 with music training experience; 14 without); age 10-12, 15 male/13 female	Germany	Instrumental or singing lessons	Unspecified	Unspecified	Reading ability	No effect	No significant differences between trained & untrained children on verbal scale of WISC-III	Very Low
Piro and Ortiz	2009	No	Non-randomized longitudinal cohort study	103 2nd grade elementary school children (mixed gender) in their 3rd year of piano instruction	USA	Piano instruction	10 months	40-45 min, twice weekly	Reading ability	Positive	Improved reading (vocabulary and verbal sequencing subtests of the Meeker Structure of Intellect) in music training group, but not control (no music) group	Very Low
Portowitz et al.	2009	No	Non-randomized longitudinal cohort study	81 'high-risk' elementary schoolchildren, aged 6-12 years: sex unspecified	Israel	Multimodal music education (music appreciation, private instrumental instruction, ensemble performances)	2 years	2-3 hours, weekly (split up)	IQ	Positive	Improved general IQ (Raven assessment) in music training group	Low
Register	2001	No	Non-randomized longitudinal cohort study	50 participants (25 control; 25 music training); 61 originally involved but 11 drop outs not analyzed; age 4-5; sex unspecified - mixed	USA	Music therapy sessions' (experimental group focused on teaching/ reinforcing prereading/pre-writing concepts; control group - general 'music therapy')	30 weeks	30 min, twice weekly	Writing ability	Positive	Improvements in both music groups in 'prewriting' (Print Awareness Test of Word Identification)	Very Low
									Reading ability	Positive	Improvements in both music groups in 'prereading' (The Print Awareness Test for Logos; Print Concepts Checklist)	Very Low
Rickard, Bambrick & Gill	2012	No	RCT	84 7th graders (47 music training group; 37 control (drama lessons); aged 10-13, sex unspecified - mixed	Australia	General music education	6.5 months	Unspecified	IQ	No effect	No significant differences in music vs. control in non-verbal IQ (Kaufman Brief Intelligence Test)	Low
									Writing ability	No effect	No significant differences in music vs.	Low

										control in verbal IQ (Kaufman Brief Intelligence Test)	
Rickard, Bambrick & Gill	2012	No	RCT	69 5th and 6th graders (38 in music group; 31 in control (no intervention)); sex unspecified - mixed	Australia	General music education	5 months	1 hour, weekly	Reading ability	Negative	Significantly worse improvement in reading skills (Victorian Education Learning Standards (VELS)) in music intervention vs. control	High
									Mathematical ability	Positive	Significantly greater improvement in mathematical achievement (VELS) in music group vs. control	High
Schellenberg	2004	No	RCT	144 6-year old children (36 keyboard lessons, 36 voice lessons, 36 drama lessons, 36 control (waitlist)); sex unspecified - mixed	Canada	Keyboard or singing lessons	36 weeks	Unspecified	IQ	Positive	Significantly greater improvement in IQ in music training groups vs. controls (drama & no training)(WISC-III)	High
									Mathematical ability	No effect	No significant differences between music training & control groups in mathematical achievement (Kaufman Test of Educational Achievement (K-TEA) mathematical applications and computation subscales)	High
									Reading ability	No effect	No significant differences between music training & control groups in reading achievement (Kaufman Test of Educational Achievement (K-TEA) reading decoding, reading comprehension & spelling subscales)	High
Tsang & Conrad	2011	No	Cross-sectional	69 children between the ages of 5 and 9	Canada	Formal music lessons (21	Average 1.27 years (range .5	Unspecified	Cognitive - auditory	Positive	Significantly better performance by music	Very Low

				(43 control; 26 with music training; sex unspecified - mixed)		piano, 2 violin, 2 voice, 1 voice & cello)	- 5 years) of music training				trained children on phonological skills test (Test of Auditory Analysis Skills)	
									Reading ability	No effect	No significant differences in trained vs. untrained children in receptive vocabulary (Woodcock Reading Mastery Test)	Very Low
									Reading ability	No effect	No significant differences between trained and untrained individuals on word identification(Peabody Picture Vocabulary Test)	Very Low
Moore, 2013												
Ball et al.	2007	Excluded - music listening										
Baumgartner et al.	2006	Excluded - music listening										
Bengtsson et al	2007	No	Cross-sectional	11 professional male Swedish concert pianists (mean age = 32)	Sweden	Piano	40 second trials ('improvise' & 'reproduce')	Once	Emotional Regulation	Unclear	Frontal & temporal association areas activated when musicians improvised; dlPFC implicated in creative aspect of behavior when adapted to satisfy a result	Very Low
Berkowitz & Ansari	2008	No	Cross-sectional	12 classically trained undergraduate pianists (mean age = 21.9), 8 female/4 male	USA	Piano	23.3 minutes	Once	Emotional regulation (Anterior Cingulate Cortex Activation)	Positive	Increased ACC activation during melodic and rhythmic improvisation	Very Low
Berns & Moore	2012	Excluded - music listening										
Berns et al.	2010	Excluded - music listening										
Blood & Zatorre	2001	Excluded - music listening										
Brown & Martinez	2006	Excluded - music listening										
Brown et al	2004	Excluded - music listening										

Brown, Martinez & Parsons	2006	No	Cross-sectional	10 amateur musicians (mean age 25 years), 5 male/5 female	USA	Singing	8.4 minutes	Once	Emotional regulation (Anterior Cingulate Cortex Activation)	Positive	Increased ACC activation during melodic improvisation	Very Low
Callan et al	2006	No	Cross-sectional	16 adults with no significant musical experience (mean age = 26), 5 female/11 male	Japan	Singing	Unclear (48 bars)	Once	Emotional regulation (Orbitofrontal cortex activation)	No effect	No significant increase in OFC activation during singing vs speaking (increased activation during listening to singing only)	Very Low
Coen et al.	2009	Excluded - music listening										
de Manzano & Ullen	2012	No	Cross-sectional	18 right handed classical concert pianists (mean age = 39; 1 female, 17 male)	Sweden	Piano	20 minutes	Once	Emotional regulation (Anterior cingulate cortex & dorsolateral prefrontal cortex activation)	Positive	Increased cortical activation associated with response inhibition and selection but unclear implications for emotional regulation	Very Low
Dyck et al.	2011	Excluded - music listening										
Eldar et al.	2007	Excluded - music listening										
Flores-Gutierrez et al	2007	Excluded - music listening										
Ford et al.	2011	Excluded - music listening										
Foss et al	2007	Excluded - music listening										
Fujisawa & Cook	2011	Excluded - music listening										
Green et al.	2008	Excluded - music listening										
Hugdahl et al.	1999	Excluded - music listening										
James et al.	2008	Excluded - music listening										
Jenata	2009	Excluded - music listening										
Jeffries, Fritz & Braun	2003	No	Cross-sectional	20 adults with no musical experience (mean age = 34), 8 female/12 male	USA	Singing	Unclear	Once	Emotional regulation (dorsolateral prefrontal cortex activation)	Positive	Increased dorsolateral prefrontal cortex activation in singing vs speaking	Very Low
Jerde et al.	2011	Excluded - music listening										
Kleber et al	2010	No	Cross-sectional	49 adults with varied singing experience (10 professional opera	Germany	Singing	11 min	Once	Emotional regulation (anterior cingulate cortex,	Positive	Increased dlPFC activation associated with increasing singing practice; increased	Very Low

				singers (mean age = 38; 7 females, 3 males); 21 vocal students (mean age 25 years; 14 females, 7 males); 18 laymen (mean age 23.5 years; 6 females, 12 males))					ventrolateral prefrontal cortex, amygdala activation)		activation of ACC in university singers	
Kleber et al	2007	No	Cross-sectional	16 classical singers (mean age 31.1; 5 male, 11 female)	Germany	Singing	Unclear	Once	Emotional regulation (dorsolateral prefrontal cortex activation)	Unclear	Increased dlPFC activation during imagined vs overt singing	Very Low
Knösche et al	2005	Excluded - music listening										
Koelsch et al.	2006	Excluded - music listening										
Koelsch et al.	2008	Excluded - music listening										
Lee et al.	2011	Excluded - music listening										
Lerner et al.	2009	Excluded - music listening										
Limb & Braun	2008	No	Cross-sectional	6 right-handed male professional pianists (mean age = 34.2)	USA	Piano	34.3 minutes	Once	Emotional regulation (orbital prefrontal cortex, dorsolateral prefrontal cortex & amygdala activation)	Mixed	Increased OFC & amygdala activation during improvisation; however, also decreased activation in dlPFC during improvisation	Very Low
Menon & Levitin	2005	Excluded - music listening										
Mitters-Chiffthaler et al.	2007	Excluded - music listening										
Mizuno & Sugishita	2007	Excluded - music listening										
Mutshuler et al.	2010	Excluded - music listening										
Nakamura et al.	1999	Excluded - music listening										
Ohnishi et al.	2001	Excluded - music listening										
Pallesen et al.	2009	Excluded - music listening										
Pallesen et al.	2005	Excluded - music listening										
Pallesen et al.	2010	Excluded - music listening										

Perry et al.	1999		No	Cross-sectional	13 adults without substantial musical training (mean age = 24), 6 male/7 female	Canada	Singing	1 minute	Once	Emotional regulation (anterior cingulate cortex activation)	Positive	Increased ACC activation during singing	Very Low
Ruiz, Jabusch & Altenmueller	2009		No	Cross-sectional	19 healthy professional pianists (mean age = 22), 8 female/11 male	Germany	Piano	2 hours	Once	Emotional regulation (anterior cingulate cortex activation)	Unclear	Increased ACC activation associated with error monitoring during piano performance, but unclear significance for associations between music performance and emotional regulation	Very Low
Satoh et al.	2001	Excluded - music listening											
Suda et al.	2008	Excluded - music listening											
Thaut et al.	2009	Excluded - music listening											
Vogt et al	2007		No	Cross-sectional	32 healthy adults (16 male, 16 female; 16 non-guitarists - mean age = 26.6; 16 guitarists - mean age = 27.3)	Germany	Guitar	Unclear	Once	Emotional regulation (dorsolateral prefrontal cortex activation)	Positive	Increased activation of lPFC during chord performance in both non-guitarists and guitarists	Very Low
Zarate & Zatorre	2008		No	Cross-sectional	24 healthy adults (mean age = 23; 12 experienced singers, 6 male/6 female; 12 non-musicians, 6 male/6 female)	Canada	Singing	Unclear	Once	Emotional regulation (anterior cingulate cortex activation)	Positive	Increased ACC activation during singing in both singers & non-musicians	Very Low
Zarate, Wood & Zatorre	2010		No	Cross-sectional	9 healthy, experienced singers (mean age = 23), 3 male/6 female	Canada	Singing	Unclear	Once	Emotional regulation (anterior cingulate cortex activation)	Positive	Increased ACC activation during 'compensate tasks' (i.e. singer must compensate for pitch shifts)	Very Low
Phillips & Becker, 2019													
Anderson & Gustavson	2016	Excluded - study of knitting, not music, intervention											
Bittman et al.	2003		Yes (Yamaha, Remo)	RCT	125 healthcare workers (Retirement Community; mean age 43.8), 24 male/101 female	USA	Group drumming	6 weeks	1 hour, weekly	Burnout (emotional exhaustion)	Positive	Significant pre/post improvement (**1-tailed t-test**) following intervention but no analysis of data	Low

											vs control (Maslach Burnout Inventory)	
									Burnout (depersonalization)	No effect	No significant pre/post differences (Maslach Burnout Inventory)	Low
									Burnout (personal accomplishment)	Positive	Significant pre/post improvement (**1-tailed t-test**) following intervention but no analysis of data vs control (Maslach Burnout Inventory)	Low
									Mood	Positive	Significant pre/post decrease in mood disturbance (although no analysis of data vs control)(Profile of Mood States)	Low
Bormann et al.	2006	No	Single group study	62 Healthcare workers (mean age = 47.8; 54 female)	USA	'mantram' singing	5 weeks	90 minutes, weekly	Stress	Positive	Significant pre-post reduction in stress (Perceived Stress Scale)	Very Low
									Anxiety (state)	No effect	No significant pre-post effect on state anxiety (Spielburger State-Trait Anxiety Inventory)	Very Low
									Anxiety (trait)	Positive	Significant pre-post reduction in trait anxiety(Spielburger State-Trait Anxiety Inventory)	Very Low
									Anger (state)	No effect	No significant pre-post effect on state anger (Spielburger State-Trait Anger Inventory)	Very Low
									Anger (trait)	Positive	Significant pre-post reduction in trait anger (Spielburger State-Trait Anger Inventory)	Very Low
									Quality of life	Positive	Significant pre-post improvement in quality of life (Quality of Life Enjoyment & Satisfaction Short Form)	Very Low

									Spiritual Wellbeing	Positive	Significant pre-post improvement in spiritual wellbeing (Total Spiritual Well-Being Scale)	Very Low
Hilliard	2006	No	Nonrandomized experimental study	17 hospice workers (age range 28-60; 11 female, 6 male)	USA	Group music-making	6 weeks	60 minutes, weekly	Compassion fatigue	No effect	No pre-post effect on compassion fatigue (subscale of Compassion Satisfaction/Fatigue Self-Test for Helpers)	Very Low
Ifrach & Miller	2016	Excluded - visual art, not music, intervention										
Italia et al.	2008	Excluded - visual art, not music, intervention										
Karpaviciute & Macijauskiene	2016	Excluded - painting, not music, intervention										
Kravits et al.	2010	Excluded - visual art, not music, intervention										
MacPherson	2008	Excluded - storytelling, not music, intervention										
Potash et al.	2014	Excluded - visual art, not music, intervention										
Rice et al.	2014	Excluded - storytelling, not music, intervention										
Salzano et al.	2013	Excluded - visual art, not music, intervention										
Sands et al.	2008	Excluded - 'narrative training', not music, intervention										
Wlodarcyzyk	2013	Excluded - 'songwriting component the main therapeutic intervention'; involves fill in the blank adaptation of 'Fire and Rain' by James Taylor with subsequent lyric analysis; active music participation not primary component										
Raglio et al., 2019												
Beck et al.	2015	Excluded - guided music imagery, not music participation, intervention										
Bittman et al.	2004	Other review - Yap, Kwan & Ang, 2017										
Bae	2011	No	RCT (only comparative, not inactive control group)	20 female graduate music therapy students (mean age = 27.6)	South Korea	Group drumming	4 weeks	60 mins, weekly	Job engagement	No effect	No significant pre-post impact of group drumming on job engagement; *effects vs other music group (guided musical imagery) not considered due to unclear relevance to umbrella review aims (Utrecht work and wellbeing survey (UWES))	Low
									Anxiety	No effect	No significant pre-post impact of group	Low

			drumming on anxiety; *effects vs other music group (guided musical imagery) not considered due to unclear relevance to umbrella review aims (State-trait anxiety inventory (STAI))	
	Mood	No effect	No significant pre-post impact of group drumming on mood disturbance; *effects vs other music group (guided musical imagery) not considered due to unclear relevance to umbrella review aims (Profile of mood states (POMS))	Low
	Perceived self-efficacy	No effect	No significant pre-post impact of group drumming on perceived self-efficacy; *effects vs other music group (guided musical imagery) not considered due to unclear relevance to umbrella review aim (General self-efficacy scale (GSE))	Low
	Positive affect	Positive	Significant pre-post improvement in positive affect; *effects vs other music group (guided musical imagery) not considered due to unclear relevance to umbrella review aims (Positive affect, negative affect schedule (PANAS))	Low
	Negative affect	No effect	No significant pre-post impact of group drumming on negative	Low

											affect; *effects vs other music group (guided musical imagery) not considered due to unclear relevance to umbrella review aims (Positive affect, negative affect schedule (PANAS))	
Brooks et al.	2010	Excluded - music & imagery, not music participation, intervention										
DuRousseau et al.	2011	Excluded - brain music, not music participation, intervention										
Huang & Shih	2011	Excluded - music listening										
Lesiuk	2008	Excluded - music listening										
Lesiuk	2010	Excluded - music listening										
Miskovic et al.	2008	Excluded - music listening										
Shih et al.	2009	Excluded - music listening										
Shih et al.	2012	Excluded - music listening										
Shih et al.	2016	Excluded - music listening										
Speer	2011	Excluded - music listening										
Wlodarczyk	2010	Excluded - 'songwriting component the main therapeutic intervention'; involves fill in the blank adaptation of 'Fire and Rain' by James Taylor with subsequent lyric analysis; active music participation not primary component										
Skingley & Vella-Burrows, 2010 *'Music in everyday life' section only (excluded Table 1 - 'Studies relating to music or singing and people with dementia' and Table 2 - 'Studies relating to music and specific disorders')*												
Bungay & Skingley	2008	Excluded - qualitative results only										
Cohen et al.	2006	Other review - Daykin et al., 2018										
Hays	2005	Excluded - qualitative results only										
Hays & Mihichiello	2005	Excluded - qualitative results only										
Pickles	2003	Excluded - qualitative results only										
Talmini et al, 2017												
Anaya, Pisoni & Kronenberger	2016	No	Cross-sectional	48 adults (mean age 22.1; 24 musicians, 24 nonmusicians); sex unspecified	USA	Piano/organ	average 15.5 years of musical training	average 16.3 hours of weekly practice	Short-term memory	Meta-analysis		Low
Bialystok & DePape	2009	No	Cross-sectional	46 adults (mean age 24.3; 22 musicians,	Canada, UK	Instrumental	Average 16.6 years of	Average 2 hours of	Short-term memory	Meta-analysis		Low

				24 nonmusicians); sex unspecified			musical training	daily practice	Working memory	Meta-analysis	Low
Boebinger et al.	2015	No	Cross-sectional	50 adults (mean age 27.2; 25 musicians, 25 nonmusicians)l sex unspecified	UK	Instrumental	Average 22.7 years of musical training	Practice at least 3x/weekly over the 3 years before testing	Short-term memory	Meta-analysis	Low
									Working memory	Meta-analysis	Low
Brandler & Rammsayer	2003	No	Cross-sectional	70 adults (mean age 28.5, 39 female/31 male; 35 musicians, 35 nonmusicians)	Germany	Instrumental	>14 years of musical training	Unspecified	Long-term memory	Meta-analysis	Low
									Long-term memory	Meta-analysis	Low
Chan, Ho & Cheung	1998	No	Cross-sectional	60 adults (mean age 19.8; 30 musicians, 30 nonmusicians), sex unspecified	Hong Kong	Instrumental	At least 6 years of music training before age 12	Unspecified	Long-term memory	Meta-analysis	Low
									Long-term memory	Meta-analysis	Low
Clayton et al.	2016	No	Cross-sectional	34 adults (mean age 23.5; 17 musicians, 17 nonmusicians), sex unspecified	USA	Instrumental	average 14.4 years of music training	Unclear	Working memory	Meta-analysis	Low
Franklin et al.	2008	No	Cross-sectional	25 adults (mean age 19.7; 12 musicians, 13 nonmusicians), 19 female/6 male	USA	Instrumental	At least 9 years continuous music training, beginning before age 10	Practice/ performance at least 15 hours/week	Long-term memory	Meta-analysis	Low
				20 adults (mean age 21.6; 11 musicians, 9 nonmusicians), 10 female/10 male					Working memory	Meta-analysis	Low
George & Coch	2011	No	Cross-sectional	32 adults (mean age 20.1; 16 musicians, 16 nonmusicians), 16 female/16 male	USA	Instrumental	Average 12.1 years of music training	Average 6.3 hours of weekly practice	Short-term memory	Meta-analysis	Low
									Working memory	Meta-analysis	Low
									Short-term memory	Meta-analysis	Low
Hansen, Wallentin & Vuust	2012	No	Cross-sectional	40 adults (mean age 21.1; 20 musicians, 20 nonmusicians), sex breakdown unspecified for included comparison - mixed	Denmark	Instrumental	Average 10.5 years of music training	Average 23.5 hours of weekly practice	Short-term memory	Meta-analysis	Low
									Working memory	Meta-analysis	Low
									Short-term memory	Meta-analysis	Low
									Working memory	Meta-analysis	Low
Helmbold, Rammsayer & Altenmueller	2005	No	Cross-sectional	140 adults (mean age 22.5; 70 musicians, 70	Germany	Instrumental	Average 17 years of music training	Unspecified	Long-term memory	Meta-analysis	Low

				nonmusicians), 74 female/66 male					Long-term memory	Meta-analysis	Low
Huang et al.	2010	No	Cross-sectional	20 adults (mean age 21.5; 10 musicians, 10 nonmusicians), sex unspecified	China	Piano	At least 8 years continuous music training begun before age 9	Unspecified	Long-term memory	Meta-analysis	Low
Jakobson et al.	2008	No	Cross-sectional	36 adults (mean age 19; 15 musicians, 21 nonmusicians), 23 female/13 male	Canada	Piano	Average 11.5 years of music training	Unspecified (11 of 15 in music group 'actively engaged in musical activities')	Long-term memory	Meta-analysis	Low
									Long-term memory	Meta-analysis	Low
Lee, Lu & Ko	2007	No	Cross-sectional	40 adults (mean age 22; 20 musicians, 20 nonmusicians), sex unspecified	Taiwan	Instrumental	Average 14.3 years of music training	Unspecified	Short-term memory	Meta-analysis	Low
									Working memory	Meta-analysis	Low
									Working memory	Meta-analysis	Low
Monahan, Kendall & Carterette	1987	No	Cross-sectional	22 adults (mean age not provided, introductory psychology students and older); 12 musicians, 10 nonmusicians), sex unspecified	USA	Instrumental	At least 5 years of music training	Unspecified	Short-term memory	Meta-analysis	Low
Okhrei, Kutsenko & Makarchuk	2017	No	Cross-sectional	66 adults (mean age 20; 28 musicians, 36 nonmusicians), sex unspecified	Ukraine	Music experience' (may include singing	10-14 years of music training	Unspecified	Short-term memory	Meta-analysis	Low
									Short-term memory	Meta-analysis	Low
Pallesen et al.	2010	No	Cross-sectional	21 adults (mean age 26.5; 11 musicians, 10 nonmusicians), 14 female/7 male	Finland	Instrumental	Unspecified (graduates of Sibelius Music Academy)	Unspecified	Short-term memory	Meta-analysis	Low
									Working memory	Meta-analysis	Low
Parbery-Clark et al.	2011	No	Cross-sectional	37 adults (mean age 50; 18 musicians, 19 nonmusicians); age range 45-65; sex unspecified	USA	Piano & violin	Average 50 years music training	practice at least 3x/weekly over the 3 years before testing	Working memory	Meta-analysis	Low
	2012	No	Cross-sectional	60 adults (mean age 19.5; 30 musicians,	USA	Unspecified	8+ years music training;		Short-term memory	Meta-analysis	Low

Ramachandra, Meighan & Gradzki				30 nonmusicians), sex unspecified			'University music students who began music training before age 10'	Practice at least 4 hours/week	Working memory	Meta-analysis	Low
Rodrigues, Loureiro & Caramelli	2014	No	Cross-sectional	76 adults (mean age 32.2; 38 musicians, 38 nonmusicians), 56 male/20 female	Brazil	Instrumental (symphony orchestra musicians)	Average 23 years music training	average 3.2 hours daily practice	Short-term memory	Meta-analysis	Low
Schiavio & Timmers	2016	No	Cross-sectional	20 adults (mean age 24.8; 10 musicians, 10 nonmusicians); sex unspecified	UK	Instrumental	Minimum 5 years music training	Unspecified	Long-term memory	Meta-analysis	Very Low
Schulze, Dowling & Tillmann	2012	No	Cross-sectional	40 adults (mean age 22.7; 20 musicians, 20 nonmusicians), sex unspecified	France	Instrumental	Average 17.7 years music training	Unspecified	Short-term memory	Meta-analysis	Low
									Working memory	Meta-analysis	Low
Schulze, Mueller & Koelsch	2011	No	Cross-sectional	33 adults (mean age 24.5; 16 musicians, 17 nonmusicians), 18 male/15 female	Germany	Instrumental	Unspecified (began music training at average 6.1 years old)	"Practice for several hours each day"	Short-term memory	Meta-analysis	Low
Schulze et al.	2011	No	Cross-sectional	33 adults (mean age 24.5; 16 musicians, 17 nonmusicians), 18 male/15 female	Germany	Instrumental	Unspecified	Unspecified	Working memory	Meta-analysis	Low
									Working memory	Meta-analysis	Low
Suarez, Elangovan & Au	2015	No	Cross-sectional	54 adults (mean age 22.6; 24 musicians, 30 nonmusicians), 11 male/43 female	Singapore	Instrumental	Average 10.4 years music training	Unspecified	Working memory	Meta-analysis	Low
									Short-term memory	Meta-analysis	Low
									Short-term memory	Meta-analysis	Low
									Long-term memory	Meta-analysis	Low
Talamini, Carretti, & Grassi	2016	No	Cross-sectional	36 adults (mean age 22.6; 18 musicians, 18 nonmusicians), 22 female/14 male	Italy	Instrumental	Average 12.3 years music training	Average 19.6 hours weekly practice	Short-term memory	Meta-analysis	Low
									Working memory	Meta-analysis	Low
Taylor & Dewhurst	2017	No	Cross-sectional	40 adults (mean age 21.7; 20 musicians, 20 nonmusicians), 22 male/18 female	UK	Instrumental	At least 4 years of music training	Practice at least once pre week	Long-term memory	Meta-analysis	Low
Vasuki et al.	2016	No	Cross-sectional	35 adults (mean age 25.8; 17 musicians,	Australia	Instrumental or singing	At least 10 years of music/singing	Unspecified - 'Still actively	Short-term memory	Meta-analysis	Low
									Working memory	Meta-analysis	Low

				18 nonmusicians), 10 male/25 female			experience beginning before age 9	practiced music'			
Weiss et al.	2014	No	Cross-sectional	57 adults (mean age 23.4; 42 musicians, 15 nonmusicians), sex unspecified	Israel	Instrumental or singing	Average 11.9 years music training	Unspecified	Short-term memory	Meta-analysis	Low
Zuk et al	2014	No	Cross-sectional	30 adults (mean age 24.8; 15 musicians, 15 nonmusicians), 18 male/12 female	USA	Instrumental	Unclear, likely 15+ years)	Average 21.9 hours weekly practice	Working memory	Meta-analysis	Low
Vaughn, 2000											
Annello	1972	No	Cross-sectional	326 9th-12th graders, 170 male/156 female	USA	Instrumental music education	1-4 years	Unspecified	Mathematics (correlation)	Meta-analysis	Low
Catterall, Chapleau & Iwanaga	1999	No	Cross-sectional	1476 high school seniors, sex unspecified	USA	Instrumental music education	1-5 years	Unspecified	Mathematics (correlation)	Meta-analysis	Low
Ciepluch	1988	No	Cross-sectional	80 9th-12th graders, sex unspecified	USA	Instrumental music education (band)	1-4 years	Unspecified	Mathematics (correlation)	Meta-analysis	Very Low
College Board	1988-1998 (not 1993)	No	Cross-sectional	5,732,282 high school seniors, mixed sex	USA	Instrumental or vocal music class	At least 1 course	Not specified	Mathematics (correlation)	Meta-analysis	Moderate
Costa-Giomi	1999	No	Quasi-randomized longitudinal cohort study	129 4th graders; age 9 at start of intervention (58 girls, 59 boys)	Canada	Piano lessons	3 years	30-45 minutes, weekly (instruction time only)	Mathematics (causation - experimental)	Meta-analysis	Very Low
Engdahl	1994	No	Retrospective cohort study	598 elementary school students (enrolled as 3rd graders), sex unspecified	USA	Instrumental music education (orchestra & band)	3 years	At least 90 minutes, weekly (school weeks)	Mathematics (correlation)	Meta-analysis	Low
Friedman	1959	No	Longitudinal cohort study	28 5th & 6th graders, sex unspecified	USA	Instrumental music education	1-2 years	7-8 hours, weekly	Mathematics (causation - experimental)	Meta-analysis	Low
Graziano, Peterson & Shaw	1999	Yes (Yamaha Corp)	Non randomized experimental cohort study	55 2nd graders, sex unspecified	USA	Piano lessons	14 weeks	60 minutes, 3x/week	Mathematics (causation - experimental)	Meta-analysis	Low

Kvet	1982	No	Cross-sectional	350 3rd-6th graders, sex breakdown unspecified - mixed	USA	Instrumental music education	8 months	70 min, weekly	Mathematics (correlation)	Meta-analysis		Low
McCarthy	1992		Unclear (correlational)	1061 college students, age & sex unavailable	USA	Orchestra	At least 2 years college music instruction	Unspecified	Mathematics (correlation)	Meta-analysis		Low
Neufeld	1986	No	Non-randomized experimental cohort study	80 children (kindergarten & pre-k), sex unavailable	USA	Kodaly Music education	1-2 years	Unspecified	Mathematics (causation - experimental)	Meta-analysis		Low
Weeden	1971	No	Non-randomized experimental cohort study	47 1st & 2nd graders, sex unspecified	USA	Suzuki violin education	4 months	2x/week	Mathematics (causation - experimental)	Meta-analysis		Low
Wheeler & Wheeler	1951	No	Cross-sectional	1969 college students, age & sex unspecified	USA	College music majors	At least 2 years college music instruction	Unspecified	Mathematics (correlation)	Meta-analysis		Low
Yap, Kwan & Ang, 2017												
Bittman	2004	Yes (Remo & Yamaha)	Nonrandomized crossover experimental study	75 nursing students (mean age 27.5; 64 women, 11 men)	USA	Group drumming	1 hour	6 weeks	Mood	Positive	Significant pre-post improvement in overall mood state (POMS), changes persisted for 6 weeks post-intervention	Very Low
									Emotional exhaustion	Positive	Significant pre-post reduction in emotional exhaustion (Malasch Burnout Inventory), changes persisted 6 weeks post-intervention	Very Low
									Depersonalization	NO change	No significant pre-post changes in feelings of depersonalization	Very Low
									Personal accomplishment	No change	No significant pre-post changes in feelings of personal accomplishment	Very Low
Dunbar et al	2012	No	Nonrandomized experimental study	32 adults (12 from samba drumming school (mean age 44.2; mean 4.25 years samba drumming	UK	Drumming (samba)	30 minutes	Once	Positive affect	Positive	Positive effect of samba drumming on positive affect vs control groups(Positive and Negative Affect Schedule (PANAS))	Low

				experience); 9 employees from city music shop (control 1; mean age 31.1); 11 Master's students (control 2 (watched video of speech), mean age 24.6), 17 male/15 female					Negative affect	No effect	No effect of samba drumming vs control groups (Positive and Negative Affect Schedule (PANAS))	Low
Ho et al	2011	No	Nonrandomized intervention study	101 5th graders (mean age ~10.5; 54 girls, 47 boys)	USA	Group drumming	40-45 min	12 weeks	Social functioning	Positive	Significant improvement in social function in group drumming groups vs control (Teacher's Report Form)	Low
Koyama et al.	2009	Other review - Fancourt, Ockelford & Belai, 2014										
Mungas & Silverman	2014	No	Nonrandomized experimental study	50 university students (17 experimental group (mean age = 20.8); 33 control group (mean age = 21.5)), 35 female/15 male	USA	Group drumming	45 min - 1 hour	Once	Drowsiness	Positive	Significantly reduced drowsiness pre-post (Quick Mood Scale)	Very Low
									Anxiety	Positive	Significantly reduced anxiety pre-post (Quick Mood Scale)	Very Low
									Depression	Positive	Significantly reduced depression pre-post (Quick Mood Scale)	Very Low
									Aggression	Positive	Significantly reduced aggression pre-post (Quick Mood Scale)	Very Low
									Confusion	Positive	Significantly reduced confusion pre-post (Quick Mood Scale)	Very Low
									Lack of coordination	No effect	NO significant pre-post effect on lack of coordination (Quick Mood Scale)	Very Low
Shimzu et al.	2013	Excluded - 'music movement therapy' (exercise program incorporating the 'Naruko clapper'; designed as exercise, not music-making)										
Smith, Viljoen & McGeachie	2014	No	Single group experimental study (pre-post analysis)	17 young adult novice drummers (mean age 23.4; 13 women, 4 men)	South Africa	Drumming (djembe)	40 minutes	Once	Blood pressure	No effect	No significant pre-post changes in systolic or diastolic blood pressure	Very Low
				17 middle aged experienced drummers (mean					Blood pressure	Positive	Significant pre-post decrease in systolic blood pressure; no pre-	Very Low

					age 53.1; 12 women, 5 men)							post change in diastolic blood pressure	
					17 young adult novice drummers (mean age 23.4; 13 women, 4 men)					Stress/anxiety	Positive	Significant pre-post decrease in stress/anxiety (stress anxiety index (SAI))	Very Low
					17 middle aged experienced drummers (mean age 53.1; 12 women, 5 men)					Stress/anxiety	Positive	Significant pre-post decrease in stress/anxiety (stress anxiety index (SAI))	Very Low
Snow & D'Amico	2010	Excluded - qualitative results only											
Wachi et al.	2007		Yes (Yamaha Corp)	RCT	40 healthy males (mean age 38.4)	Japan	Group drumming	60 minutes	Once	Mood disturbance	Positive	(Profile of Mood States (POMS))	High
										Natural killer cell activity (effector:target 25:1)	Negative	Significant decrease in NK cell activity vs control	High
										Natural killer cell activity (effector:target 12:1)	Negative	Significant decrease in NK cell activity pre-post & vs control	High
										Natural killer cell activity	Positive	Modulation of NK cell activity towards 'mid-range' pre-post drumming activity (i.e. individuals with low NK cell levels up regulated post-drumming, individuals with high NK cell activity down regulated post-drumming)	High
										CD-56+ cells	No effect	No effect of group drumming pre-post or vs control	High
										IFN-gamma gene expression (mRNA)	Positive	Significant decrease in IFN-alpha gene expression vs control (anti-inflammatory response)	High

IL-2 gene expression (mRNA)	NO effect	No effect of group drumming on IL-2 gene expression (pre-post or vs control)	High
IL-6 gene expression (mRNA)	No effect	No effect of group drumming on IL-6 gene expression (pre-post or vs control)	High
IL-10 gene expression (mRNA)	Positive	Significant increase in IL-10 gene expression pre-post group drumming and vs control	High
Leukocyte counts	No effect	No effect of group drumming on leukocyte counts vs control (increased pre-post in both groups)	High

Table S9. GRADE Certainty of Evidence ratings for each outcome measure from individual randomized controlled trials (RCTs) of dance participation contained in included reviews.

Author	*Year*	*Outcome*	***GRADE Initial quality***	*Concealed allocation*	*Blinded assessors/ data analysts*	*Low % lost to follow up*	*Intention to treat analysis*	*All outcome result(s) reported*	*Validated outcome measures used*	*Used individual randomization (not cluster randomization)*	*Carryover effects controlled (cross-over trial)*	***GRADE Bias risk***	***GRADE Indirectness***	***GRADE up rating***	***GRADE Certainty of Evidence***
Burkhardt & Brennan, 2012															
Burgess, Grogan & Burwitz	2006	Self-perception	High	Yes	Unspecified	Yes	Yes	Yes	Yes	Yes	No	Serious (immediate transition between dance & exercise groups in previously inactive girls)	None	None	Moderate
Flores	1995	Timed mile run	High	Yes	Unspecified	Yes	Yes	Yes	Yes	No (classes randomized, not individuals)	N/A	Serious (cluster randomization by classroom)	None	None	Moderate
		BMI	High	Yes	Unspecified	Yes	Yes	Yes	Yes	No (classes randomized, not individuals)	N/A	Serious (cluster randomization by classroom)	None	None	Moderate
Kim & Kim	2007	Mood	High	Questionable (all female hip-hop dance group, but sex mix in other groups)	N/A (questionnaire)	Yes	Yes	Yes	Yes	Yes	Yes	Serious (questionable blinding of allocation)	None	None	Moderate
Robinson et al	2003	BMI	High	No (family members allowed to group together)	Yes	Yes	Yes	No (no reporting of analyses of changes in blood lipid/glucose/ insulin markers)	Yes	Yes	N/A	Very serious (family members permitted to circumvent randomization to be in same group as other family members; analysis admittedly underpowered to detect between group differences)	None	None	Low
		Waist circumference	High	No (family members allowed to	Yes	Yes	Yes	No (no reporting of analyses of changes in blood	Yes	Yes	N/A	Very serious (family members permitted to circumvent	None	None	Low

				group together)				lipid/glucose/ insulin markers)				randomization to be in same group as other family members; analysis admittedly underpowered to detect between group differences)			
		Self-esteem	High	No (family members allowed to group together)	Yes	Yes	Yes	No (no reporting of analyses of changes in blood lipid/glucose/ insulin markers)	Yes	Yes	N/A	Very serious (family members permitted to circumvent randomization to be in same group as other family members; analysis admittedly underpowered to detect between group differences)	None	None	Low
Clarke et al., 2018															
Costa, Ferreira & Felicio, 2013															
Cugusi et al, 2019															
Delextrat et al.	2016	VO2 max	High	Yes	Unspecified	Yes	Yes	Yes	Yes	Yes	N/A	Minimal	None	None	High
		% fat mass	High	Yes	Unspecified	Yes	Yes	Yes	Yes	Yes	N/A	Minimal	None	None	High
		Body mass	High	Yes	Unspecified	Yes	Yes	Yes	Yes	Yes	N/A	Minimal	None	None	High
Domene et al	2016	Blood pressure	High	Yes	Unspecified	Yes	Yes	Yes	No (valid outcomes, but only pre-post analysis of results; no statistical comparison vs control)	Yes	N/A	Very serious (pre-post analyses only; no statistical comparison between dance & control groups – effectively a	None	None	Low

										single group study)			
Body mass	High	Yes	Unspecified	Yes	Yes	Yes	No (valid outcomes, but only pre-post analysis of results; no statistical comparison vs control)	Yes	N/A	Very serious (pre-post analyses only; no statistical comparison between dance & control groups – effectively a single group study)	None	None	Low
% fat mass	High	Yes	Unspecified	Yes	Yes	Yes	No (valid outcomes, but only pre-post analysis of results; no statistical comparison vs control)	Yes	N/A	Very serious (pre-post analyses only; no statistical comparison between dance & control groups – effectively a single group study)	None	None	Low
White blood cell count	High	Yes	Unspecified	Yes	Yes	Yes	No (valid outcomes, but only pre-post analysis of results; no statistical comparison vs control)	Yes	N/A	Very serious (pre-post analyses only; no statistical comparison between dance & control groups – effectively a single group study)	None	None	Low
CRP	High	Yes	Unspecified	Yes	Yes	Yes	No (valid outcomes, but only pre-post analysis of results; no statistical comparison vs control)	Yes	N/A	Very serious (pre-post analyses only; no statistical comparison between dance & control groups – effectively a single group study)	None	None	Low
IL-6	High	Yes	Unspecified	Yes	Yes	Yes	No (valid outcomes,	Yes	N/A	Very serious (pre-post	None	None	Low

								but only pre-post analysis of results; no statistical comparison vs control)			analyses only; no statistical comparison between dance & control groups – effectively a single group study)			
		Total cholesterol to HDL ratio	High	Yes	Unspecified	Yes	Yes	Yes	No (valid outcomes, but only pre-post analysis of results; no statistical comparison vs control)	Yes	N/A	Very serious (pre-post analyses only; no statistical comparison between dance & control groups – effectively a single group study)	None	None	Low
		VO2 max	High	Yes	Unspecified	Yes	Yes	Yes	No (valid outcomes, but only pre-post analysis of results; no statistical comparison vs control)	Yes	N/A	Very serious (pre-post analyses only; no statistical comparison between dance & control groups – effectively a single group study)	None	None	Low
		BMI	High	Yes	Unspecified	Yes	Yes	Yes	No (valid outcomes, but only pre-post analysis of results; no statistical comparison vs control)	Yes	N/A	Very serious (pre-post analyses only; no statistical comparison between dance & control groups – effectively a single group study)	None	None	Low
Donath et al	2014	6-minute walk test	High	Yes	Unspecified	Yes	Yes	Yes	Yes	Yes	N/A	Minimal	None	None	High
		BMI	High	Yes	Unspecified	Yes	Yes	Yes	Yes	Yes	N/A	Minimal	None	None	High
Fernandez-Arguelles et al., 2015														

Fong Yan et al., 2018															
Barene et al.	2016	Sit & Reach	High	Yes	Unspecified	Yes	Yes	Yes	Yes	No (cluster randomization)	N/A	Serious (cluster randomization + unclear blinding of assessors)	None	None	Moderate
		Postural stability	High	Yes	Unspecified	Yes	Yes	Yes	Yes	No (cluster randomization)	N/A	Serious (cluster randomization + unclear blinding of assessors)	None	None	Moderate
Barene et al.	2014	Fat mass	High	Yes	Unspecified	Yes	Yes	Yes	Yes	No (cluster randomization)	N/A	Serious (cluster randomization + unclear blinding of assessors)	None	None	Moderate
		VO2 max	High	Yes	Unspecified	Yes	Yes	Yes	Yes	No (cluster randomization)	N/A	Serious (cluster randomization + unclear blinding of assessors)	None	None	Moderate
		Triglycerides	High	Yes	Unspecified	Yes	Yes	Yes	Yes	No (cluster randomization)	N/A	Serious (cluster randomization + unclear blinding of assessors)	None	None	Moderate
		Glucose	High	Yes	Unspecified	Yes	Yes	Yes	Yes	No (cluster randomization)	N/A	Serious (cluster randomization + unclear blinding of assessors)	None	None	Moderate
		Peak ventilation	High	Yes	Unspecified	Yes	Yes	Yes	Yes	No (cluster randomization)	N/A	Serious (cluster randomization + unclear blinding of assessors)	None	None	Moderate
		Blood pressure	High	Yes	Unspecified	Yes	Yes	Yes	Yes	No (cluster randomization)	N/A	Serious (cluster randomization + unclear blinding of assessors)	None	None	Moderate
		Respiratory exchange ratio	High	Yes	Unspecified	Yes	Yes	Yes	Yes	No (cluster randomization)	N/A	Serious (cluster randomization + unclear blinding of assessors)	None	None	Moderate
		Total cholesterol	High	Yes	Unspecified	Yes	Yes	Yes	Yes	No (cluster randomization)	N/A	Serious (cluster randomization + unclear blinding of assessors)	None	None	Moderate
		Bone mineral density	High	Yes	Unspecified	Yes	Yes	Yes	Yes	No (cluster randomization)	N/A	Serious (cluster randomization +	None	None	Moderate

											unclear blinding of assessors)			
		HDL cholesterol	High	Yes	Unspecified	Yes	Yes	Yes	Yes	No (cluster randomization)	N/A	Serious (cluster randomization + unclear blinding of assessors)	None	None	Moderate
		Body mass	High	Yes	Unspecified	Yes	Yes	Yes	Yes	No (cluster randomization)	N/A	Serious (cluster randomization + unclear blinding of assessors)	None	None	Moderate
		BMI	High	Yes	Unspecified	Yes	Yes	Yes	Yes	No (cluster randomization)	N/A	Serious (cluster randomization + unclear blinding of assessors)	None	None	Moderate
Belardinelli et al.	2008	VO2 max	High	Yes	Unspecified	Yes	Yes	Yes	Yes	Yes	N/A	Minimal	None	None	High
		Triglycerides	High	Yes	Unspecified	Yes	Yes	Yes	Yes	Yes	N/A	Minimal	None	None	High
		Total cholesterol	High	Yes	Unspecified	Yes	Yes	Yes	Yes	Yes	N/A	Minimal	None	None	High
		HDL cholesterol	High	Yes	Unspecified	Yes	Yes	Yes	Yes	Yes	N/A	Minimal	None	None	High
Burgess, Grogan & Burwitz	2006	BMI	High	Yes	Unspecified	Yes	Yes	Yes	Yes	Yes	No	Serious (immediate transition between dance & exercise groups in previously inactive girls)	None	None	Moderate
Garber, McKinney & Carleton	1992	VO2 max	High	Yes	Unspecified	Yes	No (analyses only performed on those with 85%+ adherence)	Yes	Yes	Yes	N/A	Serious (analysis only on participants with >85% adherence)	None	None	Moderate
		Peak ventilation	High	Yes	Unspecified	Yes	No (analyses only performed on those with 85%+ adherence)	Yes	Yes	Yes	N/A	Serious (analysis only on participants with >85% adherence)	None	None	Moderate
		Respiratory exchange ratio	High	Yes	Unspecified	Yes	No (analyses only performed on those with	Yes	Yes	Yes	N/A	Serious (analysis only on participants with >85% adherence)	None	None	Moderate

							85%+ adherence)								
		Body mass	High	Yes	Unspecified	Yes	No (analyses only performed on those with 85%+ adherence)	Yes	Yes	Yes	N/A	Serious (analysis only on participants with >85% adherence)	None	None	Moderate
		BMI	High	Yes	Unspecified	Yes	No (analyses only performed on those with 85%+ adherence)	Yes	Yes	Yes	N/A	Serious (analysis only on participants with >85% adherence)	None	None	Moderate
Hackney et al.	2007	Timed up and go	High	Yes	Yes	Yes	Yes	Yes	Yes	Yes	N/A	Minimal	None	None	High
		Berg balance test	High	Yes	Yes	Yes	Yes	Yes	Yes	Yes	N/A	Minimal	None	None	High
Hashimoto et al.	2015	Timed up and go	High	Yes	Yes	Yes	Yes	Yes	Yes	Yes	N/A	Minimal	None	None	High
		Berg balance test	High	Yes	Yes	Yes	Yes	Yes	Yes	Yes	N/A	Minimal	None	None	High
Janyacharoen et al	2013	Sit & Reach	High	Yes	Yes	Yes (4/42)	Yes	Yes	Yes	Yes	N/A	Minimal	None	None	High
Kalsatou et al.	2014	VO2 max	High	Yes	Yes	Yes	Yes	Yes	Yes	Yes	N/A	Minimal	None	None	High
		Peak ventilation	High	Yes	Yes	Yes	Yes	Yes	Yes	Yes	N/A	Minimal	None	None	High
		Berg Balance test	High	Yes	Yes	Yes	Yes	Yes	Yes	Yes	N/A	Minimal	None	None	High
		Body mass	High	Yes	Yes	Yes	Yes	Yes	Yes	Yes	N/A	Minimal	None	None	High
		BMI	High	Yes	Yes	Yes	Yes	Yes	Yes	Yes	N/A	Minimal	None	None	High
Kin Isler, Kosar & Korkusuz	2001	Triglycerides	High	Yes	Unspecified	Yes	Yes	Yes	Yes	Yes	N/A	Minimal	None	None	High
		Body weight	High	Yes	Unspecified	Yes	Yes	Yes	Yes	Yes	N/A	Minimal	None	None	High
		Total cholesterol	High	Yes	Unspecified	Yes	Yes	Yes	Yes	Yes	N/A	Minimal	None	None	High
		HDL cholesterol	High	Yes	Unspecified	Yes	Yes	Yes	Yes	Yes	N/A	Minimal	None	None	High
Rani & Singh	2013	Sit & reach	High	Yes	Unspecified	Unspecified	Unspecified	Yes	Yes	Yes	N/A	Serious (unclear reporting re. % lost to follow up / intent to treat analysis)	None	None	Moderate

		Sit ups	High	Yes	Unspecified	Unspecified	Unspecified	Yes	Yes	Yes	N/A	Serious (unclear reporting re. % lost to follow up / intent to treat analysis)	None	None	Moderate
		1-mile walk/run	High	Yes	Unspecified	Unspecified	Unspecified	Yes	Yes	Yes	N/A	Serious (unclear reporting re. % lost to follow up / intent to treat analysis)	None	None	Moderate
		Skinfold thickness	High	Yes	Unspecified	Unspecified	Unspecified	Yes	Yes	Yes	N/A	Serious (unclear reporting re. % lost to follow up / intent to treat analysis)	None	None	Moderate
Rios Romenets et al	2015	Timed up and go	High	Yes	Unspecified	Yes	Yes	Yes	Yes	Yes	N/A	Minimal	None	None	High
Sofianidis, Dimitriou & Hatzitaki	2017	Postural stability	High	Yes	Unspecified	Yes	Yes	Yes	Yes	Yes	N/A	Minimal	None	None	High
Volpe et al.	2013	Timed up and go	High	Yes	Yes	Yes	Yes	Yes	Yes	Yes	N/A	Minimal	None	None	High
		Berg balance test	High	Yes	Yes	Yes	Yes	Yes	Yes	Yes	N/A	Minimal	None	None	High
Hwang & Braun, 2015															
Marmeleira et al	2009	Proprioception	High	Yes	Unspecified	Yes	Yes	Yes	Yes	Yes	N/A	Minimal	None	None	High
Keogh et al., 2009															
Letton, Thom, & Ward, 2020															
Liu, Shen & Tsai, 2020															
Bennett & Hackney	2017	Balance	High	Yes	No	Yes	Yes	Yes	Yes	No (block randomization)	N/A	Serious (potential selection bias due to block randomization)	None	None	Moderate
		Gait speed	High	Yes	No	Yes	Yes	Yes	Yes	No (block randomization)	N/A	Serious (potential selection bias due	None	None	Moderate

												to block randomization)			
Cepeda et al	2015	Endurance	High	Yes	Unspecified	Yes	Yes	Yes	Yes	Yes	N/A	Minimal	None	None	High
		Mobility	High	Yes	Unspecified	Yes	Yes	Yes	Yes	Yes	N/A	Minimal	None	None	High
Eyigor et al.	2009	Balance	High	Yes	Yes	Yes (3/40 lost to follow up)	Yes	Yes	Yes	Yes	N/A	Minimal	None	None	High
		Endurance	High	Yes	Yes	Yes (3/40 lost to follow up)	Yes	Yes	Yes	Yes	N/A	Minimal	None	None	High
		Mobility	High	Yes	Yes	Yes (3/40 lost to follow up)	Yes	Yes	Yes	Yes	N/A	Minimal	None	None	High
		General health	High	Yes	Yes	Yes (3/40 lost to follow up)	Yes	Yes	Yes	Yes	N/A	Minimal	None	None	High
Granacher et al.	2012	Balance	High	Yes	Unspecified	Yes	Yes	Yes	Yes	Yes	N/A	Minimal	None	None	High
		Gait speed	High	Yes	Unspecified	Yes	Yes	Yes	Yes	Yes	N/A	Minimal	None	None	High
Hamacher et al.	2015	Gait speed	High	Yes	Yes	Borderline (13/48) lost to follow up)	Yes	Yes	Yes	Yes	N/A	Minimal	None	None	High
Holmerova et al.	2009	Flexibility	High	Yes	Yes	Borderline (20/72 lost to follow up)	Yes	Yes	Yes	Yes	N/A	Minimal	None	None	High
		Mobility	High	Yes	Yes	Borderline (20/72 lost to follow up)	Yes	Yes	Yes	Yes	N/A	Minimal	None	None	High
Hui, Chui & Woo	2009	Endurance	High	Yes	Yes	Yes (9/111)	Yes	Yes	Yes	No (random allocation of 2 groups)	N/A	Serious (group, rather than individual, randomization)	None	None	Moderate
		Flexibility	High	Yes	Yes	Yes (9/111)	Yes	Yes	Yes	No (random allocation of 2 groups)	N/A	Serious (group, rather than individual, randomization)	None	None	Moderate
		Mobility	High	Yes	Yes	Yes (9/111)	Yes	Yes	Yes	No (random allocation of 2 groups)	N/A	Serious (group, rather than individual, randomization)	None	None	Moderate

		General health	High	Yes	Yes	Yes (9/111)	Yes	Yes	Yes	No (random allocation of 2 groups)	N/A	Serious (group, rather than individual, randomization)	None	None	Moderate
Janyacharoen et al.	2013	Endurance	High	Yes	Yes	Yes (4/42)	Yes	Yes	Yes	Yes	N/A	Minimal	None	None	High
		Flexibility	High	Yes	Yes	Yes (4/42)	Yes	Yes	Yes	Yes	N/A	Minimal	None	None	High
		Mobility	High	Yes	Yes	Yes (4/42)	Yes	Yes	Yes	Yes	N/A	Minimal	None	None	High
Machacova et al.	2015	Flexibility	High	Yes	Yes	Borderline (20/72 lost to follow up)	Yes	Yes	Yes	Yes	N/A	Minimal	None	None	High
		Mobility	High	Yes	Yes	Borderline (20/72 lost to follow up)	Yes	Yes	Yes	Yes	N/A	Minimal	None	None	High
McKinley et al.	2008	Mobility	High	Yes	Yes	Yes	Yes	Yes	Yes	Yes	N/A	Minimal	None	None	High
		Gait speed	High	Yes	Yes	Yes	Yes	Yes	Yes	Yes	N/A	Minimal	None	None	High
Merom et al.	2016	Gait speed	High	Yes	No	Yes (~80%)	Yes	Yes	Yes	No (cluster randomized by retirement center)	N/A	Serious (cluster randomization + unblinded assessors)	None	None	Moderate
		Mobility	High	Yes	No	Yes (~80%)	Yes	Yes	Yes	No (cluster randomized by retirement center)	N/A	Serious (cluster randomization + unblinded assessors)	None	None	Moderate
Sofianidis et al.	2009	Balance	High	Yes	Unspecified	Yes	Yes	Yes	Yes	Yes	N/A	Minimal	None	None	High
Serrano-Guzman et al.	2016	Mobility	High	Yes	Yes	Yes	Yes	Yes	Yes	No (block randomized in groups of 6)	N/A	Serious (block randomization)	None	None	Moderate
Meng et al., 2020															
Alves	2013	Executive function	High	Yes	Unspecified	Yes	Yes	Yes	Yes	Yes	N/A	Minimal	None	None	High
Chen	2014	Global cognition	High	*Article in Mandarin – rating assigned based on study design*											High
Doi et al	2017	Global cognition	High	Yes	Yes	Yes	Yes	Yes	Yes	Yes	N/A	Minimal	None	None	High
		Executive function	High	Yes	Yes	Yes	Yes	Yes	Yes	Yes	N/A	Minimal	None	None	High
Kattenstroth et al	2013	Global cognition	High	Yes	Unspecified	Yes	Yes	Yes	Yes, but (validated	Yes	N/A	Serious (pre-post analyses of	None	None	Moderate

									outcome measures, but only pre-post analysis within groups; no statistical comparisons between control & intervention groups)			control & experimental groups only)			
Kosmat & Vranic	2017	Executive function	High	Yes	Unclear	Yes	Yes	Yes	Yes	Yes	N/A	Minimal	None	None	High
		Memory	High	Yes	Unclear	Yes	Yes	Yes	Yes	Yes	N/A	Minimal	None	None	High
Lazarou et al	2017	Global cognition	High	Yes	Yes	Yes	Yes	Yes	Yes	Yes	N/A	Minimal	None	None	High
		Executive function	High	Yes	Yes	Yes	Yes	Yes	Yes	Yes	N/A	Minimal	None	None	High
Merom et al	2016	Executive function	High	Yes	No	No (65-70% complete data)	Yes	Yes	Yes	Yes	N/A	Serious (high % lost to follow up; participants lost to follow up had lower baseline scores than other participants)	None	None	Moderate
		Memory	High	Yes	No	No (65-70% complete data)	Yes	Yes	Yes	Yes	N/A	Serious (high % lost to follow up; participants lost to follow up had lower baseline scores than other participants)	None	None	Moderate
Merom et al	2016	Executive function	High	Yes	No	Yes (~80%)	Yes	Yes	Yes	No (cluster randomized by retirement center)	N/A	Serious (cluster randomization + unblinded assessors)	None	None	Moderate
Zhang, Ni & Liu	2012	Global cognition	High	*Article in Mandarin – rating assigned based on study design*											High

Rodrigues-Krause et al., 2016

Rodrigues-Krause, Krause, Reischak-Oliveira, 2019

Borges et a	2012	Functional autonomy	High	Yes	Unspecified	Yes	Yes	Yes	Yes	Yes	N/A	Minimal	None	None	High
		Balance	High	Yes	Unspecified	Yes	Yes	Yes	Yes	Yes	N/A	Minimal	None	None	High
Cruz-Ferreira et al	2015	Physical Fitness	High	Yes	Unspecified	Yes	Yes	Yes	Yes	Yes	N/A	Minimal	None	None	High
		Life Satisfaction	High	Yes	Unspecified	Yes	Yes	Yes	Yes	Yes	N/A	Minimal	None	None	High
Eyigor et al.	2009	Depression	High	Yes	Yes	Yes (3/40 lost to follow up)	Yes	Yes	Yes	Yes	N/A	Minimal	None	None	High
Federici et al	2005	Balance	High	Yes	Unspecified	Yes	Yes	Yes	Yes	Yes	N/A	Minimal	None	None	High
		Smoking	High	Yes	Unspecified	Yes	Yes	Yes	No	Yes	N/A	Serious (unvalidated assessment)	None	None	Moderate
		Alcohol Consumption	High	Yes	Unspecified	Yes	Yes	Yes	No	Yes	N/A	Serious (unvalidated assessment)	None	None	Moderate
		Sexual activity	High	Yes	Unspecified	Yes	Yes	Yes	No	Yes	N/A	Serious (unvalidated assessment)	None	None	Moderate
		Sleep quality	High	Yes	Unspecified	Yes	Yes	Yes	No	Yes	N/A	Serious (unvalidated assessment)	None	None	Moderate
Granacher et al	2012	Muscular Power (lower leg)	High	Yes	Unspecified	Yes	Yes	Yes	Yes	Yes	N/A	Minimal	None	None	High
Hui et al	2009	Strength	High	Yes	Yes	Yes (9/111 lost)	Yes	Yes	Yes	No (random allocation of 2 groups)	N/A	Serious (group, rather than individual, randomization)	None	None	Moderate
		Balance	High	Yes	Yes	Yes (9/111 lost)	Yes	Yes	Yes	No (random allocation of 2 groups)	N/A	Serious (group, rather than individual, randomization)	None	None	Moderate
		Body composition	High	Yes	Yes	Yes (9/111 lost)	Yes	Yes	Yes	No (random allocation of 2 groups)	N/A	Serious (group, rather than individual, randomization)	None	None	Moderate
Lesser et al	2016	Body composition	High	Yes	Yes (blinded assessors, data analysts unclear)	Yes	Yes	Yes	Yes	Yes	N/A	Minimal	None	None	High
		Glucose	High	Yes	Yes (blinded assessors, data	Yes	Yes	Yes	Yes	Yes	N/A	Minimal	None	None	High

					analysts unclear)										
		Insulin	High	Yes	Yes (blinded assessors, data analysts unclear)	Yes	Yes	Yes	Yes	Yes	N/A	Minimal	None	None	High
		Aerobic fitness	High	Yes	Yes (blinded assessors, data analysts unclear)	Yes	Yes	Yes	Yes	Yes	N/A	Minimal	None	None	High
McKinley et al	2008	Balance confidence	High	Yes	Yes	Yes	Yes	Yes	Yes	Yes	N/A	Minimal	None	None	High
Wu et al	2016	Body composition	High	Yes	Unspecified	Yes	Yes	Yes	Yes	Yes	N/A	Minimal	None	None	High
		Cholesterol	High	Yes	Unspecified	Yes	Yes	Yes	Yes	Yes	N/A	Minimal	None	None	High
		Triglycerides	High	Yes	Unspecified	Yes	Yes	Yes	Yes	Yes	N/A	Minimal	None	None	High
		Glucose	High	Yes	Unspecified	Yes	Yes	Yes	Yes	Yes	N/A	Minimal	None	None	High
		Bone density	High	Yes	Unspecified	Yes	Yes	Yes	Yes	Yes	N/A	Minimal	None	None	High
		Flexibility	High	Yes	Unspecified	Yes	Yes	Yes	Yes	Yes	N/A	Minimal	None	None	High
		Strength	High	Yes	Unspecified	Yes	Yes	Yes	Yes	Yes	N/A	Minimal	None	None	High
Teixeira-Machado, Arida & de Jesus Mari, 2019															
Baniqued et al.	2018	Cardio-respiratory fitness	High	Yes	Unspecified	Yes	No (inclusion criteria revised from MMSE = 23 to MMSE = 27 for this analysis)	Yes	Yes	Yes	N/A	Minimal (higher cognitive performance unlikely to impact certainty of general outcome classifications in umbrella review)	None	None	High
		Executive function	High	Yes	Unspecified	Yes	No (inclusion criteria revised from MMSE = 23 to MMSE = 27 for this analysis)	Yes	Yes	Yes	N/A	Minimal (higher cognitive performance unlikely to impact certainty of general outcome classifications in umbrella review)	None	None	High
		Perceptual speed	High	Yes	Unspecified	Yes	No (inclusion	Yes	Yes	Yes	N/A	Minimal (higher cognitive	None	None	High

							criteria revised from MMSE = 23 to MMSE = 27 for this analysis)					performance unlikely to impact certainty of general outcome classifications in umbrella review)			
		Episodic memory	High	Yes	Unspecified	Yes	No (inclusion criteria revised from MMSE = 23 to MMSE = 27 for this analysis)	Yes	Yes	Yes	N/A	Minimal (higher cognitive performance unlikely to impact certainty of general outcome classifications in umbrella review)	None	None	High
		Vocabulary	High	Yes	Unspecified	Yes	No (inclusion criteria revised from MMSE = 23 to MMSE = 27 for this analysis)	Yes	Yes	Yes	N/A	Minimal (higher cognitive performance unlikely to impact certainty of general outcome classifications in umbrella review)	None	None	High
Ehlers et al.	2017	Loneliness	High	Yes	Unspecified	Yes	Yes	Yes	Yes (validated measure, although no inactive control; comparison vs exercise only)	Yes	N/A	Serious (no inactive control reported)	None	None	Moderate
		Social Support	High	Yes	Unspecified	Yes	Yes	Yes	Yes (validated measure, although no inactive control; comparison vs exercise only)	Yes	N/A	Serious (no inactive control reported)	None	None	Moderate

		Stress	High	Yes	Unspecified	Yes	Yes	Yes	Yes (validated measure, although no inactive control; comparison vs exercise only)	Yes	N/A	Serious (no inactive control reported)	None	None	Moderate
Kattenstroth et al.	2013	Lifestyle/ quality of life	High	Yes	Unspecified	Yes	Yes	Yes	Yes, but (validated outcome measures, but only pre-post analysis within groups; no comparisons between control & intervention groups)	Yes	N/A	Very Serious (pre-post analyses of control & experimental groups only, effectively a single group study)	None	None	Low
		Attention	High	Yes	Unspecified	Yes	Yes	Yes	Yes, but (validated outcome measures, but only pre-post analysis within groups; no comparisons between control & intervention groups)	Yes	N/A	Very Serious (pre-post analyses of control & experimental groups only, effectively a single group study)	None	None	Low
		Intelligence (nonverbal)	High	Yes	Unspecified	Yes	Yes	Yes	Yes, but (validated outcome measures, but only pre-post analysis within groups; no comparisons	Yes	N/A	Very Serious (pre-post analyses of control & experimental groups only, effectively a single group study)	None	None	Low

							between control & intervention groups)						
Fluid intelligence	High	Yes	Unspecified	Yes	Yes	Yes	Yes, but (validated outcome measures, but only pre-post analysis within groups; no comparisons between control & intervention groups)	Yes	N/A	Very Serious (pre-post analyses of control & experimental groups only, effectively a single group study)	None	None	Low
Reaction time	High	Yes	Unspecified	Yes	Yes	Yes	Yes, but (validated outcome measures, but only pre-post analysis within groups; no comparisons between control & intervention groups)	Yes	N/A	Very Serious (pre-post analyses of control & experimental groups only, effectively a single group study)	None	None	Low
Balance	High	Yes	Unspecified	Yes	Yes	Yes	Yes, but (validated outcome measures, but only pre-post analysis within groups; no comparisons between control & intervention groups)	Yes	N/A	Very Serious (pre-post analyses of control & experimental groups only, effectively a single group study)	None	None	Low

		Fine-motor performance	High	Yes	Unspecified	Yes	Yes	Yes	Yes, but (validated outcome measures, but only pre-post analysis within groups; no comparisons between control & intervention groups)	Yes	N/A	Very Serious (pre-post analyses of control & experimental groups only, effectively a single group study)	None	None	Low
		Tactile performance	High	Yes	Unspecified	Yes	Yes	Yes	Yes, but (validated outcome measures, but only pre-post analysis within groups; no comparisons between control & intervention groups)	Yes	N/A	Very Serious (pre-post analyses of control & experimental groups only, effectively a single group study)	None	None	Low
		Cardio-respiratory fitness	High	Yes	Unspecified	Yes	Yes	Yes	Yes, but (validated outcome measures, but only pre-post analysis within groups; no comparisons between control & intervention groups)	Yes	N/A	Very Serious (pre-post analyses of control & experimental groups only, effectively a single group study)	None	None	Low
Muller et al.	2017	Cardiovascular fitness	High	Yes	Unspecified	No (52 enrolled, final analysis on 22 subjects)	No (analyses only performed on those with	Yes	Yes	Yes	N/A	Serious (high % lost to follow up, no intention-to-treat analysis)	None	None	Moderate

							70%+ participation)								
		Memory	High	Yes	Unspecified	No (52 enrolled, final analysis on 22 subjects)	No (analyses only performed on those with 70%+ participation)	No (some neuropsychological tests excluded from reporting for unspecified rationale)	Yes	Yes	N/A	Very serious (high % lost to follow up, incomplete outcome reporting, no intention-to-treat analysis)	None	None	Low
		Attention	High	Yes	Unspecified	No (52 enrolled, final analysis on 22 subjects)	No (analyses only performed on those with 70%+ participation)	No (some neuropsychological tests excluded from reporting for unspecified rationale)	Yes	Yes	N/A	Very serious (high % lost to follow up, incomplete outcome reporting, no intention-to-treat analysis)	None	None	Low
		BDNF	High	Yes	Unspecified	No (52 enrolled, final analysis on 22 subjects)	No (analyses only performed on those with 70%+ participation)	Yes	Yes	Yes	N/A	Serious (high % lost to follow up, no intention-to-treat analysis)	None	None	Moderate
		Cognitive structure	High	Yes	Unspecified	No (52 enrolled, final analysis on 22 subjects)	No (analyses only performed on those with 70%+ participation)	Yes	Yes	Yes	N/A	Serious (high % lost to follow up, no intention-to-treat analysis)	None	None	Moderate
Rehfeld et al.	2017	Balance	High	Yes	Unspecified	No (52 enrolled, final analysis on 26 subjects)	No (analyses only performed on those with 70%+ participation)	Yes	Yes	Yes	N/A	Serious (high % lost to follow up, no intention-to-treat analysis)	None	None	Moderate
		Hippocampal grey matter volume	High	Yes	Unspecified	No (52 enrolled, final analysis on 26 subjects)	No (analyses only performed on those with 70%+ participation)	Yes	Yes	Yes	N/A	Serious (high % lost to follow up, no intention-to-treat analysis)	None	None	Moderate
Veronese et al., 2017															

da Silva Borges	2014	Falls	High	Yes	Unspecified	Yes	Yes	Yes	Yes	Yes	N/A	Minimal	None	None	High
Pichierri, Murer & de Bruin	2012	Fear of falling	High	Yes	No	Borderline (9/31 lost)	No (analysis only performed on individuals who completed >75% of intervention)	Yes	Yes	Yes	N/A	Serious (no intention to treat analysis + moderate % lost to follow up)	None	None	Moderate
Merom et al.	2016	Falls	High	Yes	No	Yes (~80%)	Yes	Yes	Yes	No (cluster randomized by retirement center)	N/A	Minimal	None	None	High
		Falls risk	High	Yes	No	Yes (~80%)	Yes	Yes	Yes	No (cluster randomized by retirement center)	N/A	Minimal	None	None	High
Wu et al.	2016	Fear of falling	High	Yes	Unspecified	Yes	Yes	Yes	Yes	Yes	N/A	Minimal	None	None	High
		Falls	High	Yes	Unspecified	Yes	Yes	Yes	Yes	Yes	N/A	Minimal	None	None	High

Table S10. GRADE Certainty of Evidence ratings for each outcome measure from individual non-randomized studies of dance participation contained in included reviews.

Author	Year	Outcome	**GRADE Initial quality**	*Eligibility criteria developed and appropriately applied*	*Exposed/unexposed sourced from a common population?*	*Appropriate & rigorous measurement of exposure & outcome*	*Identical surveillance for outcome in exposed & unexposed*	*Known confounders adequately measured, controlled & adjusted for?*	*Follow up period complete*	**GRADE Bias risk**	**GRADE Indirectness**	**GRADE up rating**	**GRADE Certainty of Evidence**
**Burkhardt & Brennan, 2012**													
Adiputra et al	1996	VO2 max	Low	Yes	Yes	Yes	Yes	Yes	Yes	Minimal	None	None	Low
Bennell et al	2000	Bone mineral density	Low	Yes	Yes	Yes	N/A (cross-sectional)	Yes	N/A (cross-sectional)	Minimal	None	None	Low
Blackman et al	1988	VO2 max	Low	Yes	Yes	No (pre-post analyses and comparison of post-test values only; no analysis of change in parameters in dance vs control groups)	Yes	Yes	Yes	Serious (pre-post analyses & post-test comparisons between dance & control groups only)	None	None	Very Low
		Sit and reach	Low	Yes	Yes	No (pre-post analyses and comparison of post-test values only; no analysis of change in parameters in dance vs control groups)	Yes	Yes	Yes	Serious (pre-post analyses & post-test comparisons between dance & control groups only)	None	None	Very Low
		Strength	Low	Yes	Yes	No (pre-post analyses and comparison of post-test values only; no analysis of change in parameters in dance vs control groups)	Yes	Yes	Yes	Serious (pre-post analyses & post-test comparisons between dance & control groups only)	None	None	Very Low
		% body fat	Low	Yes	Yes	No (pre-post analyses and comparison of post-test values only; no analysis of change in parameters in dance vs control groups)	Yes	Yes	Yes	Serious (pre-post analyses & post-test comparisons between dance & control groups only)	None	None	Very Low
		Self esteem	Low	Yes	Yes	No (pre-post analyses and comparison of post-test values only; no analysis of change in parameters in dance vs control groups)	Yes	Yes	Yes	Serious (pre-post analyses & post-test comparisons between dance & control groups only)	None	None	Very Low
		Body satisfaction (Cathexis)	Low	Yes	Yes	No (pre-post analyses and comparison of post-test values only; no analysis of change in parameters in dance vs control groups)	Yes	Yes	Yes	Serious (pre-post analyses & post-test comparisons between dance & control groups only)	None	None	Very Low
		Blood Pressure	Low	Yes	Yes	No (pre-post analyses and comparison of post-test values only; no analysis of	Yes	Yes	Yes	Serious (pre-post analyses & post-test comparisons between	None	None	Very Low

						change in parameters in dance vs control groups)				dance & control groups only)			
		Body mass	Low	Yes	Yes	No (pre-post analyses and comparison of post-test values only; no analysis of change in parameters in dance vs control groups)	Yes	Yes	Yes	Serious (pre-post analyses & post-test comparisons between dance & control groups only)	None	None	Very Low
Daley & Buchanan	1999	Self-perception	Low	Yes	Yes	Yes	Yes	Yes	Yes	Minimal	None	None	Low
Leste & Rust	1990	Anxiety	Low	Yes	Yes	Yes	Yes	Yes	Yes	Minimal	None	None	Low
Matthews et al	2006	Bone mineral density	Low	Yes	Yes	Yes	Yes	Yes	Yes	Minimal	None	None	Low
Steinberg et al	2008	Musculoskeletal development	Low	Yes	Yes	Yes	N/A (cross-sectional)	Yes	N/A (cross-sectional)	Minimal	None	None	Low
Viscki-Stalec et al	2007	Core strength	Low	Yes	Yes	Yes	Yes	Yes	Yes	Minimal	None	None	Low
		Power	Low	Yes	Yes	Yes	Yes	Yes	Yes	Minimal	None	None	Low
		Agility	Low	Yes	Yes	Yes	Yes	Yes	Yes	Minimal	None	None	Low
Clarke et al., 2018													
Ambegaonkar et al.	2013	Balance	Low				*Rating extracted from review*						Low
Bronner	2012	Balance	Low				*Rating extracted from review*						Low
Casabona et al.	2016	Balance	Low				*Rating extracted from review*						Low
Crotts et al.	1996	Balance	Low				*Rating extracted from review*						Low
Gerbino, Griffin & Zurakowski	2007	Balance	Low				*Rating extracted from review*						Low
Golomer & Dupui	2000	Balance	Low				*Rating extracted from review*						Low
Golomer et al.	1999	Balance	Low				*Rating extracted from review*						Low
Golomer et al.	2009	Balance	Low				*Rating extracted from review*						Low
Golomer et al.	2010	Balance	Low				*Rating extracted from review*						Low
Golomer, Dupui & Monod	1997	Balance	Low				*Rating extracted from review*						Low
Guillou, Dupui & Golomer	2007	Balance	Low				*Rating extracted from review*						Low
Hopper et al.	2014	Balance	Low				*Rating extracted from review*						Low
Hugel et al.	1999	Balance	Low				*Rating extracted from review*						Low
Jarvis, Smith & Kulig	2014	Balance	Low				*Rating extracted from review*						Low
Kiefer et al.	2011	Balance	Low				*Rating extracted from review*						Low

Kilroy et al.	2016	Balance	Low				*Rating extracted from review*						Low
Krityakiarana & Jongkamonwiwat	2016	Balance	Low				*Rating extracted from review*						Low
Lin et al.	2011	Balance	Low				*Rating extracted from review*						Low
Lin et al.	2014	Balance	Low				*Rating extracted from review*						Low
Perez et al	2014	Balance	Low				*Rating extracted from review*						Low
Perrin et al.	2002	Balance	Low				*Rating extracted from review*						Low
Rein et al.	2011	Balance	Low				*Rating extracted from review*						Low
Schmit, Regis & Riley	2005	Balance	Low				*Rating extracted from review*						Low
Schmitt, Kuni & Sabo	2005	Balance	Low				*Rating extracted from review*						Low
Simmons	2005	Balance	Low				*Rating extracted from review*						Low
Simmons	2005	Balance	Low				*Rating extracted from review*						Low
Thullier & Moufti	2004	Balance	Low				*Rating extracted from review*						Low
Costa, Ferreira & Felicio, 2013													
Cheng et al	2011	Balance	Low	Yes	Yes	Yes	N/A (cross sectional)	Yes	N/A (cross sectional)	Minimal	None	None	Low
Gillou, Dupu & Golomer	2007	Balance	Low	Yes	Yes	Yes	N/A (cross sectional)	Yes	N/A (cross sectional)	Minimal	None	None	Low
Thiesen & Sumiya	2011	Balance	Low	Yes	Yes	Yes	N/A (cross sectional)	Yes	N/A (cross sectional)	Minimal	None	None	Low
Cugusi et al., 2019													
Araneta & Tanori	2015	Blood pressure	Low	Yes	N/A (single group study; no control group)	No (single group uncontrolled study)	N/A (single group study; no control group)	Unclear	Yes	Serious (single group study)	None	None	Very Low
		Triglycerides	Low	Yes	N/A (single group study; no control group)	No (single group uncontrolled study)	N/A (single group study; no control group)	Unclear	Yes	Serious (single group study)	None	None	Very Low
		HDL cholesterol	Low	Yes	N/A (single group study; no control group)	No (single group uncontrolled study)	N/A (single group study; no control group)	Unclear	Yes	Serious (single group study)	None	None	Very Low
		Body mass	Low	Yes	N/A (single group study; no control group)	No (single group uncontrolled study)	N/A (single group study;	Unclear	Yes	Serious (single group study)	None	None	Very Low

							no control group)						
		Glucose	Low	Yes	N/A (single group study; no control group)	No (single group uncontrolled study)	N/A (single group study; no control group)	Unclear	Yes	Serious (single group study)	None	None	Very Low
Cugusi et al	2016	Blood pressure	Low	Yes	N/A (single group study; no control group)	No (single group uncontrolled study)	N/A (single group study; no control group)	Unclear	Yes	Serious (single group study)	None	None	Very Low
		% fat mass	Low	Yes	N/A (single group study; no control group)	No (single group uncontrolled study)	N/A (single group study; no control group)	Unclear	Yes	Serious (single group study)	None	None	Very Low
		Waist circumference	Low	Yes	N/A (single group study; no control group)	No (single group uncontrolled study)	N/A (single group study; no control group)	Unclear	Yes	Serious (single group study)	None	None	Very Low
		Body mass	Low	Yes	N/A (single group study; no control group)	No (single group uncontrolled study)	N/A (single group study; no control group)	Unclear	Yes	Serious (single group study)	None	None	Very Low
		BMI	Low	Yes	N/A (single group study; no control group)	No (single group uncontrolled study)	N/A (single group study; no control group)	Unclear	Yes	Serious (single group study)	None	None	Very Low
Krishnan et al	2015	Body mass	Low	Yes	N/A (single group study; no control group)	Yes	N/A (single group study; no control group)	Yes	Somewhat (>30% lost to follow up)	Very serious (single group uncontrolled study, high % lost to follow up)	None	None	Very Low
		BMI	Low	Yes	N/A (single group study; no control group)	Yes	N/A (single group study; no control group)	Yes	Somewhat (>30% lost to follow up)	Very serious (single group uncontrolled study, high % lost to follow up)	None	None	Very Low
		% body fat	Low	Yes	N/A (single group study; no control group)	No (bioelectric impedance notably unreliable)	N/A (single group study; no control group)	Yes	Somewhat (>30% lost to follow up)	Very serious (single group uncontrolled study, high % lost to follow up, unreliable outcomes)	None	None	Very Low
		Waist circumference	Low	Yes	N/A (single group study; no control group)	Yes	N/A (single group study; no control group)	Yes	Somewhat (>30% lost to follow up)	Very serious (single group uncontrolled study, high % lost to follow up)	None	None	Very Low

		Blood pressure	Low	Yes	N/A (single group study; no control group)	Yes	N/A (single group study; no control group)	Yes	Somewhat (>30% lost to follow up)	Very serious (single group uncontrolled study, high % lost to follow up)	None	None	Very Low
		Glucose	Low	Yes	N/A (single group study; no control group)	Yes	N/A (single group study; no control group)	Yes	Somewhat (>30% lost to follow up)	Very serious (single group uncontrolled study, high % lost to follow up)	None	None	Very Low
		HbA1c	Low	Yes	N/A (single group study; no control group)	Yes	N/A (single group study; no control group)	Yes	Somewhat (>30% lost to follow up)	Very serious (single group uncontrolled study, high % lost to follow up)	None	None	Very Low
		Insulin	Low	Yes	N/A (single group study; no control group)	Yes	N/A (single group study; no control group)	Yes	Somewhat (>30% lost to follow up)	Very serious (single group uncontrolled study, high % lost to follow up)	None	None	Very Low
		Total cholesterol	Low	Yes	N/A (single group study; no control group)	Yes	N/A (single group study; no control group)	Yes	Somewhat (>30% lost to follow up)	Very serious (single group uncontrolled study, high % lost to follow up)	None	None	Very Low
		HDL cholesterol	Low	Yes	N/A (single group study; no control group)	Yes	N/A (single group study; no control group)	Yes	Somewhat (>30% lost to follow up)	Very serious (single group uncontrolled study, high % lost to follow up)	None	None	Very Low
		Triglycerides	Low	Yes	N/A (single group study; no control group)	Yes	N/A (single group study; no control group)	Yes	Somewhat (>30% lost to follow up)	Very serious (single group uncontrolled study, high % lost to follow up)	None	None	Very Low
		VO2 max	Low	Yes	N/A (single group study; no control group)	No (VO2 max based on estimate of time to completion and HR from 1-mile walk test)	N/A (single group study; no control group)	Yes	Somewhat (>30% lost to follow up)	Very serious (single group uncontrolled study, high % lost to follow up, unreliable outcomes)	None	None	Very Low
Micallef	2014	Body mass	Low	Yes	N/A (single group study; no control group)	No (single group uncontrolled study)	N/A (single group study; no control group)	No	Yes	Serious (single group study, no apparent consideration for apparent confounding variables (i.e. age))	None	None	Very Low
		BMI	Low	Yes	N/A (single group study; no control group)	No (single group uncontrolled study)	N/A (single group study; no control group)	No	Yes	Serious (single group study, no apparent consideration for apparent confounding variables (i.e. age))	None	None	Very Low

Rossmeissl et al	2016	VO2 max	Low	Yes	N/A (single group study; no control group)	Yes	N/A (single group study; no control group)	Yes	Yes	Serious (single group uncontrolled study)	None	None	Very Low
		Body mass	Low	Yes	N/A (single group study; no control group)	Yes	N/A (single group study; no control group)	Yes	Yes	Serious (single group uncontrolled study)	None	None	Very Low
		Waist circumference	Low	Yes	N/A (single group study; no control group)	Yes	N/A (single group study; no control group)	Yes	Yes	Serious (single group uncontrolled study)	None	None	Very Low
		BMI	Low	Yes	N/A (single group study; no control group)	Yes	N/A (single group study; no control group)	Yes	Yes	Serious (single group uncontrolled study)	None	None	Very Low
		Blood pressure	Low	Yes	N/A (single group study; no control group)	Yes	N/A (single group study; no control group)	Yes	Yes	Serious (single group uncontrolled study)	None	None	Very Low
		Resting HR	Low	Yes	N/A (single group study; no control group)	Yes	N/A (single group study; no control group)	Yes	Yes	Serious (single group uncontrolled study)	None	None	Very Low
		% fat mass	Low	Yes	N/A (single group study; no control group)	Yes	N/A (single group study; no control group)	Yes	Yes	Serious (single group uncontrolled study)	None	None	Very Low
Fernandez-Arguelles et al., 2015													
Fong Yan et al., 2018													
Ford et al.	1989	Sit & reach	Low	Yes	Yes	Yes	Yes	Yes	Yes	Minimal	None	None	Low
		Sit ups	Low	Yes	Yes	Yes	Yes	Yes	Yes	Minimal	None	None	Low
		Step test	Low	Yes	Yes	Yes	Yes	Yes	Yes	Minimal	None	None	Low
		Fat mass	Low	Yes	Yes	No (skin calipers not gold standard for % body fat measurement; combining with DXA measurements in meta-analysis questionable)	Yes	Yes	Yes	Serious (skin caliper measurements for % body fat)	None	None	Very Low
Heffron, Davey & Cochrane	1997	Bone mineral density	Low	Yes	Yes	Yes	Yes	Yes	Yes	Minimal	None	None	Low

Kouli et al	2009	Range of motion	Low	Yes	Yes	Yes	Yes	Yes	Yes	Minimal	None	None	Low
		Sit ups	Low	Yes	Yes	Yes	Yes	Yes	Yes	Minimal	None	None	Low
		1-mile walk/run	Low	Yes	Yes	Yes	Yes	Yes	Yes	Minimal	None	None	Low
		Sit & reach (each leg separated)	Low	Yes	Yes	Yes	Yes	Yes	Yes	Minimal	None	None	Low
		Push ups	Low	Yes	Yes	Yes	Yes	Yes	Yes	Minimal	None	None	Low
Mangeri et al.	2014	Blood pressure	Low	Yes	Yes	Yes	Yes	Yes	Yes	Minimal	None	None	Low
		Glucose	Low	Yes	Yes	Yes	Yes	Yes	Yes	Minimal	None	None	Low
		6-minute walk test	Low	Yes	Yes	Yes	Yes	Yes	Yes	Minimal	None	None	Low
		Triglycerides	Low	Yes	Yes	Yes	Yes	Yes	Yes	Minimal	None	None	Low
		Body mass	Low	Yes	Yes	Yes	Yes	Yes	Yes	Minimal	None	None	Low
		BMI	Low	Yes	Yes	Yes	Yes	Yes	Yes	Minimal	None	None	Low
		Waist circumference	Low	Yes	Yes	Yes	Yes	Yes	Yes	Minimal	None	None	Low
		Total cholesterol	Low	Yes	Yes	Yes	Yes	Yes	Yes	Minimal	None	None	Low
		HDL cholesterol	Low	Yes	Yes	Yes	Yes	Yes	Yes	Minimal	None	None	Low
Mavridis et al.	2004	Range of motion	Low	Yes	Yes	Yes	Yes	Yes	Yes	Minimal	None	None	Low
		Sit ups	Low	Yes	Yes	Yes	Yes	Yes	Yes	Minimal	None	None	Low
		1-mile walk/run	Low	Yes	Yes	Yes	Yes	Yes	Yes	Minimal	None	None	Low
		Sit & reach (each leg separated)	Low	Yes	Yes	Yes	Yes	Yes	Yes	Minimal	None	None	Low
		Push ups	Low	Yes	Yes	Yes	Yes	Yes	Yes	Minimal	None	None	Low
		Skinfold thickness	Low	Yes	Yes	Yes	Yes	Yes	Yes	Minimal	None	None	Low
Milburn & Butts	1983	VO2 max	Low	Yes	Yes	Yes	Yes	Yes	Yes	Minimal	None	None	Low
		Peak ventilation	Low	Yes	Yes	Yes	Yes	Yes	Yes	Minimal	None	None	Low
Shimamoto et al.	1998	VO2 max	Low	Yes	Yes	Yes	Yes	Yes	Yes	Minimal	None	None	Low
		VO2 at anaerobic threshold	Low	Yes	Yes	Yes	Yes	Yes	Yes	Minimal	None	None	Low
		Body mass	Low	Yes	Yes	Yes	Yes	Yes	Yes	Minimal	None	None	Low
		BMI	Low	Yes	Yes	Yes	Yes	Yes	Yes	Minimal	None	None	Low
		% fat mass	Low	Yes	Yes	Yes	Yes	Yes	Yes	Minimal	None	None	Low
		Waist circumference	Low	Yes	Yes	Yes	Yes	Yes	Yes	Minimal	None	None	Low

		Skinfold thickness	Low	Yes	Yes	Yes	Yes	Yes	Yes	Minimal	None	None	Low
Viskic et al	2007	Sit & reach	Low	Yes	Yes	Yes	Yes	Yes	Yes	Minimal	None	None	Low
		Range of motion	Low	Yes	Yes	Yes	Yes	Yes	Yes	Minimal	None	None	Low
		Step test	Low	Yes	Yes	Yes	Yes	Yes	Yes	Minimal	None	None	Low
		Body mass	Low	Yes	Yes	Yes	Yes	Yes	Yes	Minimal	None	None	Low
		% fat mass	Low	Yes	Yes	Yes	Yes	Yes	Yes	Minimal	None	None	Low
Hwang & Braun, 2015													
Ferrufino et al.	2011	Balance	Low	Yes	Yes	Yes	Yes	Yes	Yes	Minimal	None	None	Low
Keogh et al., 2009													
Kudlacek et al	1997	Bone density (peripheral and spinal)	Low	Yes	Yes	No (single group uncontrolled study)	Yes	Yes	Yes	Serious (single group uncontrolled study	None	None	Very Low
Letton, Thom, & Ward, 2020													
Hedgepeth	1987	Strength	Low	Yes	Yes	Yes	N/A (cross sectional)	Yes	N/A (cross-sectional)	Minimal	None	None	Low
		Power	Low	Yes	Yes	Yes	N/A (cross sectional)	Yes	N/A (cross-sectional)	Minimal	None	None	Low
		Endurance (muscular)	Low	Yes	Yes	Yes	N/A (cross sectional)	Yes	N/A (cross-sectional)	Minimal	None	None	Low
Kujala et al	1997	Flexibility	Low	Yes	Yes	Yes	N/A (cross sectional)	Yes	N/A (cross-sectional)	Minimal	None	None	Low
Moller & Masharawi	2011	Flexibility	Low	Yes	Yes	Yes	Yes	Yes	Yes	Minimal	None	None	Low
		Body composition	Low	Yes	Yes	Yes	Yes	Yes	Yes	Minimal	None	None	Low
		Posture	Low	Yes	Yes	Yes	Yes	Yes	Yes	Minimal	None	None	Low
Ozdinc & Turan	2016	Foot anthropometrics	Low	Yes	Yes	Yes	N/A (cross sectional)	Yes	N/A (cross-sectional)	Minimal	None	None	Low
Pigeon et al	1997	Growth	Low	Yes	Yes	Yes	No (prospective for dancers; retrospective for controls)	Yes	Yes	Serious (prospective data collection for dancers; retrospective data collection for controls	None	None	Very Low
		Puberty onset	Low	Yes	Yes	Yes	No (prospective for dancers;	Yes	Yes	Serious (prospective data collection for dancers; retrospective	None	None	Very Low

							retrospective for controls)			data collection for controls			
Liu, Shen & Tsai, 2020													
Meng et al., 2020													
Coubard et al	2011	Executive function	Low	Yes	Yes	Yes	Yes	Yes	Yes	Minimal	None	None	Low
Hackney et al	2015	Global cognition	Low	Yes	Yes	Yes	Yes	Yes	No (high % (40%) not included in final analysis due to noncompliance (<100% compliance))	Serious (high % lost to follow up)	Non	None	Very Low
		Executive function		Yes	Yes	Yes	Yes	Yes	No (high % (40%) not included in final analysis due to noncompliance (<100% compliance))	Serious (high % lost to follow up)	Non	None	Very Low
Kim et al	2011	Global cognition	Low	Yes	Yes	Yes	Yes	Yes	Yes	Minimal	None	None	Low
		Executive function	Low	Yes	Yes	Yes	Yes	Yes	Yes	Minimal	None	None	Low
Rodrigues-Krause et al., 2016													
Rodrigues-Krause, Krause & Reischak-Oliveira, 2019													
Alpert et al	2009	Depression	Low	Yes	N/A (single group study; no control group)	Yes	N/A (single group study; no control group)	Yes	Yes	Serious (single group uncontrolled study)	None	None	Very Low
		Balance	Low	Yes	N/A (single group study; no control group)	Yes	N/A (single group study; no control group)	Yes	Yes	Serious (single group uncontrolled study)	None	None	Very Low
		Cognition	Low	Yes	N/A (single group study; no control group)	Yes	N/A (single group study;	Yes	Yes	Serious (single group uncontrolled study)	None	None	Very Low

							no control group)						
Kattenstroth et al	2011	Independent living	Low	Yes	Unclear (both elderly populations but unclear where sourced)	Yes	N/A (cross-sectional)	Yes	N/A (cross-sectional)	Minimal	None	None	Low
		Cognitive performance	Low	Yes	Unclear (both elderly populations but unclear where sourced)	Yes	N/A (cross-sectional)	Yes	N/A (cross-sectional)	Minimal	None	None	Low
		Reaction time	Low	Yes	Unclear (both elderly populations but unclear where sourced)	Yes	N/A (cross-sectional)	Yes	N/A (cross-sectional)	Minimal	None	None	Low
		Balance	Low	Yes	Unclear (both elderly populations but unclear where sourced)	Yes	N/A (cross-sectional)	Yes	N/A (cross-sectional)	Minimal	None	None	Low
		Mobility	Low	Yes	Unclear (both elderly populations but unclear where sourced)	Yes	N/A (cross-sectional)	Yes	N/A (cross-sectional)	Minimal	None	None	Low
		Fine motor performance	Low	Yes	Unclear (both elderly populations but unclear where sourced)	Yes	N/A (cross-sectional)	Yes	N/A (cross-sectional)	Minimal	None	None	Low
		Tactile performance	Low	Yes	Unclear (both elderly populations but unclear where sourced)	Yes	N/A (cross-sectional)	Yes	N/A (cross-sectional)	Minimal	None	None	Low
Kattenstroth et al	2010	Independent living	Low	Yes	Unclear (both elderly populations but unclear where sourced)	Yes	N/A (cross-sectional)	Yes	N/A (cross-sectional)	Minimal	None	None	Low
		Cognitive performance	Low	Yes	Unclear (both elderly populations but unclear where sourced)	Yes	N/A (cross-sectional)	Yes	N/A (cross-sectional)	Minimal	None	None	Low
		Reaction time	Low	Yes	Unclear (both elderly populations but unclear where sourced)	Yes	N/A (cross-sectional)	Yes	N/A (cross-sectional)	Minimal	None	None	Low
		Balance	Low	Yes	Unclear (both elderly populations	Yes	N/A (cross-sectional)	Yes	N/A (cross-sectional)	Minimal	None	None	Low

					but unclear where sourced)								
		Mobility	Low	Yes	Unclear (both elderly populations but unclear where sourced)	Yes	N/A (cross-sectional)	Yes	N/A (cross-sectional)	Minimal	None	None	Low
		Fine motor performance	Low	Yes	Unclear (both elderly populations but unclear where sourced)	Yes	N/A (cross-sectional)	Yes	N/A (cross-sectional)	Minimal	None	None	Low
		Tactile performance	Low	Yes	Unclear (both elderly populations but unclear where sourced)	Yes	N/A (cross-sectional)	Yes	N/A (cross-sectional)	Minimal	None	None	Low
Krampe et al	2010	Balance	Low	Yes	N/A (single group study; no control group)	Yes	N/A (single group study; no control group)	Yes	Yes	Serious (single group uncontrolled study)	None	None	Very Low
		Mobility	Low	Yes	N/A (single group study; no control group)	Yes	N/A (single group study; no control group)	Yes	Yes	Serious (single group uncontrolled study)	None	None	Very Low
Mavrovouniotis et al	2008	Anxiety	Low	Yes	Yes (self-report questionnaires, unclear blinding of analysis)	Yes	Yes	Yes	Yes	Minimal	None	None	Low
		Psychological wellbeing	Low	Yes	Yes (self-report questionnaires, unclear blinding of analysis)	Yes	Yes	Yes	Yes	Minimal	None	None	Low
		Psychological distress	Low	Yes	Yes (self-report questionnaires, unclear blinding of analysis)	Yes	Yes	Yes	Yes	Minimal	None	None	Low
		Fatigue	Low	Yes	Yes (self-report questionnaires, unclear blinding of analysis)	Yes	Yes	Yes	Yes	Minimal	None	None	Low
Verghese	2006	General mental status	Low	Yes	Yes	Yes	N/A (cross-sectional)	Yes	N/A (cross-sectional)	Minimal	None	None	Low
		Memory (episodic)	Low	Yes	Yes	Yes	N/A (cross-sectional)	Yes	N/A (cross-sectional)	Minimal	None	None	Low
		Executive function	Low	Yes	Yes	Yes	N/A (cross-sectional)	Yes	N/A (cross-sectional)	Minimal	None	None	Low

		Depression	Low	Yes	Yes	Yes	N/A (cross-sectional)	Yes	N/A (cross-sectional)	Minimal	None	None	Low
		Gait	Low	Yes	Yes	Yes	N/A (cross-sectional)	Yes	N/A (cross-sectional)	Minimal	None	None	Low
		Balance	Low	Yes	Yes	Yes	N/A (cross-sectional)	Yes	N/A (cross-sectional)	Minimal	None	None	Low
		Strength	Low	Yes	Yes	Yes	N/A (cross-sectional)	Yes	N/A (cross-sectional)	Minimal	None	None	Low
Wallmann et al	2009	Balance	Low	Yes	N/A (single group study)	Yes	N/A (single group study; no control group)	Yes	Yes	Serious (single group uncontrolled study)	None	None	Very Low
Wu et al	2011	Body composition	Low	Yes	Unclear	Yes	N/A (cross sectional)	Yes	N/A (cross sectional)	Minimal	None	None	Low
		Flexibility	Low	Yes	Unclear	Yes	N/A (cross sectional)	Yes	N/A (cross sectional)	Minimal	None	None	Low
		Bone density	Low	Yes	Unclear	Yes	N/A (cross sectional)	Yes	N/A (cross sectional)	Minimal	None	None	Low
		Strength	Low	Yes	Unclear	Yes	N/A (cross sectional)	Yes	N/A (cross sectional)	Minimal	None	None	Low
Zhang et al	2008	Gait speed	Low	Yes	Yes	Yes	N/A (cross-sectional)	Yes	N/A (cross-sectional)	Minimal	None	None	Low
		Flexibility	Low	Yes	Yes	Yes	N/A (cross-sectional)	Yes	N/A (cross-sectional)	Minimal	None	None	Low
		Reaction time	Low	Yes	Yes	Yes	N/A (cross-sectional)	Yes	N/A (cross-sectional)	Minimal	None	None	Low
		Balance	Low	Yes	Yes	Yes	N/A (cross-sectional)	Yes	N/A (cross-sectional)	Minimal	None	None	Low
Teixeira-Machado, Arida & de Jesus Mari, 2019													
Burzynska et al.	2017	Fluid intelligence	Low	Yes	Yes	Yes	N/A (cross-sectional)	Yes	N/A (cross-sectional)	Minimal	None	None	Low
		Perceptual speed	Low	Yes	Yes	Yes	N/A (cross-sectional)	Yes	N/A (cross-sectional)	Minimal	None	None	Low
		Spatial/relational memory	Low	Yes	Yes	Yes	N/A (cross-sectional)	Yes	N/A (cross-sectional)	Minimal	None	None	Low
		Balance	Low	Yes	Yes	Yes	N/A (cross-sectional)	Yes	N/A (cross-sectional)	Minimal	None	None	Low
		Cognitive structure & function	Low	Yes	Yes	Yes	N/A (cross-sectional)	Yes	N/A (cross-sectional)	Minimal	None	None	Low
Veronese et al., 2017													

Table S11. GRADE Certainty of Evidence ratings for each outcome measure from individual randomized controlled trials (RCTs) of music participation contained in included reviews.

Author	*Year*	*Outcome*	***GRADE Initial quality***	*Allocation concealed*	*Blinded assessors/ data analysts?*	*Low % lost to follow up?*	*Intention to treat analysis?*	*All outcome result(s) reported?*	*Validated outcome measures used*	*Used individual randomization (not cluster randomization)*	*Carryover effects controlled for (cross-over trial)?*	***GRADE Bias risk***	***GRADE Indirectness***	***GRADE up rating***	***GRADE Certainty of Evidence***
Butzlaff, 2000															
Kelly	1981	Reading ability (experimental)	High	Yes	No	Yes	Yes	Yes	Yes	Yes	N/A	Serious (assessments performed by classroom teachers who were likely aware of group allocations)	None	None	Moderate
Clift et al., 2010															
Unwin, Kenny & Davis	2002	Mood	High	Yes	N/A (self-report instrument); unspecified blinding for data analysis	Yes	Yes	Yes	Yes	Yes	Yes	Serious (active music listening control group)	None	None	Moderate
		Mood	High	Yes	N/A (self-report instrument); unspecified blinding for data analysis	Yes	Yes	Yes	Yes	Yes	Yes	Serious (active music listening control group)	None	None	Moderate
Coffey, Mogilever & Zatorre, 2017															
Slater et al.	2015	Speech in Noise	High	Yes	Unclear (likely not, but randomized administration of tasks during speech in noise testing)	No (>50%; 80 initially enrolled, 38 available for analysis)	No (selective analysis of those who participated in whole intervention)	Yes	Yes	Yes	N/a	Very serious (high % lost to follow up + selective analysis of active participants + likely no blinding of assessors/data analysts)	None	None	Low

Daykin et al, 2013															
Bittman, Dickson & Coddington	2009	Social functioning	High	Yes	No	Yes	Unclear (8 participants excluded for missing 2 or more sessions due to 'illness, discharge, or physician appointments)	Yes	Yes	Yes	Yes	Serious (unblinded data collection & analysis + questionable intent to treat analysis)	None	None	Moderate
		Psychosocial function	High	YEs	No (N/A for assessors; self-report	Yes	Unclear (8 participants excluded for missing 2 or more sessions due to 'illness, discharge, or physician appointments)	Yes	Yes	Yes	Yes	Serious (unblinded data analysis + questionable intent to treat analysis)	None	None	Moderate
		Anger	High	Yes	No (N/A for assessors; self-report	Yes	Unclear (8 participants excluded for missing 2 or more sessions due to 'illness, discharge, or physician appointments)	Yes	Yes	Yes	Yes	Serious (unblinded data analysis + questionable intent to treat analysis)	None	None	Moderate
		Depression	High	Yes	No (N/A for assessors; self-report	Yes	Unclear (8 participants excluded for missing 2 or more sessions due to 'illness, discharge, or physician appointments)	Yes	Yes	Yes	Yes	Serious (unblinded data analysis + questionable intent to treat analysis)	None	None	Moderate
Kennedy	1998	Self-esteem	High	Yes	No (self-report measure)	Yes (0%)	Yes	Yes	Yes	Yes	N/A	Minimal	None	None	High
Daykin et al., 2018															

Coulton et al.	2015	Quality of Life (mental)	High	Yes	N/A; unspecified (self report questionnaires; unspecified blinding of analysts)	Yes (20% lost to follow up)	Yes	Yes	Yes	Yes	N/A	Minimal	None	None	High
		Quality of Life (physical)	High	Yes	N/A; unspecified (self report questionnaires; unspecified blinding of analysts)	Yes (20% lost to follow up)	Yes	Yes	Yes	Yes	N/A	Minimal	None	None	High
		Anxiety	High	Yes	N/A; unspecified (self report questionnaires; unspecified blinding of analysts)	Yes (20% lost to follow up)	Yes	Yes	Yes	Yes	N/A	Minimal	None	None	High
		Depression	High	Yes	N/A; unspecified (self report questionnaires; unspecified blinding of analysts)	Yes (20% lost to follow up)	Yes	Yes	Yes	Yes	N/A	Minimal	None	None	High
Koelsch, Offermanns & Franzke	2010	Depression/ anxiety	High	Yes	N/A; unspecified (self report questionnaires; unspecified blinding of analysts)	N/A (experiment completed in a single day)	Yes	Yes	Yes	Yes	N/A	Minimal	None	None	High
		Fatigue	High	Yes	N/A; unspecified (self report questionnaires; unspecified blinding of analysts)	N/A (experiment completed in a single day)	Yes	Yes	Yes	Yes	N/A	Minimal	None	None	High
		Vigor	High	Yes	N/A; unspecified	N/A (experiment	Yes	Yes	Yes	Yes	N/A	Minimal	None	None	High

			(self report questionnaire s; unspecified blinding of assessors)	completed in a single day)										
Mood (Irritability)	High	Yes	N/A; unspecified (self report questionnaire s; unspecified blinding of analysts)	N/A (experiment completed in a single day)	Yes	Yes	Yes	Yes	N/A	Minimal	None	None		High
Valence / positive affect	High	Yes	N/A; unspecified (self report questionnaire s; unspecified blinding of analysts)	N/A (experiment completed in a single day)	Yes	Yes	Yes	Yes	N/A	Minimal	None	None		High
Arousal	High	Yes	N/A; unspecified (self report questionnaire s; unspecified blinding of analysts)	N/A (experiment completed in a single day)	Yes	Yes	Yes	Yes	N/A	Minimal	None	None		High
Mood (Happiness)	High	Yes	N/A; unspecified (self report questionnaire s; unspecified blinding of analysts)	N/A (experiment completed in a single day)	Yes	Yes	Yes	Yes	N/A	Minimal	None	None		High
Mood (Anger)	High	Yes	N/A; unspecified (self report questionnaire s; unspecified blinding of analysts)	N/A (experiment completed in a single day)	Yes	Yes	Yes	Yes	N/A	Minimal	None	None		High
Mood (Sadness)	High	Yes	N/A; unspecified (self report questionnaire	N/A (experiment completed in a single day)	Yes	Yes	Yes	Yes	N/A	Minimal	None	None		High

					s; unspecified blinding of assessors)										
		Anxiety	High	Yes	N/A; unspecified (self report questionnaires; unspecified blinding of analysts)	N/A (experiment completed in a single day)	Yes	Yes	Yes	Yes	N/A	Minimal	None	None	High
		Mood (Disgust)	High	Yes	N/A; unspecified (self report questionnaires; unspecified blinding of analysts)	N/A (experiment completed in a single day)	Yes	Yes	Yes	Yes	N/A	Minimal	None	None	High
Eells, 2014															
Fancourt, Ockelford & Belai, 2014															
Bittman et al	2001	Anxiety	High	Yes	Not specified (analysis likely not blinded)	N/A (single visit intervention	No	No (outcomes from 1 of 5 drumming groups 'with the largest effect on NK cell activity' reported only; additional 50 control participants with unreported data and unclear utility)	Yes	Yes	N/A	Very serious (unblinded assessment & analysis + substantial amounts of selective outcome reporting)	None	None	Low
		Depression	High	Yes	Not specified (analysis likely not blinded)	N/A (single visit intervention	No	No (outcomes from 1 of 5 drumming groups 'with the largest effect on NK cell activity' reported only;	Yes	Yes	N/A	Very serious (unblinded assessment & analysis + substantial amounts of selective	None	None	Low

							additional 50 control participants with unreported data and unclear utility)				outcome reporting)			
	White blood cell count	High	Yes	Not specified (analysis likely not blinded)	N/A (single visit intervention	No	No (outcomes from 1 of 5 drumming groups ‘with the largest effect on NK cell activity‘ reported only; additional 50 control participants with unreported data and unclear utility)	Yes	Yes	N/A	Very serious (unblinded assessment & analysis + substantial amounts of selective outcome reporting)	None	None	Low
	Serum cortisol	High	Yes	Not specified (analysis likely not blinded)	N/A (single visit intervention	No	No (outcomes from 1 of 5 drumming groups ‘with the largest effect on NK cell activity‘ reported only; additional 50 control participants with unreported data and unclear utility)	Yes	Yes	N/A	Very serious (unblinded assessment & analysis + substantial amounts of selective outcome reporting)	None	None	Low
	NK cell activity	High	Yes	Not specified (analysis likely not blinded)	N/A (single visit intervention	No	No (outcomes from 1 of 5 drumming groups ‘with the largest effect on NK cell activity‘ reported only; additional 50 control	Yes	Yes	N/A	Very serious (unblinded assessment & analysis + substantial amounts of selective outcome reporting)	None	None	Low

							participants with unreported data and unclear utility)							
	LAK cell activity	High	Yes	Not specified (analysis likely not blinded)	N/A (single visit intervention	No	No (outcomes from 1 of 5 drumming groups ‘with the largest effect on NK cell activity‘ reported only; additional 50 control participants with unreported data and unclear utility)	Yes	Yes	N/A	Very serious (unblinded assessment & analysis + substantial amounts of selective outcome reporting)	None	None	Low
	IL-2	High	Yes	Not specified (analysis likely not blinded)	N/A (single visit intervention	No	No (outcomes from 1 of 5 drumming groups ‘with the largest effect on NK cell activity‘ reported only; additional 50 control participants with unreported data and unclear utility)	Yes	Yes	N/A	Very serious (unblinded assessment & analysis + substantial amounts of selective outcome reporting)	None	None	Low
	IFN-alpha	High	Yes	Not specified (analysis likely not blinded)	N/A (single visit intervention	No	No (outcomes from 1 of 5 drumming groups ‘with the largest effect on NK cell activity‘ reported only; additional 50 control participants with unreported	Yes	Yes	N/A	Very serious (unblinded assessment & analysis + substantial amounts of selective outcome reporting)	None	None	Low

								data and unclear utility)							
		Plasma DHEA	High	Yes	Not specified (analysis likely not blinded)	N/A (single visit intervention	No	No (outcomes from 1 of 5 drumming groups 'with the largest effect on NK cell activity' reported only; additional 50 control participants with unreported data and unclear utility)	Yes	Yes	N/A	Very serious (unblinded assessment & analysis + substantial amounts of selective outcome reporting)	None	None	Low
		DHEA to cortisol ratio	High	Yes	Not specified (analysis likely not blinded)	N/A (single visit intervention	No	No (outcomes from 1 of 5 drumming groups 'with the largest effect on NK cell activity' reported only; additional 50 control participants with unreported data and unclear utility)	Yes	Yes	N/A	Very serious (unblinded assessment & analysis + substantial amounts of selective outcome reporting)	None	None	Low
Kuhn	2002	Serum Immunoglobulin-A	High	Yes	Unclear	Yes	Yes	Yes	Yes	Yes	N/A	Minimal (unclear blinding of laboratory analysis of salivary samples unlikely to have major impact)	None	None	High
Gick, 2012															

Gordon, Fehd & McCandliss, 2015															
Cogo-Moreira et al.	2013	Reading fluency	High	Yes	Somewhat (intended, but group status often revealed)	Yes (only 13/235 lost to follow up)	Yes	Yes	Yes	No (cluster randomized by school)	N/A	Serious (cluster randomized, plus variable blinding of assessors)	None	None	Moderate
		Phonological awareness (other)	High	Yes	Somewhat (intended, but group status often revealed)	Yes (only 13/235 lost to follow up)	Yes	Yes	Yes	No (cluster randomized by school)	N/A	Serious (cluster randomized, plus variable blinding of assessors)	None	None	Moderate
Dege & Schwarzer	2011	Phonological awareness (other)	High	Yes	Not specified (likely not blinded)	Yes	Yes	Yes	Yes	Yes	N/A	Serious (unblinded assessors)	None	None	Moderate
Herrera et al	2011	Phonological awareness (rhyming)	High	Unclear	Unspecified	Yes	Yes	Yes	Yes	Unclear	Yes (6 month break)	Serious (unclear randomization procedures)	None	None	Moderate
		Phonological awareness (other)	High	Unclear	Unspecified	Yes	Yes	Yes	Yes	Unclear	Yes (6 month break)	Serious (unclear randomization procedures)	None	None	Moderate
		Phonological awareness (other)	High	Unclear	Unspecified	Yes	Yes	Yes	Yes	Unclear	Yes (6 month break)	Serious (unclear randomization procedures)	None	None	Moderate
		Phonological awareness (rhyming)	High	Unclear	Unspecified	Yes	Yes	Yes	Yes	Unclear	Yes (6 month break)	Serious (unclear randomization procedures)	None	None	Moderate
Moreno et al.	2011	Phonological awareness (rhyming)	High	Yes	Yes	Yes	No (5 participants excluded for exhibiting floor or ceiling effects on visual/auditory learning tests at baseline)	Yes	Yes	Yes	N/A	Minimal	None	None	High
Moreno et al.	2009	Reading fluency	High	Unclear (‘pseudo-random-ization’)	Unspecified	Yes	Yes	Yes	Yes	Unclear	N/A	Serious (unclear randomization procedures)	None	None	Moderate

Hetland, 2000														
Flohr et al	1998	Spatial reasoning	High	Yes	Unclear	Yes	Yes	Yes	Yes	Yes	N/A	Minimal	None	None	High
Flohr, Miller and Persellin	1998	Spatial reasoning, spatial ability	High	Yes	Unclear	Yes	Yes	Yes	Yes	Yes	N/A	Minimal	None	None	High
Flohr, Miller and Persellin	2000	Spatial reasoning	High	Yes	Unclear	Yes	Yes	Yes	Yes	Yes	N/A	Minimal	None	None	High
Rauscher	1999	Spatial reasoning	High	Yes	Unclear	Yes	Yes	Yes	Yes	Yes	N/A	Minimal	None	None	High
Rauscher	1999	Spatial reasoning	High	Yes	Unclear	Yes	Yes	Yes	Yes	Yes	N/A	Minimal	None	None	High
Jaschke et al., 2013														
Costa-Giomi	2004	Language processing	High	Yes	Not specified (analysis likely not blinded)	No (117 enrolled; 81 available for analysis)	Yes	Yes	Yes	Yes	N/A	Serious (unblinded analysis + high % lost to follow up)	None	None	Moderate
		Mathematical ability	High	Yes	Not specified (analysis likely not blinded)	No (117 enrolled; 80 available for analysis)	Yes	Yes	Yes	Yes	N/A	Serious (unblinded analysis + high % lost to follow up)	None	None	Moderate
Dege & Schwarzer	2011	Reading ability	High	Yes	Not specified (likely not blinded)	Yes	Yes	Yes	Yes	Yes	N/A	Serious (unblinded assessors)	None	None	Moderate
Rickard, Bambrick & Gill	2012	IQ	High	No (students had some input into their allocated group)	Not specified	Yes	Yes	Yes	Yes	Yes	N/A	Very serious (participants involved in randomization process + unblinded assessment & analysis of outcomes)	None	None	Low
		Writing ability	High	No (students had some input into their	Not specified	Yes	Yes	Yes	Yes	Yes	N/A	Very serious (participants involved in randomization process + unblinded	None	None	Low

				allocated group)								assessment & analysis of outcomes)			
Rickard, Bambrick & Gill	2012	Reading ability	High	Yes	Not specified	Yes	Yes	Yes	Yes	Yes	N/A	Minimal (administration and distribution of VELS results by Victorian State Government (external to study))	None	None	High
		Mathematical ability	High	Yes	Not specified	Yes	Yes	Yes	Yes	Yes	N/A	Minimal (administration and distribution of VELS results by Victorian State Government (external to study))	None	None	High
Schellen-berg	2004	IQ	High	Yes	Yes	Yes	Yes	Yes	Yes	Yes	N/A	Minimal	None	None	High
		Mathematical ability	High	Yes	Yes	Yes	Yes	Yes	Yes	Yes	N/A	Minimal	None	None	High
		Reading ability	High	Yes	Yes	Yes	Yes	Yes	Yes	Yes	N/A	Minimal	None	None	High
Moore, 2013															
Phillips & Becker, 2019															
Bittman et al.	2003	Burnout (emotional exhaustion)	High	No (random selection of subjects, but assign-ment based on schedules)	N/A; unspecified (self-report assessments, but no stated blinding of statistical analyses)	Yes	Yes	Yes	Yes* (validated measures but results not rigorously analyzed (1-tailed t-test, separate analysis of pre/post effects of music & control groups)	Yes	Yes	Very serious (no rigorous statistical analysis - 1-tailed t-test, separate pre/post analysis of music & control groups only)	None	None	Low

		Burnout (depersonal-ization)	High	No (random selection of subjects, but assign-ment based on schedules)	N/A; unspecified (self-report assessments, but no stated blinding of statistical analyses)	Yes	Yes	Yes	Yes* (validated measures but results not rigorously analyzed (1-tailed t-test, separate analysis of pre/post effects of music & control groups)	Yes	Yes	Very serious (no rigorous statistical analysis - 1-tailed t-test, separate pre/post analysis of music & control groups only)	None	None	Low
		Burnout (personal accomplish-ment)	High	No (random selection of subjects, but assignmen t based on schedules)	N/A; unspecified (self-report assessments, but no stated blinding of statistical analyses)	Yes	Yes	Yes	Yes* (validated measures but results not rigorously analyzed (1-tailed t-test, separate analysis of pre/post effects of music & control groups)	Yes	Yes	Very serious (no rigorous statistical analysis - 1-tailed t-test, separate pre/post analysis of music & control groups only)	None	None	Low
		Mood	High	No (random selection of subjects, but assignmen t based on schedules)	N/A; unspecified (self-report assessments, but no stated blinding of statistical analyses)	Yes	Yes	Yes	Yes* (validated measures but results not rigorously analyzed (1-tailed t-test, separate analysis of pre/post effects of music & control groups)	Yes	Yes	Very serious (no rigorous statistical analysis - 1-tailed t-test, separate pre/post analysis of music & control groups only)	None	None	Low
Raglio et al., 2019															
Bae	2011	Job engagement	High	Yes-ish (random allocation 'after being matched with the available days and	Not specified (unlikely)	Yes	No (10 of initial 30 participants excluded from analysis because of 'time conflicts and	Yes	Yes	Yes	N/A	Very serious (potentially biased randomization; not intention to treat analysis; comparative (rather than inactive)	None	None	Low

			times'; potentially not truly unbiased randomization)			inconsistent participation')					control group meaning that only pre-post results considered for purposes of this review)			
	Anxiety	High	High	Yes-ish (random allocation 'after being matched with the available days and times'; potentially not truly unbiased randomization)	Not specified (unlikely)	Yes	No (10 of initial 30 participants excluded from analysis because of 'time conflicts and inconsistent participation')	Yes	Yes	Yes	Very serious (potentially biased randomization; not intention to treat analysis; comparative (rather than inactive) control group meaning that only pre-post results considered for purposes of this review)	None	None	Low
	Mood	High	High	Yes-ish (random allocation 'after being matched with the available days and times'; potentially not truly unbiased randomization)	Not specified (unlikely)	Yes	No (10 of initial 30 participants excluded from analysis because of 'time conflicts and inconsistent participation')	Yes	Yes	Yes	Very serious (potentially biased randomization; not intention to treat analysis; comparative (rather than inactive) control group meaning that only pre-post results considered for purposes of this review)	None	None	Low
	Perceived self-efficacy	High	High	Yes-ish (random allocation 'after being matched with	Not specified (unlikely)	Yes	No (10 of initial 30 participants excluded from analysis because of 'time	Yes	Yes	Yes	Very serious (potentially biased randomization; not intention to	None	None	Low

				the available days and times'; potentially not truly unbiased randomization)			conflicts and inconsistent participation')				treat analysis; comparative (rather than inactive) control group meaning that only pre-post results considered for purposes of this review)			
	Positive affect	High	High	Yes-ish (random allocation 'after being matched with the available days and times'; potentially not truly unbiased randomization)	Not specified (unlikely)	Yes	No (10 of initial 30 participants excluded from analysis because of 'time conflicts and inconsistent participation')	Yes	Yes	Yes	Very serious (potentially biased randomization; not intention to treat analysis; comparative (rather than inactive) control group meaning that only pre-post results considered for purposes of this review)	None	None	Low
	Negative affect	High	High	Yes-ish (random allocation 'after being matched with the available days and times'; potentially not truly unbiased randomization)	Not specified (unlikely)	Yes	No (10 of initial 30 participants excluded from analysis because of 'time conflicts and inconsistent participation')	Yes	Yes	Yes	Very serious (potentially biased randomization; not intention to treat analysis; comparative (rather than inactive) control group meaning that only pre-post results considered for purposes of this review)	None	None	Low

Skingley & Vella-Burrows, 2010															
Talmini et al, 2017															
Vaughn, 2000															
Yap, Kwan & Ang, 2017															
Wachi et al.	2007	Mood disturbance	High	Yes	Not specified	Yes	Yes (1 of 40)	Yes	Yes	Yes	Yes	Minimal	None	None	High
		Natural killer cell activity (effector:target 25:1)	High	Yes	Not specified	Yes	Yes (1 of 40)	Yes	Yes	Yes	Yes	Minimal	None	None	High
		Natural killer cell activity (effector:target 12:1)	High	Yes	Not specified	Yes	Yes (1 of 40)	Yes	Yes	Yes	Yes	Minimal	None	None	High
		Natural killer cell activity/ regulation	High	Yes	Not specified	Yes	Yes (1 of 40)	Yes	Yes	Yes	Yes	Minimal	None	None	High
		CD-56+ cells	High	Yes	Not specified	Yes	Yes (1 of 40)	Yes	Yes	Yes	Yes	Minimal	None	None	High
		IFN-gamma gene expression (mRNA)	High	Yes	Not specified	Yes	Yes (1 of 40)	Yes	Yes	Yes	Yes	Minimal	None	None	High
		IL-2 gene expression (mRNA)	High	Yes	Not specified	Yes	Yes (1 of 40)	Yes	Yes	Yes	Yes	Minimal	None	None	High
		IL-6 gene expression (mRNA)	High	Yes	Not specified	Yes	Yes (1 of 40)	Yes	Yes	Yes	Yes	Minimal	None	None	High
		IL-10 gene expression (mRNA)	High	Yes	Not specified	Yes	Yes (1 of 40)	Yes	Yes	Yes	Yes	Minimal	None	None	High
		Leukocyte counts	High	Yes	Not specified	Yes	Yes (1 of 40)	Yes	Yes	Yes	Yes	Minimal	None	None	High

Table S12. GRADE Certainty of Evidence ratings for each outcome measure from individual non-randomized studies of music participation contained in included reviews.

Author	*Year*	*Outcome*	***GRADE Initial quality***	*Eligibility criteria developed and appropriately applied*	*Exposed/ unexposed sourced from a common population*	*Appropriate & rigorous measurement of exposure & outcome*	*Identical surveillance for outcome in exposed & unexposed*	*Known confounders adequately measured, controlled & adjusted for*	*Follow up period complete*	***GRADE Bias risk***	***GRADE Indirectness***	***GRADE up rating***	***GRADE Certainty of Evidence***
Butzlaff, 2000													
College Board	1988-1998 (not 1993)	Reading ability (correlation)	Low	Yes	Yes	Yes	N/A (cross sectional study)	Yes	N/A (cross sectional)	Minimal	None	'+1 massive sample size with consistent results across years	Moderate
Douglas & Willats	1994	Reading ability (experimental)	Low	Yes	N/A (no control group; correlational analyses only)	Yes	N/A (cross sectional study)	Yes	N/A (cross sectional)	Minimal	None	None	Low
Engdahl	1994	Reading ability (correlation)	Low	Yes	Yes	Yes	Yes	Yes	Yes	Minimal	None	None	Low
Fetzer	1994	Reading ability (experimental)	Low	Yes	Yes	Yes	Yes	Uncertain	Yes	Minimal	None	None	Low
Friedman	1959	Reading ability (correlation)	Low	Yes	Yes	Yes	Yes	Yes	Yes	Minimal	None	None	Low
Groff	1963	Reading ability (correlation)	Low	Yes	Yes	Yes	Yes	Unclear	Yes	Minimal	None	None	Low
Kvet	1985	Reading ability (correlation)	Low	Yes	Yes	Yes	Yes	Yes	Yes	Minimal	None	None	Low
Lamar	1989	Reading ability (correlation)	Low	Yes	N/A (no control group; correlational analyses only)	Yes	N/A (cross sectional study)	Yes	Yes	Minimal	None	None	Low
McCarthy	1992	Reading ability (correlation)	Low	Article access unavailable - risk of bias extrapolated from review data							None	None	Low
Olanoff & Kirschener	1969	Reading ability (experimental)	Low	Article access unavailable - risk of bias extrapolated from review data							None	None	Low
Roberts	1978	Reading ability (experimental)	Low	Article access unavailable - risk of bias extrapolated from review data							None	None	Low
Roskam	1979	Reading ability (experimental)	Low	Yes	Yes	Yes	Yes	Yes	Yes	Minimal	None	None	Low
Weeden	1971	Reading ability (correlation)	Low	Yes	Yes	Yes	Yes	Yes	Yes	Minimal	None	None	Low
Clift et al, 2010													
Beck et al.	2000	Immunoglobulin A	Low	Yes	N/A (single group analysis)	Yes	N/A (single group study)	Yes	N/A (analysis of acute effects of singing	Serious (single group	None	None	Very Low

								rehearsals and performances)	uncontrolled study)			
		Cortisol	Low	Yes	N/A (single group analysis)	Yes	N/A (single group study)	Yes	N/A (analysis of acute effects of singing rehearsals and performances)	Serious (single group uncontrolled study)	None	None	Very Low
Cohen et al	2007	Overall health	Low	Yes	Yes	No (unvalidated, bespoke instrument)	Yes	Yes	No (analysis of less than 50% of enrolled patients included in analysis - only those with available 12 month & 24 month data)	Very serious (unvalidated questionnaire + highly incomplete follow up)	None	None	Very Low
		Health system utilization	Low	Yes	Yes	No (unvalidated, bespoke instrument)	Yes	Yes	No (analysis of less than 50% of enrolled patients included in analysis - only those with available 12 month & 24 month data)	Very serious (unvalidated questionnaire + highly incomplete follow up)	None	None	Very Low
		Medication usage (over the counter + prescription)	Low	Yes	Yes	No (unvalidated, bespoke instrument)	Yes	Yes	No (analysis of less than 50% of enrolled patients included in analysis - only those with available 12 month & 24 month data)	Very serious (unvalidated questionnaire + highly incomplete follow up)	None	None	Very Low
		Falls incidence	Low	Yes	Yes	No (unvalidated, bespoke instrument)	Yes	Yes	No (analysis of less than 50% of enrolled patients included in analysis - only those with	Very serious (unvalidated questionnaire + highly incomplete follow up)	None	None	Very Low

									available 12 month & 24 month data)				
		Morale	Low	Yes	Yes	Yes	Yes	Yes	No (analysis of less than 50% of enrolled patients included in analysis - only those with available 12 month & 24 month data)	Serious (incomplete follow up)	None	None	Very Low
		Depression	Low	Yes	Yes	Yes	Yes	Yes	No (analysis of less than 50% of enrolled patients included in analysis - only those with available 12 month & 24 month data)	Serious (incomplete follow up)	None	None	Very Low
		Loneliness	Low	Yes	Yes	Yes	Yes	Yes	No (analysis of less than 50% of enrolled patients included in analysis - only those with available 12 month & 24 month data)	Serious (incomplete follow up)	None	None	Very Low
		Social activity level	Low	Yes	Yes	No (unvalidated, bespoke instrument)	Yes	Yes	No (analysis of less than 50% of enrolled patients included in analysis - only those with available 12 month & 24 month data)	Very serious (unvalidated questionnaire + highly incomplete follow up)	None	None	Very Low
Houston et al	1998	Anxiety	Low	Yes	Yes	Yes	Yes	Yes	Yes	Minimal	None	None	Low

		Somatic symptoms	Low	Yes	Yes	Yes	Yes	Yes	Yes	Minimal	None	None	Low
		Depression	Low	Yes	Yes	Yes	Yes	Yes	Yes	Minimal	None	None	Low
		Social functioning	Low	Yes	Yes	Yes	Yes	Yes	Yes	Minimal	None	None	Low
		Life satisfaction	Low	Yes	No (choral members sourced from a retirement home vs comparison group randomly selected from the broader community)	No (some members of comparison group still musically active)	N/A (cross sectional study)	Yes	N/A (cross sectional	Serious (authors acknowledge that comparison group is not a true control group & likely biases results)	None	None	Very Low
Wise, Hartmann & Fisher	1992	Self-actualization	Low	Yes	No (choral members sourced from a retirement home vs comparison group randomly selected from the broader community)	No (some members of comparison group still musically active)	N/A (cross sectional study)	Yes	N/A (cross sectional	Serious (authors acknowledge that comparison group is not a true control group & likely biases results)	None	None	Very Low
		Social isolation (alienation)	Low	Yes	No (choral members sourced from a retirement home vs comparison group randomly selected from the broader community)	No (some members of comparison group still musically active)	N/A (cross sectional study)	Yes	N/A (cross sectional	Serious (authors acknowledge that comparison group is not a true control group & likely biases results)	None	None	Very Low
Coffey, Mogilever & Zatorre, 2017												
Baskent & Etienne	2016	Speech in speech	Low	Yes	Unclear (matched subjects but source population unclear	Yes	N/A (cross sectional study)	Yes	N/A (cross sectional study	Minimal (source population unclear, but subjects well matched & intergroup education difference statistically controlled)	None	None	Low
Bidelman & Weiss	2014	Auditory processing	Low	Yes	Unclear	Yes	N/A (cross sectional)	Yes	N/A (cross sectional)	Minimal (unclear source populations but likely both university + robust	None	None	Low

										neurophysiologic testing)			
Boebinger et al.	2015	Speech in noise (Masked speech perception)	Low	Yes	Yes	Yes	N/A (cross sectional study)	No (analysis is underpowered to detect possible significant advantage for musicians	N/A (cross sectional study	Serious (underpowered)	None	None	Very low
		Duration discrimination	Low	Yes	Yes	Yes	N/A (cross sectional study)	Yes	N/A (cross sectional study	Minimal	None	None	Low
		Pitch discrimination	Low	Yes	Yes	Yes	N/A (cross sectional study)	Yes	N/A (cross sectional study	Minimal	None	None	Low
		IQ (nonverbal)	Low	Yes	Yes	Yes	N/A (cross sectional study)	Yes	N/A (cross sectional study	Minimal	None	None	Low
		Selective attention	Low	Yes	Yes	Yes	N/A (cross sectional study)	Yes	N/A (cross sectional study	Minimal	None	None	Low
		Mental flexibility (Motor speed)	Low	Yes	Yes	Yes	N/A (cross sectional study)	Yes	N/A (cross sectional study	Minimal	None	None	Low
		Mental flexibility (Fluid intelligence)	Low	Yes	Yes	Yes	N/A (cross sectional study)	Yes	N/A (cross sectional study	Minimal	None	None	Low
		Mental flexibility (Task switching)	Low	Yes	Yes	Yes	N/A (cross sectional study)	Yes	N/A (cross sectional study	Minimal	None	None	Low
Clayton et al	2016	IQ (nonverbal)	Low	Yes	Unclear (likely that subjects sourced from university population but not explicitly stated)	Yes	N/A (cross sectional study)	Yes	N/A (cross sectional study	Minimal	None	None	Low
		Executive function (Inhibition control)	Low	Yes	Unclear (likely that subjects sourced from university population but not explicitly stated)	Yes	N/A (cross sectional study)	Yes	N/A (cross sectional study	Minimal	None	None	Low
		Executive function (Cognitive flexibility/goal directed behavior)	Low	Yes	Unclear (likely that subjects sourced from university population but not explicitly stated)	Yes	N/A (cross sectional study)	Yes	N/A (cross sectional study	Minimal	None	None	Low
		Speech in speech	Low	Yes	Unclear (likely that subjects sourced from university population but not explicitly stated)	Yes	N/A (cross sectional study)	Yes	N/A (cross sectional study	Minimal	None	None	Low

		Selective attention (Multiple object tracking)	Low	Yes	Unclear (likely that subjects sourced from university population but not explicitly stated)	Yes	N/A (cross sectional study)	Yes	N/A (cross sectional study	Minimal	None	None	Low
Coffey et al	2016	Pitch discrimination	Low	Yes	N/A	Yes	N/A (cross sectional)	No (very small sample of musicians (n=12) for correlational analyses)	N/A (cross sectional	Serious (small sample size undermines confidence in replicability of correlation results)	None	None	Very Low
		Auditory processing	Low	Yes	N/A	Yes	N/A (cross sectional)	No (very small sample of musicians (n=12) for correlational analyses)	N/A (cross sectional	Serious (small sample size undermines confidence in replicability of correlation results)	None	None	Very Low
Du & Zatorre	2016	Speech in noise	Low	Unclear - conference abstract summary only available, risk of bias extrapolated from review data						Unknown	None	None	Low
Fuller et al.	2014	Speech in noise	Low	Yes	Yes	Yes	N/A (cross sectional)	Yes	N/A (cross sectional	Minimal	None	None	Low
		Vocal emotion identification	Low	Yes	Yes	Yes	N/A (cross sectional)	Yes	N/A (cross sectional	Minimal	None	None	Low
		Melodic contour identification	Low	Yes	Yes	Yes	N/A (cross sectional)	Yes	N/A (cross sectional	Minimal	None	None	Low
Lee et al.	2009	Auditory processing	Low	Yes	Unclear	Yes	N/A (cross sectional)	Yes	N/A (cross sectional)	Minimal (unclear source populations but likely both university + robust testing)	None	None	Low
Musacchia et al.	2007	Auditory processing	Low	Yes	Yes	Yes	N/A (cross sectional)	Yes	N/A (cross sectional	Minimal	None	None	Low
Musacchia, Strait & Kraus	2008	Auditory processing (neural timing)	Low	Yes	Unclear	Yes	N/A (cross sectional)	Yes	N/A (cross sectional)	Minimal (unclear source populations but likely both university + robust testing)	None	None	Low
Oxenham et al.	2003	Sound in noise	Low	Yes	Unclear	Yes	N/A (cross sectional)	Yes	N/A (cross sectional)	Minimal (unclear source populations but likely both	None	None	Low

									university + robust testing)			
		Speech in noise (Frequency selectivity)	Low	Yes	Unclear	Yes	N/A (cross sectional)	Yes	N/A (cross sectional)	Minimal (unclear source populations but likely both university + robust testing)	None	None	Low
Parbery-Clark et al.	2009	Speech in Noise	Low	Yes	Unclear (likely that subjects sourced from university population but not explicitly stated)	Yes	N/A (cross sectional study)	Yes	N/A (cross sectional study	Minimal	None	None	Low
		Working memory	Low	Yes	Unclear (likely that subjects sourced from university population but not explicitly stated)	Yes	N/A (cross sectional study)	Yes	N/A (cross sectional study	Minimal	None	None	Low
		Pitch discrimination	Low	Yes	Unclear (likely that subjects sourced from university population but not explicitly stated)	Yes	N/A (cross sectional study)	Yes	N/A (cross sectional study	Minimal	None	None	Low
Parbery-Clark et al.	2011	Speech in noise	Low	Yes	Unclear (musician/nonmusician subjects well matched but source population unclear)	Yes	N/A (cross sectional)	Yes	N/A (cross sectional study	Minimal	None	None	Low
		Auditory processing (temporal acuity)	Low	Yes	Unclear (musician/nonmusician subjects well matched but source population unclear)	Yes	N/A (cross sectional)	Yes	N/A (cross sectional study	Minimal	None	None	Low
Parbery-Clark et al.	2011	Speech in noise	Low	Yes	Unclear (source population(s) not specified	Yes	N/A (cross sectional)	Yes	N/A (cross sectional)	Minimal (unclear source populations but substantial controlling of potential confounders)	None	None	Low
		IQ (nonverbal)	Low	Yes	Unclear (source population(s) not specified	Yes	N/A (cross sectional)	Yes	N/A (cross sectional)	Minimal (unclear source populations but substantial	None	None	Low

									controlling of potential confounders)			
Parbery-Clark et al.	2012	Speech in noise	Low	Yes	Unclear	Yes	N/A (cross sectional)	Yes	N/A (cross sectional)	Minimal (unclear source populations but likely both university + robust testing)	None	None	Low
		IQ (nonverbal)	Low	Yes	Unclear	Yes	N/A (cross sectional)	Yes	N/A (cross sectional)	Minimal (unclear source populations but likely both university + robust testing)	None	None	Low
Parbery-Clark et al.	2012	Auditory processing	Low	Yes	No (by design)	Yes	N/A (cross sectional)	Yes	N/A (cross sectional	Minimal	None	None	Low
Parbery-Clark, Skoe & Kraus	2009	Speech in noise	Low	Yes	Unclear	Yes	N/A (cross sectional)	Yes	N/A (cross sectional)	Minimal (unclear source populations but likely both university + robust testing)	None	None	Low
Ruggles et al.	2014	IQ	Low	Yes	Yes	Yes	N/A (cross sectional study)	Yes	N/A (cross sectional study	Minimal	None	None	Low
		Speech in noise (Voiced speech discrimination)	Low	Yes	Yes	Yes	N/A (cross sectional study)	Yes	N/A (cross sectional study	Minimal	None	None	Low
		Speech in noise (Whispered speech discrimination)	Low	Yes	Yes	Yes	N/A (cross sectional study)	Yes	N/A (cross sectional study	Minimal	None	None	Low
Ruggles et al.	2014	Pitch discrimination	Low	Yes	Yes	Unclear (unclear if validated pitch discrimination assessment used)	N/A (cross sectional study)	Yes	N/A (cross sectional study	Minimal (unclear about validation of methodology, but methodology considered and robust)	None	None	Low
		Speech in Noise	Low	Yes	Yes	Yes	N/A (cross sectional study)	Yes	N/A (cross sectional study	Minimal	None	None	Low
Slater & Kraus	2016	Speech in noise	Low	Yes	Unclear (source population(s) not specified)	Yes	N/A (cross sectional)	Yes	N/A (cross sectional)	Minimal (unclear source populations but substantial controlling of	None	None	Low

								potential confounders)			
		Speech in noise	Low	Yes	Unclear (source population(s) not specified)	Yes	N/A (cross sectional)	Yes	N/A (cross sectional)	Minimal (unclear source populations but substantial controlling of potential confounders)	None	None	Low
		Words in noise	Low	Yes	Unclear (source population(s) not specified)	Yes	N/A (cross sectional)	Yes	N/A (cross sectional)	Minimal (unclear source populations but substantial controlling of potential confounders)	None	None	Low
		IQ (nonverbal)	Low	Yes	Unclear (source population(s) not specified)	Yes	N/A (cross sectional)	Yes	N/A (cross sectional)	Minimal (unclear source populations but substantial controlling of potential confounders)	None	None	Low
Strait & Kraus	2011	Auditory processing	Low	Yes	Unclear	Yes	N/A (cross sectional)	Yes	N/A (cross sectional	Minimal (unclear source populations but likely both university + robust testing)	None	None	Low
Strait et al.	2010	Auditory working memory	Low	Yes	Unclear	Yes	N/A (cross sectional)	Yes	N/A (cross sectional)	Minimal (unclear source populations but likely both university + robust testing)	None	None	Low
		Auditory processing (attention)	Low	Yes	Unclear	Yes	N/A (cross sectional)	Yes	N/A (cross sectional)	Minimal (unclear source populations but likely both university + robust testing)	None	None	Low
		Visual attention	Low	Yes	Unclear	Yes	N/A (cross sectional)	Yes	N/A (cross sectional)	Minimal (unclear source populations but	None	None	Low

									likely both university + robust testing)			
		Frequency discrimination	Low	Yes	Unclear	Yes	N/A (cross sectional)	Yes	N/A (cross sectional)	Minimal (unclear source populations but likely both university + robust testing)	None	None	Low
		Speech in noise (backward masking)	Low	Yes	Unclear	Yes	N/A (cross sectional)	Yes	N/A (cross sectional)	Minimal (unclear source populations but likely both university + robust testing)	None	None	Low
		Speech in noise (forward masking)	Low	Yes	Unclear	Yes	N/A (cross sectional)	Yes	N/A (cross sectional)	Minimal (unclear source populations but likely both university + robust testing)	None	None	Low
		IQ (nonverbal)	Low	Yes	Unclear	Yes	N/A (cross sectional)	Yes	N/A (cross sectional)	Minimal (unclear source populations but likely both university + robust testing)	None	None	Low
Swaminathan et al.	2015	Speech in noise	Low	Yes	Unclear	Yes	N/A (cross sectional)	No reported controlling or adjustment for confounders other than 'no known neurological conditions'	N/A (cross sectional)	Serious (unclear if musician & nonmusician cohorts sourced from similar population + minimal adjustment for potential confounders (i.e. education))	None	None	Very low
Tierney, Krizman & Kraus	2015	Language skills (Phonological awareness)	Low	Yes	Yes	Yes	Yes	Yes	High % lost to follow up (68 enrolled; 40 analyzed)	Serious	None	None	Very Low

		Language skills (Phonological memory)	Low	Yes	Yes	Yes	Yes	Yes	High % lost to follow up (68 enrolled; 40 analyzed)	Serious	None	None	Very Low
		Language skills (Rapid naming)	Low	Yes	Yes	Yes	Yes	Yes	High % lost to follow up (68 enrolled; 40 analyzed)	Serious	None	None	Very Low
		Auditory processing (Subcortical response consistency)	Low	Yes	Yes	Yes	Yes	Yes	High % lost to follow up (68 enrolled; 40 analyzed)	Serious	None	None	Very Low
		Auditory processing (cortical onset response)	Low	Yes	Yes	Yes	Yes	Yes	High % lost to follow up (68 enrolled; 40 analyzed)	Serious	None	None	Very Low
Varnet et al.	2015	Speech in noise	Low	Yes	Yes	Yes	N/A (cross sectional)	Yes	N/A (cross sectional	Minimal	None	None	Low
		Auditory processing	Low	Yes	Yes	Yes	N/A (cross sectional)	Yes	N/A (cross sectional	Minimal	None	None	Low
Zendel & Alain	2012	Speech in noise	Low	Yes	Unclear	Yes	N/A (cross sectional)	Yes	N/A (cross sectional)	Minimal (unclear source populations but likely both university + robust testing)	None	None	Low
		Auditory processing (threshold)	Low	Yes	Unclear	Yes	N/A (cross sectional)	Yes	N/A (cross sectional)	Minimal (unclear source populations but likely both university + robust testing)	None	None	Low
		Auditory processing (temporal acuity (gap detection))	Low	Yes	Unclear	Yes	N/A (cross sectional)	Yes	N/A (cross sectional)	Minimal (unclear source populations but likely both university + robust testing)	None	None	Low
		Pitch discrimination (Mistuned harmonic detection)	Low	Yes	Unclear	Yes	N/A (cross sectional)	Yes	N/A (cross sectional)	Minimal (unclear source populations but likely both university + robust testing)	None	None	Low

Zendel & Alain	2009	Pitch discrimination (Two-tone detection)	Low	Yes	Unclear	Yes	N/A (cross sectional)	Yes	N/A (cross sectional)	Minimal (unclear source populations but likely both university + robust testing)	None	None	Low
Zendel et al.	2015	Words in noise	Low	Yes	Yes	Yes	N/A (cross sectional)	Yes	N/A (cross sectional)	Minimal (words in noise test procedure appears to be original but based reasonably in precedent)	None	None	Low
Daykin et al, 2013													
Daykin et al, 2018													
Cohen et al.	2006	Overall health	Low	Yes	Yes	No (unvalidated measure)	Yes	No (statistical analysis of post-intervention data only; no analysis of change from baseline levels vs control)	Yes	Very serious (unvalidated measure + non-rigorous statistical analysis)	None	None	Very Low
		Health system utilization	Low	Yes	Yes	Yes (self-report of health system utilization shown to be valid)	Yes	No (statistical analysis of post-intervention data only; no analysis of change from baseline levels vs control)	Yes	Serious (non-rigorous statistical analysis)	None	None	Very Low
		Medication usage (over the counter)	Low	Yes	Yes	No (unvalidated measure)	Yes	No (statistical analysis of post-intervention data only; no analysis of change from baseline levels vs control)	Yes	Very serious (unvalidated measure + non-rigorous statistical analysis)	None	None	Very Low
		Medication usage (prescription)	Low	Yes	Yes	No (unvalidated measure)	Yes	No (statistical analysis of post-intervention	Yes	Very serious (unvalidated measure + non-	None	None	Very Low

								data only; no analysis of change from baseline levels vs control)		rigorous statistical analysis)			
		Falls incidence	Low	Yes	Yes	No (unvalidated measure)	Yes	No (statistical analysis of post-intervention data only; no analysis of change from baseline levels vs control)	Yes	Very serious (unvalidated measure + non-rigorous statistical analysis)	None	None	Very Low
		Other health problems	Low	Yes	Yes	No (unvalidated measure)	Yes	No (statistical analysis of post-intervention data only; no analysis of change from baseline levels vs control)	Yes	Very serious (unvalidated measure + non-rigorous statistical analysis)	None	None	Very Low
		Morale	Low	Yes	Yes	Yes	Yes	No (statistical analysis of post-intervention data only; no analysis of change from baseline levels vs control)	Yes	Serious (non-rigorous statistical analysis)	None	None	Very Low
		Depression	Low	Yes	Yes	Yes	Yes	Yes	Yes	Minimal	None	None	Low
		Loneliness	Low	Yes	Yes	Yes	Yes	Yes	Yes	Minimal	None	None	Low
		Social activity level	Low	Yes	Yes	No (unvalidated measure)	Yes	No (statistical analysis of post-intervention data only; no analysis of change from baseline levels vs control)	Yes	Very serious (unvalidated measure + non-rigorous statistical analysis)	None	None	Very Low
Cohen	2009	Wellbeing	Low	Yes	Yes	Yes	Yes	Yes	N/A (analysis of acute wellbeing effects of a	Minimal	None	None	Low

								single performance				
		Wellbeing	Low	Yes	Yes	Yes	Yes	Yes	N/A (analysis of acute wellbeing effects of a single performance	Minimal	None	None	Low
		Mood (Positive feelings)	Low	Yes	Yes	No (unvalidated measure of positive & negative feelings)	Yes	Yes	N/A (experiment compares acute effects of 2 separate 30 minute sessions)	Serious (use of unvalidated measure)	None	None	Very Low
		Mood (Negative feelings)	Low	Yes	Yes	No (unvalidated measure of positive & negative feelings)	Yes	Yes	N/A (experiment compares acute effects of 2 separate 30 minute sessions)	Serious (use of unvalidated measure)	None	None	Very Low
Kreutz	2014	Cortisol	Low	Yes	Yes	Yes	Yes	Yes	N/A (experiment compares acute effects of 2 separate 30 minute sessions)	Minimal	None	None	Low
		DHEA	Low	Yes	Yes	Yes	Yes	Yes	N/A (experiment compares acute effects of 2 separate 30 minute sessions)	Minimal	None	None	Low
		Cortisol-DHEA ratio	Low	Yes	Yes	Yes	Yes	Yes	N/A (experiment compares acute effects of 2 separate 30 minute sessions)	Minimal	None	None	Low
	2014	Wellbeing (mental)	Low	Yes	Yes	Yes	Yes	Yes	Yes	Minimal	None	None	Low

Perkins & Williamon		Nutrition	Low	Yes	Yes	Yes	Yes	Yes	Yes	Minimal	None	None	Low
		Interpersonal Relations	Low	Yes	Yes	Yes	Yes	Yes	Yes	Minimal	None	None	Low
		Spiritual Growth	Low	Yes	Yes	Yes	Yes	Yes	Yes	Minimal	None	None	Low
		Stress Management	Low	Yes	Yes	Yes	Yes	Yes	Yes	Minimal	None	None	Low
		Health responsibility	Low	Yes	Yes	Yes	Yes	Yes	Yes	Minimal	None	None	Low
Valentine & Evans	2001	Blood pressure	Low	Yes	Yes	No (no robust comparisons with control group)	Yes	Yes	N/A (experiment conducted over 1 day)	Serious (uncontrolled study	None	None	Very Low
		Mood (tense arousal)	Low	Yes	Yes	No (no robust comparisons with control group)	Yes	Yes	N/A (experiment conducted over 1 day)	Serious (uncontrolled study	None	None	Very Low
		Mood (energetic arousal)	Low	Yes	Yes	No (no robust comparisons with control group)	Yes	Yes	N/A (experiment conducted over 1 day)	Serious (uncontrolled study	None	None	Very Low
		Mood (hedonic tone)	Low	Yes	Yes	No (no robust comparisons with control group)	Yes	Yes	N/A (experiment conducted over 1 day)	Serious (uncontrolled study	None	None	Very Low
Eells, 2014												
Fancourt, Ockelford & Belai, 2014												
Koyama et al.	2009	Tension/anxiety	Low	Yes	N/A (no control group)	Yes	N/A (no control group)	Yes	N/A (single visit intervention study)	Serious (uncontrolled study)	None	None	Very Low
		Depression/dejection	Low	Yes	N/A (no control group)	Yes	N/A (no control group)	Yes	N/A (single visit intervention study)	Serious (uncontrolled study)	None	None	Very Low
		Anger/hostility	Low	Yes	N/A (no control group)	Yes	N/A (no control group)	Yes	N/A (single visit intervention study)	Serious (uncontrolled study)	None	None	Very Low
		Vigor/activity	Low	Yes	N/A (no control group)	Yes	N/A (no control group)	Yes	N/A (single visit intervention study)	Serious (uncontrolled study)	None	None	Very Low

	Fatigue/inertia	Low	Yes	N/A (no control group)	Yes	N/A (no control group)	Yes	N/A (single visit intervention study)	Serious (uncontrolled study)	None	None	Very Low
	Confusion/ bewilderment	Low	Yes	N/A (no control group)	Yes	N/A (no control group)	Yes	N/A (single visit intervention study)	Serious (uncontrolled study)	None	None	Very Low
	Total mood disturbance	Low	Yes	N/A (no control group)	Yes	N/A (no control group)	Yes	N/A (single visit intervention study)	Serious (uncontrolled study)	None	None	Very Low
	Adrenaline	Low	Yes	N/A (no control group)	Yes	N/A (no control group)	Yes	N/A (single visit intervention study)	Serious (uncontrolled study)	None	None	Very Low
	Noradrenaline	Low	Yes	N/A (no control group)	Yes	N/A (no control group)	Yes	N/A (single visit intervention study)	Serious (uncontrolled study)	None	None	Very Low
	NK cell activity	Low	Yes	N/A (no control group)	Yes	N/A (no control group)	Yes	N/A (single visit intervention study)	Serious (uncontrolled study)	None	None	Very Low
	White blood cells	Low	Yes	N/A (no control group)	Yes	N/A (no control group)	Yes	N/A (single visit intervention study)	Serious (uncontrolled study)	None	None	Very Low
	Neutrophils	Low	Yes	N/A (no control group)	Yes	N/A (no control group)	Yes	N/A (single visit intervention study)	Serious (uncontrolled study)	None	None	Very Low
	Lymphocytes	Low	Yes	N/A (no control group)	Yes	N/A (no control group)	Yes	N/A (single visit intervention study)	Serious (uncontrolled study)	None	None	Very Low
	T cells	Low	Yes	N/A (no control group)	Yes	N/A (no control group)	Yes	N/A (single visit intervention study)	Serious (uncontrolled study)	None	None	Very Low
	B cells	Low	Yes	N/A (no control group)	Yes	N/A (no control group)	Yes	N/A (single visit intervention study)	Serious (uncontrolled study)	None	None	Very Low

	CD4+ T cells	Low	Yes	N/A (no control group)	Yes	N/A (no control group)	Yes	N/A (single visit intervention study)	Serious (uncontrolled study)	None	None	Very Low
	CD8+ T cells	Low	Yes	N/A (no control group)	Yes	N/A (no control group)	Yes	N/A (single visit intervention study)	Serious (uncontrolled study)	None	None	Very Low
	CD4/CD8 ratio	Low	Yes	N/A (no control group)	Yes	N/A (no control group)	Yes	N/A (single visit intervention study)	Serious (uncontrolled study)	None	None	Very Low
	Naive T cells	Low	Yes	N/A (no control group)	Yes	N/A (no control group)	Yes	N/A (single visit intervention study)	Serious (uncontrolled study)	None	None	Very Low
	Memory T cells	Low	Yes	N/A (no control group)	Yes	N/A (no control group)	Yes	N/A (single visit intervention study)	Serious (uncontrolled study)	None	None	Very Low
	Naive/memory ratio	Low	Yes	N/A (no control group)	Yes	N/A (no control group)	Yes	N/A (single visit intervention study)	Serious (uncontrolled study)	None	None	Very Low
	NK cells (count	Low	Yes	N/A (no control group)	Yes	N/A (no control group)	Yes	N/A (single visit intervention study)	Serious (uncontrolled study)	None	None	Very Low
	IFN-alpha production	Low	Yes	N/A (no control group)	Yes	N/A (no control group)	Yes	N/A (single visit intervention study)	Serious (uncontrolled study)	None	None	Very Low
	IL-2 production	Low	Yes	N/A (no control group)	Yes	N/A (no control group)	Yes	N/A (single visit intervention study)	Serious (uncontrolled study)	None	None	Very Low
	IL-4 production	Low	Yes	N/A (no control group)	Yes	N/A (no control group)	Yes	N/A (single visit intervention study)	Serious (uncontrolled study)	None	None	Very Low
	IL-6 production	Low	Yes	N/A (no control group)	Yes	N/A (no control group)	Yes	N/A (single visit intervention study)	Serious (uncontrolled study)	None	None	Very Low

		IL-10 production	Low	Yes	N/A (no control group)	Yes	N/A (no control group)	Yes	N/A (single visit intervention study)	Serious (uncontrolled study)	None	None	Very Low
	Young cohort ***above****; Old cohort* ***below***	Tension/anxiety	Low	Yes	N/A (no control group)	Yes	N/A (no control group)	Yes	N/A (single visit intervention study)	Serious (uncontrolled study)	None	None	Very Low
		Depression/dejection	Low	Yes	N/A (no control group)	Yes	N/A (no control group)	Yes	N/A (single visit intervention study)	Serious (uncontrolled study)	None	None	Very Low
		Anger/hostility	Low	Yes	N/A (no control group)	Yes	N/A (no control group)	Yes	N/A (single visit intervention study)	Serious (uncontrolled study)	None	None	Very Low
		Vigor/activity	Low	Yes	N/A (no control group)	Yes	N/A (no control group)	Yes	N/A (single visit intervention study)	Serious (uncontrolled study)	None	None	Very Low
		Fatigue/inertia	Low	Yes	N/A (no control group)	Yes	N/A (no control group)	Yes	N/A (single visit intervention study)	Serious (uncontrolled study)	None	None	Very Low
		Confusion/ bewilderment	Low	Yes	N/A (no control group)	Yes	N/A (no control group)	Yes	N/A (single visit intervention study)	Serious (uncontrolled study)	None	None	Very Low
		Total mood disturbance	Low	Yes	N/A (no control group)	Yes	N/A (no control group)	Yes	N/A (single visit intervention study)	Serious (uncontrolled study)	None	None	Very Low
		Adrenaline	Low	Yes	N/A (no control group)	Yes	N/A (no control group)	Yes	N/A (single visit intervention study)	Serious (uncontrolled study)	None	None	Very Low
		Noradrenaline	Low	Yes	N/A (no control group)	Yes	N/A (no control group)	Yes	N/A (single visit intervention study)	Serious (uncontrolled study)	None	None	Very Low

	NK cell activity	Low	Yes	N/A (no control group)	Yes	N/A (no control group)	Yes	N/A (single visit intervention study)	Serious (uncontrolled study)	None	None	Very Low
	White blood cells	Low	Yes	N/A (no control group)	Yes	N/A (no control group)	Yes	N/A (single visit intervention study)	Serious (uncontrolled study)	None	None	Very Low
	Neutrophils	Low	Yes	N/A (no control group)	Yes	N/A (no control group)	Yes	N/A (single visit intervention study)	Serious (uncontrolled study)	None	None	Very Low
	Lymphocytes	Low	Yes	N/A (no control group)	Yes	N/A (no control group)	Yes	N/A (single visit intervention study)	Serious (uncontrolled study)	None	None	Very Low
	T cells	Low	Yes	N/A (no control group)	Yes	N/A (no control group)	Yes	N/A (single visit intervention study)	Serious (uncontrolled study)	None	None	Very Low
	B cells	Low	Yes	N/A (no control group)	Yes	N/A (no control group)	Yes	N/A (single visit intervention study)	Serious (uncontrolled study)	None	None	Very Low
	CD4+ T cells	Low	Yes	N/A (no control group)	Yes	N/A (no control group)	Yes	N/A (single visit intervention study)	Serious (uncontrolled study)	None	None	Very Low
	CD8+ T cells	Low	Yes	N/A (no control group)	Yes	N/A (no control group)	Yes	N/A (single visit intervention study)	Serious (uncontrolled study)	None	None	Very Low
	CD4/CD8 ratio	Low	Yes	N/A (no control group)	Yes	N/A (no control group)	Yes	N/A (single visit intervention study)	Serious (uncontrolled study)	None	None	Very Low
	Naive T cells	Low	Yes	N/A (no control group)	Yes	N/A (no control group)	Yes	N/A (single visit intervention study)	Serious (uncontrolled study)	None	None	Very Low
	Memory T cells	Low	Yes	N/A (no control group)	Yes	N/A (no control group)	Yes	N/A (single visit intervention study)	Serious (uncontrolled study)	None	None	Very Low

		Naive/memory ratio	Low	Yes	N/A (no control group)	Yes	N/A (no control group)	Yes	N/A (single visit intervention study)	Serious (uncontrolled study)	None	None	Very Low
		NK cells (count	Low	Yes	N/A (no control group)	Yes	N/A (no control group)	Yes	N/A (single visit intervention study)	Serious (uncontrolled study)	None	None	Very Low
		IFN-alpha production	Low	Yes	N/A (no control group)	Yes	N/A (no control group)	Yes	N/A (single visit intervention study)	Serious (uncontrolled study)	None	None	Very Low
		IL-2 production	Low	Yes	N/A (no control group)	Yes	N/A (no control group)	Yes	N/A (single visit intervention study)	Serious (uncontrolled study)	None	None	Very Low
		IL-4 production	Low	Yes	N/A (no control group)	Yes	N/A (no control group)	Yes	N/A (single visit intervention study)	Serious (uncontrolled study)	None	None	Very Low
		IL-6 production	Low	Yes	N/A (no control group)	Yes	N/A (no control group)	Yes	N/A (single visit intervention study)	Serious (uncontrolled study)	None	None	Very Low
		IL-10 production	Low	Yes	N/A (no control group)	Yes	N/A (no control group)	Yes	N/A (single visit intervention study)	Serious (uncontrolled study)	None	None	Very Low
		Positive affect	Low	Yes	Yes	Yes	N/A (experimental intervention study conducted over 2 visits)	Yes	N/A (experimental intervention study conducted over 2 visits)	Minimal	None	None	Low
Kreutz et al.	2004	Negative affect	Low	Yes	Yes	Yes	N/A (experimental intervention study conducted over 2 visits)	Yes	N/A (experimental intervention study conducted over 2 visits)	Minimal	None	None	Low
		Serum immunoglobulin A/albumin	Low	Yes	Yes	Yes	N/A (experimental intervention study conducted over 2 visits)	Yes	N/A (experimental intervention study conducted over 2 visits)	Minimal	None	None	Low

		Cortisol	Low	Yes	Yes	Yes	N/A (experimental intervention study conducted over 2 visits)	Yes	N/A (experimental intervention study conducted over 2 visits)	Minimal	None	None	Low
Lindblad, Hogmark & Theorell	2007	Cortisol	Low	Yes	Yes	Yes	Yes	Yes	Yes	Minimal	None	None	Low
		Self-esteem	Low	Yes	Yes	Yes	Yes	Yes	Yes	Minimal	None	None	Low
		Social anxiety	Low	Yes	Yes	Yes	Yes	Yes	Yes	Minimal	None	None	Low
		Social functioning	Low	Yes	Yes	Yes	Yes	Yes	Yes	Minimal	None	None	Low
Gick, 2012													
Pai et al	2008	Daytime somnolence	Low	Yes	Yes	Yes	N/A (cross sectional study)	Yes	N/A (cross sectional	Minimal	None	None	Low
Grape et al	2003	Cortisol	Low	Yes	Yes	No (no true control group; comparison of amateur & professional singers only)	N/A (cross sectional study)	Yes	N/A (cross sectional study)	Serious (no non-singing control group; impact of singing vs no singing unclear)	None	None	Very Low
		Heart rate variability	Low	Yes	Yes	No (no true control group; comparison of amateur & professional singers only)	N/A (cross sectional study)	Yes	N/A (cross sectional study)	Serious (no non-singing control group; impact of singing vs no singing unclear)	None	None	Very Low
		TNF-alpha	Low	Yes	Yes	No (no true control group; comparison of amateur & professional singers only)	N/A (cross sectional study)	Yes	N/A (cross sectional study)	Serious (no non-singing control group; impact of singing vs no singing unclear)	None	None	Very Low
		Positive affect	Low	Yes	Yes	No (no true control group; comparison of amateur & professional singers only)	N/A (cross sectional study)	Yes	N/A (cross sectional study)	Serious (no non-singing control group; impact of singing vs no singing unclear)	None	None	Very Low
Beck et al	2006	Immunoglobulin A	Low	Yes	N/A (no control group)	No (self-collection of saliva samples by musicians (non expert collection))	N/A (no control group)	Yes	N/A (series of acute pre/post analyses)	Very serious (single group study + self-collection of saliva samples by musicians)	None	None	Very Low

		Cortisol	Low	Yes	N/A (no control group)	No (self-collection of saliva samples by musicians (non expert collection))	N/A (no control group)	Yes	N/A (series of acute pre/post analyses)	Very serious (single group study + self-collection of saliva samples by musicians)	None	None	Very Low
Gordon, Fehd & McCandliss, 2015													
Bolduc & Lefebvre	2012	Phonological awareness (other)	Low	Yes	Yes	Yes	Yes	Yes	Yes	Minimal	None	None	Low
Gromko	2005	Reading fluency	Low	Yes	No (exposure & control groups sourced from two different schools with significant socioeconomic differences)	Yes	Yes	No (baseline differences in socioeconomic status not controlled for in analysis)	Yes	Serious (comparison of two different groups without statistical adjustment)	None	None	Very Low
		Phonological awareness (other)	Low	Yes	No (exposure & control groups sourced from two different schools, with significant socioeconomic differences)	Yes	Yes	No (baseline differences in socioeconomic status not controlled for in analysis)	Yes	Serious (comparison of two different groups without statistical adjustment)	None	None	Very Low
Moritz et al	2013	Phonological awareness (rhyming)	Low	Yes	Yes	Yes	N/A (cross sectional study)	Yes	N/A (cross-sectional study)	Minimal	None	None	Low
		Phonological awareness (other)	Low	Yes	Yes	Yes	N/A (cross sectional study)	Yes	N/A (cross-sectional study)	Minimal	None	None	Low
Myant, Armstrong & Healy	2008	Phonological awareness (rhyming)	Low	Article access unavailable - risk of bias extrapolated from review data						Minimal	None	None	Low
		Phonological awareness (other)	Low	Article access unavailable - risk of bias extrapolated from review data						Minimal	None	None	Low
Register	2004	Reading fluency	Low	Yes	Yes	Yes	Yes	Yes	Yes	Minimal	None	None	Low
		Phonological awareness (other)	Low	Yes	Yes	Yes	Yes	Yes	Yes	Minimal	None	None	Low
Thomson, Leong & Goswami	2013	Reading fluency	Low	Yes	Yes	Yes	Yes	Yes	Yes	Minimal	None	None	Low
		Phonological awareness (rhyming)	Low	Yes	Yes	Yes	Yes	Yes	Yes	Minimal	None	None	Low
		Phonological awareness (other)	Low	Yes	Yes	Yes	Yes	Yes	Yes	Minimal	None	None	Low
	2009	Phonological awareness (rhyming)	Low	Yes	Yes	Yes	Yes	Yes	Yes	Minimal	None	None	Low

Yazejian & Peisner-Feinberg		Phonological awareness (other)	Low	Yes	Yes	Yes	Yes	Yes	Yes	Minimal	None	None	Low
Hetland, 2000													
Bilhartz, Terry & Olson	2000	Spatial ability	Low	Yes	Yes	Yes	Yes	Yes	yes	Minimal	None	None	Low
Costa-Giomi	1999	Spatial reasoning	Low	Yes	Yes	Yes	Yes	Yes	No (78 analyzed out of 117 enrolled)	Serious (analysis of only 78 of 117 enrolled children)	None	None	Very Low
Graziano, Peterson & Shaw	1999	Spatial reasoning	Low	Yes	Yes	Yes	Yes	Yes	Yes	Minimal	None	None	Low
Gromko & Poorman	1998	Spatial reasoning, spatial ability	Low	Yes	Yes	Yes	Yes	Yes	Yes	Minimal	None	None	Low
Hurwitz et al	1975	Spatial reasoning, intelligence, spatial ability	Low	Yes	Yes	Yes	No (7 months surveillance for music group, but unclear surveillance of control group activities over same period)	Yes	N/A (cross sectional	Serious (unclear activities of control group over 7 months that music group received music training)	None	None	Very Low
Lazco	1985	Intelligence	Low	Yes	Yes	Yes	Yes	Yes	Yes	Minimal	None	None	Low
Mallory & Philbrick	1995	Spatial reasoning	Low	Yes	Yes	Yes	Yes	Yes	Yes	Minimal	None	None	Low
Parente & O'Malley	1975	Spatial ability	Low	Yes	Yes	Yes	Yes	Yes	Yes	Minimal	None	None	Low
Persellin	1999	Spatial reasoning	Low	Yes	Yes	Yes	Yes	Yes	Yes	Minimal	None	None	Low
Rauscher et al	1994	Spatial reasoning	Low	Yes	Yes	Yes	Yes	Yes	Yes	Minimal	None	None	Low
Rauscher et al	1997	Spatial reasoning, spatial ability	Low	Yes	Yes	Yes	Yes	Yes	Somewhat (34 of 111 withdrew from study, but evenly distributed amongst experimental groups)	Minimal	None	None	Low
Ruascher & Zupan	1999	Spatial reasoning	Low	Yes	Yes	Yes	Yes	Yes	Yes	Minimal	None	None	Low

Taetle	1999	Spatial reasoning, spatial ability	Low	Yes	Yes	Yes	Yes	Yes	Yes	Minimal	None	None	Low
Zulauf	1993	Intelligence, spatial ability	Low	Yes	Yes	Yes	Yes	Yes	Yes	Minimal	None	None	Low
Jaschke et al., 2013													
Gromko	2005	Reading ability	Low	Yes	No (exposure & control groups sourced from two different schools, with significant socioeconomic differences)	Yes	Yes	No (baseline differences in socioeconomic status not controlled for in analysis)	Yes	Serious (comparison of two different groups without statistical adjustment)	None	None	Very Low
Ho, Cheung and Chan	2003	IQ	Low	Yes	Yes	Yes	N/A (cross-sectional study)	Yes	N/A (cross-sectional)	Minimal	None	None	Low
Jentschke, Koelsch & Friederici	2005	Reading ability	Low	Yes	No (populations sourced from different public schools - music school vs. general public schools)	Yes	N/A (cross-sectional study)	No (no adjustment for any baseline differences between trained & untrained	N/A (cross-sectional)	Serious (no adjustment for potential confounding & trained/untrained cohorts sourced from two different locations	None	None	Very Low
Piro and Ortiz	2009	Reading ability	Low	Yes	No (exposure & control groups sourced from two different schools, with significant differences in reading scores & median income)	Yes	Yes	No (baseline differences in socioeconomic status not controlled for in analysis)	Yes	Serious (comparison of two different groups without statistical adjustment)	None	None	Very Low
Portowitz et al.	2009	IQ	Low	Yes	Yes (different centers of same institution with similar eligibility criteria)	Yes	Yes	Yes	Yes	Minimal	None	None	Low
Register	2001	Writing ability	Low	Yes	Yes	Yes	Yes	No identification or adjustment for any potential confounding variables	Not for all participants (11 of 61 lost to follow up)	Serious (no adjusting for confounding + analysis only of participants with >80% attendance)	None	None	Very Low
		Reading ability	Low	Yes	Yes	Yes	Yes	No identification or adjustment for any potential	Not for all participants (11 of 61 lost to follow up)	Serious (no adjusting for confounding + analysis only of	None	None	Very Low

								confounding variables		participants with >80% attendance)			
Tsang & Conrad	2011	Cognitive - auditory	Low	Yes	Yes	Yes	N/A (cross-sectional study)	No (between group socioeconomic differences noted but not controlled for)	N/A (cross-sectional)	Serious (comparison of two different groups without statistical adjustment)	None	None	Very Low
		Reading ability	Low	Yes	Yes	Yes	N/A (cross-sectional study)	No (between group socioeconomic differences noted but not controlled for)	N/A (cross-sectional)	Serious (comparison of two different groups without statistical adjustment)	None	None	Very Low
		Reading ability	Low	Yes	Yes	Yes	N/A (cross-sectional study)	No (between group socioeconomic differences noted but not controlled for)	N/A (cross-sectional)	Serious (comparison of two different groups without statistical adjustment)	None	None	Very Low
Moore, 2013													
Bengtsson et al	2007	Emotional Regulation	Low	Yes	N/A (single group analysis)	Yes	N/A (single group study)	Yes	N/A (single cross-sectional assessment)	Minimal	Serious (emotional regulation measured through activation in proposed relevant brain regions)	None	Very Low
Berkowitz & Ansari	2008	Emotional regulation (Anterior Cingulate Cortex Activation)	Low	Yes	N/A (single group analysis)	Yes	N/A (single group study)	Yes	N/A (single cross-sectional assessment)	Minimal	Serious (as above)	None	Very Low
Brown, Martinez & Parsons	2006	Emotional regulation (Anterior Cingulate Cortex Activation)	Low	Yes	N/A (single group analysis)	Yes	N/A (single group study)	Yes	N/A (single cross-sectional assessment)	Minimal	Serious (as above)	None	Very Low
Callan et al	2006	Emotional regulation (Orbitofrontal cortex activation)	Low	Yes	N/A (single group analysis)	Yes	N/A (single group study)	Yes	N/A (single cross-sectional assessment)	Minimal	Serious (as above)	None	Very Low

de Manzano & Ullen	2012	Emotional regulation (Anterior cingulate cortex & dorsolateral prefrontal cortex activation)	Low	Yes	N/A (single group analysis)	Yes	N/A (single group study)	Yes	N/A (single cross-sectional assessment)	Minimal	Serious (as above)	None	Very Low
Jeffries, Fritz & Braun	2003	Emotional regulation (dorsolateral prefrontal cortex activation)	Low	Yes	N/A (single group analysis)	Yes	N/A (single group study)	Yes	N/A (single cross-sectional assessment)	Minimal	Serious (as above)	None	Very Low
Kleber et al	2010	Emotional regulation (anterior cingulate cortex, ventrolateral prefrontal cortex, amygdala activation)	Low	Yes	N/A (single group analysis)	Yes	N/A (single group study)	Yes	N/A (single cross-sectional assessment)	Minimal	Serious (as above)	None	Very Low
Kleber et al	2007	Emotional regulation (dorsolateral prefrontal cortex activation)	Low	Yes	N/A (single group analysis)	Yes	N/A (single group study)	Yes	N/A (single cross-sectional assessment)	Minimal	Serious (as above)	None	Very Low
Limb & Braun	2008	Emotional regulation (orbital prefrontal cortex, dorsolateral prefrontal cortex & amygdala activation)	Low	Yes	N/A (single group analysis)	Yes	N/A (single group study)	Yes	N/A (single cross-sectional assessment)	Minimal	Serious (as above)	None	Very Low
Perry et al.	1999	Emotional regulation (anterior cingulate cortex activation)	Low	Yes	N/A (single group analysis)	Yes	N/A (single group study)	Yes	N/A (single cross-sectional assessment)	Minimal	Serious (as above)	None	Very Low
Ruiz, Jabusch & Altenmueller	2009	Emotional regulation (anterior cingulate cortex activation)	Low	Yes	N/A (single group analysis)	Yes	N/A (single group study)	Yes	N/A (single cross-sectional assessment)	Minimal	Serious (as above)	None	Very Low
Vogt et al	2007	Emotional regulation (dorsolateral prefrontal cortex activation)	Low	Yes	N/A (single group analysis)	Yes	N/A (single group study)	Yes	N/A (single cross-sectional assessment)	Minimal	Serious (as above)	None	Very Low
Zarate & Zatorre	2008	Emotional regulation (anterior	Low	Yes	N/A (single group analysis)	Yes	N/A (single group study)	Yes	N/A (single cross-sectional assessment)	Minimal	Serious (as above)	None	Very Low

		cingulate cortex activation)											
Zarate, Wood & Zatorre	2010	Emotional regulation (anterior cingulate cortex activation)	Low	Yes	N/A (single group analysis)	Yes	N/A (single group study)	Yes	N/A (single cross-sectional assessment)	Minimal	Serious (as above)	None	Very Low
Phillips & Becker, 2019													
Bormann et al.	2006	Stress	Low	Yes	N/A (single group study; no control group)	Yes	N/A (single group study)	Yes	Yes	Serious (single group uncontrolled study)	None	None	Very Low
		Anxiety (state)	Low	Yes	N/A (single group study; no control group)	Yes	N/A (single group study)	Yes	Yes	Serious (single group uncontrolled study)	None	None	Very Low
		Anxiety (trait)	Low	Yes	N/A (single group study; no control group)	Yes	N/A (single group study)	Yes	Yes	Serious (single group uncontrolled study)	None	None	Very Low
		Anger (state)	Low	Yes	N/A (single group study; no control group)	Yes	N/A (single group study)	Yes	Yes	Serious (single group uncontrolled study)	None	None	Very Low
		Anger (trait)	Low	Yes	N/A (single group study; no control group)	Yes	N/A (single group study)	Yes	Yes	Serious (single group uncontrolled study)	None	None	Very Low
		Quality of life	Low	Yes	N/A (single group study; no control group)	Yes	N/A (single group study)	Yes	Yes	Serious (single group uncontrolled study)	None	None	Very Low
		Spiritual Wellbeing	Low	Yes	N/A (single group study; no control group)	Yes	N/A (single group study)	Yes	Yes	Serious (single group uncontrolled study)	None	None	Very Low
Hilliard	2006	Compassion fatigue	Low	Yes	N/A (uncontrolled study with 2 experimental groups	Yes	N/A (uncontrolled study with 2 experimental groups	No (uncontrolled study with 2 experimental groups)	Yes	Serious (uncontrolled study)	None	None	Very Low
Raglio et al., 2019													

Skingley & Vella-Burrows, 2010													
Talmini et al, 2017													
Anaya, Pisoni & Kronenberger	2016	Short-term memory	Low	Yes	Yes	Yes	N/A (cross-sectional)	Yes	N/A (cross-sectional study)	Minimal	None	None	Low
Bialystok & DePape	2009	Short-term memory	Low	Yes	Yes	Yes	N/A (cross-sectional)	Yes	N/A (cross-sectional study)	Minimal	None	None	Low
		Working memory	Low	Yes	Yes	Yes	N/A (cross-sectional)	Yes	N/A (cross-sectional study)	Minimal	None	None	Low
Boebinger et al.	2015	Short-term memory	Low	Yes	Yes	Yes	N/A (cross-sectional)	Yes	N/A (cross-sectional study)	Minimal	None	None	Low
		Working memory	Low	Yes	Yes	Yes	N/A (cross-sectional)	Yes	N/A (cross-sectional study)	Minimal	None	None	Low
Brandler & Rammsayer	2003	Long-term memory	Low	Yes	Yes	Yes	N/A (cross-sectional)	Yes	N/A (cross-sectional study)	Minimal	None	None	Low
		Long-term memory	Low	Yes	Yes	Yes	N/A (cross-sectional)	Yes	N/A (cross-sectional study)	Minimal	None	None	Low
Chan, Ho & Cheung	1998	Long-term memory	Low	Yes	Yes	Yes	N/A (cross-sectional)	Yes	N/A (cross-sectional study)	Minimal	None	None	Low
		Long-term memory	Low	Yes	Yes	Yes	N/A (cross-sectional)	Yes	N/A (cross-sectional study)	Minimal	None	None	Low
Clayton et al.	2016	Working memory	Low	Yes	Unclear, but probably (groups mostly similarly aged in young 20s; musicians sourced from Boston University, likely nonmusicians as well)	Yes	N/A (cross-sectional)	Yes	N/A (cross-sectional study)	Minimal	None	None	Low
Franklin et al.	2008	Long-term memory	Low	Yes	Yes	Yes	N/A (cross-sectional)	Yes	N/A (cross-sectional study)	Minimal	None	None	Low
		Working memory	Low	Yes	Yes	Yes	N/A (cross-sectional)	Yes	N/A (cross-sectional study)	Minimal	None	None	Low
George & Coch	2011	Short-term memory	Low	Yes	Yes	Yes	N/A (cross-sectional)	Yes	N/A (cross-sectional study)	Minimal	None	None	Low
		Working memory	Low	Yes	Yes	Yes	N/A (cross-sectional)	Yes	N/A (cross-sectional study)	Minimal	None	None	Low
		Short-term memory	Low	Yes	Yes	Yes	N/A (cross-sectional)	Yes	N/A (cross-sectional study)	Minimal	None	None	Low
	2012	Short-term memory	Low	Yes	Yes	Yes	N/A (cross-sectional)	Yes	N/A (cross-sectional study)	Minimal	None	None	Low

Hansen, Wallentin & Vuust		Working memory	Low	Yes	Yes	Yes	N/A (cross-sectional)	Yes	N/A (cross-sectional study)	Minimal	None	None	Low
		Short-term memory	Low	Yes	Yes	Yes	N/A (cross-sectional)	Yes	N/A (cross-sectional study)	Minimal	None	None	Low
		Working memory	Low	Yes	Yes	Yes	N/A (cross-sectional)	Yes	N/A (cross-sectional study)	Minimal	None	None	Low
Helmbold, Rammsayer & Altenmueller	2005	Long-term memory	Low	Yes	Yes	Yes	N/A (cross-sectional)	Yes	N/A (cross-sectional study)	Minimal	None	None	Low
		Long-term memory	Low	Yes	Yes	Yes	N/A (cross-sectional)	Yes	N/A (cross-sectional study)	Minimal	None	None	Low
Huang et al.	2010	Long-term memory	Low	Yes	Yes	Yes	N/A (cross-sectional)	Yes	N/A (cross-sectional study)	Minimal	None	None	Low
Jakobson et al.	2008	Long-term memory	Low	Yes	Yes	Yes	N/A (cross-sectional)	Yes	N/A (cross-sectional study)	Minimal	None	None	Low
		Long-term memory	Low	Yes	Yes	Yes	N/A (cross-sectional)	Yes	N/A (cross-sectional study)	Minimal	None	None	Low
Lee, Lu & Ko	2007	Short-term memory	Low	Yes	Yes	Yes	N/A (cross-sectional)	Yes	N/A (cross-sectional study)	Minimal	None	None	Low
		Working memory	Low	Yes	Yes	Yes	N/A (cross-sectional)	Yes	N/A (cross-sectional study)	Minimal	None	None	Low
		Working memory	Low	Yes	Yes	Yes	N/A (cross-sectional)	Yes	N/A (cross-sectional study)	Minimal	None	None	Low
Monahan, Kendall & Carterette	1987	Short-term memory	Low	Yes	Yes	Yes	N/A (cross-sectional study)	Yes	N/A (cross-sectional study)	Minimal	None	None	Low
Okhrei, Kutsenko & Makarchuk	2017	Short-term memory	Low	Yes	Yes	Yes	N/A (cross-sectional study)	Yes	N/A (cross-sectional study)	Minimal	None	None	Low
		Short-term memory	Low	Yes	Yes	Yes	N/A (cross-sectional study)	Yes	N/A (cross-sectional study)	Minimal	None	None	Low
Pallesen et al.	2010	Short-term memory	Low	Yes	Yes	Yes	N/A (cross-sectional study)	Yes	N/A (cross-sectional study)	Minimal	None	None	Low
		Working memory	Low	Yes	Yes	Yes	N/A (cross-sectional study)	Yes	N/A (cross-sectional study)	Minimal	None	None	Low
Parbery-Clark et al.	2011	Working memory	Low	Yes	Yes	Yes	N/A (cross-sectional study)	Yes	N/A (cross-sectional study)	Minimal	None	None	Low
Ramachandra, Meighan & Gradzki	2012	Short-term memory	Low	Yes	Yes	Yes	N/A (cross-sectional study)	Yes	N/A (cross-sectional study)	Minimal	None	None	Low
		Working memory	Low	Yes	Yes	Yes	N/A (cross-sectional study)	Yes	N/A (cross-sectional study)	Minimal	None	None	Low
Rodrigues, Loureiro & Caramelli	2014	Short-term memory	Low	Yes	Yes	Yes	N/A (cross-sectional study)	Yes	N/A (cross-sectional study)	Minimal	None	None	Low
Schiavio & Timmers	2016	Long-term memory	Low	Yes	Unclear	Yes	N/A (cross-sectional study)	Unclear	N/A (cross-sectional study)	Serious (population	None	None	Very Low

										details and rigor of adjustment for confounding unclear			
Schulze, Dowling & Tillmann	2012	Short-term memory	Low	Yes	Yes	Yes	N/A (cross-sectional study)	Yes	N/A (cross-sectional study)	Minimal	None	None	Low
		Working memory	Low	Yes	Yes	Yes	N/A (cross-sectional study)	Yes	N/A (cross-sectional study)	Minimal	None	None	Low
Schulze, Mueller & Koelsch	2011	Short-term memory	Low	Yes	Unclear, but probably both groups sourced from university cohorts	Yes	N/A (cross-sectional study)	Yes	N/A (cross-sectional study)	Minimal	None	None	Low
Schulze et al.	2011	Working memory	Low	Yes	Unclear, but probably both groups sourced from university cohorts	Yes	N/A (cross-sectional study)	Yes	N/A (cross-sectional study)	Minimal	None	None	Low
		Working memory	Low	Yes	Unclear, but probably both groups sourced from university cohorts	Yes	N/A (cross-sectional study)	Yes	N/A (cross-sectional study)	Minimal	None	None	Low
Suarez, Elangovan & Au	2015	Working memory	Low	Yes	Yes	Yes	N/A (cross-sectional study)	Yes	N/A (cross-sectional study)	Minimal	None	None	Low
		Short-term memory	Low	Yes	Yes	Yes	N/A (cross-sectional study)	Yes	N/A (cross-sectional study)	Minimal	None	None	Low
		Short-term memory	Low	Yes	Yes	Yes	N/A (cross-sectional study)	Yes	N/A (cross-sectional study)	Minimal	None	None	Low
		Long-term memory	Low	Yes	Yes	Yes	N/A (cross-sectional study)	Yes	N/A (cross-sectional study)	Minimal	None	None	Low
Talamini, Carretti, & Grassi	2016	Short-term memory	Low	Yes	Unclear, but probably both groups sourced from university cohorts	Yes	N/A (cross-sectional study)	Yes	N/A (cross-sectional study)	Minimal	None	None	Low
		Working memory	Low	Yes	Unclear, but probably both groups sourced from university cohorts	Yes	N/A (cross-sectional study)	Yes	N/A (cross-sectional study)	Minimal	None	None	Low
Taylor & Dewhurst	2017	Long-term memory	Low	Yes	Yes	Yes	N/A (cross-sectional study)	Yes	N/A (cross-sectional study)	Minimal	None	None	Low
Vasuki et al.	2016	Short-term memory	Low	Yes	Yes	Yes	N/A (cross-sectional study)	Yes	N/A (cross-sectional study)	Minimal	None	None	Low
		Working memory	Low	Yes	Yes	Yes	N/A (cross-sectional study)	Yes	N/A (cross-sectional study)	Minimal	None	None	Low
Weiss et al.	2014	Short-term memory	Low	Yes	Yes	Yes	N/A (cross-sectional study)	Yes	N/A (cross-sectional study)	Minimal	None	None	Low

Zuk et al	2014	Working memory	Low	Yes	Yes	Yes	N/A (cross-sectional study)	Yes	N/A (cross-sectional study)	Minimal	None	None	Low
Vaughn, 2000													
Annello	1972	Mathematics (correlation)	Low	Yes	Yes	Yes	N/A (cross sectional study)	Yes	N/A (cross sectional)	Minimal	None	None	Low
Catterall, Chapleau & Iwanaga	1999	Mathematics (correlation)	Low	Yes	Yes	Yes	N/A (cross sectional study)	Unclear (generally unclear description of statistical methods)	N/A (cross sectional)	Minimal	None	None	Low
Ciepluch	1988	Mathematics (correlation)	Low	Yes	N/A (single group study of correlations)	Yes	N/A (cross sectional study)	No (test of correlations between instrumental sightreading achievement & mathematical achievement - not a test of participation vs. math achievement; analysis confounding in the context of the rest of the meta-analysis)	N/A (cross sectional)	Serious (confounding analysis in context of rest of meta-analysis)	None	None	Very Low
College Board	1988-1998 (not 1993)	Mathematics (correlation)	Low	Yes	Yes	Yes	N/A (cross sectional study)	Yes	N/A (cross sectional)	Minimal	None	'+1 massive sample size with consistent results across years	Moderate
Costa-Giomi	1999	Mathematics (causation - experimental)	Low	Yes	Yes	Yes	Yes	Yes	No (78 analyzed out of 117 enrolled)	Serious (analysis of only 78 of 117 enrolled children)	None	None	Very Low
Engdahl	1994	Mathematics (correlation)	Low	Yes	Yes	Yes	Yes	Yes	Yes	Minimal	None	None	Low
Friedman	1959	Mathematics (causation - experimental)	Low	Yes	Yes	Yes	Yes	Yes	Yes	Minimal	None	None	Low

Graziano, Peterson & Shaw	1999	Mathematics (causation - experimental)	Low	Yes	Yes	Yes	Yes	Yes	Yes	Minimal	None	None	Low
Kvet	1982	Mathematics (correlation)	Low	Yes	Yes	Yes	Yes	Yes	Yes	Minimal	None	None	Low
McCarthy	1992	Mathematics (correlation)	Low	Yes	Yes	Yes	Yes	Yes	Yes	Minimal	None	None	Low
Neufeld	1986	Mathematics (causation - experimental)	Low	Yes	Yes	Yes	Yes	Yes	Yes	Minimal	None	None	Low
Weeden	1971	Mathematics (causation - experimental)	Low	Yes	Yes	Yes	Yes	Yes	Yes	Minimal	None	None	Low
Wheeler & Wheeler	1951	Mathematics (correlation)	Low	Yes	Yes	Yes	N/A (cross sectional study)	Yes	N/A (cross sectional)	Minimal	None	None	Low
Yap, Kwan & Ang, 2017													
Bittman	2004	Mood	Low	Yes	Yes	No (no direct statistical comparisons between pre-post effects in intervention vs control groups)	Yes	Yes	Yes	Serious (results not rigorously analyzed; single group study based on the way it's analyzed)	None	None	Very Low
		Emotional exhaustion	Low	Yes	Yes	No (no direct statistical comparisons between pre-post effects in intervention vs control groups)	Yes	Yes	Yes	Serious (results not rigorously analyzed; single group study based on the way it's analyzed)	None	None	Very Low
		Depersonalization	Low	Yes	Yes	No (no direct statistical comparisons between pre-post effects in intervention vs control groups)	Yes	Yes	Yes	Serious (results not rigorously analyzed; single group study based on the way it's analyzed)	None	None	Very Low
		Personal accomplishment	Low	Yes	Yes	No (no direct statistical comparisons between pre-post effects in	Yes	Yes	Yes	Serious (results not rigorously analyzed; single group study based on the way it's analyzed)	None	None	Very Low

						intervention vs control groups)							
Dunbar et al	2012	Positive affect	Low	Yes	No (by design)	Yes	Yes	Yes	N/A (experiment all conducted in 1 day)	Minimal	None	None	Low
		Negative affect	Low	Yes	No (by design)	Yes	Yes	Yes	N/A (experiment all conducted in 1 day)	Minimal	None	None	Low
Ho et al	2011	Social functioning	Low	Yes	Yes	Yes	Yes	Yes	Yes	Minimal	None	None	Low
Mungas & Silverman	2014	Drowsiness	Low	Yes	No (significant differences in musical experience between groups)	Yes	Yes	No (substantial gender difference between groups not accounted for)	N/A (experiment conducted in single day)	Serious (no controlling for gender confound + significantly greater musical experience in intervention vs control group)	None	None	Very Low
		Anxiety	Low	Yes	No (significant differences in musical experience between groups)	Yes	Yes	No (substantial gender difference between groups not accounted for)	N/A (experiment conducted in single day)	Serious (no controlling for gender confound + significantly greater musical experience in intervention vs control group)	None	None	Very Low
		Depression	Low	Yes	No (significant differences in musical experience between groups)	Yes	Yes	No (substantial gender difference between groups not accounted for)	N/A (experiment conducted in single day)	Serious (no controlling for gender confound + significantly greater musical experience in intervention vs control group)	None	None	Very Low
		Aggression	Low	Yes	No (significant differences in musical experience between groups)	Yes	Yes	No (substantial gender difference between groups not accounted for)	N/A (experiment conducted in single day)	Serious (no controlling for gender confound + significantly greater musical experience in intervention vs control group)	None	None	Very Low

		Confusion	Low	Yes	No (significant differences in musical experience between groups)	Yes	Yes	No (substantial gender difference between groups not accounted for)	N/A (experiment conducted in single day)	Serious (no controlling for gender confound + significantly greater musical experience in intervention vs control group)	None	None	Very Low
		Lack of coordination	Low	Yes	No (significant differences in musical experience between groups)	Yes	Yes	No (substantial gender difference between groups not accounted for)	N/A (experiment conducted in single day)	Serious (no controlling for gender confound + significantly greater musical experience in intervention vs control group)	None	None	Very Low
Smith, Viljoen & McGeachie	2014	Blood pressure	Low	Yes	N/A (single group study)	Yes	N/A (single group study)	Yes (pre-post design)	N/A (experiment all conducted in 1 day)	Serious (uncontrolled study)	None	None	Very Low
		Blood pressure	Low	Yes	N/A (single group study)	Yes	N/A (single group study)	Yes (pre-post design)	N/A (experiment all conducted in 1 day)	Serious (uncontrolled study)	None	None	Very Low
		Stress/anxiety	Low	Yes	N/A (single group study)	Yes	N/A (single group study)	Yes (pre-post design)	N/A (experiment all conducted in 1 day)	Serious (uncontrolled study)	None	None	Very Low
		Stress/anxiety	Low	Yes	N/A (single group study)	Yes	N/A (single group study)	Yes (pre-post design)	N/A (experiment all conducted in 1 day)	Serious (uncontrolled study)	None	None	Very Low

Table S13. Review articles excluded after full-text review, with reasons.

Author	Year	Title	Journal	Music/Dance	Review type	Reason for exclusion
Alain et al.	2014	Turning down the noise: the benefit of musical training on the aging auditory brain	Hearing Research	Music	Narrative	No reproducible search methodology / evidence of comprehensive synthesis
Angioi et al	2009	Fitness in Contemporary Dance: A Systematic Review	International Journal of Sports Medicine	Dance	Narrative	Focus on studies describing the physical fitness of dancers & evaluating the effect of supplementary exercise programs on dancers
Antoniadou, Michaelidis, Tsara	2012	Lung function in wind instrument players	Pneumon	Music	Narrative	No reproducible search methodology / evidence of comprehensive synthesis
Bashwiner & Bacon	2019	Musical creativity and the motor system	Current Opinion in Behavioral Sciences	Music	Narrative	No reproducible search methodology / evidence of comprehensive synthesis
Bonde, Juel, & Ekholm	2018	Associations between music and health-related outcomes in adult non-musicians, amateur musicians and professional musicians - Results from a nationwide Danish study	Nordic Journal of Music Therapy	Music	Narrative	No reproducible search methodology / evidence of comprehensive synthesis
Chobert & Besson	2013	Musical expertise and second language learning	Brain Sciences	Music	Narrative	No reproducible search methodology / evidence of comprehensive synthesis
Croom	2015	Music practice and participation for psychological well-being: A review of how music influences positive emotion, engagement, relationships, meaning, and accomplishment	Musicae Scientiae	Music	Narrative	No reproducible search methodology / evidence of comprehensive synthesis
Dawson	2014	Benefits of music training are widespread and lifelong: a bibliographic review of their non-musical effects	Medical Problems of Performing Artists	Music	Narrative	Focus not on comprehensively reviewing the benefits of music participation; largely non-reproducible search methodology
Dawson	2011	How and why musicians are different from nonmusicians: A bibliographic review	Medical Problems of Performing Artists	Music	Narrative	Focus not on comprehensively reviewing the benefits of music participation; largely non-reproducible search methodology
Ellis & Thayer	2010	Music and autonomic nervous system (DYS) function	Music Perception	Music	Narrative	No reproducible search methodology / evidence of comprehensive synthesis
Fernandez	2018	Music and brain development	Pediatric Annals	Music	Narrative	No reproducible search methodology / evidence of comprehensive synthesis
Herholz & Zatorre	2012	Musical Training as a Framework for Brain Plasticity: Behavior, Function, and Structure	Neuron	Music	Narrative	No reproducible search methodology / evidence of comprehensive synthesis
Huotilainen & Tervaniemi	2018	Planning music-based amelioration and training in infancy and childhood based on neural evidence	Annals of the New York Academy of Sciences	Music	Narrative	No reproducible search methodology / evidence of comprehensive synthesis
Jordan	2019	When I'm 64: A review of instrumental music-making and brain health in later life	Experimental Gerontology	Music	Narrative	No reproducible search methodology / evidence of comprehensive synthesis
Karpati et al	2015	Dance and the brain: a review	Annals of the New York Academy of Sciences	Dance	Narrative	No reproducible search methodology / evidence of comprehensive synthesis

Kraus & Chandrasekaran	2010	Music training for the development of auditory skills	Nature reviews	Music	Narrative (Perspective)	No reproducible search methodology / evidence of comprehensive synthesis
Marks	2016	Narrative review of dance-based exercise and its specific impact on depressive symptoms in older adults	AIMS Medical Science	Dance	Narrative	Focus on dance as a clinical therapeutic
Miendlarzewska	2014	How musical training affects cognitive development: Rhythm, reward and other modulating variables	Frontiers in Neuroscience	Music	Narrative	No reproducible search methodology / evidence of comprehensive synthesis
Pantev & Herholz	2011	Plasticity of the human auditory cortex related to musical training	Neuroscience and Biobehavioral Reviews	Music	Narrative	No reproducible search methodology / evidence of comprehensive synthesis
Perrone-Capano, Volpicelli, di Porzio	2017	Biological bases of human musicality	Reviews in the Neurosciences	Music	Narrative	No reproducible search methodology / evidence of comprehensive synthesis
Perrot & Collet	2014	Function and plasticity of the medial olivocochlear system in musicians: A review	Hearing Research	Music	Narrative	No reproducible search methodology / evidence of comprehensive synthesis
Sarkamo	2018	Cognitive, emotional, and neural benefits of musical leisure activities in aging and neurological rehabilitation: A critical review	Annals of Physical and Rehabilitation Medicine	Music	Narrative	No reproducible search methodology / evidence of comprehensive synthesis
Schellenberg	2001	Music and nonmusical abilities	Annals of the New York Academy of Sciences	Music	Narrative	No reproducible search methodology / evidence of comprehensive synthesis
Standley	1996	A meta-analysis on the effects of music as reinforcement for education/therapy objectives	Journal of Research in Music Education	Music	Meta-analysis	Meta-analysis of contingent music (i.e. music lessons as a reward for achieving a certain outcome)
Strassel et al	2011	A systematic review of the evidence for the effectiveness of dance therapy	Alternative Therapies	Dance	Narrative	Focus on dance as a clinical therapeutic
Sutcliffe, Du, Ruffman	2020	Music Making and Neuropsychological Aging: A Review	Neuroscience and Biobehavioral Reviews	Music	Narrative	No reproducible search methodology / evidence of comprehensive synthesis
Twitchett, Koutedakis & Wyon	2009	Psychological fitness and professional classical ballet performance: A brief review	Journal of Strength and Conditioning Research	Dance	Narrative	No reproducible search methodology / evidence of comprehensive synthesis
Wan & Schlaug	2010	Music making as a tool for promoting brain plasticity across the life span	The Neuroscientist	Music	Narrative	No reproducible search methodology / evidence of comprehensive synthesis
Wolff	1978	Nonmusical Outcomes of Music Education: A Review of the literature	Bulletin of the Council for Research in Music Education	Music	Narrative	No reproducible search methodology / evidence of comprehensive synthesis
Zalatel, Gabrilio & Peric	2013	The training effects of dance aerobics: A review with an emphasis on the perspectives of investigations	Collegium Antropologicum	Dance	Narrative	No listing of included studies + unintelligible citations
Zimmerman & Lahav	2012	The multisensory brain and its ability to learn music	Annals of the New York Academy of Sciences	Music	Narrative	No reproducible search methodology / evidence of comprehensive synthesis
Zuk & Gaab	2018	Evaluating predisposition and training in shaping the musician's brain: The need for a developmental perspective	Annals of the New York Academy of Sciences	Music	Narrative	No reproducible search methodology / evidence of comprehensive synthesis

Table S14. Details of included studies evaluating the impact of performing arts participation on non-communicable disease risk.

Author	Year	Design	Subjects	Study Country	Music/ Dance	Type of participation	Definition of participation	Outcome	Effect of participation	Outcome details	GRADE certainty of evidence
Balbag, Pederson & Gatz	2014	Case-control	157 pairs of Swedish twins at least 65 years old and discordant for dementia or cognitive impairment and with available data re. musical participation, mixed sex	Sweden	Music	Instrumental	Questionnaire regarding experience playing a musical instrument: played new and/or familiar music, frequency of playing, if still playing at time of assessment or stopped at a specific age; 'musicians' classified as those who played new and/or familiar music frequently and/or occasionally at the time of assessment OR if they stopped playing within 5 years of dementia onset	Dementia or cognitive impairment	Positive	Cognitive assessment at time of questionnaire + historical analysis of medical records	Low
Bygren, Konlaan, & Johansson	1996	Longitudinal cohort	12,675 Swedish citizens aged 16-74 years; mixed sex	Sweden	Music	Instrumental or vocal	Survey (recall period unclear) – how often participants 'played music or sang in a choir' – dichotomized into 'now and then' or less frequently than 'now and then'	All cause mortality	No effect	7-8 year follow up	Very Low
Chen et al.	2013	Cross-sectional	15,514 individuals (6,952 men; 8.562 women) aged 50-70 years from the Dongfeng-Tongji Cohort (retirees of the Dongfeng Motor Corporation) in Shiyan, China; participants excluded if history of cancer, diabetes, CVD or stroke or any missing data	China	Dance	Unspecified	Interview re. frequency and average duration of a range of physical activities (undefined recall period); average METs from ACSM compendium of physical activity used to approximate dance energy expenditure (5 METs per hour), then multiplied by frequency and duration = total MET hours per week	Metabolic syndrome	Positive	Metabolic syndrome defined using updated National Cholesterol Education Program/Adult treatment Panel III criteria for Asian Americans	Very Low
Ekholm & Bonde	2018	Cross-sectional	14,265 Danish adults (16 or older), mixed sex	Denmark	Music	Instrumental or vocal	Postal questionnaire: 'have you ever voluntarily sung in a choir/band or played a musical instrument in a band, orchestra or musical ensemble?'(yes, sing or play now; yes, previously; no); if yes, 'have you ever sung or played as a professional musician?' (same answer choices)	Asthma	No effect		Very Low
								Diabetes	No effect		Very Low
								Hypertension	No effect		Very Low
								Osteoarthritis	No effect		Very Low
								Rheumatoid Arthritis	No effect		Very Low
								Tinnitus	Negative		Very Low
Hughes et al.	2010	Longitudinal cohort	942 older adults in Pennsylvania, mixed sex	USA	Music	Instrumental	Survey – yes/no 'engagement in musical instruments as a hobby'; time spent in hobbies overall calculated, but not for individual hobbies	Dementia	No effect	Average 6 year follow up	Very Low
Konlaan, Bygren, Johansson	2000	Longitudinal cohort	10,609 Swedish citizens aged 25-74, mixed sex	Sweden	Music	Instrumental or vocal	Survey (recall period unclear) – how often participants 'played music or sang in a choir' – dichotomized into 'now and then' or less frequently than 'now and then'	All-cause mortality	Positive	14 year follow up	Very Low

Lin et al.	2019	Cross-sectional	18,424 Taiwan Biobank participants – aged 30-70 with no history of cancer and unrelated to other biobank subjects, mixed sex	Taiwan	Dance	International standard	Survey of physical activity (undefined recall period); 'regular participation' defined as weekly dancing at least 30 min/3x weekly	Obesity metrics	Positive	Surveyed metrics: BMI, body fat %, waist circumference, hip circumference, waist-to-hip ratio	Very Low
Merom, Ding, Stamatakis	2016	Longitudinal cohort	48,390 British/Scottish residents; Aged >/=40 and without baseline cardiovascular disease; n=3169 reported recreational dancing; mixed	UK	Dance	Unspecified	Health Survey for England + Scottish Health Survey (4 week recall); Interviewer-administered questionnaire re. dance participation & intensity (moderate intensity defined as 'effort usually enough to make you out of breath or sweaty')	Cardiovascular Disease Mortality	Mixed (positive – moderate intensity walking; no effect – light intensity dance)	3-17 year follow up	Very Low
Vergehese et al.	2003	Longitudinal cohort	469 community-dwellers without baseline dementia & aged 75-85 (Bronx Aging Study, mixed sex); participants also excluded with severe hearing or visual impairment, previous diagnosis of idiopathic Parkinson's, liver disease, alcoholism or known terminal illness	USA	Dance & Music	Unspecified & instrumental	Interview (undefined recall period) re. frequency of 6 cognitive activities (including 'playing musical instruments') and 11 physical activities (including 'dancing'); Participation frequency scored as: 7 (daily); 4 (several days per week); 1 (once weekly); 0 (monthly, occasionally, or never); summed scores from 6 cognitive activities = 'cognitive activity score' (out of 42); summed scores from 11 physical activities = 'physical activity score' (out of 77)	Dementia diagnosis	Positive	Median 5.1 year follow up	Low

Table S15. GRADE Certainty of Evidence ratings of each outcome measure of included studies evaluating the impact of performing arts participation on non-communicable disease risk.

**Author**	**Year**	**Outcome**	**Grade Initial Quality**	*Eligibility criteria developed and appropriately applied*	*Exposed/ unexposed sourced from a common population?*	*Appropriate & rigorous measurement of exposure & outcome*	*Identical surveillance for outcome in exposed & unexposed*	*Known confounders adequately measured, controlled & adjusted for*	*Follow up period complete?*	***GRADE Bias risk***	***GRADE Indirectness***	***GRADE up rating***	***GRADE Certainty of Evidence***
Balbag, Pederson & Gatz	2014	Dementia or cognitive impairment	Low	Yes	Yes	Yes	Yes	Yes	Yes	Minimal	None	None	Low
Bygren, Konlaan, & Johansson	1996	All cause mortality	Low	Yes	Yes	No (unvalidated/relatively insensitive measure of performing arts participation)	Yes	Yes	Yes	Serious (questionable methods & sensitivity of performing arts questionnaire)	None	None	Very Low
Chen et al.	2013	Metabolic syndrome	Low	Yes	Yes	No (undefined recall period + use of standardized conversions from ACSM for MET equivalents for all dance modes)	N/A (cross-sectional)	Yes	N/A (cross-sectional)	Serious (unvalidated measure + indirect conversion to METs)	None	None	Very Low
Ekholm & Bonde	2018	Asthma	Low	Yes	Yes	No (self-reported incidence of non-communicable diseases; insensitive measure of music participation	N/A (cross-sectional)	Yes	N/A (cross-sectional)	Serious (self-reported incidence of non-communicable diseases; insensitive measure of music participation)	None	None	Very Low
		Diabetes	Low	Yes	Yes	No (self-reported incidence of non-communicable diseases; insensitive measure of music participation	N/A (cross-sectional)	Yes	N/A (cross-sectional)	Serious (self-reported incidence of non-communicable diseases; insensitive measure of music participation)	None	None	Very Low
		Hypertension	Low	Yes	Yes	No (self-reported incidence of non-communicable diseases; insensitive measure of music participation	N/A (cross-sectional)	Yes	N/A (cross-sectional)	Serious (self-reported incidence of non-communicable diseases; insensitive measure of music participation)	None	None	Very Low
		Osteoarthritis	Low	Yes	Yes	No (self-reported incidence of non-communicable diseases; insensitive measure of music participation	N/A (cross-sectional)	Yes	N/A (cross-sectional)	Serious (self-reported incidence of non-communicable diseases;	None	None	Very Low

										insensitive measure of music participation)			
		Rheumatoid Arthritis	Low	Yes	Yes	No (self-reported incidence of non-communicable diseases; insensitive measure of music participation	N/A (cross-sectional)	Yes	N/A (cross-sectional)	Serious (self-reported incidence of non-communicable diseases; insensitive measure of music participation)	None	None	Very Low
		Tinnitus	low	Yes	Yes	No (self-reported incidence of non-communicable diseases; insensitive measure of music participation	N/A (cross-sectional)	Yes	N/A (cross-sectional)	Serious (self-reported incidence of non-communicable diseases; insensitive measure of music participation)	None	None	Very Low
Hughes et al.	2010	Dementia	Low	Yes	Yes	No (binary yes/no definition of playing musical instruments lacks sensitivity)	Yes	Yes	Yes	Serious (binary yes/no classification of playing a musical instrument lacks sensitivity	None	None	Very Low
Konlaan, Bygren, Johansson	2000	All-cause mortality	Low	Yes	Yes	No (unvalidated/relatively insensitive measure of performing arts participation)	Yes	Yes	Yes	Serious (questionable methods & sensitivity of performing arts questionnaire)	None	None	Very Low
Lin et al.	2019	Obesity metrics	Low	Yes	Yes	No (dance defined as 'exercise'; undefined recall period)	N/A (cross-sectional)	Yes	N/A (cross-sectional)	Serious (dance defined as 'exercise'; undefined recall period)	None	None	Very Low
Merom, Ding, Stamatakis	2016	Cardiovascular Disease Mortality	Low	Yes	Yes	No (unvalidated measure of dance participation)	Yes	Yes	Yes	Serious (unvalidated measure of dance participation)	None	None	Very Low
Vergehese et al.	2003	Dementia diagnosis	Low	Yes	Yes	Yes (unvalidated instrument, but good sensitivity in questions about frequency & analysis)	Yes	Yes	Yes	Minimal	None	None	Low

Table S16. Studies of non-communicable disease risk excluded after full-text review, with reasons.

Author	Year	Title	Journal	Reason for Exclusion
Bonde, Juel & Ekholm	2018	Associations between music and health-related outcomes in adult non-musicians, amateur musicians and professional musicians – Results from a nationwide Danish study	Nordic Journal of Music Therapy	No data on the impact of music participation on noncommunicable diseases or mortality; included as review article
Chomistek et al	2012	Vigorous-intensity leisure-time physical activity and risk of major chronic disease in men	Medicine and Science in Sports and Exercise	No examination of performing arts participation
Cuypers et al.	2011	Patterns of receptive and creative cultural activities and their association with perceived health, anxiety, depression and satisfaction with life among adults in the HUNT study, Norway	Journal of Epidemiology and Community Health	No examination of noncommunicable disease risk
Danneberg et al	1989	Leisure time physical activity in the Framingham Offspring study. Description, seasonal variation, and risk factor correlates	American Journal of Epidemiology	Excluded, no individual analysis of PA modes
Darweesh et al	2018	Professional occupation and the risk of Parkinson's disease	European Journal of Neurology	All arts occupations (visual & performing) grouped together
Davies, Knuiman & Rosenberg	2016	The art of being mentally healthy: A study to quantify the relationship between recreational arts engagement and Mental well-being in the general population	BMC Public Health	Relationships only examined between all arts engagement (participation & attendance at a range of art forms) and mental well-being; no specific analyses of music/dance participation
Ekholm, Bonde & Juel	2016	Music and Public Health – An empirical study of the use of music in the daily life of the adult Danish citizens and the health implications of musical participation	Arts & Health	No data on the impact of music participation on noncommunicable diseases or mortality
Ekholm, Juel & Bonde	2016	Associations between daily musicking and health: Results from a nationwide survey in Denmark	Scandinavian Journal of Public Health	No data on the impact of music participation on noncommunicable diseases or mortality
Hyyppa & Maki	2001	Individual-level relationships between social capital and self-rated health in a bilingual community	Preventive Medicine	Correlations only reported between singing in a choir and self-rated health
Hyyppa et al	2007	Individual-level measures of social capital as predictors of all-cause and cardiovascular mortality: A population-based prospective study of men and women in Finland	European Journal of Epidemiology	Music participation only analyzed as a group, not stand alone, variable
Lee et al.	2010	Comparisons of leisure-time physical activity and cardiorespiratory fitness as predictors of all-cause mortality in men and women	British Journal of Sports Medicine	No independent examination of performing arts participation – only analyzed as grouped 'physical activity' variable
Merom et al.	2014	Swimming and other sporting activities and the rate of falls in older men: Longitudinal findings from the concord health and ageing in men project	American Journal of Epidemiology	No independent examination of performing arts participation – only analyzed as grouped 'moderate activity' variable
Moisan, Meyer, Gingras	1991	Leisure physical activity and age at menarche	Medicine and Science in Sports and Exercise	Dancing only presented as grouped variable with gymnastics, figure skating, synchronized swimming, diving
Peeters et al.	2010	Which types of activities are associated with risk of recurrent falling in older persons	The Journals of Gerontology	No examination of performing arts participation
Tanasescu et al.	2002	Exercise type and intensity in relation to coronary heart disease in men	JAMA	Performing arts participation not analyzed
Vaisto et al	2014	Physical activity and sedentary behavior in relation to cardiometabolic risk in children: Cross-sectional findings from the Physical Activity and Nutrition in Children (PANIC) Study	International Journal of Behavioral Nutrition and Physical Activity	Examination of general cardiometabolic factors, not noncommunicable disease risk (i.e. metabolic syndrome)

Table S17. Details of all included mean heart rate data from included studies of dance participation.

	Dance style	Type of performance	Mean Heart Rate (% max)	Authors	Year
**Very Light (<57% max)**	Active Video Game Dance (DDR)	Laboratory (30 min)	50.5%	Sell, Lillie & Taylor	2008
	Active Video Game Dancing (Dance Central)	Laboratory (unspecified duration; 3 songs	52.4%	Bronner et al.	2016
	Active Video Game Dancing (DDR)	Laboratory (30 min)	54.5%	Rauber et al.	2018
	Active Video Game Dance (DDR)	Laboratory (15 min - level 1)	54.6%	Graf et al.	2009
	Active Video Game Dancing (Dance Central)	Laboratory (unspecified duration; 3 songs	54.8%	Bronner et al.	2016
	Modern Dance	Rehearsal (unspecified duration)	56.4%	Wyon et al.	2004
	Active Video Game Dance (JustDance)	Laboratory (3 songs - unspecified duration)	56.2 - 57.8%	Jin	2015
**Light (57-63% max heart rate)**	Active Video Game Dance (Kinect Dance Central)	Laboratory (13 min)	57.1%	Bronner, Pinsker & Noah	2013
	Active Video Game Dance (JustDance)	Laboratory (10 min)	57.7%	Marks, Rispen & Calara	2015
	Active Video Game Dance (JustDance)	Laboratory (3 songs - unspecified duration)	56.2 - 57.8%	Jin	2015
	Modern	Class (90 min)	58.1%	Wyon et al.	2002
	Active Video Game Dancing (Dance Central)	Laboratory (unspecified duration; 3 songs	58.2%	Bronner et al.	2016
	Modern Dance	Rehearsal (unspecified duration)	58.5%	Wyon et al.	2004
	Waltz	Class (5 min)	59.0%	Nelson et al.	2011
	Fox trot	Class (5 min)	59.2%	Nelson et al.	2011
	Ballet	Class (90 min)	59.4%	Sanders et al.	2019
	Rhumba	Class (4 min)	59.5%	Nelson et al.	2011
	Active Video Game Dance (using DDR-style mat)	Laboratory (10 min - level 1)	59.5%	Fawkner et al	2010
	Salsa	Class (60 min)	59.6%	Emerenziani et al	2013
	Active Video Game Dancing (Dance Central)	Laboratory (15 min)	59.7%	Smallwood et al.	2012
	Tango	Class (5 min)	59.8%	Nelson et al.	2011
	Active Video Game Dance (Wii Just Dance 3)	Laboratory (21 min)	60.3%	Bronner, Pinsker & Noah	2013
	Active Video Game Dance	Laboratory (4 min)	60.6%	Oh et al.	2017
	Merengue	Class (4 min	60.9%	Nelson et al.	2011
	Modern Dance	Class (unspecified duration)	61.1%	Wyon et al.	2004
	Active Video Game Dancing (Dance Central)	Laboratory (unspecified duration; 3 songs	61.2%	Bronner et al.	2016
	Ballet	Class (24 minutes - barre exercises)	61.4%	Cohen et al	1982
	Modern	Class (90 min)	61.6%	Wyon et al.	2002
	Modern Dance	Class (unspecified duration)	61.6%	Wyon et al.	2004
	Mixed ('Dancing Classrooms')	Class (50 - 80 min)	61.6%	Pennington & Nelson	2020
	Active Video Game Dance (DDR)	Laboratory (15 min - level 2)	61.6%	Graf et al.	2009
	Active Video Game Dance (DDR)	Laboratory (30 min)	62.1%	Kraft et al.	2011
	Modern Dance	Class (90 min)	62.4%	Sanders et al.	2019

	Active Video Game Dance (using DDR-style mat)	Laboratory (10 min - level 2)	63.1%	Fawkner et al	2010
**Moderate (64-76% max heart rate)**	Fun Dance	Class (6 min)	64.0%	Nelson et al.	2011
	Active Video Game Dance (DDR)	Laboratory (12 min)	64.1%	Unnithan, Houser & Fernhall	2006
	Mixture - 'specially designed to develop functional and fitness elements that tend to naturally deteriorate with the aging process'	Class (60 minute)	64.7%	Rodrigues-Krause et al.	2018
	Salsa	Class (60 min - 'rueda de casino' style)	64.7%	Emerenziani et al	2013
	Salsa	Nightclub (60 min)	65.3%	Emerenziani et al	2013
	Salsa	Nightclub (60 min)	65.7%	Emerenziani et al	2013
	Line dancing	Class (~90 min)	65.8%	Zan, Hannon & Carson	2009
	Hip-hop - 'New style'	Laboratory (unspecified duration - routine 1)	65.8%	Wyon et al.	2018
	Maori haka	Laboratory (5 min)	66.6%	Zhu et al	2018
	Active Video Game Dance (Dance game that comes with Xbox 360)	Laboratory (8 min)	66.6%	Canabrava et al.	2018
	Tongan	Laboratory (5 min)	66.7%	Zhu et al	2018
	Salsa	Class (60 min)	67.4%	Emerenziani et al	2013
	Active Video Game Dance (Wii DDR)	Laboratory (9 min)	67.6%	Bronner, Pinsker & Noah	2013
	Latin	Laboratory (15 min)	67.9%	Domene & Easton	2014
	Active Video Game Dance (using DDR-style mat)	Laboratory (10 min - level 3)	68.0%	Fawkner et al	2010
	Zumba (video instructor)	Laboratory (60 min)	68.0%	Schneekloth & Brown	2018
	Active Video Game Dance (JustDance - Xbox Kinect)	Laboratory (60 min)	68.2%	Eason et al.	2016
	Maori poi balls	Laboratory (5 min)	68.3%	Zhu et al	2018
	Modern Dance	Dress rehearsal (unspecified duration)	68.9%	Wyon et al.	2004
	Disco	Party (6 min)	69.4%	Leger	1982
	Modern	Class (90 min)	69.4%	Wyon et al.	2002
	Modern Dance	Dress rehearsal (unspecified duration)	69.9%	Wyon et al.	2004
	Disco	Party (6 min)	70.3%	Leger	1982
	Active Video Game Dance (DDR)	Laboratory (10 min - maximum possible difficulty for each individual)	70.3%	Tan et al.	2002
	Ballet	Class (24 minutes - barre exercises)	70.4%	Cohen et al	1982
	Active Video Game Dance (JustDance)	Laboratory (15 min - Playstation)	70.4%	Powers et al.	2016
	Active Video Game Dance (JustDance)	Laboratory (15 min - Wii)	70.4%	Powers et al.	2016
	Aerobic Dance	Laboratory (3 min - level 1	70.8%	Thomsen & Ballor	1991
	Active Video Game Dance	Laboratory (20 min)	71.1%	Cakir-Atabek, Aygun & Dokumaci	2020
	Ballet	Performance (unspecified duration)	71.2%	Seliger et al	1970
	Active Video Game Dance (Zumba Fitness Rush)	Laboratory (50 min - multiplayer mode)	71.2%	Giancotti et al	2018

	Pole Dancing	Class (min)	71.3%	Nicholas et al.	2019
	Swing	Class (5 min)	71.6%	Nelson et al.	2011
	Aerobic Dance	Laboratory (3 min - level 1	71.7%	Thomsen & Ballor	1991
	Active Video Game Dance (Dance Central 3)	Laboratory (15 min	71.7%	Aygun, Dokumaci & Cakir	2018
	Ballet	Class (7 minutes - center floor exercises)	72.0%	Cohen et al	1982
	Hawaiian Hula	Laboratory (5 min)	72.3%	Zhu et al	2018
	Unspecified	Dance sequence (4 min - timepoint 3)	72.5%	Beck, Wyon & Redding	2018
	Salsa	Class (60 min - 'rueda de casino' style)	72.6%	Emerenziani et al	2013
	Active Video Game Dance (JustDance)	Laboratory (15 min - Xbox Kinect)	72.6%	Powers et al.	2016
	Aerobic dance	Laboratory (15 min - low impact)	72.8%	Rousanoglou & Boudolos	2005
	Active Video Game Dance (Kinect Just Dance 3)	Laboratory (21 min)	73.0%	Bronner, Pinsker & Noah	2013
	Dance Fitness Class	Laboratory (60 min)	73.1%	Eason et al.	2016
	Fijian	Laboratory (5 min)	73.7%	Zhu et al	2018
	Active Video Game Dance (Zumba Fitness Rush)	Laboratory (50 min - single player mode)	73.9%	Giancotti et al	2018
	Unspecified	Dance sequence (4 min - timepoint 2)	74.0%	Beck, Wyon & Redding	2018
	Unspecified	Dance sequence (4 min - timepoint 3)	74.3%	Beck, Wyon & Redding	2018
	Zumba - DVD	Home workout (60 min)	74.5%	Delextrat & Neupert	2016
	Ballet	Class (30 minute)	75.6%	Rodrigues-Krause et al.	2014
	Zumba	Class (60 min)	75.9%	Strejcova et al	2013
	Aerobic bench stepping	Class (60 minute)	76.2%	Rixon, Rehor & Bemben	2006
	Samoan sasa	Laboratory (5 min)	76.3%	Zhu et al	2018
	Ballet - Plies	Laboratory (3 min)	76.3%	Rodrigues-Krause	2014
	Unspecified	Dance sequence (4 min - timepoint 1)	76.4%	Beck, Wyon & Redding	2018
**Vigorous (77-95% max heart rate)**	Ballet - Tendus	Laboratory (2 min)	76.7%	Rodrigues-Krause	2014
	Highland Dance	Class (77 min)	77.1%	Bailie, Wyon & Head	2007
	Zumba - class	Class (60 min)	77.7%	Delextrat & Neupert	2016
	Unspecified	Dance sequence (4 min - timepoint 2)	78.2%	Beck, Wyon & Redding	2018
	Ballet - Grand Battement	Laboratory (1 min)	78.3%	Rodrigues-Krause	2014
	Musical Theater (dance + singing)	Laboratory (4 min)	78.5%	Stephens & Wyon	2020
	Aerobic Dance	Laboratory (3 min - level 1	78.8%	Thomsen & Ballor	1991
	Hula (low intensity)	Laboratory (12-16 min)	78.8%	Usgawa et al	2014
	Samoan slap	Laboratory (5 min)	78.8%	Zhu et al	2018
	Step Aerobic Dance	Class (20 min - aerobic portion)	79.0%	Forte et al.	2001
	Unspecified	Dance sequence (4 min - timepoint 1)	79.1%	Beck, Wyon & Redding	2018

Aerobic Dance	Laboratory (3 min - level 2)	79.5%	Thomsen & Ballor	1991
Musical Theater (dance only)	Laboratory (4 min)	80.0%	Stephens & Wyon	2020
Sardinian folk dance (ballu sardu)	Laboratory (14 min)	80.2%	Cugusi et al.	2015
Aerobic dance	Class (18 min)	80.3%	Grant et al	2002
Ballet	Class (7 minutes - center floor exercises)	80.4%	Cohen et al	1982
Ballet - Rond de Jambes	Laboratory (3 min)	80.8%	Rodrigues-Krause	2014
Aerobic dancing	Laboratory (10 min)	80.8%	Carroll, Otto & Wygand	1991
Zumba (human instructor)	Class (60 min)	81.0%	Schneekloth & Brown	2018
Sports Dancing	Laboratory (10 min)	81.1%	Uspuriene & Cepulenas	2012
Aerobic dance - march	Laboratory (6 min)	81.2%	Darby, Browder & Reeves	1995
Hip-hop - 'New style'	Laboratory (unspecified duration - routine 2)	81.3%	Wyon et al.	2018
Aerobic Dance	Class (30 min)	81.5%	Sekulic, Rausavljevic & Zenic	2006
Aerobic dance (low impact, vigorous arms)	Laboratory (3 min)	81.6%	Schaeffer-Gerschutz, Darby & Browder	2000
Aerobic dance (low impact, no arm involvement)	Laboratory (3 min)	81.6%	Schaeffer-Gerschutz, Darby & Browder	2000
Active Video Game Dance (StepMania)	Laboratory (9 min)	81.8%	Bronner, Pinsker & Noah	2013
Ballet	Performance (unspecified duration)	82.1%	Seliger et al	1970
Tahitian	Laboratory (5 min)	82.2%	Zhu et al	2018
Aerobic dancing	Laboratory (10 min - 6 in bench)	82.4%	Grier et al.	2002
Active Video Game Dance (Dance Central 3)	Laboratory (15 min)	82.6%	Aygun, Dokumaci & Cakir	2018
Aerobic dance	Laboratory (15 min - high impact)	82.6%	Rousanoglou & Boudolos	2005
Sports Dancing	Laboratory (10 min)	82.7%	Uspuriene & Cepulenas	2012
Ballet - Jetes	Laboratory (2 min)	83.4%	Rodrigues-Krause	2014
Active Video Game Dance (DDR)	Laboratory (30 min)	83.4%	Sell, Lillie & Taylor	2008
Aerobic Dance	Class (30 min)	84.1%	Sekulic, Rausavljevic & Zenic	2006
Aerobic Dance	Laboratory (3 min - level 2)	84.2%	Thomsen & Ballor	1991
Aerobic Dance	Class (40 min)	84.3-85.9%	Rockefeller & Burke	1979
Aerobic bench stepping	Laboratory (20 min)	84.7%	Lowe et al	2010
Aerobic Dance	Class (55 min)	84.7%	De Angelis et al.	1998
Step Aerobic Dance	Class (30 min - central portion (no warm up / cool down))	85.4%	La Torre et al.	2005
Ballet - Fondus	Laboratory (3 min)	85.9%	Rodrigues-Krause	2014
Aerobic dance - jumping jack	Laboratory (6 min)	85.9%	Darby, Browder & Reeves	1995
Aerobic (high & low impact)	Laboratory (25 min - 5 min warmup, 15 min session, 5 min cool down)	86.1%	Thompson et al.	1991
Aerobic dance - jog	Laboratory (6 min)	86.4%	Darby, Browder & Reeves	1995
Aerobic Dance	Laboratory (3 min - level 3)	86.5%	Thomsen & Ballor	1991

Aerobic dance (low impact)	Class (40 min)	86.5%	Tarrant & McNaughton	1997
Aerobic dance (low impact)	Laboratory (9 min)	86.7%	Bronstein et al	1990
Ballet - Temps Leves	Laboratory (1 min)	87.0%	Rodrigues-Krause	2014
Musical Theater (dance only)	Laboratory (4 min)	87.0%	Stephens & Wyon	2020
Polish folk dancing	Laboratory (8 min)	87.6%	Maciejczyk & Fec	2013
Highland Dance	Rehearsal (unspecified duration)	87.6%	Bailie, Wyon & Head	2007
Aerobic dancing	Laboratory (10 min - 8 in bench)	87.7%	Grier et al.	2002
Aerobic dance - power jack	Laboratory (6 min)	88.0%	Darby, Browder & Reeves	1995
Ballroom - 'Latin sequence'	Laboratory (unspecified duration - 'competition simulation')	88.1%	Blanksby & Reidy	1988
Aerobic Dance	Laboratory (24 min)	88.2%	Schaeffer et al.	1995
Tap dance	Laboratory (36 min)	88.5%	Oliveira et al.	2010
Aerobic Dance	Laboratory (3 min - level 3)	88.6%	Thomsen & Ballor	1991
Ballet - Grant Adage	Laboratory (2 min)	88.7%	Rodrigues-Krause	2014
Ballroom - 'Modern sequence'	Laboratory (unspecified duration - 'competition simulation')	89.1%	Blanksby & Reidy	1988
Musical Theater (dance + singing)	Laboratory (4 min)	89.1%	Stephens & Wyon	2020
Aerobic dance (high impact, vigorous arms)	Laboratory (3 min)	90.0%	Schaeffer-Gerschutz, Darby & Browder	2000
Ballroom - 'Modern sequence'	Laboratory (unspecified duration - 'competition simulation')	90.2%	Blanksby & Reidy	1988
Ballet	Rehearsal (30 minute)	90.6%	Rodrigues-Krause et al.	2014
Sports Dancing (standard)	Laboratory (10 min)	90.6%	Liiv et al.	2014
Sport Dancing (standard)	Laboratory (10 min)	90.8%	Bria et al.	2011
Aerobic Dance	Laboratory (3 min - level 2)	91.3%	Thomsen & Ballor	1991
Swedish folk dance (hambo)	Laboratory (4 min)	91.4%	Wigaeus & Kilbom	1980
Hula (high intensity)	Laboratory (12-16 min)	91.5%	Usgawa et al	2014
Sports Dancing (standard)	Laboratory (10 min)	91.8%	Liiv et al.	2014
Sports Dancing (Ten Dance)	Laboratory (10 min)	91.9%	Liiv et al.	2014
Tinikling (traditional Filipino dance)	Laboratory (5 min)	92.2%	Heil et al.	2019
Ballroom - 'Latin sequence'	Laboratory (unspecified duration - 'competition simulation')	92.3%	Blanksby & Reidy	1988
Sports Dancing (Ten Dance)	Laboratory (10 min)	92.5%	Liiv et al.	2014
Sport Dancing (standard)	Laboratory (10 min)	92.6%	Bria et al.	2011
Hip-hop - Breakdancing	Laboratory (unspecified duration - set 2)	92.7%	Wyon et al.	2018
Polish folk dancing	Laboratory (8 min)	93.1%	Maciejczyk & Fec	2013
Aerobic dance (high impact, no arm involvement)	Laboratory (3 min)	93.1%	Schaeffer-Gerschutz, Darby & Browder	2000
Hip-hop - Breakdancing	Laboratory (unspecified duration - set 1)	93.2%	Wyon et al.	2018
Aerobic Dance	Laboratory (3 min - level 3)	94.2%	Thomsen & Ballor	1991

Sports Dancing (Latin)	Laboratory (10 min)	94.7%	Liiv et al.	2014
Swedish folk dance (hambo)	Laboratory (4 min)	94.8%	Wigaeus & Kilbom	1980
Sports Dancing	Laboratory (10 min)	95.0%	Pilch et al.	2017
Sport Dancing (Latin)	Laboratory (10 min)	95.3%	Bria et al.	2011
Sport Dancing (Latin)	Laboratory (10 min)	95.5%	Bria et al.	2011
Highland Dance	Performance (unspecified duration)	99.0%	Bailie, Wyon & Head	2007
Sports Dancing (Latin)	Laboratory (10 min)	99.4%	Liiv et al.	2014

Table S18. Details of all included mean heart rate data from included studies of music participation.

	Instrument/singing	Type of performance	Mean heart rate (% max)	Author	Year
Very Light (<57% max heart rate)	Trumpet	Seated trumpet exercises	42.3-44.5%	Robertson, Federoff & Eisensmith	2010
	Trumpet	Seated trumpet exercises (1 min each)	47.2-53.5%	Robertson, Federoff & Eisensmith	2010
	Strings (n=23)	Rehearsal	47.7%	Inesta et al	2008
	Varied instruments in orchestra	Concert 2 - rehearsal	49.2%	Mulcahy et al.	1990
	Stringed instrument	Playing in experimental conditions for 60 mins	49.4%	Horswill, Kien & Zipf	1995
	Marching band - light instruments (<5 pounds)	Rehearsal (75 min)	49.7-50.6%	Strand & Sommer	2005
	Piano (n=10)	Rehearsal	49.8%	Inesta et al	2008
	Varied instruments in orchestra	Concert 1 - rehearsal	50.7%	Mulcahy et al.	1990
	Varied instruments in orchestra	Rehearsal ('private session')	51.1%	Studer et al.	2014
	Singing/flute	Rehearsal	51.6%	Harmat & Theorell	2010
	Band' instruments - i.e. guitar/bass/drums/vocals/keys/violin/trumpet/saxophone	Rehearsal	52.1%	Vellers, Irwin & Lightfoot	2015
	Winds (n=25)	Rehearsal	53.2%	Inesta et al	2008
	Singing/flute	Rehearsal	54.2%	Harmat & Theorell	2010
	Varied instruments in orchestra	Concert 1 - performance	54.3%	Mulcahy et al.	1990
	Percussion - drum corps (battery & front ensemble)	Rehearsal - 96 BPM	54.4%	Dye & Barry	2016
	Marching band - heavy instruments (5 pounds or greater)	Rehearsal (75 min)	54.6-55.5%	Strand & Sommer	2005
	Varied instruments in orchestra	Concert 2 - performance	55.2%	Mulcahy et al.	1990
	Classical Indian Music (n=2)	Performance	56.7%	Inesta et al	2008
Light (57-63% max heart rate)	Strings (n=23)	Performance	58.2%	Inesta et al	2008
	Band' instruments - i.e. guitar/bass/drums/vocals/keys/violin/trumpet/saxophone	Performance	59.1%	Vellers, Irwin & Lightfoot	2015
	Percussion (n=2	Performance	59.3%	Inesta et al	2008
	Percussion - drum corps (battery & front ensemble)	Rehearsal - 176 BPM	60.5%	Dye & Barry	2016
	Clarinet	1st Movement - Repertoire performance (Schumann, Phantasiestucke - 3 movements of increasing intensity)	60.6%	Hahnengress & Boning	2010
	Winds (n=25)	Performance	62.3%	Inesta et al	2008
	Operetta	Performance	62.5%	Seliger et al	1970
	Clarinet	2nd Movement - Repertoire performance (Schumann, Phantasiestucke - 3 movements of increasing intensity)	62.7%	Hahnengress & Boning	2010
	Trumpet	Laboratory (etude - undefined length)	61.0-67.7%	Hunsaker LA	1994
Moderate (64-76% max heart rate)	Singing/flute	Performance	64.0%	Harmat & Theorell	2010
	Varied instruments in orchestra	Performance ('public session')	65.8%	Studer et al.	2014
	Clarinet	3rd Movement - Repertoire performance (Schumann, Phantasiestucke - 3 movements of increasing intensity)	66.9%	Hahnengress & Boning	2010

	Trumpet	Laboratory (etude - undefined length)	61.0-67.7%	Hunsaker LA	1994
	Opera	Performance	68.6%	Seliger et al	1970
	Singing/flute	Performance	68.7%	Harmat & Theorell	2010
	Opera	Performance	70.2%	Seliger et al	1970
	Bagpipes	Laboratory (5 min)	70.2-80.4%	Barr et al	2000
	Conductor (opera)	Performance	72.3%	Seliger et al	1970
	Piano (n=10)	Performance	73.7%	Inesta et al	2008
	Marching band - average of wind, percussion & flag carrier	Parade performance (132 steps/min; 50 min)	75.4%	Erdmann et al	2003
	Drum set	8 metal songs in lab	76.7%	Romero et al.	2016
**Vigorous (77-95% max heart rate)**	Operetta	Performance	77.4%	Seliger et al	1970
	Musical theater (singing & dance)	Laboratory (4 min)	78.5%	Stephens & Wyon	2020
	Bagpipes	Laboratory (5 min)	70.2-80.4%	Barr et al	2000
	Drum set	Concert (mean duration 38.6 min)	87.8%	De La Rue et al.	2013
	Musical theater (singing & dance)	Laboratory (4 min)	89.1%	Stephens & Wyon	2020

Table S19. Studies of the physical demands of performing arts participation excluded after full-text review, with reasons.

Author	Year	Title	Journal	Music/Dance	Reason for Exclusion
Adams	2009	High School Physical Education Students' Heart Rates During Different Activities	The Journal of Physical Education, Recreation & Dance	Dance	No reporting of mean HR during dance performance
Altamirano-Diaz et al	2019	Are active video games effective in eliciting at least moderate intensity physical activity in children and do children enjoy playing them?	Canadian Journal of Cardiology	Dance	No reporting of HR during dance performance
Angioi et al	2009	Fitness in contemporary dance: A systematic review	International Journal of Sports Medicine	Dance	Review article, no reporting of raw HR data
Bailey & McInnis	2011	Energy cost of exergaming: a comparison of the energy cost of 6 forms of exergaming	Archives of Pediatrics & Adolescent Medicine	Dance	No reporting of mean HR during dance performance
Bell & Bassey	1994	A comparison of the relation between oxygen uptake and heart rate during different styles of aerobic dance and a traditional step test in women	European Journal of Applied Physiology	Dance	No reporting of mean HR, only peak HR
Blessing et al.	1987	The physiologic effects of eight weeks of aerobic dance with and without hand-held weights	American Journal of Sports Medicine	Dance	No reporting of mean HR during dance performance
Blessing, Tucker & Williford	1987	Training factors and physical fitness among aerobic dance instructors.	Perceptual & Motor Skills	Dance	Incompatible data, reporting only of %HR max using experimentally determined max HR
Blyth & Goslin	1985	Cardiorespiratory responses to "aerobic dance"	Journal of Sports Medicine & Physical Fitness	Dance	No article access
Cain et al.	2015	Physical activity in youth dance classes	Pediatrics	Dance	No reporting of mean HR during dance performance
Campos et al.	2020	Evaluation of the exercise intensity generated by active video gaming in patients with cystic fibrosis and healthy individuals	Journal of Cystic Fibrosis	Dance	No reporting of mean HR during dance performance
Cheng & Zheng	1997	Study on the training load of aerobic dance in men's individual event	Journal of Guangzhou Physical Education Institute	Dance	Article in Mandarin
Chernozub et al.	2018	The influence of dance and power fitness loads on the body morphometric parameters and peculiarities of adaptive-compensatory reactions of organism of young women	Journal of Physical Education & Sport	Dance	No reporting of mean HR during dance performance
Chren, Spanik & Kyselovicova	2010	Blood lactate concentration of ballroom dancers according to the length of their routines	Acta Facultatis Educationis Physicae Universitatis Comenianae	Dance	No reporting of HR during dance performance
Cohen	1984	Dance - aerobic and anaerobic	Journal of Physical Education, Recreation & Dance	Dance	Review article, no reporting of raw HR data
Cohen, Segal & McArdle	1982	Heart rate response to ballet stage performance	Physician and Sportsmedicine	Dance	No synthesis of HR data, reporting of selected individual data only
Cossette et al	2008	Chest wall dynamics and muscle recruitment during professional flute playing	Respiratory Physiology and Neurobiology	Music	No reporting of HR during music performance
Dahlstrom	1997	Physical effort during dance training: a comparison between teachers and students	Journal of Dance Medicine & Science	Dance	No reporting of mean HR during dance performance
Dahlstrom et al	1996	Physical fitness and physical effort in dancers: a comparison of four major dance styles	Impulse: International Journal of Dance Science, Medicine & Education	Dance	No reporting of mean HR during dance performance
Dahlstrom et al.	1990	Discrepancy between estimated energy intake and requirement in female dancers	Clinical Physiology	Dance	No reporting of mean HR during dance performance
D'Angelo	1994	The effect of foot articulation on exercise intensity during aerobic dance	Thesis	Dance	Study of isolated individual aerobic dance movements (march stepping) only

Domene et al.	2014	Physiological and perceptual responses to Latin partnered social dance	Human Movement Science	Dance	No reporting of mean HR during dance performance
Domene et al.	2016	Salsa dance and Zumba fitness: Acute responses during community-based classes	Journal of Sport and Health Science	Dance	Incompatible data, reporting only of %HRmax & %HRR using laboratory measured HR max values
D'Ottavio et al.	2016	Energy Expenditure in Professional DanceSport	Journal of Dance Medicine & Science	Dance	HR only collected during the last 30 seconds of each dance
Drinkwater & Klopper	2010	Quantifying the physical demands of a musical performance and their effects on performance quality	Medical Problems of Performing Artists	Music	No reporting of HR during music performance
Duan, Zhang & Shao-jun	2010	A Research on the Heart Rates' Variation during the Second Set Performance of the Northern Lion Dance	Journal of Beijing Sport University	Dance	Article in Mandarin
Elliot, Morton & Johnston	1991	Biomechanical and physiological responses to modes of locomotion used in aerobic dance	Australian Journal of Science and Medicine in Sport	Dance	No examination of dancing, only 'aerobic dance locomotion' (walking & jogging)
Engels, Bowen & Wirth	1995	Routine use of external weights during a low-impact aerobic dance conditioning program: Training benefit	Sports Medicine, Training & Rehabilitation	Dance	No reporting of mean HR during dance performance
Evardsen, Ingjer & Bo	2011	Fit women are not able to use the whole aerobic capacity during aerobic dance	Journal of Strength & Conditioning Research	Dance	No reporting of mean HR during dance performance
Froberg et al.	2017	Levels of physical activity during physical education lessons in Sweden	Acta Paediatrica	Dance	No reporting of mean HR during dance performance
Froemel, Oscheutz & Schaller	1999	Heart rate and energy costs during dancing (country dancing) by elderly women	Sportonomics	Dance	No reporting of mean HR during dance performance
Grant et al.	1998	A comparison of physiological responses and rating of perceived exertion between high-impact and low-impact aerobic dance sessions	European Journal of Applied Physiology and Occupational Physiology	Dance	Reporting of % HR max calculated using actual HR max (not predicted) only, no raw HR values reported
Guidetti et al.	2008	Energy cost and energy sources of a ballet dance exercise in female adolescents with different technical ability	European Journal of Applied Physiology	Dance	No reporting of mean HR during dance performance
Guidetti et al.	2007	Exercise intensities during a ballet lesson in female adolescents with different technical ability	International Journal of Sports Medicine	Dance	No reporting of raw HR data (only statistical analyses of HR data)
Hausken & Dyrstad	2016	Using heart rate monitors to assess energy expenditure in four training types	Gazzetta Medica Italiana Archivio per le Scienze Mediche	Dance	Incompatible data, reporting only of %HR max using experimentally determined max HR
Hawley, Williams & Hurley	1990	Physiological and psychological response to aerobic dance classes	New Zealand Journal of Sports Medicine	Dance	No reporting of mean HR during dance performance
Helin	1988	Activation in professional ballet dancers	Physiology & Behavior	Dance	No reporting of HR during dance performance
Hirsch et al	2003	Energy balance and physical activity patterns in university ballet dancers	Journal of Dance Medicine & Science	Dance	No article access + appears to focus on total daily EE (not dance-specific)
Hollis et al.	2017	A systematic review and meta-analysis of moderate-to-vigorous physical activity levels in secondary school physical education lessons	International Journal of Behavioral Nutrition and Physical Activity	Dance	Review article, no reporting of raw HR data
Hunsaker & Ramsey	1998	Cardiac dynamics in marching band trumpet players	Medical Problems of Performing Artists	Music	Reporting of maximal HR data only during performance
Ipsen	1994	Cardiovascular and body composition responses to aerobic dance training of varying frequencies and total program lengths	Thesis	Dance	No reporting of mean HR during dance performance
Jakubec et al	2008	Changes in heart rate variability after a six month long aerobic dance or step-dance program in women 40-65 years old: The influence of different degrees of adherence, intensity and initial levels	Acta Universitatis Palackianae Olomucensis. Gymnica	Dance	No reporting of mean HR during dance performance
Jeffries, Wallace & Coutts	2017	Quantifying Training Loads in Contemporary Dance	International Journal of Sports Physiology and Performance	Dance	No reporting of mean HR during dance performance
Jette & Inglis	1975	Energy cost of square dancing	Journal of Applied Physiology	Dance	No reporting of mean HR during dance performance

Kirkendall & Calabrese	1983	Physiological aspects of dance	Clinics in Sports Medicine	Dance	No reporting of mean HR during dance performance
Kozai et al.	2020	Workload Intensity and Rest Periods in Professional Ballet: Connotations for Injury	International Journal of Sports Medicine	Dance	No reporting of mean HR during dance performance
Lankford et al.	2014	The Energy Expenditure of Recreational Ballroom Dance	International Journal of Exercise Science	Dance	No reporting of mean HR during dance performance
Larger & Ledoux	1996	Dimsdale JE, Nelesen RA. French-horn hypertension. N EnglJ Med 1995; 333: 326-27.	Lancet	Music	Letter to the editor only describing heart response of one note at a time
Liebana et al.	2018	Muscle Activation in the Main Muscle Groups of the Lower Limbs in High-Level Dancesport Athletes	Medical Problems of Performing Artists	Dance	No reporting of mean HR during dance performance
Lira et al.	2018	Acute Caffeine Supplementation Does Not Change Sweat Rate and Blood Pressure in Ballet Dancers: A Double-Blind and Placebo-Controlled Study	Journal of Dance Medicine & Science	Dance	No reporting of HR during dance performance
Lyons et al	2011	Energy expenditure and enjoyment during video game play: differences by game type	Medicine and Science in Sports and Exercise	Dance	No reporting of mean HR during dance performance
Lyons et al.	2014	Engagement, enjoyment and energy expenditure during active video game play	Health Psychology	Dance	No reporting of mean HR during dance performance
Massidda et al	2011	Energy expenditure during competitive Latin American dancing simulation	Medical Problems of Performing Artists	Dance	No reporting of mean HR during dance performance
Matthe et al.	2019	Central retinal venous pressure is higher than intraocular pressure during amateur trumpet playing	Graefe's Archive for Clinical and Experimental Opthalmology	Music	No reporting of HR during music performance
Mavrovouniotis et al	2008	Greek traditional dances and quality of old people's life	Journal of Body and Movement Therapy	Dance	No reporting of mean HR during dance performance
McDonough et al	2018	Comparison of college students' energy expenditure, physical activity, and enjoyment during exergaming and traditional exercise	Journal of Clinical Medicine	Dance	No reporting of mean HR during dance performance
Mengelkock, Highsmith & Morris	2014	Comparison of the Metabolic Demands of Dance Performance Using Three Mobility Devices for a Dancer with Spinal Cord Injury and an Able-Bodied Dancer	Medical Problems of Performing Artists	Dance	Analysis of dance in 'clinical situations' (different types of mobility devices) only
Metcalf	1983	Telemetered heart rate responses in adult men and women aerobic dance instructors	Sports Medicine Digest	Dance	No article access
Metcalf et al.	1981	ECG effects of aerobic dance: A study of five exercise-conditioned young women	Postgraduate Medicine	Dance	No reporting of mean HR during dance performance
Milburn & Butts	1983	A comparison of the training responses to aerobic dance and jogging in college females	Medicine and Science in Sports and Exercise	Dance	Dancing with a 'target heart rate'
Miranda et al	2017	Energy Cost of Active and Sedentary Music Video Games: Drum and Handheld Gaming vs. Walking and Sitting	International Journal of Exercise Science	Music	No reporting of HR during music performance
Morgan	1985	A comparison of the energy requirements of selected dance programs	Physical Educator	Dance	Review article, no reporting of raw HR data
Nelson et al.	1988	Cardiac frequency and caloric cost of aerobic dancing in young women	Research Quarterly for Exercise and Sport	Dance	No reporting of mean HR during dance performance
Noah et al.	2011	Vigorous Energy Expenditure with a Dance Exer-game	Journal of Exercise Physiology Online	Dance	No reporting of mean HR during dance performance
Novak, Magill & Schutte	1978	Maximal oxygen intake and body composition of female dancers	European Journal of Applied Physiology and Occupational Physiology	Dance	No reporting of mean HR during dance performance
Papatzikis & Papatziki	2016	Investigating heart rate and rhythm changes in an infant's music education course: A case study	Psychology of Music	Music	No reporting of HR during music performance
Parker et al.	1989	Failure of target heart rate to accurately monitor intensity during aerobic dance	Medicine and Science in Sports and Exercise	Dance	Aerobic dance performed based on 'target HR

Perry et al.	1988	A comparison of training responses to interval versus continuous aerobic dance	The Journal of Sports Medicine and Physical Fitness	Dance	Article unable to be accessed, insufficient information in abstract
Raymond et al	2005	Biofeedback and dance performance: a preliminary investigation	Applied Psychophysiology and Biofeedback	Dance	No reporting of mean HR, only HR variability
Raynor, Cardoso & Bond	2016	Effect of exposure to greater active videogame variety on time spent in moderate- to vigorous-intensity physical activity	Physiology and Behavior	Dance	No reporting of mean HR during dance performance
Redding et al.	2009	The development of a high intensity dance performance fitness test	Journal of Dance Medicine & Science	Dance	Study of a dance-based fitness test, not dance performance
Redding et al.	2004	Validity of using heart rate as a predictor of oxygen consumption in dance	Journal of Dance Medicine & Science	Dance	No reporting of mean HR during dance performance
Rensing, Schemmann & Zalpour	2018	Musculoskeletal Demands in Violin and Viola Playing: A Literature Review	Medical Problems of Performing Artists	Music	No reporting of HR during music performance
Rice, Gurchiek & McBride	2018	Physiological and Biomechanical Responses to an Acute Bout of High Kicking in Dancers	Journal of Strength & Conditioning Research	Dance	Analysis of fitness, not dance, routine
Rimmer, Jay & Plowman	1994	Physiological characteristics of trained dancers and intensity level of ballet class and rehearsal	Impulse: International Journal of Dance Science, Medicine & Education	Dance	No reporting of mean HR during dance performance
Rinne, Miilunpalo & Heinonen	2007	Evaluation of required motor abilities in commonly practiced exercise modes and potential training effects among adults	Journal of Physical Activity & Health	Dance	No reporting of mean HR during dance performance
Rokka, Mavridis & Kouli	2010	The impact of exercise intensity on mood state of participants in dance aerobics programs	Studies in Physical Culture & Tourism	Dance	No reporting of mean HR during dance performance
Roopchand-Martin et al.	2015	A pilot study using the XBOX Kinect for exercise conditioning in sedentary female university students	Technology and Healthcare	Dance	No reporting of mean HR during dance performance
Schantz & Astrand	1984	Physiological characteristics of classical ballet	Medicine and Science in Sports and Exercise	Dance	No reporting of mean HR during dance performance
Scharff-Olson, Williford & Smith	1992	The heart rate VO2 relationship of aerobic dance: A comparison of target heart rate methods	Journal of Sports Medicine & Physical Fitness	Dance	No reporting of mean HR during dance performance
Shi et al	2001	Preliminary study of physiological characteristics of dragon dance	Journal of Beijing Sport University	Dance	Article in Mandarin
Shimzu et al.	2012	Catecholamine responses to 6h extended daily practice of marching band activities as a performing art	Journal of Science and Medicine in Sport	Music	No reporting of HR during music performance
Silva et al.	2012	Analysis of Acute Cardiovascular Responses in Experienced Practitioners of Capoeira: A Brazilian Art Form	Journal of Exercise Physiology Online	Dance	No reporting of HR during dance performance
Silva et al.	2013	Heart Rate Responses During and After the Practice of Capoeira: A Brazilian Art Form -- Part II	Journal of Exercise Physiology Online	Dance	No reporting of mean HR during dance performance
Skarsem Reitlo et al.	2017	Exercise patterns in older adults randomized to moderate-or high-intensity training: The Generation 100 study	European Geriatric Medicine	Dance	No reporting of mean HR during dance performance
Surgenor & Wyon	2019	Measuring Training Load in Dance: The Construct Validity of Session-RPE	Medical Problems of Performing Artists	Dance	No reporting of mean HR during dance performance
Taskin & Vardar Yagli	2019	An evaluation of physical activity level, respiratory muscle endurance and pain relationship in professional dancers	Turkish Journal of Physiotherapy and Rehabilitation	Dance	No reporting of mean HR during dance performance
Taylor et al	2011	Activity-promoting gaming systems in exercise and rehabilitation	Journal of Rehabilitation Research and Development	Dance	No reporting of HR during dance performance
Trost, Drovandi & Pfeiffer	2016	Developmental Trends in the Energy Cost of Physical Activities Performed by Youth	Journal of Physical Activity & Health	Dance	No reporting of mean HR during dance performance
Tucker, Faulkner & Horvath	1971	Electrocardiography and lung function in brass instrument players	Archives of Environmental Health	Music	HR data only for brief arpeggios

Vaczi et al.	2016	Ballroom dancing is more intensive for the female partners due to their unique hold technique	Physiology International	Dance	No reporting of mean HR, only peak HR
Verhoeven et al	2015	Energy Expenditure During Xbox Kinect Play in Early Adolescents: The Relationship with Player Mode and Game Enjoyment	Games for Health Journal	Dance	No reporting of mean HR during dance performance
Vogelaar	1998	Castrati in western art music, Part 2: Selection and musical training: Physical and psychosocial implications	Medical Problems of Performing Artists	Music	No reporting of HR during music performance
Wanke et al.	2020	Work related cardiovascular load in professional dance teachers-a pilot study	Journal of Occupational Medicine and Toxicology	Dance	Incompatible data, reporting only of %HR max using experimentally determined max HR
Wasley et al.	2012	Influence of fitness and physical activity on cardiovascular reactivity to musical performance	Work	Music	No reporting of HR during music performance
Williford, Scharff-Olson & Blessing	1989	The physiological effects of aerobic dance. A review	Sports Medicine	Dance	No reporting of mean HR during dance performance
Wyon	2005	Cardiorespiratory Training for Dancers	Journal of Dance Medicine & Science	Dance	Review article, no reporting of raw HR data
Yang	2013	Samba fitness efficacy research based on analysis of body compositions changes	Journal of Chemical and Pharmaceutical Research	Dance	No reporting of mean HR during dance performance
Zanchini & Malaguti	2014	Energy requirements in top-level DanceSport athletes	Journal of Human Sport & Exercise	Dance	No reporting of mean HR during dance performance

REFERENCE LIST

Included reviews – Dance

Burkhardt, J. and C. Brennan (2012). "The effects of recreational dance interventions on the health and well-being of children and young people: a systematic review." Arts & Health **4**(2): 148-161.

Clarke, F., et al. (2018). "Balance in theatrical dance performance: a systematic review." Medical Problems of Performing Artists **33**(4): 275-285.

Cugusi, L., et al. (2019). "Zumba Fitness and Women's Cardiovascular Health: A SYSTEMATIC REVIEW." Journal of cardiopulmonary rehabilitation and prevention **39**(3): 153-160.

da Silveira Costa, M. S., et al. (2013). "Static and dynamic balance in ballet dancers: a literature review." Fisioter. Pesqui **20**: 299-305.

Fernández-Argüelles, E. L., et al. (2015). "Effects of dancing on the risk of falling related factors of healthy older adults: a systematic review." Archives of gerontology and geriatrics **60**(1): 1-8.

Hwang, P. W.-N. and K. L. Braun (2015). "The effectiveness of dance interventions to improve older adults' health: a systematic literature review." Alternative therapies in health and medicine **21**(5): 64.

Keogh, J. W., et al. (2009). "Physical benefits of dancing for healthy older adults: a review." Journal of aging and physical activity **17**(4): 479-500.

Letton, M. E., et al. (2020). "The Effectiveness of Classical Ballet Training on Health-Related Outcomes: A Systematic Review." Journal of Physical Activity and Health **17**(5): 566-574.

Liu, X., et al. (2020). "Dance intervention effects on physical function in healthy older adults: a systematic review and meta-analysis." Aging clinical and experimental research: 1-11.

Meng, X., et al. (2020). "Effects of dance intervention on global cognition, executive function and memory of older adults: a meta-analysis and systematic review." Aging clinical and experimental research **32**(1): 7-19.

Rodrigues-Krause, J., et al. (2016). "Effects of dance interventions on cardiovascular risk with ageing: systematic review and meta-analysis." Complementary Therapies in Medicine **29**: 16-28.

Rodrigues-Krause, J., et al. (2019). "Dancing for Healthy Aging: Functional and Metabolic Perspectives." Alternative Therapies in Health & Medicine **25**(1).

Teixeira-Machado, L., et al. (2019). "Dance for neuroplasticity: A descriptive systematic review." Neuroscience & Biobehavioral Reviews **96**: 232-240.

Veronese, N., et al. (2017). "Dance movement therapy and falls prevention." Maturitas **102**: 1-5.

Yan, A. F., et al. (2018). "The effectiveness of dance interventions on physical health outcomes compared to other forms of physical activity: a systematic review and meta-analysis." Sports Medicine **48**(4): 933-951.

Included Reviews – Music

Butzlaff, R. (2000). "Can music be used to teach reading?" Journal of aesthetic education **34**(3/4): 167-178.

Clift, S., et al. (2010). "Group singing, wellbeing and health: A systematic mapping of research evidence." Unesco Observatory **2**(1).

Coffey, E. B., et al. (2017). "Speech-in-noise perception in musicians: A review." Hearing research **352**: 49-69.

Daykin, N., et al. (2013). "Music making for health, well-being and behaviour change in youth justice settings: a systematic review." Health promotion international **28**(2): 197-210.

Daykin, N., et al. (2018). "What works for wellbeing? A systematic review of wellbeing outcomes for music and singing in adults." Perspectives in Public Health **138**(1): 39-46.

Eells, K. (2014). "The use of music and singing to help manage anxiety in older adults." Mental Health Practice **17**(5).

Fancourt, D., et al. (2014). "The psychoneuroimmunological effects of music: A systematic review and a new model." Brain, behavior, and immunity **36**: 15-26.

Gick, M. L. (2011). "Singing, health and well-being: A health psychologist's review." Psychomusicology: Music, Mind and Brain **21**(1-2): 176.

Gordon, R. L., et al. (2015). "Does music training enhance literacy skills? A meta-analysis." Frontiers in psychology **6**: 1777.

Hetland, L. (2000). "Learning to make music enhances spatial reasoning." Journal of aesthetic education **34**(3/4): 179-238.

Jaschke, A. C., et al. (2013). "Music education and its effect on intellectual abilities in children: a systematic review." Reviews in the Neurosciences **24**(6): 665-675.

Moore, K. S. (2013). "A systematic review on the neural effects of music on emotion regulation: implications for music therapy practice." Journal of music therapy **50**(3): 198-242.

Phillips, C. S. and H. Becker (2019). "Systematic Review: Expressive arts interventions to address psychosocial stress in healthcare workers." Journal of advanced nursing **75**(11): 2285-2298.

Raglio, A., et al. (2019). "Music in the workplace: A narrative literature review of intervention studies." Journal of Complementary and Integrative Medicine **1**(ahead-of-print).

Skingley, A. and T. Vella-Burrows (2010). "Therapeutic effects of music and singing for older people." Nursing standard **24**(19).

Talamini, F., et al. (2017). "Musicians have better memory than nonmusicians: A meta-analysis." PloS one **12**(10): e0186773.

Vaughn, K. (2000). "Music and mathematics: Modest support for the oft-claimed relationship." Journal of aesthetic education **34**(3/4): 149-166.

Yap, A. F., et al. (2017). "A systematic review on the effects of active participation in rhythm-centred music making on different aspects of health." European Journal of Integrative Medicine **9**: 44-49.

Included Individual Studies *(from included reviews)* **– Dance**

Adiputra, N., et al. (1996). "Balinese dance exercises improve the maximum aerobic capacity." Journal of human ergology **25**(1): 25.

Alpert, P. T., et al. (2009). "The effect of modified jazz dance on balance, cognition, and mood in older adults." Journal of the American Academy of Nurse Practitioners **21**(2): 108-115.

Alves, H. (2013). Dancing and the aging brain: the effects of a 4-month ballroom dance intervention on the cognition of healthy older adults, University of Illinois at Urbana-Champaign.

Ambegaonkar, J. P., et al. (2013). "Balance comparisons between female dancers and active nondancers." Research quarterly for exercise and sport **84**(1): 24-29.

Araneta, M. and D. Tanori (2014). "Benefits of Zumba Fitness® among sedentary adults with components of the metabolic syndrome: a pilot study." The Journal of sports medicine and physical fitness **55**(10): 1227-1233.

Baniqued, P. L., et al. (2018). "Brain network modularity predicts exercise-related executive function gains in older adults." Frontiers in aging neuroscience **9**: 426.

Barene, S., et al. (2016). "Effects on muscle strength, maximal jump height, flexibility and postural sway after soccer and Zumba exercise among female hospital employees: a 9-month randomised controlled trial." Journal of sports sciences **34**(19): 1849-1858.

Barene, S., et al. (2014). "Soccer and Zumba as health-promoting activities among female hospital employees: a 40-weeks cluster randomised intervention study." Journal of sports sciences **32**(16): 1539-1549.

Barene, S., et al. (2014). "Effects of the workplace health promotion activities soccer and Zumba on muscle pain, work ability and perceived physical exertion among female hospital employees." PloS one **9**(12): e115059.

Belardinelli, R., et al. (2008). "Waltz dancing in patients with chronic heart failure: new form of exercise training." Circulation: Heart Failure **1**(2): 107-114.

Bennell, K., et al. (2000). "Activity-associated differences in bone mineral are evident before puberty: a cross-sectional study of 130 female novice dancers and controls." Pediatric Exercise Science **12**(4): 371-381.

Bennett, C. G. and M. E. Hackney (2018). "Effects of line dancing on physical function and perceived limitation in older adults with self-reported mobility limitations." Disability and rehabilitation **40**(11): 1259-1265.

Blackman, L., et al. (1988). "The effects of dance team participation on female adolescent physical fitness and self-concept." Adolescence **23**(90): 437.

Bronner, S. (2012). "Differences in segmental coordination and postural control in a multi-joint dance movement: developpe arabesque." Journal of dance medicine & science **16**(1): 26-35.

Burgess, G., et al. (2006). "Effects of a 6-week aerobic dance intervention on body image and physical self-perceptions in adolescent girls." Body image **3**(1): 57-66.

Burzynska, A. Z., et al. (2017). "The dancing brain: structural and functional signatures of expert dance training." Frontiers in human neuroscience **11**: 566.

Casabona, A., et al. (2016). "Specificity of foot configuration during bipedal stance in ballet dancers." Gait & posture **46**: 91-97.

Cepeda, C. C., et al. (2015). "Effect of an eight-week ballroom dancing program on muscle architecture in older adult females." Journal of aging and physical activity **23**(4): 607-612.

Chen, Z. (2014). "The Effect of square dance movement intervention on cognitive function in the elderly." Chinese Rural Health Service Administration **34**: 879-881.

Coubard, O. A., et al. (2011). "Practice of contemporary dance improves cognitive flexibility in aging." Frontiers in aging neuroscience **3**: 13.

Crotts, D., et al. (1996). "Balance abilities of professional dancers on select balance tests." Journal of Orthopaedic & Sports Physical Therapy **23**(1): 12-17.

Cruz-Ferreira, A., et al. (2015). "Creative dance improves physical fitness and life satisfaction in older women." Research on aging **37**(8): 837-855.

Cugusi, L., et al. (2015). "Cardiovascular effects, body composition, quality of life and pain after a Zumba fitness program in Italian overweight women." The Journal of sports medicine and physical fitness **56**(3): 328-335.

da Silva Borges, E. G., et al. (2012). "The effect of ballroom dance on balance and functional autonomy among the isolated elderly." Archives of gerontology and geriatrics **55**(2): 492-496.

da Silva Borges, E. G., et al. (2014). "Postural balance and falls in elderly nursing home residents enrolled in a ballroom dancing program." Archives of gerontology and geriatrics **59**(2): 312-316.

Daley, A. J. and J. Buchanan (1999). "Aerobic dance and physical self-perceptions in female adolescents: Some implications for physical education." Research quarterly for exercise and sport **70**(2): 196-200.

Delextrat, A. A., et al. (2016). "An 8-week exercise intervention based on Zumba improves aerobic fitness and psychological well-being in healthy women." Journal of Physical Activity and Health **13**(2): 131-139.

Doi, T., et al. (2017). "Effects of cognitive leisure activity on cognition in mild cognitive impairment: results of a randomized controlled trial." Journal of the American Medical Directors Association **18**(8): 686-691.

Domene, P. A., et al. (2016). "The health-enhancing efficacy of Zumba® fitness: An 8-week randomised controlled study." Journal of sports sciences **34**(15): 1396-1404.

Donath, L., et al. (2014). "The effects of Zumba training on cardiovascular and neuromuscular function in female college students." European Journal of Sport Science **14**(6): 569-577.

Ehlers, D. K., et al. (2017). "Regional brain volumes moderate, but do not mediate, the effects of group-based exercise training on reductions in loneliness in older adults." Frontiers in aging neuroscience **9**: 110.

Eyigor, S., et al. (2009). "A randomized controlled trial of Turkish folklore dance on the physical performance, balance, depression and quality of life in older women." Archives of gerontology and geriatrics **48**(1): 84-88.

Federici, A., et al. (2005). "Does dance-based training improve balance in adult and young old subjects? A pilot randomized controlled trial." Aging clinical and experimental research **17**(5): 385-389.

Ferrufino, L., et al. (2011). "Practice of contemporary dance promotes stochastic postural control in aging." Frontiers in human neuroscience **5**: 169.

Flores, R. (1995). "Dance for health: improving fitness in African American and Hispanic adolescents." Public health reports **110**(2): 189.

Ford Jr, H. T., et al. (1989). "Effects of selected physical activities on health-related fitness and psychological well-being." Psychological Reports **64**(1): 203-208.

Garber, C. E., et al. (1992). "Is aerobic dance an effective alternative to walk-jog exercise training?" The Journal of sports medicine and physical fitness **32**(2): 136-141.

Gerbino, P. G., et al. (2007). "Comparison of standing balance between female collegiate dancers and soccer players." Gait & posture **26**(4): 501-507.

Goertzen, D., et al. (1984). "The functional capacity and physical activity levels of women over 60 years of age." Journal of sports medicine and physical fitness **24**(1): 30-36.

Golomer, E. and P. Dupui (2000). "Spectral analysis of adult dancers' sways: sex and interaction vision-proprioception." International Journal of Neuroscience **105**(1-4): 15-26.

Golomer, E., et al. (1997). "Sex-linked differences in equilibrium reactions among adolescents performing complex sensorimotor tasks." Journal of Physiology-Paris **91**(2): 49-55.

Golomer, E., et al. (1999). "The contribution of vision in dynamic spontaneous sways of male classical dancers according to student or professional level." Journal of Physiology-Paris **93**(3): 233-237.

Golomer, E., et al. (2010). "Right hemisphere in visual regulation of complex equilibrium: the female ballet dancers' experience." Neurological Research **32**(4): 409-415.

Golomer, E., et al. (2009). "Spontaneous whole body rotations and classical dance expertise: How shoulder–hip coordination influences supporting leg displacements." Journal of Electromyography and Kinesiology **19**(2): 314-321.

Granacher, U., et al. (2012). "Effects of a salsa dance training on balance and strength performance in older adults." Gerontology **58**(4): 305-312.

Grant, S., et al. (2002). "A comparison of physiological responses and rating of perceived exertion in two modes of aerobic exercise in men and women over 50 years of age." British Journal of Sports Medicine **36**(4): 276-281.

Guillou, E., et al. (2007). "Dynamic balance sensory motor control and symmetrical or asymmetrical equilibrium training." Clinical Neurophysiology **118**(2): 317-324.

Hackney, M. E., et al. (2015). "Adapted Tango improves mobility, motor–cognitive function, and gait but not cognition in older adults in independent living." Journal of the American Geriatrics Society **63**(10): 2105-2113.

Hackney, M. E., et al. (2007). "Effects of tango on functional mobility in Parkinson's disease: a preliminary study." Journal of neurologic physical therapy **31**(4): 173-179.

Hamacher, D., et al. (2015). "The effect of a six-month dancing program on motor-cognitive dual-task performance in older adults." Journal of aging and physical activity **23**(4): 647-652.

Hashimoto, H., et al. (2015). "Effects of dance on motor functions, cognitive functions, and mental symptoms of Parkinson's disease: a quasi-randomized pilot trial." Complementary Therapies in Medicine **23**(2): 210-219.

Hedgepeth, S. L. C. (1987). The effects of ballet training on isokinetic strength, power, and muscular endurance, Virginia Tech.

Heffron, M., et al. (1997). "Weight-training and bone mass in post-menopausal women." Sports Excercise and Injury **3**(3): 143-149.

Holmerová, I., et al. (2010). "Effect of the exercise dance for seniors (EXDASE) program on lower-body functioning among institutionalized older adults." Journal of aging and health **22**(1): 106-119.

Hopper, D. M., et al. (2014). "The effects of vestibular stimulation and fatigue on postural control in classical ballet dancers." Journal of dance medicine & science **18**(2): 67-73.

Houston, S. and A. McGill (2013). "A mixed-methods study into ballet for people living with Parkinson's." Arts & Health **5**(2): 103-119.

Hugel, F., et al. (1999). "Postural control of ballet dancers: a specific use of visual input for artistic purposes." International journal of sports medicine **20**(02): 86-92.

Hui, E., et al. (2009). "Effects of dance on physical and psychological well-being in older persons." Archives of gerontology and geriatrics **49**(1): e45-e50.

Isler, A. K., et al. (2001). "Effects of step aerobics and aerobic dancing on serum lipids and lipoproteins." Journal of sports medicine and physical fitness **41**(3): 380-385.

Janyacharoen, T., et al. (2013). "Physical performance in recently aged adults after 6 weeks traditional Thai dance: a randomized controlled trial." Clinical interventions in aging **8**: 855.

Jarvis, D. N., et al. (2014). "Trunk coordination in dancers and nondancers." Journal of applied biomechanics **30**(4): 547-554.

Kaltsatou, A. C., et al. (2014). "Functional and psychosocial effects of either a traditional dancing or a formal exercising training program in patients with chronic heart failure: a comparative randomized controlled study." Clinical Rehabilitation **28**(2): 128-138.

Kattenstroth, J.-C., et al. (2013). "Six months of dance intervention enhances postural, sensorimotor, and cognitive performance in elderly without affecting cardio-respiratory functions." Frontiers in aging neuroscience **5**: 5.

Kattenstroth, J.-C., et al. (2011). "Balance, sensorimotor, and cognitive performance in long-year expert senior ballroom dancers." Journal of Aging Research **2011**.

Kattenstroth, J.-C., et al. (2010). "Superior sensory, motor, and cognitive performance in elderly individuals with multi-year dancing activities." Frontiers in aging neuroscience **2**: 31.

Kiefer, A. W., et al. (2011). "Multi-segmental postural coordination in professional ballet dancers." Gait & posture **34**(1): 76-80.

Kilroy, E. A., et al. (2016). "The effect of single-leg stance on dancer and control group static balance." International Journal of Exercise Science **9**(2): 110.

Kim, S. and J. Kim (2007). "Mood after various brief exercise and sport modes: aerobics, hip-hop dancing, ice skating, and body conditioning." Perceptual and motor skills **104**(3_suppl): 1265-1270.

Kim, S.-H., et al. (2011). "Effect of dance exercise on cognitive function in elderly patients with metabolic syndrome: a pilot study." Journal of sports science & Medicine **10**(4): 671.

Kosmat, H. and A. Vranic (2017). "The efficacy of a dance intervention as cognitive training for the old-old." Journal of aging and physical activity **25**(1): 32-40.

Kouli, O., et al. (2009). "The effects of an aerobic program on health-related fitness and intrinsic motivation in elementary school pupils." Studies in Physical Culture & Tourism **16**(3): 301-306.

Krampe, J., et al. (2010). "Dance-based therapy in a program of all-inclusive care for the elderly: an integrative approach to decrease fall risk." Nursing administration quarterly **34**(2): 156-161.

Krishnan, S., et al. (2015). "Zumba® dance improves health in overweight/obese or type 2 diabetic women." American journal of health behavior **39**(1): 109-120.

Krityakiarana, W. and N. Jongkamonwiwat (2016). "Comparison of balance performance between Thai classical dancers and non-dancers." Journal of dance medicine & science **20**(2): 72-78.

Kudlacek, S., et al. (1997). "The impact of a senior dancing program on spinal and peripheral bone mass1." American journal of physical medicine & rehabilitation **76**(6): 477-481.

Kujala, U., et al. (1997). "Training does not increase maximal lumbar extension in healthy adolescents." Clinical biomechanics **12**(3): 181-184.

Lazarou, I., et al. (2017). "International ballroom dancing against neurodegeneration: a randomized controlled trial in Greek community-dwelling elders with mild cognitive impairment." American Journal of Alzheimer's Disease & Other Dementias® **32**(8): 489-499.

Lesser, I. A., et al. (2016). "Effectiveness of exercise on visceral adipose tissue in older South Asian Women." Med Sci Sports Exerc **48**(7): 1371-1378.

Lesté, A. and J. Rust (1990). "Effects of dance on anxiety." American Journal of Dance Therapy **12**(1): 19-25.

Lin, C.-F., et al. (2011). "Comparison of postural stability between injured and uninjured ballet dancers." The American journal of sports medicine **39**(6): 1324-1331.

Lin, C.-W., et al. (2014). "A comparison of ballet dancers with different level of experience in performing single-leg stance on retiré position." Motor control **18**(2): 199-212.

López-Ortiz, C., et al. (2016). "Pilot study of a targeted dance class for physical rehabilitation in children with cerebral palsy." SAGE open medicine **4**: 2050312116670926.

Machacova, K., et al. (2017). "Dance as prevention of late life functional decline among nursing home residents." Journal of Applied Gerontology **36**(12): 1453-1470.

Mangeri, F., et al. (2014). "A standard ballroom and Latin dance program to improve fitness and adherence to physical activity in individuals with type 2 diabetes and in obesity." Diabetology & metabolic syndrome **6**(1): 74.

Marmeleira, J., et al. (2009). "Creative dance can enhance proprioception in older adults." Journal of sports medicine and physical fitness **49**(4): 480-485.

Matthews, B., et al. (2006). "Dancing for bone health: a 3-year longitudinal study of bone mineral accrual across puberty in female non-elite dancers and controls." Osteoporosis International **17**(7): 1043-1054.

Mavridis, G., et al. (2004). "The effect of a health-related aerobic dance program on elementary school children." Journal of Human Movement Studies **47**(4): 337-349.

Mavrovouniotis, F. H., et al. (2010). "Greek traditional dances and quality of old people's life." Journal of Bodywork and movement therapies **14**(3): 209-218.

McKinley, P., et al. (2008). "Effect of a community-based Argentine tango dance program on functional balance and confidence in older adults." Journal of aging and physical activity **16**(4): 435-453.

Merom, D., et al. (2016). "Cognitive benefits of social dancing and walking in old age: the dancing mind randomized controlled trial." Frontiers in aging neuroscience **8**: 26.

Merom, D., et al. (2016). "Social dancing and incidence of falls in older adults: a cluster randomised controlled trial." PLoS medicine **13**(8): e1002112.

Micallef, C. (2014). "The effectiveness of an 8-week Zumba programme for weight reduction in a group of Maltese overweight and obese women." Sport Sciences for Health **10**(3): 211-217.

Milburn, S. and N. K. Butts (1983). "A comparison of the training responses to aerobic dance and jogging in college females." Medicine and Science in Sports and Exercise **15**(6): 510-513.

Moller, A. and Y. Masharawi (2011). "The effect of first ballet classes in the community on various postural parameters in young girls." Physical therapy in sport **12**(4): 188-193.

Müller, P., et al. (2017). "Evolution of neuroplasticity in response to physical activity in old age: the case for dancing." Frontiers in aging neuroscience **9**: 56.

Ozdinc, S. A. and F. N. Turan (2016). "Effects of ballet training of children in Turkey on foot anthropometric measurements and medial longitudinal arc development." J. Pak. Med. Assoc **66**(7): 869-874.

Park, J.-H., et al. (2015). "Effects of a 12-week healthy-life exercise program on oxidized low-density lipoprotein cholesterol and carotid intima-media thickness in obese elderly women." Journal of physical therapy science **27**(5): 1435-1439.

Pérez, R. M., et al. (2014). "Visual availability, balance performance and movement complexity in dancers." Gait & posture **40**(4): 556-560.

Perrin, P., et al. (2002). "Judo, better than dance, develops sensorimotor adaptabilities involved in balance control." Gait & posture **15**(2): 187-194.

Pichierri, G., et al. (2012). "A cognitive-motor intervention using a dance video game to enhance foot placement accuracy and gait under dual task conditions in older adults: a randomized controlled trial." BMC geriatrics **12**(1): 74.

Pigeon, P., et al. (1997). "Intensive dance practice: repercussions on growth and puberty." The American journal of sports medicine **25**(2): 243-247.

Rani, S. and S. P. Sing (2013). "Effect of selected yogic practices and aerobic dance on health related physical fitness variables among Nicobari women students." Int J Phys Educ Fitness Sports **2**(4): 76-79.

Rehfeld, K., et al. (2017). "Dancing or fitness sport? The effects of two training programs on hippocampal plasticity and balance abilities in healthy seniors." Frontiers in human neuroscience **11**: 305.

Rein, S., et al. (2011). "Postural control and functional ankle stability in professional and amateur dancers." Clinical Neurophysiology **122**(8): 1602-1610.

Romenets, S. R., et al. (2015). "Tango for treatment of motor and non-motor manifestations in Parkinson's disease: a randomized control study." Complementary Therapies in Medicine **23**(2): 175-184.

Rossmeissl, A., et al. (2016). "ZumBeat: Evaluation of a Zumba dance intervention in postmenopausal overweight women." Sports **4**(1): 5.

Schmit, J. M., et al. (2005). "Dynamic patterns of postural sway in ballet dancers and track athletes." Experimental Brain Research **163**(3): 370-378.

Schmitt, H., et al. (2005). "Influence of professional dance training on peak torque and proprioception at the ankle." Clinical Journal of Sport Medicine **15**(5): 331-339.

Serrano-Guzmán, M., et al. (2016). "Effectiveness of a flamenco and sevillanas program to enhance mobility, balance, physical activity, blood pressure, body mass, and quality of life in postmenopausal women living in the community in Spain: a randomized clinical trial." Menopause **23**(9): 965-973.

Shimamoto, H., et al. (1998). "Low impact aerobic dance as a useful exercise mode for reducing body mass in mildly obese middle-aged women." Applied Human Science **17**(3): 109-114.

Simmons, R. W. (2005). "Neuromuscular responses of trained ballet dancers to postural perturbations." International Journal of Neuroscience **115**(8): 1193-1203.

Simmons, R. W. (2005). "Sensory organization determinants of postural stability in trained ballet dancers." International Journal of Neuroscience **115**(1): 87-97.

Sofianidis, G., et al. (2017). "A comparative study of the effects of pilates and Latin dance on static and dynamic balance in older adults." Journal of aging and physical activity **25**(3): 412-419.

Sofianidis, G., et al. (2009). "Effect of a 10-week traditional dance program on static and dynamic balance control in elderly adults." Journal of aging and physical activity **17**(2): 167-180.

Stalder, M. A., et al. (1990). "The effects of supplemental weight training for ballet dancers." The Journal of Strength & Conditioning Research **4**(3): 95-102.

Štalec, J., et al. (2007). "The impact of dance-aerobics training on the morpho-motor status in female high-schoolers." Collegium antropologicum **31**(1): 259-266.

Steinberg, N., et al. (2008). "Growth and development of female dancers aged 8–16 years." American Journal of Human Biology: The Official Journal of the Human Biology Association **20**(3): 299-307.

Thiesen, T. and A. Sumiya (2011). "Equilíbrio e arco plantar no balé clássico." Conscientiae Saúde **10**(1): 138-142.

Thullier, F. and H. Moufti (2004). "Multi-joint coordination in ballet dancers." Neuroscience letters **369**(1): 80-84.

Tsanaka, A., et al. (2017). "Effects of a modified ballet class on strength and jumping ability in college ballet dancers." Journal of dance medicine & science **21**(3): 97-101.

Verghese, J. (2006). "Cognitive and mobility profile of older social dancers." Journal of the American Geriatrics Society **54**(8): 1241-1244.

Volpe, D., et al. (2013). "A comparison of Irish set dancing and exercises for people with Parkinson's disease: a phase II feasibility study." BMC geriatrics **13**(1): 1-6.

Wallmann, H. W., et al. (2009). "The effect of a senior jazz dance class on static balance in healthy women over 50 years of age: a pilot study." Biological research for nursing **10**(3): 257-266.

White, M. K., et al. (1984). "The effects of exercise on the bones of postmenopausal women." International orthopaedics **7**(4): 209-214.

Wu, H. Y., et al. (2016). "Effects of low-impact dance on blood biochemistry, bone mineral density, the joint range of motion of lower extremities, knee extension torque, and fall in females." Journal of aging and physical activity **24**(1): 1-7.

Wu, H.-Y., et al. (2011). "The effects of low-impact dance on knee torque and lower extremity mobility in middle-aged and older women." Journal of Nursing Research **19**(4): 267-274.

Zhang, J.-G., et al. (2008). "Postural stability and physical performance in social dancers." Gait & posture **27**(4): 697-701.

Zhang, X., et al. (2012). "Study about effects of square dancing on the cognitive function and emotion of the aged." Chin Foreign Med Res **10**: 4-5.

Included Individual Studies *(from included reviews)* **– Music**

Anaya, E. M., et al. (2016). "Long-term musical experience and auditory and visual perceptual abilities under adverse conditions." The Journal of the Acoustical Society of America **140**(3): 2074-2081.

Annello, J. A. (1972). "A Comparison of Academic Achievement between Instrumental Music Students and Non-Music Students in the El Dorado and Valencia High Schools of the Placentia United School District." Doctoral Diss., Brigham Young University.

Bae, M. (2011). "Effect of group music therapy on student music therapists' anxiety, mood, job engagement, and self-efficacy." Univ Kansas **132**: 3458209.

Başkent, D. and E. Gaudrain (2016). "Musician advantage for speech-on-speech perception." The Journal of the Acoustical Society of America **139**(3): EL51-EL56.

Beck, R., et al. (2000). "Choral singing, performance perception, and immune system changes in salivary immunoglobulin A and cortisol." Music Perception **18**(1): 87-106.

Beck, R. J., et al. (2006). "Supporting the Health of College Solo Singers: The Relationship of Positive Emotions and Stress to Changes in Salivary IgA and Cortisol during Singing." Journal for Learning through the Arts **2**(1): 19.

Bengtsson, S. L., et al. (2007). "Cortical regions involved in the generation of musical structures during improvisation in pianists." Journal of cognitive neuroscience **19**(5): 830-842.

Berkowitz, A. L. and D. Ansari (2008). "Generation of novel motor sequences: the neural correlates of musical improvisation." Neuroimage **41**(2): 535-543.

Bialystok, E. and A.-M. DePape (2009). "Musical expertise, bilingualism, and executive functioning." Journal of Experimental Psychology: Human Perception and Performance **35**(2): 565.

Bidelman, G. M., et al. (2014). "Coordinated plasticity in brainstem and auditory cortex contributes to enhanced categorical speech perception in musicians." European Journal of Neuroscience **40**(4): 2662-2673.

Bilhartz, T. D., et al. (1999). "The effect of early music training on child cognitive development." Journal of Applied Developmental Psychology **20**(4): 615-636.

Bittman, B., et al. (2003). "Recreational music-making: a cost-effective group interdisciplinary strategy for reducing burnout and improving mood states in long-term care workers." Advances in Mind Body Medicine **19**(3/4): 4-15.

Bittman, B., et al. (2009). "Creative musical expression as a catalyst for quality-of-life improvement in inner-city adolescents placed in a court-referred residential treatment program." Advances in mind-body medicine **24**(1): 8-19.

Bittman, B. B., et al. (2001). "Composite effects of group drumming music therapy on modulation of neuroendocrine-immune parameters in normal subjects." Alternative therapies in health and medicine **7**(1): 38.

Bittman, B. B., et al. (2004). "Recreational music-making: an integrative group intervention for reducing burnout and improving mood states in first year associate degree nursing students: insights and economic impact." International Journal of Nursing Education Scholarship **1**(1).

Boebinger, D., et al. (2015). "Musicians and non-musicians are equally adept at perceiving masked speech." The Journal of the Acoustical Society of America **137**(1): 378-387.

Bolduc, J. and P. Lefebvre (2012). "Using nursery rhymes to foster phonological and musical processing skills in kindergarteners."

Bormann, J. E., et al. (2006). "Relationship of frequent mantram repetition to emotional and spiritual well-being in healthcare workers." The Journal of Continuing Education in Nursing **37**(5): 218-224.

Brandler, S. and T. H. Rammsayer (2003). "Differences in mental abilities between musicians and non-musicians." Psychology of music **31**(2): 123-138.

Brown, S., et al. (2006). "Music and language side by side in the brain: a PET study of the generation of melodies and sentences." European Journal of Neuroscience **23**(10): 2791-2803.

Callan, D. E., et al. (2006). "Song and speech: brain regions involved with perception and covert production." Neuroimage **31**(3): 1327-1342.

Catterall, J. S., et al. (1999). "Involvement in the arts and human development: General involvement and intensive involvement in music and theater arts." Champions of change: The impact of the arts on learning **1**: 1-18.

Chan, A. S., et al. (1998). "Music training improves verbal memory." Nature **396**(6707): 128-128.

Ciepluch, G. M. (1989). "Sightreading achievement in instrumental music performance, learning gifts, and academic achievement: A correlation study."

Clayton, K. K., et al. (2016). "Executive function, visual attention and the cocktail party problem in musicians and non-musicians." PloS one **11**(7): e0157638.

Coffey, E. B., et al. (2017). "Neural correlates of early sound encoding and their relationship to speech-in-noise perception." Frontiers in neuroscience **11**: 479.

Coffey, E. B., et al. (2016). "Cortical contributions to the auditory frequency-following response revealed by MEG." Nature communications **7**(1): 1-11.

Cogo-Moreira, H., et al. (2013). "Effectiveness of music education for the improvement of reading skills and academic achievement in young poor readers: a pragmatic cluster-randomized, controlled clinical trial." PloS one **8**(3): e59984.

Cohen, G. D., et al. (2006). "The impact of professionally conducted cultural programs on the physical health, mental health, and social functioning of older adults." The Gerontologist **46**(6): 726-734.

Cohen, M. L. (2009). "Choral singing and prison inmates: Influences of performing in a prison choir." Journal of Correctional Education (1974-) **60**(1): 52-65.

College Board (1988-1999). College bound seniors profile of SAT and achievement test takers.

Costa-Giomi, E. (1999). "The effects of three years of piano instruction on children's cognitive development." Journal of research in music education **47**(3): 198-212.

Costa-Giomi, E. (2004). "Effects of three years of piano instruction on children’s academic achievement, school performance and self-esteem." Psychology of music **32**(2): 139-152.

Coulton, S., et al. (2015). "Effectiveness and cost-effectiveness of community singing on mental health-related quality of life of older people: randomised controlled trial." The British Journal of Psychiatry **207**(3): 250-255.

de Manzano, Ö. and F. Ullén (2012). "Goal-independent mechanisms for free response generation: Creative and pseudo-random performance share neural substrates." Neuroimage **59**(1): 772-780.

Degé, F. and G. Schwarzer (2011). "The effect of a music program on phonological awareness in preschoolers." Frontiers in psychology **2**: 124.

Douglas, S. and P. Willatts (1994). "The relationship between musical ability and literacy skills." Journal of Research in reading **17**(2): 99-107.

Du, Y. and R. J. Zatorre (2017). "Musical training sharpens and bonds ears and tongue to hear speech better." Proceedings of the National Academy of Sciences **114**(51): 13579-13584.

Dunbar, R. I., et al. (2012). "Performance of music elevates pain threshold and positive affect: implications for the evolutionary function of music." Evolutionary Psychology **10**(4): 147470491201000403.

Engdahl, P. M. (1994). "The effect of pull-out programs on the academic achievement of sixth grade students in South Bend, Indiana."

Fetzer, L. (1996). "Facilitating print awareness and literacy development with familiar children's songs."

Flohr, J. W. (1996). "Children's Electrophysiological Responses to Music."

Flohr, J. W., et al. (1998). Quantitative EEG responses to music stimuli. Music Educators National Conference, Phoenix, Ariz.

Flohr, J. W., et al. (2000). "Brain Research Applied to Music Education." Music Educators National Conference.

Franklin, M. S., et al. (2008). "The effects of musical training on verbal memory." Psychology of music **36**(3): 353-365.

Friedman, B. (1959). An evaluation of the achievement in reading and arithmetic of pupils in elementary school instrumental music classes, New York University, School of Education.

Fuller, C. D., et al. (2014). "The musician effect: does it persist under degraded pitch conditions of cochlear implant simulations?" Frontiers in neuroscience **8**: 179.

George, E. M. and D. Coch (2011). "Music training and working memory: An ERP study." Neuropsychologia **49**(5): 1083-1094.

Gold, C., et al. (2014). "Music therapy for prisoners: Pilot randomised controlled trial and implications for evaluating psychosocial interventions." International journal of offender therapy and comparative criminology **58**(12): 1520-1539.

Grape, C., et al. (2002). "Does singing promote well-being?: An empirical study of professional and amateur singers during a singing lesson." Integrative Physiological & Behavioral Science **38**(1): 65-74.

Graziano, A. B., et al. (1999). "Enhanced learning of proportional math through music training and spatial-temporal training." Neurological Research **21**(2): 139-152.

Groff, F. H. (1963). Effect on academic achievement of excusing elementary school pupils from classes to study instrumental music, University of Connecticut.

Gromko, J. E. (2005). "The effect of music instruction on phonemic awareness in beginning readers." Journal of research in music education **53**(3): 199-209.

Gromko, J. E. and A. S. Poorman (1998). "The effect of music training on preschoolers' spatial-temporal task performance." Journal of research in music education **46**(2): 173-181.

Hansen, M., et al. (2013). "Working memory and musical competence of musicians and non-musicians." Psychology of music **41**(6): 779-793.

Helmbold, N., et al. (2005). "Differences in primary mental abilities between musicians and nonmusicians." Journal of Individual Differences **26**(2): 74-85.

Herrera, L., et al. (2011). "Effects of phonological and musical training on the reading readiness of native- and foreign-Spanish-speaking children." Psychology of music **39**(1): 68-81.

Hilliard, R. E. (2006). "The effect of music therapy sessions on compassion fatigue and team building of professional hospice caregivers." The Arts in Psychotherapy **33**(5): 395-401.

Ho, P., et al. (2011). "The impact of group drumming on social-emotional behavior in low-income children." Evidence-Based Complementary and Alternative Medicine **2011**.

Ho, Y.-C., et al. (2003). "Music training improves verbal but not visual memory: cross-sectional and longitudinal explorations in children." Neuropsychology **17**(3): 439.

Houston, D. M., et al. (1998). "Using humour to promote psychological wellbeing in residential homes for older people." Aging & Mental Health **2**(4): 328-332.

Huang, Z., et al. (2010). "Verbal memory retrieval engages visual cortex in musicians." Neuroscience **168**(1): 179-189.

Hurwitz, I., et al. (1975). "Nonmusicol effects of the kodaly music curriculum in primary grade children." Journal of learning Disabilities **8**(3): 167-174.

Jakobson, L. S., et al. (2008). "Memory for verbal and visual material in highly trained musicians." Music Perception **26**(1): 41-55.

Jeffries, K., et al. (2003). "Words in melody: an H215O PET study of brain activation during singing and speaking." Neuroreport **14**(5): 749-754.

Jentschke, S., et al. (2005). "Investigating the relationship of music and language in children: influences of musical training and language impairment." Annals of the New York Academy of Sciences **1060**(1): 231-242.

Kelley, L. L. (1981). "A combined experimental and descriptive study of the effect of music on reading and language."

Kennedy, R. (1998). The effects of musical performance, rational emotive therapy and vicarious experience on the self-efficacy and self-esteem of juvenile delinquents and disadvantaged children, University of Kansas, Music and Dance.

Kleber, B., et al. (2007). "Overt and imagined singing of an Italian aria." Neuroimage **36**(3): 889-900.

Kleber, B., et al. (2010). "The brain of opera singers: experience-dependent changes in functional activation." Cerebral Cortex **20**(5): 1144-1152.

Koelsch, S., et al. (2010). "Music in the treatment of affective disorders: an exploratory investigation of a new method for music-therapeutic research." Music Perception **27**(4): 307-316.

Koyama, M., et al. (2009). "Recreational music-making modulates immunological responses and mood states in older adults." Journal of medical and dental sciences **56**(2): 79-90.

Kreutz, G. (2014). "Does singing facilitate social bonding." Music Med **6**(2): 51-60.

Kreutz, G., et al. (2004). "Effects of choir singing or listening on secretory immunoglobulin A, cortisol, and emotional state." Journal of behavioral medicine **27**(6): 623-635.

Kuhn, D. (2002). "The effects of active and passive participation in musical activity on the immune system as measured by salivary immunoglobulin A (SlgA)." Journal of music therapy **39**(1): 30-39.

Kvet, E. J. (1985). "Excusing elementary school students from regular classroom activities for the study of instrumental music: The effect on sixth-grade reading, language, and mathematics achievement." Journal of research in music education **33**(1): 45-54.

Laczo, Z. (1985). "The nonmusical outcomes of music education: Influence on intelligence?" Bulletin of the Council for Research in Music Education: 109-118.

Lamar, H. B. (1989). An examination of the congruency of music aptitude scores and mathematics and reading achievement scores of elementary children, University of Southern Mississippi.

Lee, K. M., et al. (2009). "Selective subcortical enhancement of musical intervals in musicians." Journal of Neuroscience **29**(18): 5832-5840.

Lee, Y.-s., et al. (2007). "Effects of skill training on working memory capacity." Learning and Instruction **17**(3): 336-344.

Limb, C. J. and A. R. Braun (2008). "Neural substrates of spontaneous musical performance: An fMRI study of jazz improvisation." PloS one **3**(2): e1679.

Lindblad, F., et al. (2007). "Music intervention for 5th and 6th graders—effects on development and cortisol secretion." Stress and Health: Journal of the International Society for the Investigation of Stress **23**(1): 9-14.

Mallory, M. E. and K. E. Philbrick (1995). "Music training and spatial skills in preschool children." America Psychological Association of Music, New York.

McCarthy, K. J. (1992). "Music Performance Group Membership and Academic Success: A Descriptive Study of One 4-year High School." Colorado Music Educators Association.

Monahan, C. B., et al. (1987). "The effect of melodic and temporal contour on recognition memory for pitch change." Perception & Psychophysics **41**(6): 576-600.

Moreno, S., et al. (2011). "Effect of music training on promoting preliteracy skills: Preliminary causal evidence." Music Perception **29**(2): 165-172.

Moreno, S., et al. (2009). "Musical training influences linguistic abilities in 8-year-old children: more evidence for brain plasticity." Cerebral Cortex **19**(3): 712-723.

Moritz, C., et al. (2013). "Links between early rhythm skills, musical training, and phonological awareness." Reading and Writing **26**(5): 739-769.

Mungas, R. and M. J. Silverman (2014). "Immediate effects of group-based wellness drumming on affective states in university students." The Arts in Psychotherapy **41**(3): 287-292.

Musacchia, G., et al. (2007). "Musicians have enhanced subcortical auditory and audiovisual processing of speech and music." Proceedings of the National Academy of Sciences **104**(40): 15894-15898.

Musacchia, G., et al. (2008). "Relationships between behavior, brainstem and cortical encoding of seen and heard speech in musicians and non-musicians." Hearing research **241**(1-2): 34-42.

Neufeld, K. A. (1986). "Understanding of selected pre-number concepts: Relationships to a formal music program." Alberta Journal of Educational Research.

Okhrei, A., et al. (2016). "Performance of working memory of musicians and non-musicians in tests with letters, digits, and geometrical shapes." Biologija **62**(4).

Olanoff, M. and L. Kirschner (1969). Musical Ability Utilization Program. Final Report, ERIC Clearinghouse.

Oxenham, A. J., et al. (2003). "Informational masking and musical training." The Journal of the Acoustical Society of America **114**(3): 1543-1549.

Pai, I., et al. (2008). "The effect of singing on snoring and daytime somnolence." Sleep and Breathing **12**(3): 265-268.

Pallesen, K. J., et al. (2010). "Cognitive control in auditory working memory is enhanced in musicians." PloS one **5**(6): e11120.

Parbery-Clark, A., et al. (2012). "Musical experience offsets age-related delays in neural timing." Neurobiology of aging **33**(7): 1483. e1481-1483. e1484.

Parbery-Clark, A., et al. (2012). "Musical experience strengthens the neural representation of sounds important for communication in middle-aged adults." Frontiers in aging neuroscience **4**: 30.

Parbery-Clark, A., et al. (2009). "Musician enhancement for speech-in-noise." Ear and hearing **30**(6): 653-661.

Parbery-Clark, A., et al. (2011). "Musical experience and the aging auditory system: implications for cognitive abilities and hearing speech in noise." PloS one **6**(5): e18082.

Parbery-Clark, A., et al. (2011). "Context-dependent encoding in the auditory brainstem subserves enhanced speech-in-noise perception in musicians." Neuropsychologia **49**(12): 3338-3345.

Parente, J. A. and J. J. O'Malley (1975). "Training in musical rhythm and field dependence of children." Perceptual and motor skills **40**(2): 392-394.

Perkins, R. and A. Williamon (2014). "Learning to make music in older adulthood: A mixed-methods exploration of impacts on wellbeing." Psychology of music **42**(4): 550-567.

Perry, D. W., et al. (1999). "Localization of cerebral activity during simple singing." Neuroreport **10**(16): 3453-3458.

Piro, J. M. and C. Ortiz (2009). "The effect of piano lessons on the vocabulary and verbal sequencing skills of primary grade students." Psychology of music **37**(3): 325-347.

Portowitz, A., et al. (2009). "Underlying mechanisms linking music education and cognitive modifiability." Research Studies in Music Education **31**(2): 107-128.

Ramachandra, V., et al. (2012). "The Impact of Musical Training on the Phonological Memory and the Central Executive: A Brief Report." North American Journal of Psychology **14**(3).

Rauscher, F., et al. (1997). "Music training causes long-term enhancement of preschool children's spatial–temporal reasoning." Neurological Research **19**(1): 2-8.

Rauscher, F. H. (1994). "Music and Spatial Task Performance: A Causal Relationship."

Rauscher, F. H. (1999). Music, cognitive development, and the classroom: Franklin Elementary. American Orff-Schulwerk Association National Conference, Phoenix, Ariz.

Rauscher, F. H. and M. A. Zupan (2000). "Classroom keyboard instruction improves kindergarten children's spatial-temporal performance: A field experiment." Early childhood research quarterly **15**(2): 215-228.

Register, D. (2001). "The effects of an early intervention music curriculum on prereading/writing." Journal of music therapy **38**(3): 239-248.

Register, D. (2004). "The effects of live music groups versus an educational children's television program on the emergent literacy of young children." Journal of music therapy **41**(1): 2-27.

Rickard, N. S., et al. (2012). "Absence of widespread psychosocial and cognitive effects of school-based music instruction in 10–13-year-old students." International Journal of Music Education **30**(1): 57-78.

Roberts, D. L. (1978). An experimental study of the relationship between musical note-reading and language reading, University of Missouri-Kansas City, Conservatory of Music.

Rodrigues, A. C., et al. (2014). "Visual memory in musicians and non-musicians." Frontiers in human neuroscience **8**: 424.

Roskam, K. (1979). "Music therapy as an aid for increasing auditory awareness and improving reading skill." Journal of music therapy **16**(1): 31-42.

Ruggles, D. R., et al. (2014). "Influence of musical training on understanding voiced and whispered speech in noise." PloS one **9**(1): e86980.

Ruiz, M. H., et al. (2009). "Detecting wrong notes in advance: neuronal correlates of error monitoring in pianists." Cerebral Cortex **19**(11): 2625-2639.

Sandgren, M. (2009). Evidence for strong immediate well-being effects of choral singing–With more enjoyment for women than for men. ESCOM 2009: 7th Triennial Conference of European Society for the Cognitive Sciences of Music.

Schellenberg, E. G. (2004). "Music lessons enhance IQ." Psychological science **15**(8): 511-514.

Schiavio, A. and R. Timmers (2016). "Motor and audiovisual learning consolidate auditory memory of tonally ambiguous melodies." Music Perception: An Interdisciplinary Journal **34**(1): 21-32.

Schorr-Lesnick, B., et al. (1985). "Pulmonary function in singers and wind-instrument players." Chest **88**(2): 201-205.

Schulze, K., et al. (2011). "Working memory for tonal and atonal sequences during a forward and a backward recognition task." Music Perception **29**(3): 255-267.

Schulze, K., et al. (2011). "Neural correlates of strategy use during auditory working memory in musicians and non-musicians." European Journal of Neuroscience **33**(1): 189-196.

Schulze, K., et al. (2011). "Neuroarchitecture of verbal and tonal working memory in nonmusicians and musicians." Human brain mapping **32**(5): 771-783.

Slater, J. and N. Kraus (2016). "The role of rhythm in perceiving speech in noise: a comparison of percussionists, vocalists and non-musicians." Cognitive processing **17**(1): 79-87.

Slater, J., et al. (2015). "Music training improves speech-in-noise perception: Longitudinal evidence from a community-based music program." Behavioural brain research **291**: 244-252.

Smith, C., et al. (2014). "African drumming: a holistic approach to reducing stress and improving health?" Journal of Cardiovascular Medicine **15**(6): 441-446.

Steurer, M., et al. (1998). "Does choir singing cause noise-induced hearing loss?" Audiology **37**(1): 38-51.

Strait, D. L. and N. Kraus (2011). "Can you hear me now? Musical training shapes functional brain networks for selective auditory attention and hearing speech in noise." Frontiers in psychology **2**: 113.

Strait, D. L., et al. (2010). "Musical experience shapes top-down auditory mechanisms: evidence from masking and auditory attention performance." Hearing research **261**(1-2): 22-29.

Suárez, L., et al. (2016). "Cross-sectional study on the relationship between music training and working memory in adults." Australian Journal of Psychology **68**(1): 38-46.

Swaminathan, J., et al. (2015). "Musical training, individual differences and the cocktail party problem." Scientific reports **5**: 11628.

Taetle, L. D. (1999). "The effect of active and passive music instruction on the spatial ability of kindergarten children."

Talamini, F., et al. (2016). "The working memory of musicians and nonmusicians." Music Perception: An Interdisciplinary Journal **34**(2): 183-191.

Taylor, A. C. and S. A. Dewhurst (2017). "Investigating the influence of music training on verbal memory." Psychology of music **45**(6): 814-820.

Thomson, J. M., et al. (2013). "Auditory processing interventions and developmental dyslexia: a comparison of phonemic and rhythmic approaches." Reading and Writing **26**(2): 139-161.

Tierney, A. T., et al. (2015). "Music training alters the course of adolescent auditory development." Proceedings of the National Academy of Sciences **112**(32): 10062-10067.

Tsang, C. D. and N. J. Conrad (2011). "Music training and reading readiness." Music Perception **29**(2): 157-163.

Unwin, M. M., et al. (2002). "The effects of group singing on mood." Psychology of music **30**(2): 175-185.

Valentine, E. and C. Evans (2001). "The effects of solo singing, choral singing and swimming on mood and physiological indices." British Journal of Medical Psychology **74**(1): 115-120.

Varnet, L., et al. (2015). "How musical expertise shapes speech perception: evidence from auditory classification images." Scientific reports **5**: 14489.

Vasuki, P. R. M., et al. (2016). "Musicians' edge: A comparison of auditory processing, cognitive abilities and statistical learning." Hearing research **342**: 112-123.

Vogt, S., et al. (2007). "Prefrontal involvement in imitation learning of hand actions: effects of practice and expertise." Neuroimage **37**(4): 1371-1383.

Wachi, M., et al. (2007). "Recreational music-making modulates natural killer cell activity, cytokines, and mood states in corporate employees." Medical Science Monitor **13**(2): CR57-CR70.

Weeden, R. E. (1972). A comparison of the academic achievement in reading and mathematics of Negro children whose parents are interested, not interested, or involved in a program of Suzuki violin, ProQuest Information & Learning.

Weiss, A. H., et al. (2014). "Spatial vision is superior in musicians when memory plays a role." Journal of vision **14**(9): 18-18.

Wheeler, L. R. and V. D. Wheeler (1951). "The intelligence of music students." Journal of Educational psychology **42**(4): 223.

Wise, G. W., et al. (1992). "Exploration of the relationship between choral singing and successful aging." Psychological Reports **70**(3_suppl): 1175-1183.

Yazejian, N. and E. S. Peisner-Feinberg (2009). "Effects of a preschool music and movement curriculum on children's language skills." NHSA dialog **12**(4): 327-341.

Zarate, J. M., et al. (2010). "Neural networks involved in voluntary and involuntary vocal pitch regulation in experienced singers." Neuropsychologia **48**(2): 607-618.

Zarate, J. M. and R. J. Zatorre (2008). "Experience-dependent neural substrates involved in vocal pitch regulation during singing." Neuroimage **40**(4): 1871-1887.

Zendel, B. R. and C. Alain (2012). "Musicians experience less age-related decline in central auditory processing." Psychology and aging **27**(2): 410.

Zendel, B. R., et al. (2015). "The impact of musicianship on the cortical mechanisms related to separating speech from background noise." Journal of cognitive neuroscience **27**(5): 1044-1059.

Zuk, J., et al. (2014). "Behavioral and neural correlates of executive functioning in musicians and non-musicians." PloS one **9**(6): e99868.

Zulauf, M. (1993). "Three-year experiment in extended music teaching in Switzerland: The different effects observed in a group of French-speaking pupils." Bulletin of the Council for Research in Music Education: 111-121.

<u>Excluded Individual Studies *(from included reviews)* – Dance</u>

Ambegaonkar, J. P., et al. (2016). "Lower extremity hypermobility, but not core muscle endurance influences balance in female collegiate dancers." International journal of sports physical therapy **11**(2): 220.

Annino, G., et al. (2007). "Effect of whole body vibration training on lower limb performance in selected high-level ballet students." The Journal of Strength & Conditioning Research **21**(4): 1072-1076.

Arzoglou, D., et al. (2013). "The effect of [alpha] tradinional dance training program on neuromuscular coordination of individuals with autism." Journal of Physical Education and Sport **13**(4): 563.

Barcellos, C. and I. LA (2002). "Alterações posturais e do equilíbrio corporal na primeira posição em ponta do balé clássico." Rev Paul Educ Fís **16**(1): 43-52.

Batson, G. (2010). "Validating a dance-specific screening test for balance: preliminary results from multisite testing." Medical Problems of Performing Artists **25**(3): 110-115.

Belardinelli, R., et al. (2008). "Waltz dancing in patients with chronic heart failure: new form of exercise training." Circulation: Heart Failure **1**(2): 107-114.

Bruyneel, A., et al. (2010). "Organization of postural equilibrium in several planes in ballet dancers." Neuroscience letters **485**(3): 228-232.

Candelario-Gorbea, C., et al. (2014). "Effectiveness of a PNF intervention on active and passive range of motion during "developpe a la second" in ballet dancers." Orthopaedic Physical Therapy Practice **26**(3): 198-202.

Caplan, N. and A. S. C. Gibson (2011). "Single leg postural sway characteristics of dancers during a rotating task: a pilot study." Arts Biomechanics **1**(1): 45.

Cheng, H. S., et al. (2011). "Preliminary results of dancing exercise on postural stability in adolescent females." The Kaohsiung journal of medical sciences **27**(12): 566-572.

Clark, T. and E. Redding (2012). "The relationship between postural stability and dancer's past and future lower-limb injuries." Medical Problems of Performing Artists **27**(4): 197-204.

Cloak, R., et al. (2010). "Vibration training improves balance in unstable ankles." International journal of sports medicine **31**(12): 894.

Coutts, R. A., et al. (2006). "Development and assessment of an incremental fatigue protocol for contemporary dance." Medical Problems of Performing Artists **21**(2): 65-70.

da Costa, P. H. L., et al. (2013). "Single leg balancing in ballet: Effects of shoe conditions and poses." Gait & posture **37**(3): 419-423.

Da Silva, C. C., et al. (2015). "The effects of 17 weeks of ballet training on the autonomic modulation, hormonal and general biochemical profile of female adolescents." Journal of human kinetics **47**(1): 61-71.

Denardi, R. A., et al. (2008). "Informação visual e controlo postural durante a execução da pirouette no ballet." Revista Portuguesa de Ciências do Desporto **8**(2): 241-250.

Doi, T., et al. (2017). "Effects of cognitive leisure activity on cognition in mild cognitive impairment: results of a randomized controlled trial." Journal of the American Medical Directors Association **18**(8): 686-691.

Duncan, R. P. and G. M. Earhart (2014). "Are the effects of community-based dance on Parkinson disease severity, balance, and functional mobility reduced with time? A 2-year prospective pilot study." The Journal of Alternative and Complementary Medicine **20**(10): 757-763.

Engels, H.-J., et al. (1998). "Effects of low-impact, moderate-intensity exercise training with and without wrist weights on functional capacities and mood states in older adults." Gerontology **44**(4): 239-244.

Golomer, E., et al. (1997). "Sex-linked differences in equilibrium reactions among adolescents performing complex sensorimotor tasks." Journal of Physiology-Paris **91**(2): 49-55.

Golomer, E., et al. (1999). "The contribution of vision in dynamic spontaneous sways of male classical dancers according to student or professional level." Journal of Physiology-Paris **93**(3): 233-237.

Golomer, E. M., et al. (2009). "Influence of vision and motor imagery styles on equilibrium control during whole-body rotations." Somatosensory & motor research **26**(4): 105-110.

Hackney, M. E. and G. M. Earhart (2009). "Effects of dance on movement control in Parkinson's disease: a comparison of Argentine tango and American ballroom." Journal of rehabilitation medicine **41**(6): 475-481.

Hackney, M. E. and G. M. Earhart (2009). "Health-related quality of life and alternative forms of exercise in Parkinson disease." Parkinsonism & related disorders **15**(9): 644-648.

Hackney, M. E. and G. M. Earhart (2009). "Short duration, intensive tango dancing for Parkinson disease: an uncontrolled pilot study." Complementary Therapies in Medicine **17**(4): 203-207.

Hackney, M. E., et al. (2013). "Dancing for balance: feasibility and efficacy in oldest-old adults with visual impairment." Nursing research **62**(2): 138-143.

Hackney, M. E., et al. (2007). "A study on the effects of Argentine tango as a form of partnered dance for those with Parkinson disease and the healthy elderly." American Journal of Dance Therapy **29**(2): 109-127.

Holmerová, I., et al. (2010). "Effect of the exercise dance for seniors (EXDASE) program on lower-body functioning among institutionalized older adults." Journal of aging and health **22**(1): 106-119.

Hopkins, D. R., et al. (1990). "Effect of low-impact aerobic dance on the functional fitness of elderly women." The Gerontologist **30**(2): 189-192.

Jeon, M. Y., et al. (2005). "The effects of a Korean traditional dance movement program in elderly women." Journal of Korean Academy of Nursing **35**(7): 1268-1276.

Jeon, M. Y., et al. (2000). "Effect of Korean traditional dance movement training on balance, gait and leg strength in home bound elderly women." Journal of Korean Academy of Nursing **30**(3): 647-658.

Kaltsatou, A. C., et al. (2014). "Functional and psychosocial effects of either a traditional dancing or a formal exercising training program in patients with chronic heart failure: a comparative randomized controlled study." Clinical Rehabilitation **28**(2): 128-138.

Kattenstroth, J.-C., et al. (2013). "Six months of dance intervention enhances postural, sensorimotor, and cognitive performance in elderly without affecting cardio-respiratory functions." Frontiers in aging neuroscience **5**: 5.

Khan, K. M., et al. (2000). "Can 16–18-year-old elite ballet dancers improve their hip and ankle range of motion over a 12-month period?" Clinical Journal of Sport Medicine **10**(2): 98-103.

Kim, C.-G., et al. (2003). "Effects of a health-promotion program on cardiovascular risk factors, health behaviors, and life satisfaction in institutionalized elderly women." International journal of nursing studies **40**(4): 375-381.

Kim, H.-S., et al. (2007). "Effects of nutrition education and exercise programs on perceived dietary behaviors, food intake and serum lipid profiles in elderly Korean women living in residential homes." Asian Nursing Research **1**(1): 35-47.

Kim, S.-H., et al. (2011). "Effect of dance exercise on cognitive function in elderly patients with metabolic syndrome: a pilot study." Journal of sports science & Medicine **10**(4): 671.

Kirkendall, D., et al. (1984). "Isokinetic characteristics of ballet dancers and the response to a season of ballet training." Journal of Orthopaedic & Sports Physical Therapy **5**(4): 207-211.

Koutedakis, Y., et al. (1996). "The effects of strength training in male ballet dancers." Impulse **4**(3): 210-219.

Koutedakis, Y. and N. C. Sharp (2004). "Thigh-muscles strength training, dance exercise, dynamometry, and anthropometry in professional ballerinas." Journal of strength and conditioning research **18**(4): 714-718.

Krampe, J. (2013). "Exploring the effects of dance-based therapy on balance and mobility in older adults." Western journal of nursing research **35**(1): 39-56.

Krampe, J., et al. (2014). "Does dance-based therapy increase gait speed in older adults with chronic lower extremity pain: A feasibility study." Geriatric Nursing **35**(5): 339-344.

Li, Z.-t., et al. (2014). "Biomechanical research on the balance control of pirouettes in classical dance." Journal of Beijing Sport University **1**.

Liederbach, M., et al. (1992). "Monitoring training status in professional ballet dancers." The Journal of sports medicine and physical fitness **32**(2): 187-195.

Lim, S.-T., et al. (2015). "Effects of a healthy life exercise program on arteriosclerosis adhesion molecules in elderly obese women." Journal of physical therapy science **27**(5): 1529-1532.

Lin, C.-F., et al. (2005). "Ankle biomechanics of ballet dancers in relevé en pointé dance." Research in Sports Medicine **13**(1): 23-35.

Lin, C.-W., et al. (2014). "Differences of ballet turns (pirouette) performance between experienced and novice ballet dancers." Research quarterly for exercise and sport **85**(3): 330-340.

López-Ortiz, C., et al. (2012). "Dance program for physical rehabilitation and participation in children with cerebral palsy." Arts & Health **4**(1): 39-54.

Marín, G. H., et al. (2009). "Evaluation of the state intervention project to improve quality of life and reduce the complications associated with aging:" Add health to your years"." Gaceta sanitaria **23**(4): 272-277.

McGill, A., et al. (2019). "Effects of a ballet-based dance intervention on gait variability and balance confidence of people with Parkinson's." Arts & Health **11**(2): 133-146.

McKee, K. E. and M. E. Hackney (2013). "The effects of adapted tango on spatial cognition and disease severity in Parkinson's disease." Journal of motor behavior **45**(6): 519-529.

McMillan, A., et al. (1998). "The effect of Pilates-based training on dancers' dynamic posture." Journal of dance medicine & science **2**(3): 101-107.

Mertz, L. and C. Docherty (2012). "Self-described differences between legs in ballet dancers: do they relate to postural stability and ground reaction force measures?" Journal of dance medicine & science **16**(4): 154-160.

Micheli, L. J., et al. (2005). "Preseason to postseason changes in body composition of professional ballet dancers." Journal of dance medicine & science **9**(2): 56-59.

Morrin, N. and E. Redding (2013). "Acute effects of warm-up stretch protocols on balance, vertical jump height, and range of motion in dancers." Journal of dance medicine & science **17**(1): 34-40.

Müller, P., et al. (2017). "Evolution of neuroplasticity in response to physical activity in old age: the case for dancing." Frontiers in aging neuroscience **9**: 56.

Murrock, C. J. and C. H. Graor (2014). "Effects of dance on depression, physical function, and disability in underserved adults." Journal of aging and physical activity **22**(3): 380-385.

Notarnicola, A., et al. (2014). "Effect of teaching with or without mirror on balance in young female ballet students." BMC research notes **7**(1): 426.

Pappas, E., et al. (2011). "Time to stability differences between male and female dancers after landing from a jump on flat and inclined floors." Clinical Journal of Sport Medicine **21**(4): 325-329.

Rehfeld, K., et al. (2018). "Dance training is superior to repetitive physical exercise in inducing brain plasticity in the elderly." PloS one **13**(7): e0196636.

Romenets, S. R., et al. (2015). "Tango for treatment of motor and non-motor manifestations in Parkinson's disease: a randomized control study." Complementary Therapies in Medicine **23**(2): 175-184.

Scheidler, A. M., et al. (2018). "Targeted ballet program mitigates ataxia and improves balance in females with mild-to-moderate multiple sclerosis." PloS one **13**(10): e0205382.

Shigematsu, R., et al. (2002). "Dance-based aerobic exercise may improve indices of falling risk in older women." Age and ageing **31**(4): 261-266.

Silvestri, L. (1986). "Effects of aerobic dance and progressive relaxation on improving physical fitness of high school girls." Perceptual and motor skills **63**(1): 131-135.

Song, R., et al. (2004). "Comparisons of motivation, health behaviors, and functional status among elders in residential homes in Korea." Public Health Nursing **21**(4): 361-371.

Uusi-Rasi, K., et al. (1999). "Long-term recreational gymnastics, estrogen use, and selected risk factors for osteoporotic fractures." Journal of Bone and Mineral Research **14**(7): 1231-1238.

Ventura, M. I., et al. (2016). "A pilot study to evaluate multi-dimensional effects of dance for people with Parkinson's disease." Contemporary clinical trials **51**: 50-55.

Volpe, D., et al. (2013). "A comparison of Irish set dancing and exercises for people with Parkinson's disease: a phase II feasibility study." BMC geriatrics **13**(1): 1-6.

Wyon, M. A., et al. (2013). "Effect of midsole thickness of dance shoes on dynamic postural stability." Medical Problems of Performing Artists **28**(4): 195-198.

Young, C., et al. (2007). "Simple, novel physical activity maintains proximal femur bone mineral density, and improves muscle strength and balance in sedentary, postmenopausal Caucasian women." Osteoporosis International **18**(10): 1379-1387.

Zaferiou, A. M., et al. (2016). "Whole-body balance regulation during the turn phase of Pique and Pirouette turns with varied rotational demands." Medical Problems of Performing Artists **31**(2): 96-103.

Excluded Individual Studies *(from included reviews)* – **Music**

Aloui, A., et al. (2015). "Listening to music during warming-up counteracts the negative effects of ramadan observance on short-term maximal performance." PloS one **10**(8): e0136400.

Anderson, K. and K. Overy (2010). "Engaging Scottish young offenders in education through music and art." International Journal of Community Music **3**(1): 47-64.

Anderson, L. W. and C. U. Gustavson (2016). "The impact of a knitting intervention on compassion fatigue in oncology nurses." Clin J Oncol Nurs **20**(1): 102-104.

Baker, S. and S. Homan (2007). "Rap, recidivism and the creative self: A popular music programme for young offenders in detention." Journal of Youth Studies **10**(4): 459-476.

Baldari, C., et al. (2010). "Effects of music during exercise in different training status." Journal of sports medicine and physical fitness **50**(3): 281.

Ball, T., et al. (2007). "Response properties of human amygdala subregions: evidence based on functional MRI combined with probabilistic anatomical maps." PloS one **2**(3): e307.

Baumgartner, T., et al. (2006). "The emotional power of music: how music enhances the feeling of affective pictures." Brain research **1075**(1): 151-164.

Beck, B. D., et al. (2015). "Coping with work-related stress through Guided Imagery and Music (GIM): Randomized controlled trial." Journal of music therapy **52**(3): 323-352.

Bekiroğlu, T., et al. (2013). "Effect of Turkish classical music on blood pressure: a randomized controlled trial in hypertensive elderly patients." Complementary Therapies in Medicine **21**(3): 147-154.

Bensimon, M., et al. (2015). "The impact of relaxing music on prisoners' levels of anxiety and anger." International journal of offender therapy and comparative criminology **59**(4): 406-423.

Bensimon, M. and A. Gilboa (2010). "The music of my life: The impact of the musical presentation on the sense of purpose in life and on self-consciousness." The Arts in Psychotherapy **37**(3): 172-178.

Berns, G. S., et al. (2010). "Neural mechanisms of the influence of popularity on adolescent ratings of music." Neuroimage **49**(3): 2687-2696.

Blood, A. J. and R. J. Zatorre (2001). "Intensely pleasurable responses to music correlate with activity in brain regions implicated in reward and emotion." Proceedings of the National Academy of Sciences **98**(20): 11818-11823.

Boothby, D. M. and S. J. Robbins (2011). "The effects of music listening and art production on negative mood: A randomized, controlled trial." The Arts in Psychotherapy **38**(3): 204-208.

Brooks, D. M., et al. (2010). "Creative approaches for reducing burnout in medical personnel." The Arts in Psychotherapy **37**(3): 255-263.

Brown, S. and M. J. Martinez (2007). "Activation of premotor vocal areas during musical discrimination." Brain and cognition **63**(1): 59-69.

Brown, S., et al. (2004). "Passive music listening spontaneously engages limbic and paralimbic systems." Neuroreport **15**(13): 2033-2037.

Bungay, H. and A. Skingley (2008). The Silver Song Club Project: Summary of a Formative Evaluation. Canterbury Christ Church University. Viitattu 12.1. 2011.

Burns, J. L., et al. (2002). "The effects of different types of music on perceived and physiological measures of stress." Journal of music therapy **39**(2): 101-116.

Bygren, L. O., et al. (2009). "Cultural participation and health: a randomized controlled trial among medical care staff." Psychosomatic Medicine **71**(4): 469-473.

Cai, G., et al. (2001). "Clinical observation of music therapy combined with anti-tumor drugs in treating 116 cases of tumor patients." Zhongguo Zhong xi yi jie he za zhi Zhongguo Zhongxiyi Jiehe Zazhi= Chinese Journal of Integrated Traditional and Western Medicine **21**(12): 891-894.

Campion, M. and L. Levita (2014). "Enhancing positive affect and divergent thinking abilities: Play some music and dance." The Journal of Positive Psychology **9**(2): 137-145.

Carissoli, C., et al. (2015). "Does a meditation protocol supported by a mobile application help people reduce stress? Suggestions from a controlled pragmatic trial." Cyberpsychology, Behavior, and Social Networking **18**(1): 46-53.

Chan, M. F., et al. (2010). "Effects of music on depression and sleep quality in elderly people: A randomised controlled trial." Complementary Therapies in Medicine **18**(3-4): 150-159.

Chan, M. F., et al. (2012). "Effects of music on depression in older people: a randomised controlled trial." Journal of Clinical Nursing **21**(5-6): 776-783.

Chang, F. Y., et al. (2010). "The effect of a music programme during lunchtime on the problem behaviour of the older residents with dementia at an institution in Taiwan." Journal of Clinical Nursing **19**(7-8): 939-948.

Chang, H.-C., et al. (2015). "The effects of music listening on psychosocial stress and maternal–fetal attachment during pregnancy." Complementary Therapies in Medicine **23**(4): 509-515.

Chang, M. Y., et al. (2008). "Effects of music therapy on psychological health of women during pregnancy." Journal of Clinical Nursing **17**(19): 2580-2587.

Chen, C. J., et al. (2015). "The effects of C hinese five-element music therapy on nursing students with depressed mood." International Journal of Nursing Practice **21**(2): 192-199.

Clift, S. and G. Hancox (2010). "The significance of choral singing for sustaining psychological wellbeing: findings from a survey of choristers in England, Australia and Germany." Music Performance Research **3**.

Clift, S., et al. (2007). Choral singing and psychological wellbeing: Findings from English choirs in a cross-national survey using the WHOQOL-BREF. Proceedings of the international symposium on performance science.

Clift, S., et al. (2009). What do Singers Say About the Effects of Choral Singing on Physical Health?-Findings from a Survey of Choristers in Australia, England and Germany. ESCOM 2009: 7th Triennial Conference of European Society for the Cognitive Sciences of Music.

Clift, S., et al. (2010). "Choral singing and psychological wellbeing: Quantitative and qualitative findings from English choirs in a cross-national survey." Journal of Applied Arts & Health **1**(1): 19-34.

Clift, S. M. and G. Hancox (2001). "The perceived benefits of singing: findings from preliminary surveys of a university college choral society." The journal of the Royal Society for the Promotion of Health **121**(4): 248-256.

Clift, S. M., et al. (2008). "Singing and health: A systematic mapping and review of non-clinical research."

Coen, S. J., et al. (2009). "Negative mood affects brain processing of visceral sensation." Gastroenterology **137**(1): 253-261. e252.

Cohen, G. D., et al. (2006). "The impact of professionally conducted cultural programs on the physical health, mental health, and social functioning of older adults." The Gerontologist **46**(6): 726-734.

Cohen, M. L. (2007). "Explorations of inmate and volunteer choral experiences in a prison-based choir." Australian Journal of Music Education(1): 61.

Cooke, M. L., et al. (2010). "A randomized controlled trial exploring the effect of music on agitated behaviours and anxiety in older people with dementia." Aging and mental health **14**(8): 905-916.

Courey, S. J., et al. (2012). "Academic music: music instruction to engage third-grade students in learning basic fraction concepts." Educational studies in mathematics **81**(2): 251-278.

De Roeper, J. and H. J. Savelsberg (2009). "Challenging the youth policy imperative: engaging young people through the arts." Journal of Youth Studies **12**(2): 209-225.

DeCarlo, A. and E. Hockman (2004). "RAP therapy: A group work intervention method for urban adolescents." Social work with Groups **26**(3): 45-59.

DuRousseau, D. R., et al. (2011). "Operational study to evaluate music-based neurotraining at improving sleep quality, mood, and daytime function in a first responder population." Journal of Neurotherapy **15**(4): 389-398.

Dyck, M., et al. (2011). "Cognitive versus automatic mechanisms of mood induction differentially activate left and right amygdala." Neuroimage **54**(3): 2503-2513.

Eldar, E., et al. (2007). "Feeling the real world: limbic response to music depends on related content." Cerebral Cortex **17**(12): 2828-2840.

Fechir, M., et al. (2008). "Patterns of sympathetic responses induced by different stress tasks." The open neurology journal **2**: 25.

Field, T., et al. (1997). "Job stress reduction therapies." Alternative therapies in health and medicine **3**(4): 54-56.

Flores-Gutiérrez, E. O., et al. (2007). "Metabolic and electric brain patterns during pleasant and unpleasant emotions induced by music masterpieces." International Journal of Psychophysiology **65**(1): 69-84.

Ford, J. H., et al. (2011). "Differential neural activity during search of specific and general autobiographical memories elicited by musical cues." Neuropsychologia **49**(9): 2514-2526.

Foss, A. H., et al. (2007). "Neural correlates of the Pythagorean ratio rules." Neuroreport **18**(15): 1521-1525.

Fujisawa, T. X. and N. D. Cook (2011). "The perception of harmonic triads: an fMRI study." Brain imaging and behavior **5**(2): 109-125.

Gann, E. (2010). "The effects of therapeutic hip-hop activity groups on perception of self and social supports in at-risk urban adolescents." Unpublished doctoral dissertation. The Wright Institute, Berkeley, CA.

Green, A. C., et al. (2008). "Music in minor activates limbic structures: a relationship with dissonance?" Neuroreport **19**(7): 711-715.

Guetin, S., et al. (2009). "Effect of music therapy on anxiety and depression in patients with Alzheimer's type dementia: randomised, controlled study." Dementia and geriatric cognitive disorders **28**(1): 36-46.

Gupta, U. and B. Gupta (2005). "Psychophysiological responsivity to Indian instrumental music." Psychology of music **33**(4): 363-372.

Harmat, L. and T. Theorell (2010). "Heart rate variability during singing and flute playing." Music and Medicine **2**(1): 10-17.

Haslam, C., et al. (2014). "Social identification moderates cognitive health and well-being following story-and song-based reminiscence." Aging & Mental Health **18**(4): 425-434.

Hays, T. (2005). "Well-being in later life through music." Australasian Journal on Ageing **24**(1): 28-32.

Hays, T. and V. Minichiello (2005). "The contribution of music to quality of life in older people: An Australian qualitative study." Ageing & Society **25**(2): 261-278.

Hicks-Moore, S. L. (2005). "Relaxing music at mealtime in nursing homes." Journal of gerontological nursing **31**(12): 26-32.

Hillman, S. (2002). "Participatory singing for older people: A perception of benefit." Health education.

Huang, R.-H. and Y.-N. Shih (2011). "Effects of background music on concentration of workers." Work **38**(4): 383-387.

Hugdahl, K., et al. (1999). "Brain activation during dichotic presentations of consonant-vowel and musical instrument stimuli: a 15O-PET study." Neuropsychologia **37**(4): 431-440.

Ifrach, E. R. and A. Miller (2016). "Social action art therapy as an intervention for compassion fatigue." The Arts in Psychotherapy **50**: 34-39.

Italia, S., et al. (2008). "Evaluation and art therapy treatment of the burnout syndrome in oncology units." Psycho-Oncology: Journal of the Psychological, Social and Behavioral Dimensions of Cancer **17**(7): 676-680.

James, C. E., et al. (2008). "Early neuronal responses in right limbic structures mediate harmony incongruity processing in musical experts." Neuroimage **42**(4): 1597-1608.

Janata, P. (2009). "The neural architecture of music-evoked autobiographical memories." Cerebral Cortex **19**(11): 2579-2594.

Jerde, T. A., et al. (2011). "Dissociable systems of working memory for rhythm and melody." Neuroimage **57**(4): 1572-1579.

Karpavičiūtė, S. and J. Macijauskienė (2016). "The impact of arts activity on nursing staff well-being: An intervention in the workplace." International Journal of Environmental Research and Public Health **13**(4): 435.

Kenny, D. T., et al. (2004). "Music performance anxiety and occupational stress amongst opera chorus artists and their relationship with state and trait anxiety and perfectionism." Journal of anxiety disorders **18**(6): 757-777.

Kim, D. S., et al. (2011). "Effects of music therapy on mood in stroke patients." Yonsei medical journal **52**(6): 977-981.

Knösche, T. R., et al. (2005). "Perception of phrase structure in music." Human brain mapping **24**(4): 259-273.

Koelsch, S., et al. (2008). "Amygdala activity can be modulated by unexpected chord functions during music listening." Neuroreport **19**(18): 1815-1819.

Koelsch, S., et al. (2006). "Investigating emotion with music: an fMRI study." Human brain mapping **27**(3): 239-250.

Kravits, K., et al. (2010). "Self-care strategies for nurses: A psycho-educational intervention for stress reduction and the prevention of burnout." Applied Nursing Research **23**(3): 130-138.

Lally, E. (2009). "The power to heal us with a smile and a song: Senior well-being, music-based participatory arts and the value of qualitative evidence." Journal of Arts & Communities **1**(1): 25-44.

Lane, D. (1994). "Effects of music therapy on immune function of hospitalized patients." Qual. Life–A Nurs Challenge **3**: 74-80.

Lashua, B. (2005). Making music, remaking leisure in the Beat of Boyle St, PhD thesis. University of Alberta, Edmonton.

Latimer, M. E. (2008). "Our voices enlighten, inspire, heal and empower. A mixed methods investigation of demography, sociology, and identity acquisition in a gay men's chorus." International Journal of Research in Choral Singing **3**(1): 23-38.

Lee, Y.-S., et al. (2011). "Investigation of melodic contour processing in the brain using multivariate pattern-based fMRI." Neuroimage **57**(1): 293-300.

Lee, Y. Y., et al. (2010). "Effectiveness of music intervention on the quality of life of older people." Journal of advanced nursing **66**(12): 2677-2687.

Lerner, Y., et al. (2009). "Eyes wide shut: amygdala mediates eyes-closed effect on emotional experience with music." PloS one **4**(7): e6230.

Lesiuk, T. (2008). "The effect of preferred music listening on stress levels of air traffic controllers." The Arts in Psychotherapy **35**(1): 1-10.

Lesiuk, T. (2010). "The effect of preferred music on mood and performance in a high-cognitive demand occupation." Journal of music therapy **47**(2): 137-154.

Lesta, B. and P. Petocz (2006). "Familiar Group Singing: Addressing Mood and Social Behaviour of Residents with Dementia Displaying Sundowning." Australian Journal of Music Therapy **17**.

Lord, V. M., et al. (2012). "Singing classes for chronic obstructive pulmonary disease: a randomized controlled trial." BMC Pulmonary Medicine **12**(1): 1-7.

Lotter, C. B. (2006). Circles of Courage: music therapy with adolescents in conflict with the law at a community based setting, University of Pretoria.

Louhivuori, J., et al. (2005). "'Singing Together': A Crosscultural Approach to the Meaning of Choirs As a Community." Cultural diversity in music education: 81.

Macpherson, C. F. (2008). "Peer-supported storytelling for grieving pediatric oncology nurses." Journal of Pediatric Oncology Nursing **25**(3): 148-163.

McCaffrey, R. and E. Freeman (2003). "Effect of music on chronic osteoarthritis pain in older people." Journal of advanced nursing **44**(5): 517-524.

McCaffrey, R. and R. Locsin (2006). "The effect of music on pain and acute confusion in older adults undergoing hip and knee surgery." Holistic nursing practice **20**(5): 218-224.

Menon, V. and D. J. Levitin (2005). "The rewards of music listening: response and physiological connectivity of the mesolimbic system." Neuroimage **28**(1): 175-184.

Miskovic, D., et al. (2008). "Randomized controlled trial investigating the effect of music on the virtual reality laparoscopic learning performance of novice surgeons." Surgical endoscopy **22**(11): 2416-2420.

Mitterschiffthaler, M. T., et al. (2007). "A functional MRI study of happy and sad affective states induced by classical music." Human brain mapping **28**(11): 1150-1162.

Mizuno, T. and M. Sugishita (2007). "Neural correlates underlying perception of tonality-related emotional contents." Neuroreport **18**(16): 1651-1655.

Mok, E. and K.-Y. Wong (2003). "Effects of music on patient anxiety." AORN journal **77**(2): 396-410.

Mutschler, I., et al. (2010). "Time scales of auditory habituation in the amygdala and cerebral cortex." Cerebral Cortex **20**(11): 2531-2539.

Nakamura, S., et al. (1999). "Analysis of music–brain interaction with simultaneous measurement of regional cerebral blood flow and electroencephalogram beta rhythm in human subjects." Neuroscience letters **275**(3): 222-226.

Ohnishi, T., et al. (2001). "Functional anatomy of musical perception in musicians." Cerebral Cortex **11**(8): 754-760.

Okada, K., et al. (2009). "Effects of music therapy on autonomic nervous system activity, incidence of heart failure events, and plasma cytokine and catecholamine levels in elderly patients with cerebrovascular disease and dementia." International heart journal **50**(1): 95-110.

Pallesen, K. J., et al. (2005). "Emotion processing of major, minor, and dissonant chords: a functional magnetic resonance imaging study." Annals of the New York Academy of Sciences **1060**(1): 450-453.

Pallesen, K. J., et al. (2009). "Cognitive and emotional modulation of brain default operation." Journal of cognitive neuroscience **21**(6): 1065-1080.

Pallesen, K. J., et al. (2010). "Cognitive control in auditory working memory is enhanced in musicians." PloS one **5**(6): e11120.

Pickles, V. (2003). "Music and the third age." Psychology of music **31**(4): 415-423.

Potash, J. S., et al. (2014). "Can art therapy reduce death anxiety and burnout in end-of-life care workers? A quasi-experimental study." International journal of palliative nursing **20**(5): 233-240.

Rice, K. L., et al. (2014). "Using second life to facilitate peer storytelling for grieving oncology nurses." Ochsner Journal **14**(4): 551-562.

Salzano, A. T., et al. (2013). "The effectiveness of a collaborative art-making task on reducing stress in hospice caregivers." The Arts in Psychotherapy **40**(1): 45-52.

Sands, S. A., et al. (2008). "Pediatric narrative oncology: interprofessional training to promote empathy, build teams, and prevent burnout." J Support Oncol **6**(7): 307-312.

Särkämö, T., et al. (2014). "Cognitive, emotional, and social benefits of regular musical activities in early dementia: randomized controlled study." The Gerontologist **54**(4): 634-650.

Satoh, M., et al. (2001). "Activated brain regions in musicians during an ensemble: a PET study." Cognitive Brain Research **12**(1): 101-108.

Shih, Y.-N., et al. (2016). "Elucidating the relationship between work attention performance and emotions arising from listening to music." Work **55**(2): 489-494.

Shih, Y.-N., et al. (2009). "Correlation between work concentration level and background music: A pilot study." Work **33**(3): 329-333.

Shih, Y.-N., et al. (2012). "Background music: Effects on attention performance." Work **42**(4): 573-578.

Shimizu, N., et al. (2013). "Effects of movement music therapy with the Naruko clapper on psychological, physical and physiological indices among elderly females: a randomized controlled trial." Gerontology **59**(4): 355-367.

Silber, L. (2005). "Bars behind bars: the impact of a women's prison choir on social harmony." Music education research **7**(2): 251-271.

Skingley, A. and H. Bungay (2010). "The Silver Song Club Project: singing to promote the health of older people." British journal of community nursing **15**(3): 135-140.

Snow, S. and M. D'Amico (2010). "The drum circle project: A qualitative study with at-risk youth in a school setting." Canadian Journal of Music Therapy **16**(1).

Solé, C., et al. (2010). "Contributions of music to aging adults' quality of life." Journal of music therapy **47**(3): 264-281.

Speer, S. (2011). "Effect of Background Music, Speech and Silence on Office Workers' Selective Attention."

Suda, M., et al. (2008). "Cortical responses to Mozart's sonata enhance spatial-reasoning ability." Neurological Research **30**(9): 885-888.

Sun, J. and N. Buys (2013). "Participatory community singing program to enhance quality of life and social and emotional well-being in Aboriginal and Torres Strait Islander Australians with chronic diseases." International Journal on Disability and Human Development **12**(3): 317-323.

Sun, J., et al. (2013). "The role of Tai Chi, cultural dancing, playing a musical instrument and singing in the prevention of chronic disease in Chinese older adults: A mind–body meditative approach." International Journal of Mental Health Promotion **15**(4): 227-239.

Sung, H. C., et al. (2010). "A preferred music listening intervention to reduce anxiety in older adults with dementia in nursing homes." Journal of Clinical Nursing **19**(7-8): 1056-1064.

Suzuki, M., et al. (2005). "Behavioral, stress and immunological evaluation methods of music therapy in elderly patients with senile dementia." Nihon Ronen Igakkai zasshi. Japanese journal of geriatrics **42**(1): 74-82.

Thaut, M. H., et al. (2009). "Distinct cortico-cerebellar activations in rhythmic auditory motor synchronization." Cortex **45**(1): 44-53.

Twiss, E., et al. (2006). "The effect of music listening on older adults undergoing cardiovascular surgery." Nursing in critical care **11**(5): 224-231.

Tyson, E. H. (2002). "Hip hop therapy: An exploratory study of a rap music intervention with at-risk and delinquent youth." Journal of Poetry Therapy **15**(3): 131-144.

Warth, M., et al. (2015). "Music therapy in palliative care: A randomized controlled trial to evaluate effects on relaxation." Deutsches Ärzteblatt International **112**(46): 788.

Wlodarczyk, N. (2007). "The effect of music therapy on the spirituality of persons in an in-patient hospice unit as measured by self-report." Journal of music therapy **44**(2): 113-122.

Wlodarczyk, N. (2013). "The effect of a group music intervention for grief resolution on disenfranchised grief of hospice workers." Progress in palliative care **21**(2): 97-106.

Wlodarczyk, N. M. (2010). "Effect of a Single-Session Music Therapy Group Intervention for Grief Resolution on the Disenfranchised Grief of Hospice Workers."

Woodward, S. C., et al. (2007). "South Africa, the arts and youth in conflict with the law." International Journal of Community Music **1**(1): 69-88.

WU, S. M. (2002). "Effects of music therapy on anxiety, depression and self-esteem of undergraduates." Psychologia **45**(2): 104-114.

Included studies – Non-communicable disease risk

Balbag, M. A., et al. (2014). "Playing a musical instrument as a protective factor against dementia and cognitive impairment: A population-based twin study." International Journal of Alzheimer’s Disease **2014**.

Bygren, L. O., et al. (1996). "Attendance at cultural events, reading books or periodicals, and making music or singing in a choir as determinants for survival: Swedish interview survey of living conditions." BMJ **313**(7072): 1577-1580.

Chen, M., et al. (2013). "Different physical activity subtypes and risk of metabolic syndrome in middle-aged and older Chinese people." PloS one **8**(1): e53258.

Ekholm, O. and L. O. Bonde (2018). Music and Health in Everyday Life in Denmark: Associations Between the Use of Music and Health-Related Outcomes in Adult Danes. Music and Public Health, Springer**:** 15-31.

Hughes, T. F., et al. (2010). "Engagement in reading and hobbies and risk of incident dementia: the MoVIES project." American Journal of Alzheimer's Disease & Other Dementias® **25**(5): 432-438.

Konlaan, B. B., et al. (2000). "Visiting the cinema, concerts, museums or art exhibitions as determinant of survival: a Swedish fourteen-year cohort follow-up." Scandinavian Journal of Public Health **28**(3): 174-178.

Lin, W., et al. (2019). "Performing different kinds of physical exercise differentially attenuates the genetic effects on obesity measures: Evidence from 18,424 Taiwan Biobank participants." PLoS Genetics **15**: e1008277.

Merom, D., et al. (2016). "Dancing participation and cardiovascular disease mortality: a pooled analysis of 11 population-based British cohorts." American journal of preventive medicine **50**(6): 756-760.

Verghese, J., et al. (2003). "Leisure activities and the risk of dementia in the elderly." New England Journal of Medicine **348**(25): 2508-2516.

Included studies – Heart Rate, Dance

Aygün, C., et al. (2018). "Active video dancing game provides high-intensity exercise for hip-hop dancers and non-dancers." South African Journal for Research in Sport, Physical Education and Recreation **40**(2): 1-10.

Baillie, Y., et al. (2007). "Highland dance: heart-rate and blood lactate differences between competition and class." International Journal of Sports Physiology and Performance **2**(4): 371-376.

Beck, S., et al. (2018). "Changes in Energy Demand of Dance Activity and Cardiorespiratory Fitness During 1 Year of Vocational Contemporary Dance Training." The Journal of Strength & Conditioning Research **32**(3): 841-848.

Blanksby, B. and P. Reidy (1988). "Heart rate and estimated energy expenditure during ballroom dancing." British Journal of Sports Medicine **22**(2): 57-60.

Bria, S., et al. (2011). "Physiological characteristics of elite sport-dancers." The Journal of sports medicine and physical fitness **51**(2): 194-203.

Bronner, S., et al. (2016). "Physiological and psychophysiological responses to an exer-game training protocol." Journal of science and medicine in sport **19**(3): 267-271.

Bronner, S., et al. (2013). "Energy cost and game flow of 5 exer-games in trained players." American journal of health behavior **37**(3): 369-380.

Bronstein, M., et al. (1990). "Physiological response to low-impact aerobic dance by trained dancers with and without handheld weights." Ann. Sports Med **5**: 74-77.

Çakir-Atabek, H., et al. (2020). "Active Video Games versus Traditional Exercises: Energy Expenditure and Blood Lactate Responses." Research quarterly for exercise and sport **91**(2): 188-196.

Canabrava, K. L., et al. (2018). "Energy expenditure and intensity of active video games in children and adolescents." Research quarterly for exercise and sport **89**(1): 47-56.

Carroll, M. W., et al. (1991). "The metabolic cost of two ranges of arm position height with and without hand weights during low impact aerobic dance." Research quarterly for exercise and sport **62**(4): 420-423.

Cohen, J. L., et al. (1982). "Cardiorespiratory responses to ballet exercise and the VO2max of elite ballet dancers." Medicine and Science in Sports and Exercise **14**(3): 212-217.

Cugusi, L., et al. (2015). "A New Type of Physical Activity from an Ancient Tradition: The Sardinian Folk Dance “Ballu Sardu”." Journal of dance medicine & science **19**(3): 118-123.

Darby, L. A., et al. (1995). "The effects of cadence, impact, and step on physiological responses to aerobic dance exercise." Research quarterly for exercise and sport **66**(3): 231-238.

De Angelis, M., et al. (1998). "Oxygen uptake, heart rate and blood lactate concentration during a normal training session of an aerobic dance class." European journal of applied physiology and occupational physiology **78**(2): 121-127.

Delextrat, A. and E. Neupert (2016). "Physiological load associated with a Zumba® fitness workout: a comparison pilot study between classes and a DVD." Journal of sports sciences **34**(1): 47-55.

Domene, P. A. and C. Easton (2014). "Combined triaxial accelerometry and heart rate telemetry for the physiological characterization of Latin dance in non-professional adults." Journal of dance medicine & science **18**(1): 29-36.

Eason, J. M., et al. (2016). "A Comparison of Energy Expenditure and Heart Rate Response Between a Dance-Based Group Fitness Class and a Dance-Based Video Game on the Xbox Kinect." Cardiopulmonary Physical Therapy Journal **27**(2): 62-67.

Emerenziani, G. P., et al. (2013). "Exercise intensity and gender difference of 3 different salsa dancing conditions." Int J Sports Med **34**(4): 330-335.

Fawkner, S. G., et al. (2010). "Adolescent girls' energy expenditure during dance simulation active computer gaming." Journal of sports sciences **28**(1): 61-65.

Forte, R., et al. (2001). "Cardiovascular response during low-intensity step-aerobic dance in middle-aged subjects." European Journal of Sport Science **1**(3): 1-7.

Gao, Z., et al. (2009). "Middle School Student's Heart Rates during Different Curricular Activities in Physical Education." ICHPER-SD Journal of Research **4**(1): 21-24.

Giancotti, G. F., et al. (2018). "Energy expenditure and perceived exertion during active video games in relation to player mode and gender." Kinesiology **50**(1): 18-24.

Graf, D. L., et al. (2009). "Playing active video games increases energy expenditure in children." Pediatrics **124**(2): 534-540.

Grant, S., et al. (2002). "A comparison of physiological responses and rating of perceived exertion in two modes of aerobic exercise in men and women over 50 years of age." British Journal of Sports Medicine **36**(4): 276-281.

Grier, T. D., et al. (2002). "Metabolic cost of aerobic dance bench stepping at varying cadences and bench heights." Journal of strength and conditioning research **16**(2): 242-249.

Heil, D. P., et al. (2019). "The Energy Expenditure of Tinikling: A Culturally Relevant Filipino Dance." International Journal of Exercise Science **12**(4): 111.

Kraft, J. A., et al. (2011). "Heart rate and perceived exertion during self-selected intensities for exergaming compared to traditional exercise in college-age participants." The Journal of Strength & Conditioning Research **25**(6): 1736-1742.

La Torre, A., et al. (2005). "Cardiovascular responses to aerobic step dance sessions with and without appendicular overload." Journal of sports medicine and physical fitness **45**(3): 264.

Léger, L. A. (1982). "Energy cost of disco dancing." Research quarterly for exercise and sport **53**(1): 46-49.

Liiv, H., et al. (2014). "Physiological characteristics of elite dancers of different dance styles." European Journal of Sport Science **14**(sup1): S429-S436.

Lin, J.-H. (2015). "“Just Dance”: The effects of exergame feedback and controller use on physical activity and psychological outcomes." Games for Health Journal **4**(3): 183-189.

Lowe, A., et al. (2010). "Accuracy of polar F6 in estimating the energy cost of aerobic dance bench stepping in college-age females." Journal of sports medicine and physical fitness **50**(4): 385.

Maciejczyk, M. and A. Feć (2013). "Evaluation of aerobic capacity and energy expenditure in folk dancers." Human movement **14**(1): 76-81.

Marks, D. W., et al. (2015). "Greater physiological responses while playing XBox Kinect compared to Nintendo Wii." International Journal of Exercise Science **8**(2): 7.

Nelson, L., et al. (2011). "Heart rates of elementary physical education students during the dancing classrooms program." Research quarterly for exercise and sport **82**(2): 256-263.

Nicholas, J. C., et al. (2019). "Pole Dancing for Fitness: The Physiological and Metabolic Demand of a 60-Minute Class." The Journal of Strength & Conditioning Research **33**(10): 2704-2710.

Oh, Y., et al. (2017). "Comparisons of Heart Rate and Energy Expenditure During Exergaming in College-age Adults." International Journal of Computer Science in Sport **16**(3): 197-206.

Oliveira, S. M., et al. (2010). "Physiological responses to a tap dance choreography: comparisons with graded exercise test and prescription recommendations." The Journal of Strength & Conditioning Research **24**(7): 1954-1959.

Pennington, C. G. and L. P. Nelson (2020). "Physical Activity Contributions of Dancing Classrooms Program on Middle School Students." The Physical Educator **77**(2).

Pilch, W., et al. (2017). "Energy expenditure and lactate concentration in sports dancers in a simulated final round of the standard style competition." Human movement **18**(2): 62-67.

Powers, M. M., et al. (2016). "A Comparison of Energy Expenditure Across 3 Gaming Platforms Using an Activity-Based Dance Game." Cardiopulmonary Physical Therapy Journal **27**(4): 108-114.

Rauber, S. B., et al. (2018). "Oxygen consumption and energy expenditure during and after street games, active video games and tv." Revista Brasileira de Medicina do Esporte **24**(5): 338-342.

Rixon, K. P., et al. (2006). "Analysis of the assessment of caloric expenditure in four modes of aerobic dance." Journal of strength and conditioning research **20**(3): 593.

Rockefeller, K. A. and E. Burke (1979). "Psycho-physiological analysis of an aerobic dance programme for women." British Journal of Sports Medicine **13**(2): 77-80.

Rodrigues-Krause, J., et al. (2014). "Oxygen consumption and heart rate responses to isolated ballet exercise sets." Journal of dance medicine & science **18**(3): 99-105.

Rodrigues-Krause, J., et al. (2018). "Cardiorespiratory responses of a dance session designed for older women: A cross sectional study." Experimental gerontology **110**: 139-145.

Rodrigues-Krause, J., et al. (2014). "Ballet dancers cardiorespiratory, oxidative and muscle damage responses to classes and rehearsals." European Journal of Sport Science **14**(3): 199-208.

Rousanoglou, E. and K. Boudolos (2005). "Ground reaction forces and heart rate profile of aerobic dance instructors during a low and high impact exercise programme." Journal of sports medicine and physical fitness **45**(2): 162.

Sanders, D. J., et al. (2019). "Training Demands and Physiological Profile of Cross-Disciplined Collegiate Female Dancers." Journal of strength and conditioning research.

Schaefeer, S. A., et al. (1995). "Perceived exertion and metabolic responses of women during aerobic dance exercise." Perceptual and motor skills **81**(2): 691-700.

Schaeffer-Gerschutz, S. A., et al. (2000). "Differentiated ratings of perceived exertion and physiological responses during aerobic dance steps by impact/type of arm movement." Perceptual and motor skills **90**(2): 457-471.

Schneekloth, B. and G. A. Brown (2018). "Comparison of physical activity during Zumba with a human or video game instructor." International Journal of Exercise Science **11**(4): 1019.

Sekulic, D., et al. (2006). "Students'-instructors' physiological responses during step aerobic exercise." Biology of Sport **23**(2): 171.

Seliger, V., et al. (1970). "Evaluation of stage artist's activities on basis of telemetrical measurements of heart rates." Internationale Zeitschrift für angewandte Physiologie einschließlich Arbeitsphysiologie **28**(2): 86-104.

Sell, K., et al. (2008). "Energy expenditure during physically interactive video game playing in male college students with different playing experience." Journal of American College Health **56**(5): 505-512.

Smallwood, S. R., et al. (2012). "Physiologic responses and energy expenditure of kinect active video game play in schoolchildren." Archives of pediatrics & adolescent medicine **166**(11): 1005-1009.

Stephens, N. and M. Wyon (2020). "Physiological Characteristics of Musical Theatre Performers and the Effect on Cardiorespiratory Demand Whilst Singing and Dancing." Medical Problems of Performing Artists **35**(1): 54-58.

Strejcová, B., et al. (2013). "The energetic cost and cardiorespiratory changes during Zumba exercise in middle aged women " International Journal of Sports Sciences & Fitness **3**(2).

Tan, B., et al. (2002). "Aerobic demands of the dance simulation game." International journal of sports medicine **23**(2): 125-129.

Tarrant, K. and L. McNaughton (1997). "A comparison of the metabolic effects of high and low impact aerobic dance exercise." Research in Sports Medicine: An International Journal **7**(3-4): 255-264.

Thompson, W. R., et al. (1991). "The effect of hand-held weights on the physiological responses to aerobic dance." The Journal of Strength & Conditioning Research **5**(4): 208-212.

Thomsen, D. and D. L. Ballor (1991). "Physiological responses during aerobic dance of individuals grouped by aerobic capacity and dance experience." Research quarterly for exercise and sport **62**(1): 68-72.

Unnithan, V. B., et al. (2006). "Evaluation of the energy cost of playing a dance simulation video game in overweight and non-overweight children and adolescents." International journal of sports medicine **27**(10): 804-809.

Usagawa, T., et al. (2014). "Metabolic equivalent determination in the cultural dance of hula." International journal of sports medicine **35**(5): 399.

Ušpurienė, A. B. and A. Čepulėnas (2012). "Physical Load Intensity in Standard and Latin American Sports Dancing Programmes for Juvenile Dancers." Baltic Journal of Sport and Health Sciences **2**(85).

Wigaeus, E. (1980). "Physical demands during folk dancing." European journal of applied physiology and occupational physiology **45**(2-3): 177-183.

Wyon, M., et al. (2002). "The cardiorespiratory responses to modern dance classes: differences between university, graduate, and professional classes." Journal of dance medicine & science **6**(2): 41-45.

Wyon, M. A., et al. (2004). "Oxygen uptake during modern dance class, rehearsal, and performance." The Journal of Strength & Conditioning Research **18**(3): 646-649.

Wyon, M. A., et al. (2018). "Cardiorespiratory profile and performance demands of elite hip-hop dancers: breaking and new style." Medical Problems of Performing Artists **33**(3): 198-204.

Zhu, W., et al. (2018). "Characterizing the Aerobic and Anaerobic Energy Costs of Polynesian Dances." International Journal of Exercise Science **11**(4): 1156.

Included studies – Heart Rate, Music

Barr, D., et al. (2000). "Physiological assessment of bagpipers: a preliminary study of cardiopulmonary parameters." Medical Problems of Performing Artists **15**(2): 51-55.

De La Rue, S., et al. (2013). "Energy expenditure in rock/pop drumming." International journal of sports medicine **34**(10): 868-872.

Dye, D. and V. Barry (2016). "The Effect of Metronome Use on Heart Rates and RPE for Drum Corps Percussionists." International Journal of Exercise Science **9**(4): 3.

Erdmann, L. D., et al. (2003). "Adolescents' energy cost in marching band." Perceptual and motor skills **97**(2): 639-646.

Hahnengress, M. L. and D. Böning (2010). "Cardiopulmonary changes during clarinet playing." European journal of applied physiology **110**(6): 1199-1208.

Harmat, L. and T. Theorell (2010). "Heart rate variability during singing and flute playing." Music and Medicine **2**(1): 10-17.

Horswill, C., et al. (1995). "Energy expenditure in adolescents during low intensity, leisure activities." Medicine & Science in Sports & Exercise.

Hunsaker, L. A. (1994). "Heart rate and rhythm responses during trumpet playing." Medical Problems of Performing Artists **9**: 69-69.

Iñesta, C., et al. (2008). "Heart rate in professional musicians." Journal of Occupational Medicine and Toxicology **3**(1): 1-11.

Mulcahy, D., et al. (1990). "Circadian variation of heart rate is affected by environment: a study of continuous electrocardiographic monitoring in members of a symphony orchestra." Heart **64**(6): 388-392.

Robertson, D. U., et al. (2010). "Cardiac response during trumpet playing." Medical Problems of Performing Artists **25**(1): 16-21.

Romero, B., et al. (2016). "Metabolic demands of heavy metal drumming." International Journal of Kinesiology and Sports Science **4**(3): 32-36.

Seliger, V., et al. (1970). "Evaluation of stage artist's activities on basis of telemetrical measurements of heart rates." Internationale Zeitschrift für angewandte Physiologie einschließlich Arbeitsphysiologie **28**(2): 86-104.

Strand, B. and C. Sommer (2005). "Should marching band be allowed to replace physical education credits: An analysis." The Physical Educator **62**(3).

Studer, R. K., et al. (2014). "Psychophysiological activation during preparation, performance, and recovery in high-and low-anxious music students." Applied Psychophysiology and Biofeedback **39**(1): 45-57.

Vellers, H. L., et al. (2015). "Heart rate response of professional musicians when playing music." Medical Problems of Performing Artists **30**(2): 100-105.
